# Mechanistic Investigation of [Co_2_(CO)_8_] Catalyzed Photoaminocarbonylation

**DOI:** 10.1021/jacs.6c04362

**Published:** 2026-06-18

**Authors:** Rowan M. Bailey, Bernd Schaefer, Mark R. Crimmin, Philip W. Miller

**Affiliations:** † Department of Chemistry, 4615 Imperial College London, W12 0BZ London, U.K.; ‡ BASF SE, Ludwigshafen am Rhein 67056, Rhineland-Palatinate, Germany

## Abstract

Recent advances in cobalt-photocatalyzed carbonylation methods offer an efficient synthetic route to a range of amides and esters. Although preliminary studies provide important mechanistic insights, the kinetics of these reactions have not been studied in detail. Herein, we report an in-depth mechanistic investigation into the [Co_2_(CO)_8_]-catalyzed aminocarbonylation of aryl halides. Rarely used ^59^Co NMR spectroscopy provided evidence for [Co­(CO)_4_]^−^ as an on-cycle catalytic intermediate. Orders in reagents, Hammett analysis, and Eyring analysis gave unique insight into both photon-limited and photon-unlimited regimes. Data strongly suggested that the turnover-limiting step of catalysis is the reaction of [Co­(CO)_4_]^−^ with the aryl bromide. Plausible mechanisms for this step were considered and differentiated by experimental data, including quantum yield measurements, radical probes, and competition experiments. It is concluded that this step most likely occurs through a light-promoted nucleophilic attack of the cobalt anion on the aryl bromide. In turn, this knowledge was used to expand catalysis to more challenging reagents such as aryl chlorides, low nucleophilicity amines, and acyclic vinyl halides. Enhanced catalyst efficiency (TON and TOF improved >10-fold) was also achieved for established substrates, strengthening the industrial use case of this technology.

## Graphical abstract

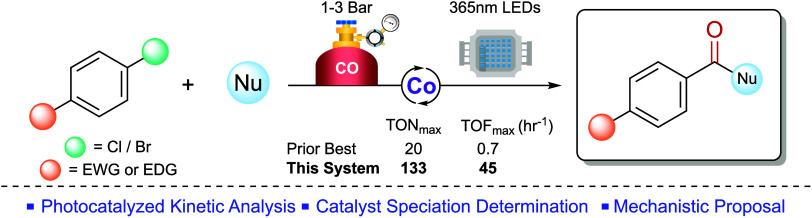

## Introduction

Amide groups are ubiquitous across drug discovery and agrochemical sectors. In 2023, the amide motif was present in 71 of the top 100 small molecule drugs.^1^ In 2018, amide synthesis through catalytic, atom-economical, and low-waste routes was named the second most important challenge for modern chemistry by industrial leaders.^2^ Yet, years later, the challenges surrounding sustainable amide synthesis still prevail. In practice, the synthesis of amides in process routes is often still reliant on atom-inefficient stoichiometric coupling agents or toxic chlorinating reagents such as PCl_5_ and SOCl_2_.^3–8^ While Pd-catalyzed aminocarbonylation of aryl or alkyl halides represents an attractive alternative, the scarcity of the metal,^9^ cost of ligands,^10^ and reliance on toxic solvents^11^ can limit the scalability of this approach. Further, the propensity for these catalysts to undergo β-hydride elimination side reactions has confined the scope for these methods to a narrow range of substrates.^12^

Photocatalysis offers a potential route to address these challenges.^13^ Within the past few years, photocatalyzed carbonylation has emerged as an exciting strategy for the synthesis of amides and related functional groups.^14^ Many of the early photocatalyzed methods used Pd-based catalysts for the transformation,^15–18^ although more recent work has demonstrated catalysts based on first-row metals, Co,^19,20^ Cu,^21^ Ni,^22^ and Mn,^23,24^ as suitable candidates. Of these systems, the [Co_2_(CO)_8_]-catalyzed aminocarbonylation of aryl halides reported by the Alexanian group posed a mild, generally applicable system that can operate at low pressures of CO (2 bar, Figure 1).^20^

**Figure 1:**
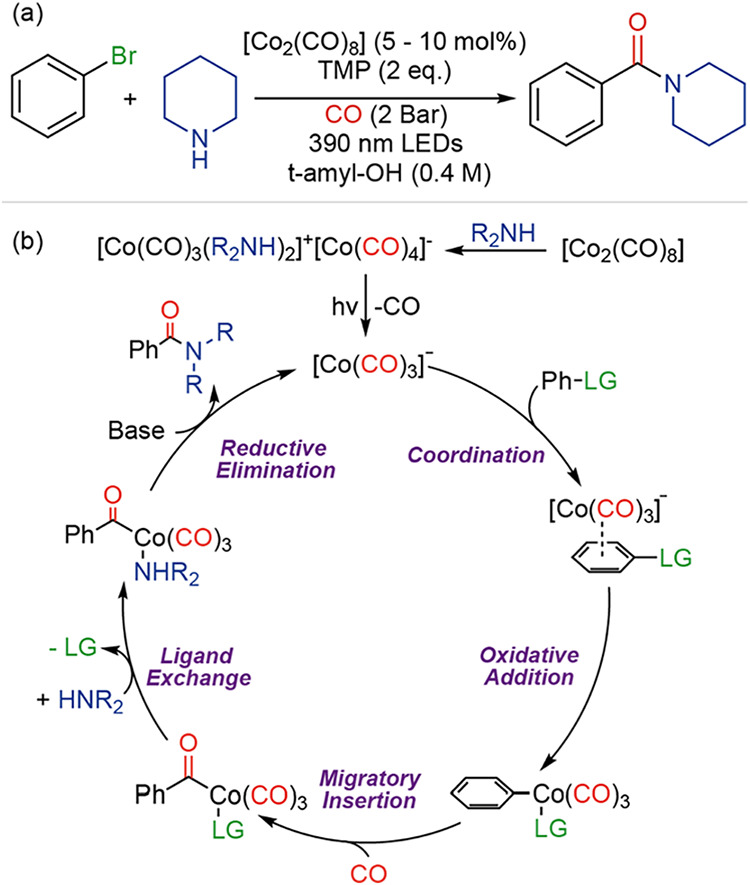
(a) [Co_2_(CO)_8_]-catalyzed aminocarbonylation conditions presented by the Alexanian group.^20^ (b) A recent proposed catalyst scheme is shown.^25^ Cation species [Co­(CO)_3_(R_2_NH)_2_]^+^ is omitted in catalyst cycle for clarity.

While detailed and impressive mechanistic investigations have been conducted for thermal Pd-catalyzed aminocarbonylation reactions,^26^ understanding of photocatalytic carbonylation methods is still in its infancy.^25,27–29^ This gap in knowledge aligns with common challenges encountered in photocatalysis, which at times can lack systematic understanding and reproducibility due to sensitivity of reaction rates to the experimental setup (e.g., light source, reactor design).^30^ Novel methods have begun to address these challenges, providing access to valuable kinetic information via tightly controlled photon delivery^31^ and *in situ* spectroscopic analysis (IR, NMR, EPR).^32,33^

Herein, we report a detailed kinetic study of a [Co_2_(CO)_8_]-photocatalyzed aminocarbonylation process. Orders in reagents and catalyst are determined, alongside a Hammett and Eyring study. Quadrupolar ^59^Co NMR spectroscopy has been applied to understand catalyst initiation, with the findings supported by IR spectroscopy. In combination, this approach has led to a deeper mechanistic understanding of [Co_2_(CO)_8_]-photocatalyzed aminocarbonylation. This has enabled a >10-fold improvement to turnover number (TON) and turnover frequency (TOF) for this system over prior literature, as well as an expansion of scope to include challenging reaction partners such as acyclic vinyl halides, electron-rich aryl chlorides, and weak nucleophiles, including phenol and aniline.

## Results

### Optimization of Photocatalysis

An Asynt Illumin8 photoreactor modified with an adapted CO inlet was used to investigate the carbonylation of *p*-bromoanisole with piperidine in the presence of 2,2,6,6-tetramethylpiperidine (TMP, 2 equiv). At 1 bar CO pressure, a modest yield (27%) of amide was obtained after 48 h, lower than the 58% reported in the literature at the same pressure.^20^ Increasing the reaction volume (from 1 to 2 mL), improving temperature control, and modifying aryl bromide concentration to 0.5 M gave consistently high yields of 97%. Even under these optimized reaction conditions, reaction times were long, and reaction mixtures were heterogeneous. Due to the sensitivity of photochemical reactions to light-scattering effects, it was imperative that a homogeneous system was used to ensure reproducibility for studying the kinetics. Variation of the base was investigated under optimal reaction conditions to solve these issues (Figure 2). The replacement of TMP with DBU improved the reaction efficiency, allowing for catalyst loadings as low as 1 mol % [Co_2_(CO)_8_] while maintaining excellent yields. Achieving such low loadings is a necessary step for potential commercial applications. Importantly, the reactions conducted with DBU were homogeneous, giving greater confidence in the consistency of the photon delivery to the reaction mixture.

**Figure 2:**
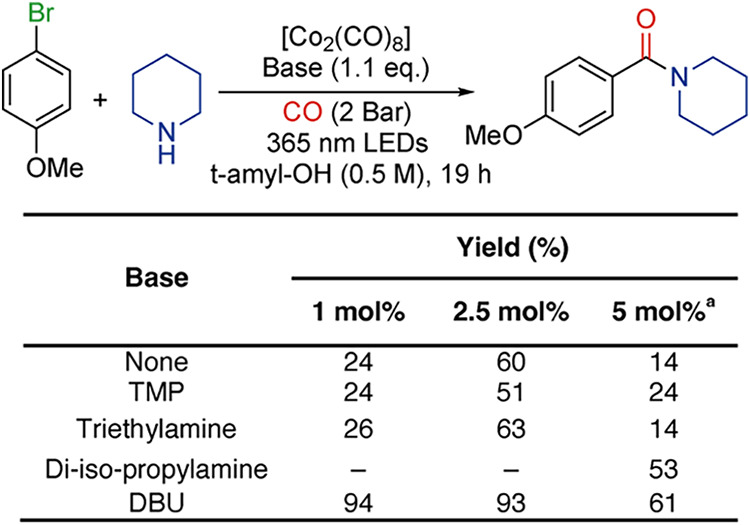
Photocatalyzed aminocarbonylation of *p*-bromoanisole with piperidine (1.3 equiv) with varied bases (1.1 equiv). Yields determined by GC-FID. ^a^5.5 h reaction time. TMP = 2,2,6,6-Tetramethylpiperidine. DBU = 1,8-Diazabicyclo[5.4.0]­undec-7-ene.

### Catalyst Initiation

To gain further insight into the catalyst initiation events, reactants were combined in the presence and absence of light and mixtures were analyzed by IR and NMR spectroscopy. Combining [Co_2_(CO)_8_], aryl bromide, piperidine, and DBU in the absence of light (365 nm LED) or CO gave rise to a near complete loss of the IR vibrations associated with [Co_2_(CO)_8_], with clear formation of an IR stretch at 1876 cm^–1^ assigned to [Co­(CO)_4_]^−^.^34,35^

These data suggest that precatalyst initiation, to form [Co­(CO)_4_]^−^, is facile even in the absence of light. Previous mechanistic studies have suggested that [Co­(CO)_4_]^−^ is a key intermediate for carbonylation of aryl bromides catalyzed by [Co_2_(CO)_8_] and by mixtures of [Co­(OAc)_2_], NaH, and *t*-amyl–OH.^36,37^ In contrast, the reaction of [Co_2_(CO)_8_] with aryl bromide showed little change in the carbonyl region of the IR spectrum; only upon addition of DBU was consumption of [Co_2_(CO)_8_] observed with formation of a new IR stretch assigned to [Co­(CO)_4_]^−^ (Figure 3a).^38^

**Figure 3:**
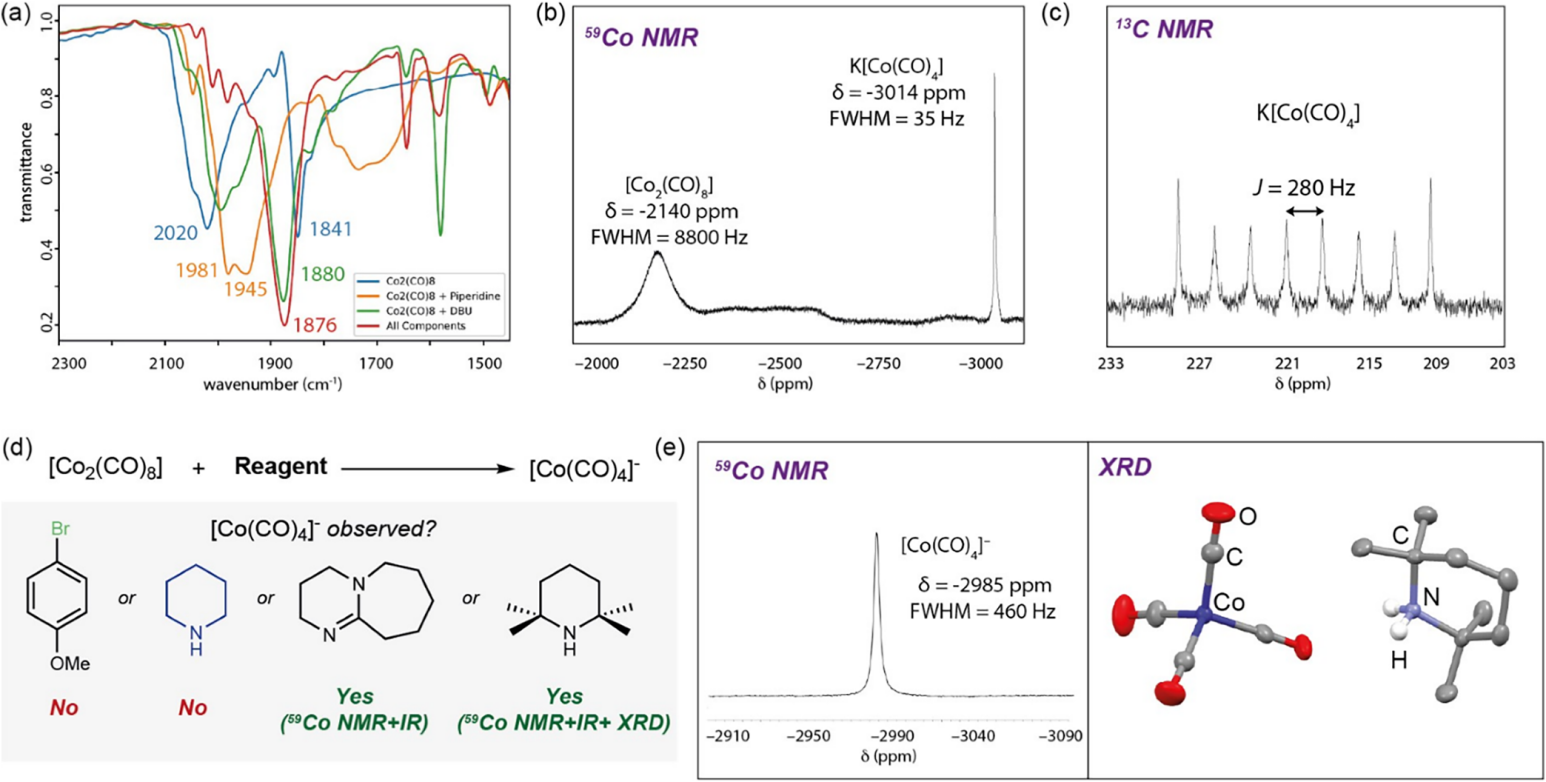
(a) IR spectroscopy analysis of stoichiometric addition of [Co_2_(CO)_8_] to reaction components. (b) Superimposed ^59^Co NMR spectra of [Co_2_(CO)_8_] and K­[Co­(CO)_4_]. (c) ^13^C NMR analysis of K­[Co­(CO)_4_]. (d) Spectroscopic investigation of precatalyst initiation. (e) Experimentally observed ^59^Co NMR spectra for [TMPH]^+^[Co­(CO)_4_]^−^ along with the crystal structure obtained directly from the reaction mixture.^39^ Prior work suggested reaction of [Co_2_(CO)_8_] with piperidine in *n*-hexane may form the [Co­(CO)_4_]^−^ anion based on IR spectroscopy.^40^

^59^Co NMR spectroscopy was used to provide further insight into catalyst initiation. ^59^Co (*I* = 7/2, 100%) is an NMR active nucleus with a chemical shift range of approximately 20,000 ppm. Though the technique is rarely reported, it provided an opportunity to observe the metal center directly. For this system, it was expected that the high symmetry of the [Co­(CO)_4_]^−^ would lead to a minimal electric field gradient across the molecule and limit line broadening known to be problematic for quadrupolar nuclei. K­[Co­(CO)_4_] was synthesized and analyzed by ^59^Co and ^13^C NMR spectroscopy.^39^ The ^59^Co NMR spectrum in THF-*d*_8_ gave a remarkably sharp NMR signal at δ = −3014 ppm (FWHM = 35.3 Hz) (Figure 3b). ^13^C NMR spectroscopy shows the expected octet splitting pattern due to a single CO environment coupling to the *I* = 7/2 ^59^Co nucleus (Figure 3c). In contrast, [Co_2_(CO)_8_] showed a broad peak in the ^59^Co spectra, centered at δ = −2140 ppm (FWHM = 8800 Hz) at 80 °C in C_6_D_6_ (Figure 3b).

Using ^59^Co NMR spectroscopy, the consumption of [Co_2_(CO)_8_] and the formation of [Co­(CO)_4_]^−^ could be tracked (Figure 3b). Independent reactions of [Co_2_(CO)_8_] with either piperidine or (trifluoromethyl)­bromobenzene did not lead to definitive formation of the sharp resonance associated with [Co­(CO)_4_]^–^. In the case of piperidine, analysis of this reaction mixture by IR spectroscopy revealed stretches that have previously been assigned to an acyl-cobalt intermediate; however, this species has not been definitively characterized and, in our hands, could not be isolated.^40,41^

Importantly, there was minimal evidence for cobaltate formation, even upon addition of excess piperidine. Irradiation (365 nm LED) of these samples led to little change. In contrast, the reaction of [Co_2_(CO)_8_] with TMP led to the formation of a sharp resonance δ = −2985 ppm (FWHM = 460 Hz) (Figure 3d). Similarly, the addition of DBU to [Co_2_(CO)_8_] produced two resonances at δ = −2964 ppm (FWHM = 233 Hz) and δ = −2982 ppm (FWHM = 255 Hz), consistent with the formation of two distinct [Co­(CO)_4_]^−^ anions, likely differing in the nature of the cation. Further titration of excess DBU into these samples shifted the distribution toward the product with the most upfield resonance. Ultimately, the assignment of these ^59^Co NMR data was confirmed through isolation and crystallographic characterization of the novel ammonium complex [TMPH]^+^[Co­(CO)_4_]^−^ formed from direct reaction of [Co_2_(CO)_8_] with excess TMP (1:20) in C_6_D_6_ (Figure 3d).

Prior work has used UV–vis spectroscopy to suggest that the aryl bromide and [Co­(CO)_4_]^−^ form an encounter complex in solution as a first step of catalysis. Consistent with this suggestion, the reaction of K­[Co­(CO)_4_] with *p*-(trifluoromethyl)­bromobenzene led to the disappearance of the sharp resonance associated with the tetrahedral cobaltate anion by ^59^Co NMR spectroscopy in the absence of light. Repeating this reaction with *p*-fluorobromobenzene, working up with d^4^-methanol, and monitoring the organic reaction products by ^1^H, ^2^H, ^13^C, and ^19^F NMR spectroscopy led to the recovery of starting material, with no clear evidence for the formation of the expected debromination product. In line with prior literature, the reaction of K­[Co­(CO)_4_] with aryl bromides appears to be unproductive.^36^ Hence, changes in the ^59^Co NMR are likely a result of reversible formation of an encounter complex, consistent with the UV–vis data.^20^

### Catalysis Kinetics with an Electron-Rich Aryl Bromide

The photoaminocarbonylation reaction of *p*-bromoanisole (0.5 M in *t*-amyl–OH) with piperidine (1.3 equiv) and DBU (1.1 equiv) was selected for kinetic investigation with catalytic [Co_2_(CO)_8_] concentrations between 2.5 and 18.5 mM (0.5–3.7 mol %). A small induction period was observed within the first 0.5 h of reaction, likely due to the equilibration of the operating temperature following a freeze–pump–thaw cycle necessary for CO introduction. Rates were dependent on catalyst concentration between 0.5 and 1.7 mol % loadings (Figure 4a). No evidence of catalyst deactivation and product inhibition was found via same-excess experiments conducted at 1.3 mol % catalyst loading. At catalyst loadings greater than 1.7 mol %, however, no further increase in rate was observed as catalyst loading was increased, suggesting the kinetics had reached saturation. Automatic variable time normalization analysis (Auto-VTNA) was applied to the data set, giving an order in catalyst of 0.9 (*R* ^2^ = 0.975).^42,43^

**Figure 4:**
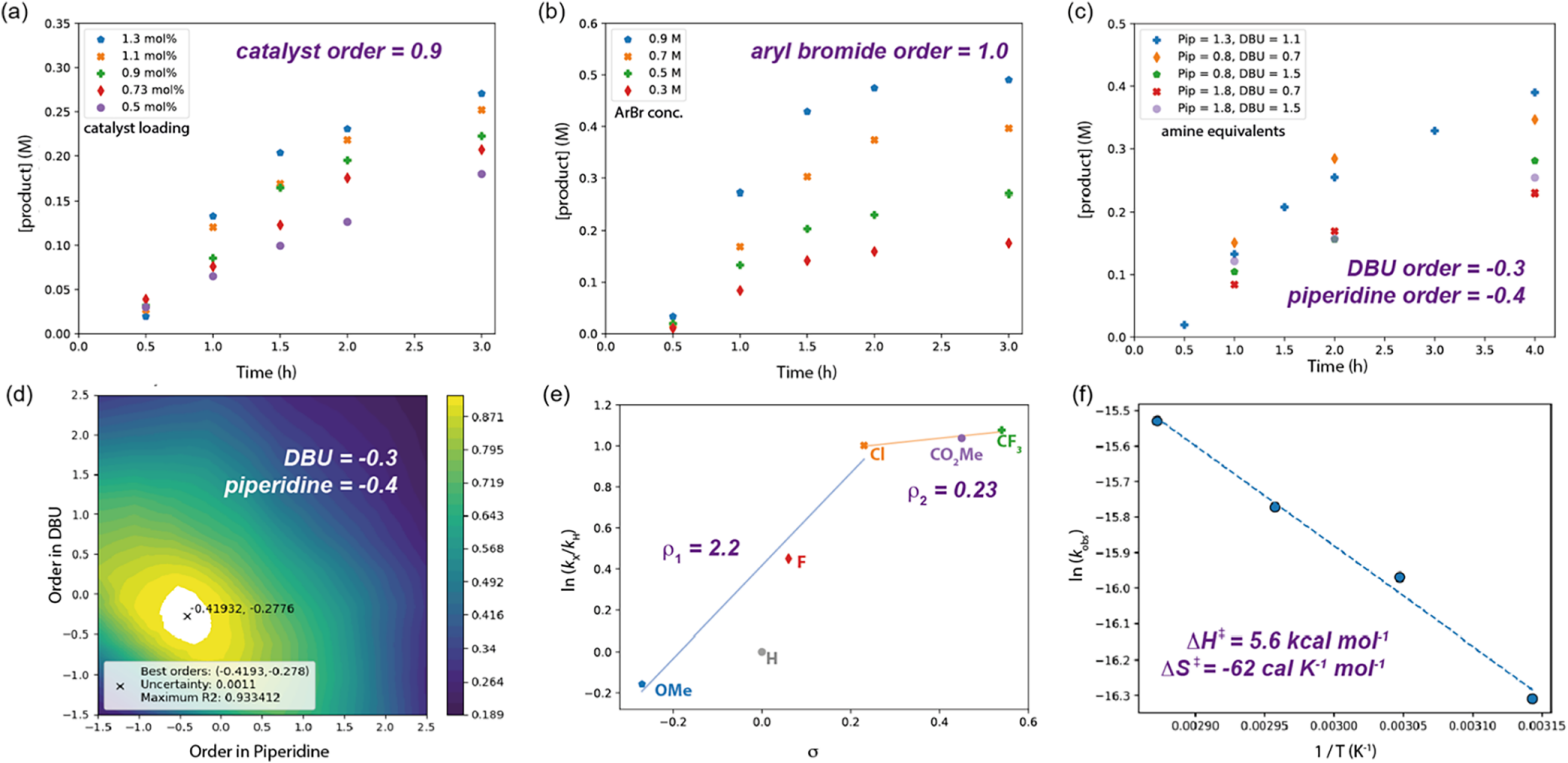
Kinetic profiles for photoaminocarbonylation catalysis with variation of (a) [Co_2_(CO)_8_], (b) *p*-bromoanisole, (c) piperidine, and DBU. (d) Auto-VTNA analysis. (e) Hammett plot for electronically modified aryl bromide substrates at 3 bar CO pressure (R = OMe, H, F, Cl, CO_2_Me, CF_3_). (f) Eyring analysis plot of *p*-bromoanisole substrate. Reactions were tested between 318 and 348 K in increments of 10 K for analysis.

Subsequently, kinetic data were collected for initial *p*-bromoanisole concentrations ranging from 0.3 to 0.9 M with a set catalyst loading of 1.3 mol % (Figure 4b). Expectedly, the rate of reaction increased with the concentration of the substrate. Auto-VTNA analysis confirmed an order of 1.0 (*R* ^2^ = 0.963) in the aryl bromide. The role of DBU and piperidine concentration on the rate of reaction was more complex, giving partial negative order values of −0.3 and −0.4, respectively (Figure 4c,d). Prior literature has suggested that piperidine may play a role as both a nucleophile and an activating agent through reaction with [Co_2_(CO)_8_] during aminocarbonylation reactions.^25^ Order in CO gas was determined through the modification of the CO pressure in the reactor headspace. Changing from 1 to 2 bar pressure had no impact on the rate, suggesting that at these pressures, the reaction may be zero order in CO. At a higher pressure of 3 bar, a dependence on CO was found, however, and a negative order (−1.7) was determined for selected substrates (Figures S11–S14).

### Catalysis Kinetics with an Electron-Poor Aryl Bromide

Further kinetic elucidation was conducted using the electron-deficient *p*-(trifluoromethyl)­bromobenzene substrate for comparison to *p*-bromoanisole. Using the same techniques as those described above, significant differences in kinetics were observed. *p*-(Trifluoromethyl)­bromobenzene reacted faster than *p*-bromoanisole, reducing the window for observation of kinetics from 4 h to approximately 2 h. Modification of the catalyst concentration from 1.3 to 0.9 mol % resulted in no change to the rate of reaction. Only at catalyst loadings below 0.5 mol % was the dependence of catalyst concentration restored. At higher catalyst loading, the system showed no rate dependence on DBU, piperidine, or CO, and a 0.5 order (*R* ^2^ = 0.997) on aryl bromide.

### Hammett and Eyring Analysis

Having observed that electronic modification of the aryl bromide caused changes to the kinetic behavior and order in reagents, a Hammett analysis was conducted (Figure 4e). Qualitatively, the Hammett plot shows an increase in reaction rate for more electron-deficient substrates, displaying a positive ρ_1_ value (ρ_1_ = 2.2, *R*_para_ = OMe to Cl). The plot is, however, nonlinear, showing an inflection point and reduced sensitivity to electronic effects for substrates with the most positive σ values (ρ_2_ = 0.2, *R*_para_ = Cl to CF_3_). This implies that saturated kinetics are reached under these conditions and rates no longer increase linearly. This behavior is reminiscent of that observed at higher initial catalyst loadings when studying the order in [Co_2_(CO)_8_] (*vide supra*). An Eyring analysis was also conducted (Figure 4f). The activation enthalpy and entropy for *p*-bromoanisole substrate were determined as Δ*H* ^‡^ = 5.6 ± 1.6 kcal mol^–1^ and Δ*S* ^‡^ = −62 ± 4.8 cal K^–1^ mol^–1^, respectively. This corresponds to an overall energy barrier of Δ*G*_318K_^‡^ = 25.3 ± 0.11 kcal mol^–1^.

### Radical Probe Experiments

Further measurements and experiments were undertaken in an effort to understand if radical intermediates might be involved in the photoaminocarbonylation mechanism. Actinometry was carried out on the Asynt Illumin8 photoreactor to quantify photon delivery to the reaction.^44^ Using this information, the quantum yield (QY) of the catalytic process was calculated as QY_max_ = 7.7%. *E/Z* mixtures of β-bromostyrene were investigated as a stereochemical probe for radical intermediates. Prior studies have concluded the carbonylation of these substrates is not stereospecific.^20,45^ Catalytic carbonylation under photochemical conditions, using either 92:8 or 100:0 (*E*/*Z*) mixture of β-bromostyrene, led, in both cases, to near 50:50 *E/*Z mixtures of isomeric amide product (Figure 5a). Control reactions in which β-bromostyrene was subjected to the photochemical conditions in the absence of catalyst or CO showed only partial isomerization of *E*-β-bromostyrene to an 80:20 *E*/*Z* mixture after 24 h irradiation with 365 nm LEDs. An intramolecular radical clock was also considered and subjected to the optimized catalytic conditions.^46^ This experiment was complicated by the low conversion of the substrate, possibly due to the alkene interacting with the catalyst,^47^ but showed no clear evidence for the formation of the expected cyclization product that would result from radical ring closure.

**Figure 5:**
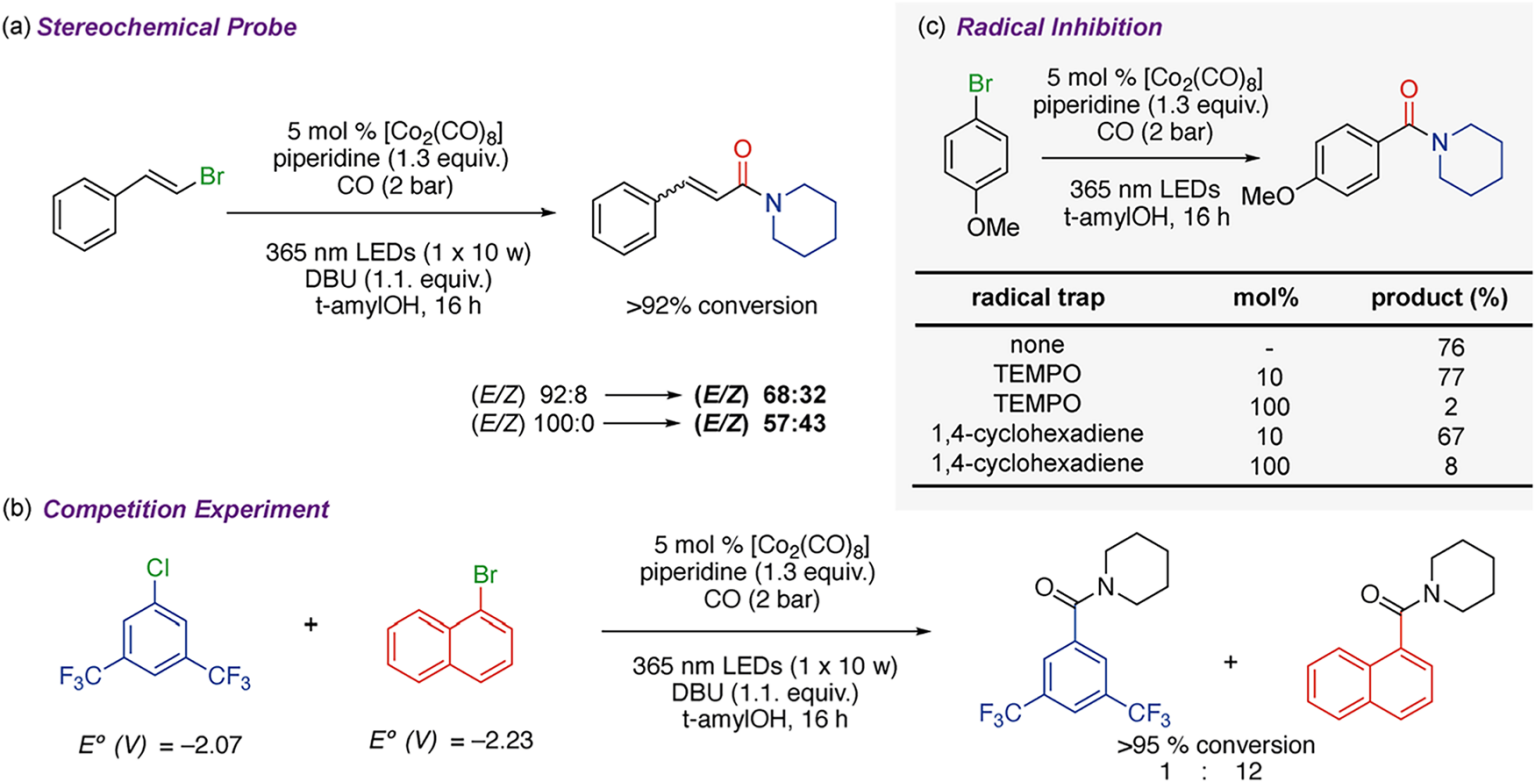
Mechanistic probe experiments for photocatalytic aminocarbonylation. (a) Stereochemical probe E/Z β-bromostyrene. (b) Competition experiment for the SET process. (c) Radical inhibition experiments using TEMPO = (2,2,6,6-tetramethylpiperidin-1-yl)­oxyl and 1,4-cyclohexadiene.

A competition experiment was carried out between an aryl chloride (*E°* = −2.07 V) and an aryl bromide (*E°* = −2.23 V) with known reduction potentials (Figure 5b). These substrates have been investigated previously to differentiate radical and closed-shell mechanisms.^46^ If single electron transfer is occurring, the easier to reduce aryl chloride should react preferentially, if not the aryl bromide with the weaker C–X bond should react first. Under catalytic conditions, a 1:1 mixture of these substrates converted to a 12:1 mixture of products with preferential reaction of the aryl bromide. Attempts to inhibit catalytic aminocarbonylation of *p-*bromofluorobenzene through the addition 10 mol % TEMPO led to a minimal reduction in yields (69% vs 70% without TEMPO). While at much higher loadings of 100 mol % TEMPO, catalysis could be effectively inhibited, and no spectroscopic evidence could be collected to support the formation of aryl TEMPoxide compounds expected from radical trapping (Figure 5c).

## Discussion

### Photon-Limited vs Non-Photon-Limited Catalysis

The kinetic data strongly suggest that the reaction operates under two different kinetic regimes. These regimes are under photon-limited and non-photon-limited conditions.^31,33^ An increase of the reaction rate, whether by increased catalyst loading or substrate activation via electron-withdrawing groups, impacts whether reaction kinetics are photon-limited or not (Figure 6).

**Figure 6:**
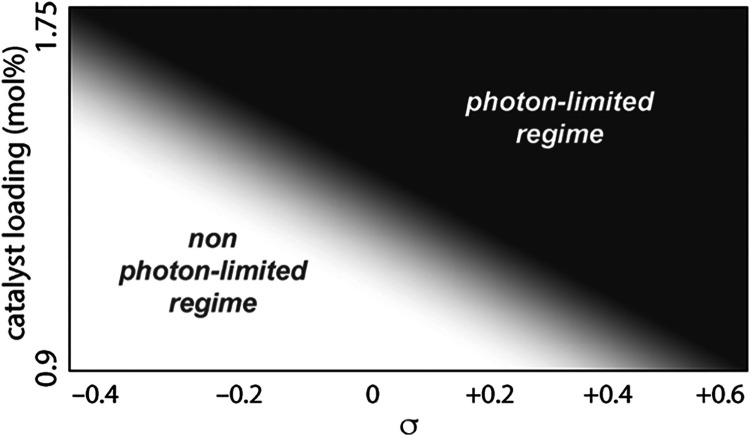
Simplified graphic representation of the transition between photon-limited and non-photon-limited catalysis with changes in catalyst loading and substrate electronics.

A consistent limit for the rate of reaction was found at approximately 0.5 mmol h^–1^, above which photons become the limiting reagent and chemical order is lost. With less activated *p*-bromoanisole, catalyst loadings higher than 1.7 mol % were photon-limited, and kinetics did not show the expected dependence on catalyst or substrate concentration above this. At lower catalyst loadings, the reaction is no longer photon-limited, and the expected near-first-order behavior in the catalyst and aryl bromide is restored. Conversely, the most electron-deficient substrates lose rate dependence at catalyst loadings as low as 0.9 mol %. The following mechanistic discussion is only relevant to the non-photon-limited regime, where photons are not considered to be a limiting reagent.

### Turnover-Limiting Step

The data collected at lower catalyst loadings, and for more electron-rich aryl bromides (*R*_para_ = OMe, H, F) was informative for determining the chemical steps involved in catalytic turnover. The positive order in catalyst and aryl bromide, along with the positive ρ_1_ value from the Hammett analysis, suggests that the reaction of the aryl bromide with [Co­(CO)_4_]^−^ is likely turnover-limiting for this aminocarbonylation reaction. This would also be consistent with the increase in activation enthalpy from the Eyring analysis of aryl bromides compared with aryl chlorides. This finding agrees with prior work of Alexanian and co-workers, substantiating their original hypothesis with robust kinetic data.^20,25,48^

Migratory insertion is not likely to be rate limiting, as this reaction is expected to proceed faster for more nucleophilic aryl groups (i.e., occur with a negative ρ value). Equally, the small negative order in base and nucleophile implies that C–N bond formation via reductive elimination or nucleophilic addition pathways is likely not turnover-limiting, as they would be expected to occur with some positive dependence on the concentration of either base or nucleophile. Rather, the negative order in these reagents suggests these reagents may all play roles in off-cycle equilibria that contribute to the overall rate, rather than being directly involved with the slowest step of the reaction itself.

### Plausible Reaction Mechanism(s) for [Co­(CO)_4_]^−^ and Aryl Bromide

The detailed study of catalyst speciation for this system is complicated by the lack of supporting ligands on cobalt, rendering isolation and characterization of intermediates highly challenging. Furthermore, based on the proposed turnover-limiting step, it might be expected that [Co­(CO)_4_]^−^ is the resting state for the system. The mechanism by which [Co­(CO)_4_]^−^ and aryl bromides react is long debated. Alexanian and co-workers originally suggested that photoaminocarbonylation occurred through the formation of an encounter complex with subsequent single electron transfer from [Co­(CO)_4_]^−^ to the aryl halide (**SET**).^20^ This hypothesis was later revised to a closed-shell mechanism involving photochemical CO dissociation to form a 16-electron complex [Co­(CO)_3_]^−^ that reacts with the aryl halide by a concerted S_N_Ar pathway (**CO dissociation then S**_**N**_ **Ar**).^22,48^ Seminal work by Caubére, Bunnet, and others concluded that this reaction most likely occurs by a radical-nucleophilic substitution, namely, a unimolecular radical chain mechanism (**S**_**RN**_ **1**).^36,37,49,50^ Further alternatives for both radical and closed-shell mechanisms can be considered, including direct reaction of [Co­(CO)_4_]^−^ with the aryl halide by a concerted or stepwise S_N_Ar pathway (**S**_**N**_ **Ar**),^51–54^ oxidative addition of the aryl halide to the 16-electron [Co­(CO)_3_]^−^ intermediate following dissociation of CO from [Co­(CO)_4_]^−^ (**CO dissociation then OA**), and, finally, a radical halogen atom abstraction process (**HAA**).^55,56^ Each of these mechanistic possibilities is discussed in light of the data presented in the results section (Figure 7).

**Figure 7:**
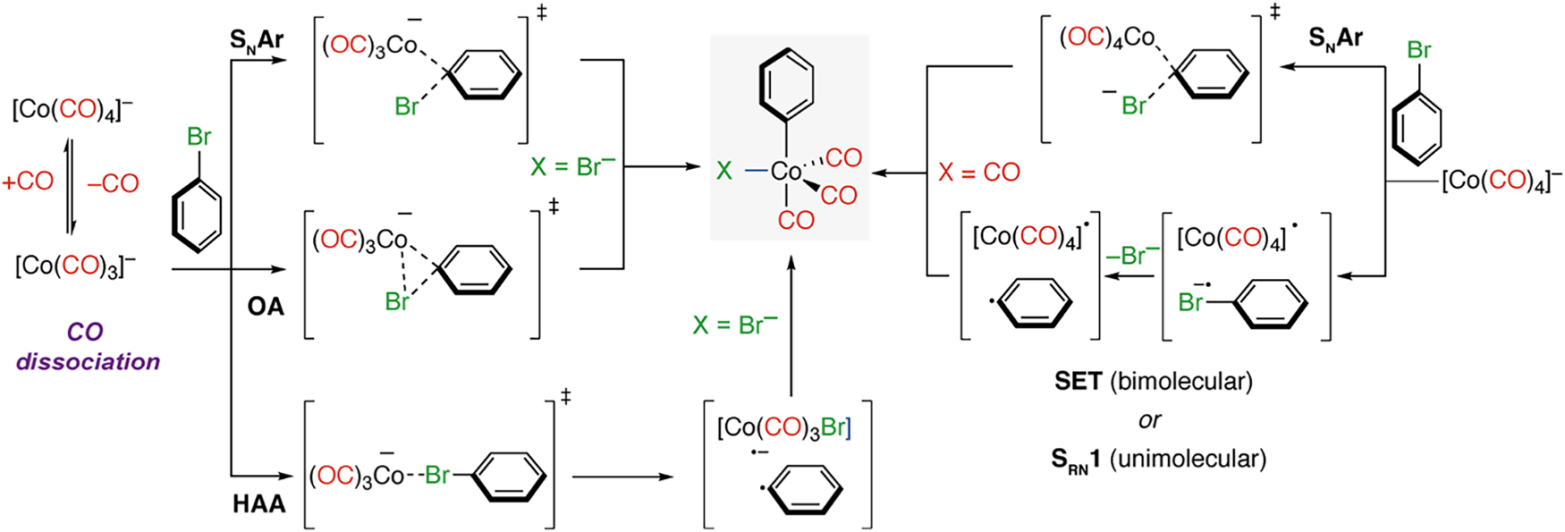
Possible reaction mechanisms for the turnover-limiting addition of [Co­(CO)_4_]^−^ to aryl bromide.

A weight of evidence suggests that the addition of [Co­(CO)_4_]^−^ to aryl bromides is not a radical process. The measured QY of 7.7% is well below that expected for a radical chain process (**S**_**RN**_ **1**), which has been reported with QYs as high as 500%.^57^ The lack of cyclization of a substrate bearing a pendant alkene known to undergo radical cyclization with a rate of ring closure of 8 × 10^9^ s^–1^ is also inconsistent with a radical process, although this experiment is complicated by the apparent effect of the alkene functional group on catalysis (≈10% conv.). Nevertheless, both the single electron transfer mechanism (**SET**) and the halogen atom abstraction (**HAA**) can be effectively discounted by the competition experiment which shows that an aryl bromide with a low reduction potential of *E*° = −2.23 V reacts in preference to an aryl chloride which is expected to be more easily reduced at *E*° = −2.07 V. Hence, electron transfer to the aryl halide is not product determining. The observation that β-bromostyrene, a stereochemical probe, undergoes *E*/*Z* isomerization under the reaction conditions could be considered evidence for a radical intermediate, but it can also be explained through the configurational instability of d^8^ cobalt­(I) vinyl intermediates. Prior work has shown that related d^7^ Co­(II) complexes are configurationally unstable.^58,59^

The remaining closed-shell pathways for the reaction of [Co­(CO)_4_]^−^ with aryl bromides can be considered in light of the Hammett analysis and activation parameters from the Eyring. The ρ_1_ = 2.2 value sits in between the ranges expected for oxidative addition (2–2.5) and concerted S_N_Ar pathways (2.5–4.0) established for palladium­(II) complexes.^51,60^ Nevertheless, this value suggests that a degree of charge separation occurs as the transition state is approached. The small activation enthalpy of Δ*H* ^‡^ = 5.6 ± 1.6 kcal mol^–1^ suggests there is very little bond reorganization and could be interpreted in terms of an early transition state, which in turn might minimize the extent of charge separation in an S_N_Ar pathway. The large and negative activation entropy of Δ*S* ^‡^ = −62 cal ± 4.8 K^–1^ mol^–1^ indicates a bimolecular process, and along with the first-order dependence on aryl bromide effectively rules out a dissociative process being rate determining. Prior work on the addition of aryl iodides to [Pd­(PPh_3_)_4_] concluded that the experimentally determined small and positive Δ*S* ^‡^ arose from a combination of a large positive value for phosphine dissociation and a large negative value for an associative reaction with the substrate.^60^ Similarly, pathways involving a pre-equilibrium step with dissociation of CO from [Co­(CO)_4_]^−^ might be expected to occur with Δ*S* ^‡^ values that are far less negative than that recorded. Currently, we favor the direct, light-promoted reaction of [Co­(CO)_4_]^−^ with the aryl bromide through a concerted S_N_Ar step as the most likely mechanism for the turnover-limiting step of catalysis (**S**_**N**_ **Ar**). The role of light is likely the generation of a photoexcited state [Co­(CO)_4_]^−^*, which is capable of direct reaction with the aryl bromide.^61^ Previous computational investigation of isoelectronic [Ni­(CO)_4_] found a range of accessible excited state structures which can arise following irradiation of these species.^62^ We cannot, however, completely rule out closed-shell pathways involving CO dissociation based on the current data, and indeed the negative order of reaction in CO at pressures above 3 bar would be consistent with the need for CO dissociation as part of the turnover-limiting sequence.

### Updated Catalytic Cycle

Based on prior hypotheses and the new data presented herein, a modified mechanism for aminocarbonylation is proposed (Figure 8). Catalysis is proposed to be initiated by the direct reaction of [Co_2_(CO)_8_] with DBU to form [Co­(CO)_4_]^−^. Subsequent reaction of [Co­(CO)_4_]^−^ (**I**) with aryl bromide via light-promoted S_N_Ar furnishes (**II**) and Br^–^. Migratory insertion results in the formation of the acyl complex [ArCOCo­(CO)_4_] (**III**), which can undergo ligand exchange with piperidine to form (**IV**). Prior work has demonstrated the insertion of CO into the cobalt­(I) aryl motifs to form an isolable low-spin cobalt acyl complex in the absence of light.^63^ In several cases, these compounds are unstable and require phosphine ligands on cobalt to allow isolation.^49,50,64^ Subsequent deprotonation and C–N bond formation are believed to occur simultaneously from this intermediate to yield amide products, based on new computational findings.^48^ There is precedent for amide formation from thermal reaction of cobalt­(I) acyl complexes with amine nucleophiles;^49^ however, these involve LiNMe_2_ as a nucleophile and only the organic, not cobalt, products of the reaction were reported. There is also a single clear example of a structurally characterized Co­(III) complex formed from the addition of aniline to [CpCo­(dppe)­CO]^+^.^65^ Based on the kinetic analysis, the later steps of the proposed cycle (**II–V**) are expected to be fast and the first on-cycle step turnover-limiting. The inverse dependence of rate with DBU and piperidine concentrations also suggests that some off-cycle ligand exchange processes are occurring. This expansive reactivity has been documented previously.^66,67^

**Figure 8:**
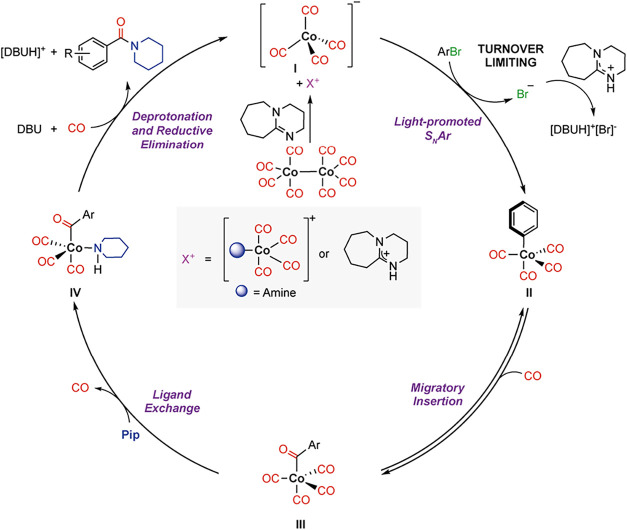
Updated catalytic cycle for the [Co_2_(CO)_8_]-catalyzed aminocarbonylation of aryl bromides.

### Improved Reaction Scope and Efficiency

Based on the mechanistic studies above, the reaction between the aryl halide and in *situ*-generated [Co­(CO)_4_]^−^ is likely turnover-limiting. As such, we envisioned that increasing reaction temperature may improve catalytic performance where overcoming thermal barriers for the turnover-limiting step was important. Eyring analysis comparing a small array of aryl bromide and aryl chloride substrates revealed that the latter have consistently higher activation energies by ΔΔ*G*_318K_^‡^ ≈ 5 kcal mol^–1^. The use of elevated temperatures, higher catalyst loadings, and longer reaction times resulted in a marked improvement for the reaction of challenging aryl chloride substrates (Figure 9). Side-by-side reactions with *p*-fluorochlorobenzene confirmed the importance of conducting these reactions at higher temperatures, with a yield of just 3% obtained with no active heating compared with 65% at 75 °C for 16 h.

**Figure 9:**
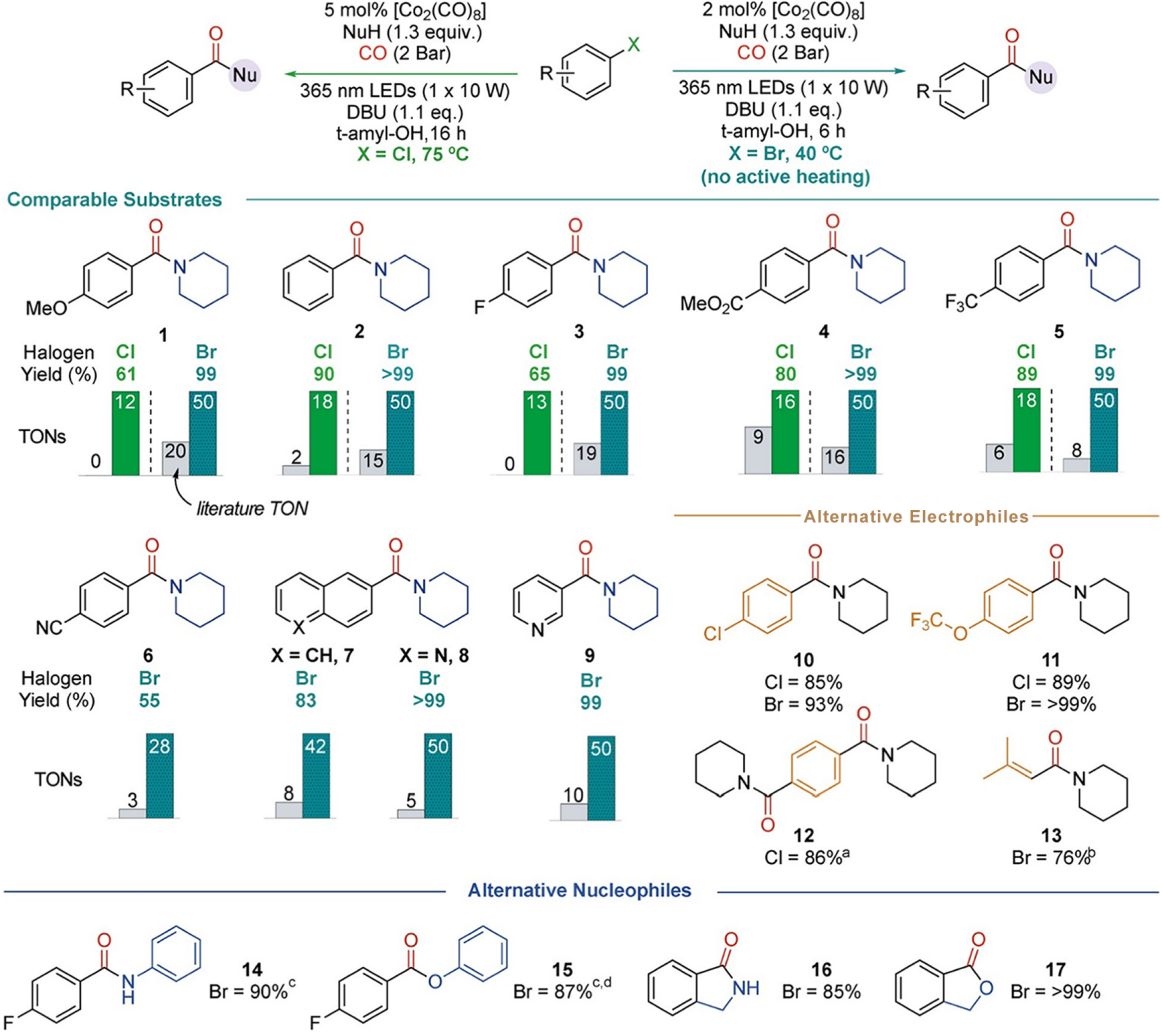
Reaction scope for photocatalyzed aminocarbonylation reaction, yields are isolated unless otherwise stated. TON and TOF comparison for *p*-bromoanisole and piperidine reagents. ^a^0.5 equiv of electrophile used. ^b^Yield determined by quantitative ^1^H NMR spectroscopy versus 1,3,5-trimethoxybenzene internal standard. ^c^Yield determined by quantitative ^19^F NMR spectroscopy relative to α,α,α-trifluorotoluene internal standard. ^d^2 equiv of DBU used.

The reaction scope was revisited using 2 mol % [Co_2_(CO)_8_] and a 6 h reaction time, and aminocarbonylation of a range of aryl halides and nucleophiles was achieved (Figure 9). High isolated yields were obtained for aryl bromides used in the kinetic study (**1–5**, **10**) along with a range of aromatic and heteroaromatic substrates (**6–9**), demonstrating that the protocol operates efficiently for both electron-rich and electron-poor substrates. Isolated yields of >95% were routinely observed for bromide precursors, and remarkably high isolated yields of >80% were achieved for aryl chlorides. Maximum TOFs of 0.7 h^– 1^ were reported for the previous, TMP-activated system with *p*-bromoanisole substrate.^20^ Conversely, TOFs up to 37 h^–1^ were displayed for the same substrates in our system. These could be increased further with *p-*(trifluoromethyl)­bromobenzene up to 45 h^–1^. Maximum turnover numbers (TONs) of >125 have also been demonstrated under our conditions at reduced catalyst loadings, compared with TON_max_ = 20 for the prior literature work.^20^

The enhanced conditions led to an improved scope in both aryl halide and nucleophile. For example, aminocarbonylation of *p*-chloroanisole, a previously inaccessible substrate, was achieved in 61% yield. The reaction of the dihalide substrate *p*-chlorobromobenzene occurred selectively at the bromide site. Exceptional selectivity was achieved for reactions using 1,4-dichlorobenzene via control of electrophile loading, supporting a switchable synthesis of mono or diaminocarbonylation products (**10 and 12**). A new example of acyclic vinyl bromide aminocarbonylation was also shown (**13**). To our knowledge, this is the first example of *N*-(3,3-dimethylacryloyl)­piperidine (**13**) synthesis via a carbonylation reaction. The application of weaker nucleophiles, aniline and phenol, is well tolerated by the conditions (**14 and 15**) with yields of 90% and 87% obtained for the corresponding amide and ester products. These nucleophiles are relevant for pharmaceutical applications as many carbonyl-containing drugs use conjugated amides and ester moieties for enhanced efficacy. Similarly, examples of biologically relevant γ-lactam and γ-lactone syntheses were achieved (**16 and 17**).

## Conclusions

An enhancement of a [Co_2_(CO)_8_]-photocatalyzed aminocarbonylation reaction using DBU as a base has been discovered. ^59^Co NMR and IR spectroscopy provided complementary methods for investigating catalyst speciation, confirming [Co­(CO)_4_]^−^ as the key catalytic species. An in-depth kinetic study of this system strongly suggests that the reaction of the aryl halide with [Co­(CO)_4_]^−^ is the turnover-limiting step for the reaction. The kinetic orders in catalyst and reagents were found to vary between photon-limited and photon-unlimited regimes, highlighting the central role of light in catalytic turnover and rate of reaction.

Radical probe experiments along with a low quantum yield of 7.7% effectively rule out catalysis occurring through single electron transfer processes. Rather, based on the Hammett analysis and activation parameters from Eyring analysis, we suggest that the most likely mechanism of the turnover-limiting step involves a direct, light-promoted reaction of [Co­(CO)_4_]^−^ with the aryl halide by a concerted S_N_Ar step. The new knowledge was applied to an enhanced catalytic protocol, including improved catalyst efficiency through enhancement of TONs and TOFs >10-fold, along with a broader functional group tolerance. A general method for aryl chloride aminocarbonylation is presented by using a combination of light and heat to favor substrate activation. This approach proved to be successful even for challenging electron-rich aryl chlorides. Examples of carbonylative aniline and phenol coupling are also reported, demonstrating a broadened nucleophile scope toward challenging and industrially relevant compounds.

## Supplementary Material



## Notes

Experimental procedures, spectroscopic data for kinetics, mechanistic discovery, and compounds (PDF)
