# Supplementary material for: Mechanistic Investigation of [Co2(CO)8] Catalyzed Photoaminocarbonylation
Source: J Am Chem Soc. 2026 Jun 18;148(25):25905–15. doi: 10.1021/jacs.6c04362 (PMC13339155; doi:10.1021/jacs.6c04362)
Supplement: Supplementary file 1 [file ja6c04362_si_001.pdf]

Supporting Information

Mechanistic Investigation of [$Co_2(CO)_8$] Catalyzed Photoaminocarbonylation

Rowan M. Bailey,[a] Bernd Schaefer,[b] Mark R. Crimmin,[a] Philip W. Miller[a*]*

[a]Department of Chemistry, Imperial College London, W12 0BZ, London, UK
[b]BASF, Ludwigshafen am Rhein, 67056, Rhineland-Palatinate, Germany

*m.crimmin@imperial.ac.uk, philip.miller@imperial.ac.uk

Contents

General Methods and Materials 2
List of Abbreviations 4
Substrate Preparation 5
Reaction Optimization Data 6
Kinetic Data 8
Kinetic Order Determination Tools 25
^{59}Co NMR Data 32
IR Data 51
GC Calibration Curves 72
^{1}H, ^{19}F NMR Spectra - Scope 84
X-Ray Data 101
References 102

General Methods and Materials

Experimental

General experimental consideration

Unless otherwise specified, reactions were carried out using standard Schlenk and glovebox techniques under an N_2 environment. Glassware was dried for a minimum of 12h at 100 °C prior to use. Chemicals were purchased from commercial sources (Merck, Alfa Aesar, VWR, Fischer, Fluorochem) and analysed by NMR spectroscopy and Gas Chromatography (GC) to ensure purity was sufficient. Where purity was not sufficient, sublimation, distillation and recrystallisation techniques were used to purify the compounds. All liquid chemicals used in the aminocarbonylation screening study were degassed via freeze-pump-thaw cycles and dried over 3Å molecular sieves, then taken into a MBRAUN Labmaster glovebox. Solid samples were dried using a high vacuum line prior to cycling into the same glovebox. $Co_2(CO)_8$ was stored in a freezer in the glove box due to its light and temperature sensitivity.

^{1}H, ^{13}C, ^{19}F, ^{31}P and ^{59}Co NMR spectra were obtained using a BRUKER 400 MHz. Chemical shifts (δ) were referenced relative to NMR solvent peaks and data was analysed using a MestReNova software package. Quantitative GC analysis was recorded using a HP-5 column in an Agilent Technologies 7820A GC instrument with FID detector. Helium was used as a carrier gas and n-dodecane used as an internal standard. Column chromatography was performed using an appropriate solvent system and 230-400 mesh silica. Thin later chromatography (TLC) analysis was performed using precoated aluminium/glass backed plates and visualised using UV light (254 nm) irradiation or potassium permanganate staining. IR measurements were taken on an Agilent Cary630 spectrometer placed within an MBraun glovebox. UV-vis analysis was carried out using an Agilent Cary 60 spectrophotometer with a J-Young capped airtight cuvette.

Asynt Illumin8 Reactor Set up

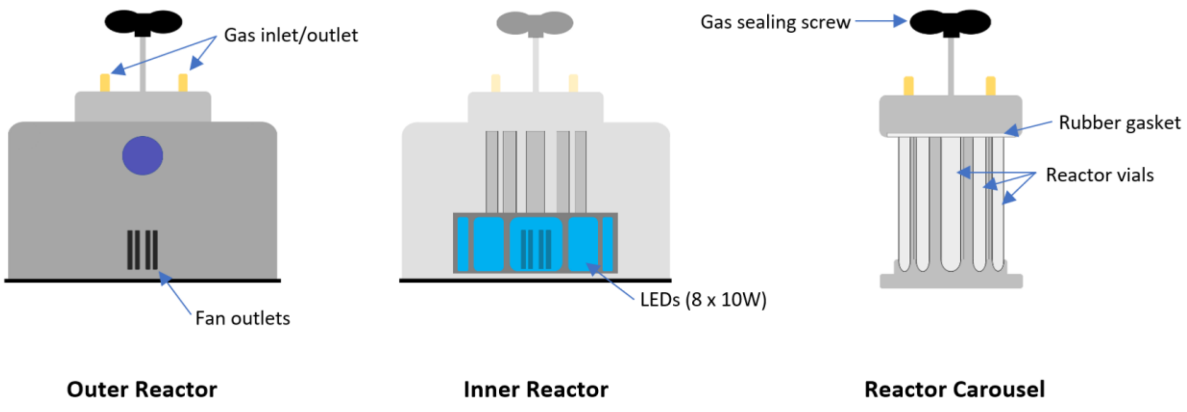

Figure S1: Asynt DrySyn Illumin8 Reactor Schematic: 8 x 10 W (power) 365 nm LEDs. Reactor carousel can be removed and loaded accordingly. (See images below)

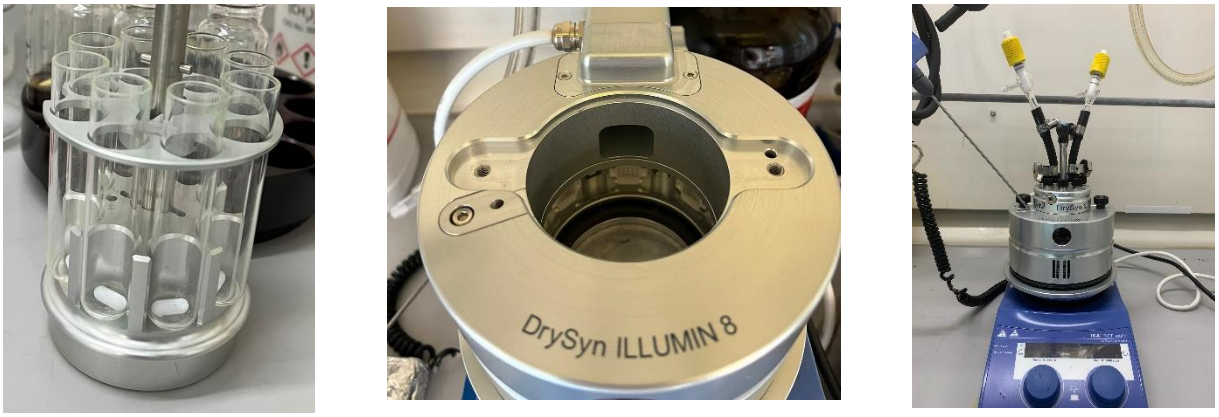

Figure S2: Reactor Carousel with Stirrer Bars (Left). Internal Bank of LEDs (Middle). External view of active reactor with modified taps for gas tight synthesis (Right).

GC Quantification

Table S1: GC Method Details

Temperature (°C)	***Hold (min)***	***Ramp (°C/min)***
50	1	2
80	0	25
105	4	25
300	4	0

Total run time = 28.8 min, Injection volume = 1 uL, Column = GC HP-5 Column.

Example Trace: 20.492 = 4-bromoanisole, 20.860 = IS (n-dodecane), 27.684 = Product Amide

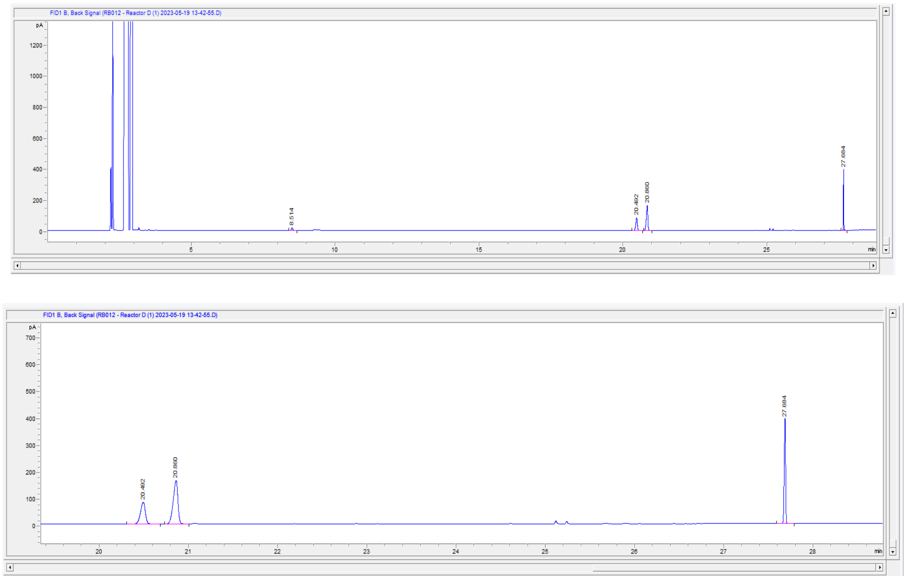

Figure S3: Example GC trace (Top). Zoomed GC trace, starting material, n-dodecane, product amide from left to right (Bottom).

List of Abbreviations

ArBr – Aryl Bromide
Bu - Butyl
Cy - Cyclohexyl
DBU - 1,8-Diazabicyclo(5.4.0)undec-7-ene
Et - Ethyl
EtOAc – Ethyl Acetate
Eq. – Equivalents
GC - Gas Chromatography
LED – Light Emitting Diode
Me – Methyl
OAc - Acetate
Ph - Phenyl
T-amyl-OH - 2-methylbutan-2-ol
tBu – tert-butyl
TEA - Triethylamine
TMP - Tetramethylpiperidine
VTNA – Variable time normalisation analysis
Xantphos - (9,9-Dimethyl-9H-xanthene-4,5-diyl)bis(diphenylphosphane)

Substrate Preparation

General Aminocarbonylation Procedure A (Optimization)

In a glovebox under N_2, $Co_2(CO)_8$ (0.5 mmol, 0.187g) was dissolved in 10 mL t-amylOH in a capped vial and stirred until no solid catalyst was visible to create a catalyst stock solution. Into a separate vial was added 1,8-Diazabicyclo[5.4.0]undec-7-ene (DBU) (1.478 ml, 9.9 mmol), piperidine (1.158 ml, 11.7 mmol) and t-amylOH (6.4 ml) to afford 9mL of an amine stock solution. Directly into each reactor vial was charged aryl halide (1 mmol), n-dodecane (0.11 mmol) and a stirrer bar, followed by the amine stock solution (1mL). Each reactor vial was subsequently stirred, followed by catalyst stock solution addition (1 mL) then stirred again. Once all reactor vials were charged, the illumin8 carousel was assembled and sealed in the glovebox using J-Young taps. The carousel was removed from the glove box and reaction mixtures were frozen using a dry ice/acetone bath. The mixtures were then subjected to three vacuum-CO (1 bar) cycles and subsequently charged with the appropriate CO pressure. The illumin8 reactor was then assembled around the CO charged vials, then stirring and LEDs were switched on. Once a sufficient reaction time had been reached, the reaction mixtures were diluted with EtOAc (3 mL) and filtered through a PTFE filter into a GC vial (0.5 mL). The sample was then diluted in a further 0.5 mL EtOAc and taken for GC analysis.

General Aminocarbonylation Procedure B (Aryl Bromide Scope)

In a glovebox under N_2, $Co_2(CO)_8$ (0.2 mmol, 0.068g) was weighed into an ampoule, sealed under N_2 and removed from the glovebox. *t*-AmylOH (5 mL) was added to the ampoule using standard Schlenk line techniques to create a catalyst stock solution. In a separate, nitrogen flushed flask was added DBU (1.642 ml, 11 mmol), piperidine (1.287 ml, 13 mmol) and t-amylOH (12.071 ml) to afford a 15 mL amine stock solution. To each illumin8 vial was added aryl bromide (1 mmol) under a flow of N_2. Subsequently, the amine stock solution (1.5 mL) was added into each reactor vial, stirred and then follow by addition of the catalyst stock solution (0.5 mL). Reaction mixtures were then frozen using a dry ice/acetone bath. The mixtures were then subjected to three vacuum-CO (1 bar) cycles and subsequently charged with the appropriate CO pressure. The illumin8 reactor was then assembled around the CO charged vials, then stirring and LEDs were switched on. After 6 hours of illumination the headspace was opened to air and reaction mixtures were diluted in EtOAc (25 mL) and HCl (1M, 10 mL). The reactions were then extracted in EtOAc (3 x 25 mL). The resulting crude organic phase was then dried and purified using Biotage flash chromatography (EtOAc:Hexane 0-100%).

General Aminocarbonylation Procedure C (Aryl Chloride Scope)

In a glovebox under N_2, $Co_2(CO)_8$ (0.5 mmol, 0.171g) was weighed into an ampoule, sealed under N_2 and removed from the glovebox. *t*-AmylOH (10 mL) was added to the ampoule using standard Schlenk line techniques to form a catalyst stock solution. In a separate, nitrogen flushed flask was added DBU (1.642 ml, 11 mmol), piperidine (1.287 ml, 13 mmol) and t-amyl-OH (7.071 ml) to afford a 10 mL amine stock solution. To each illumin8 vial was added aryl chloride (1 mmol) under a flow of N_2. Subsequently, amine stock solution (1 mL) was added into each reactor vial, stirred and then followed by addition of catalyst stock solution (1 mL). Reaction mixtures were then frozen using a dry ice/acetone bath. The mixtures were then subjected to three vacuum-CO (1 bar) cycles and subsequently charged with the appropriate CO pressure. The illumin8 reactor was then assembled around the CO charged vials, then stirring, LEDs and hotplate heating (75 °C) were switched on. After 16 hours of illumination the headspace was opened to air and reaction mixtures were diluted in EtOAc (25 mL) and HCl (1M, 10 mL). The reactions were then extracted in EtOAc (3 x 25 mL). The resulting crude organic phase was then dried and purified using Biotage flash chromatography (EtOAc:Hexane 0-100%).

General Stoichiometric Amide Synthesis Procedure

To a two-neck round bottom flask was added substituted benzoic acid (20 mmol) and a stirrer bar. A condenser was fitted to the flask in preparation for reflux condition. To the flask was then added thionyl chloride (5.8 ml, 80 mmol, 4.0 equiv.). The mixture was stirred and heated to reflux (150 °C). After 4 hours, the reaction was cooled to room temperature and evacuated to remove excess $SOCl_2$. Following this, the neat acyl chloride product was diluted in 40ml DCM. To a 2-dram vial was added piperidine (2.2 ml, 22 mmol, 1.1 equiv.) and triethylamine (3.5 ml, 25 mmol, 1.25 equiv.). A flow of nitrogen was set up over the top of the reaction mixture then the amine mixture was added *via* syringe in 0.1ml portions across 0.5 hours to the acyl chloride solution. The reaction was left to stir overnight, then worked up with 1M HCl (3 x 15 mL) and brine (3 x 15 mL). Subsequent drying of the organic layer yielded clean amide products with no further purification necessary. Procedure was developed from combined prior literature. [1, 2]

Optimised Workflow for Kinetic Data collection:

The model photoaminocarbonylation reaction of 4-bromoanisole (0.5M in t-amylOH) with piperidine was selected for the kinetic investigation with a 1.3 mol% loading of $Co_2(CO)_8$ in a modified commercially available 8-well parallel photoreactor (Asynt Illumin8). Kinetic data was collected at the stated timepoints with each visualised data point representing an average of at least two unique reactions, quantified by gas chromatography (GC). The 8-well parallel photoreactor enabled four unique reaction conditions to be tested at a single time point per day. Kinetic curves were plotted using 6 timepoints and typically required 6 lab days to collect, inclusive of work-up and quantitative analysis *via* gas chromatography (GC). High stirrer rates (1000 rpm) were used for the reactions to reduce mass-transfer limitations of CO into solution.

Reaction Optimization Data

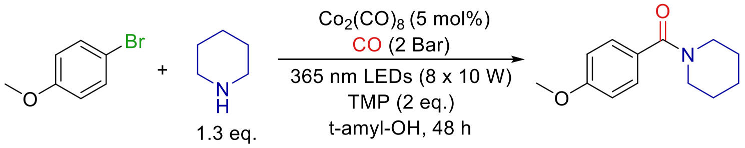

Entry	*Concentration*	*CO Pressure (Bar)*	*TMP (eq.)*	*Yield (%)*
1	0.05	1	2	35
2	0.2	1	2	52
3	0.4	1	2	89
4	0.5	1	2	97
5	0.7	1	2	96
6	1.0	1	2	91
7	0.5	2	0	70
8	0.5	2	1	93
9	0.5	2	2	97

Table S2: Concentration, CO pressure and TMP loading optimization data

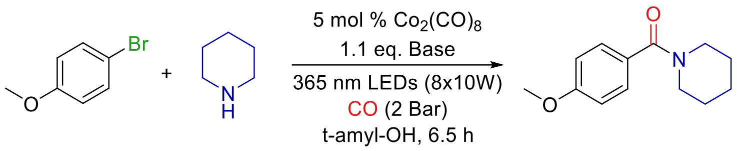

Entry	Base	Condition	Yield (%)
1	Triethylamine	Benchtop	20
2	Di-*iso*-propylamine	Benchtop (previously distilled)	68
3	TMP	Glovebox	28
4	2,6-Di-*tert*-butylpyridine	Benchtop	11
5	DBU	Glovebox	69
6	Pyridine	Glovebox	3
7	KO^{t}Bu	Glovebox	11(38 ester)

Table S3: Base testing for photoaminocarbonylation reaction.

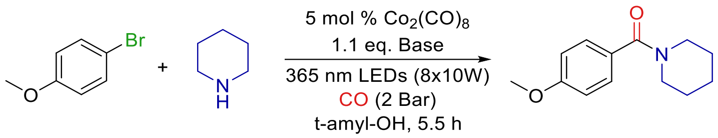

Entry	Base	Loading (eq.)	Condition	Yield (%)
1	Triethylamine	1.1	Cleaned	14
2	Triethylamine	2	Cleaned	7
3	Di-*iso*-propylamine	1.1	Glovebox	53
4	TMP	0.5	Distilled	7
5	TMP	1.1	Distilled	24
6	TMP	2	Distilled	14
7	DBU	1.1	Glovebox	61
8	None			14

Table S4: Further optimization of base choice and loading.

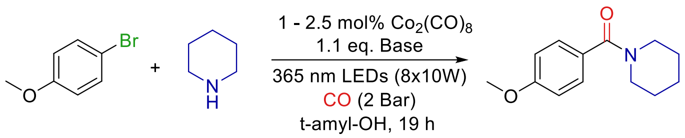

Entry	Base	Catalyst Loading (mol%)	Yield (%)
1	DBU	1	94
2	DBU	2.5	93
3	TMP	1	24
4	TMP	2.5	51
5	Triethylamine	1	26
6	Triethylamine	2.5	63
7	None	1	24
8	None	2.5	60

Table S5: Lower loading [$Co_2(CO)_8$] reactions across multiple bases.

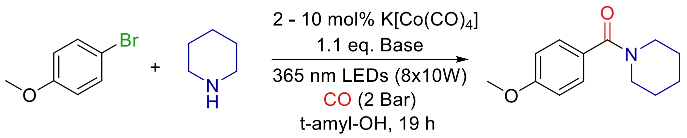

Entry	Base	Base Loading (eq.)	Catalyst Loading (mol%)	Yield (%)
1	TMP	1.1	10	96
**2**	**TMP**	**1.1**	**2**	**25**
3	DBU	0.8*	10	89
4	DBU	0.8*	2	68

*Table S6: K[Co(CO)₄] aminocarbonylation reaction comparison. *Lack of DBU availability prompted use of sub-stoichiometric loading.*

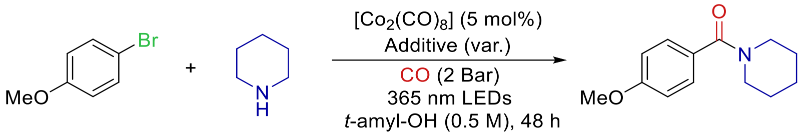

Entry	Radical Trap	Loading (mol%)	Amide Yield (%)
1			76
2	TEMPO	10	66
3	TEMPO	100	<2
4	1,4-Cyclohexadiene	10	67
5	1,4-Cyclohexadiene	50	15
6	1,4-Cyclohexadiene	100	8

Table S7: Radical trapping experiments using TEMPO and 1,4-Cyclohexadiene at variable loading

Kinetic Data

General Kinetic Catalyst Order Determination Procedure

Stock 1 (organic) procedure: A vial was charged with 4-bromoanisole (17 mL, 135 mmol), n-dodecane (2.0 mL, 8.8 mmol) and t-amyl-OH (80 mL) to allow for consistent quantitation of reaction mixtures by GC relative to internal standard (IS) n-dodecane. Concentration was selected to allow a consistent loading of 1 mmol aryl bromide per reaction vial following dilution with DBU and piperidine as described below.

In a glovebox under N_2, $Co_2(CO)_8$ (0.1 mmol, 0.034g) was dissolved in t-amylOH (8 mL) and stirred for 15 minutes to create a catalyst stock solution. In a separate vial was measured **Stock 1** (8 mL), DBU (1.8 ml, 12 mmol), piperidine (1.4 mL, 14 mmol) and stirred for 5 minutes to produce an organic stock. The organic stock (1 mL) was subsequently added into each reactor vial, followed by the appropriate amount of catalyst stock to achieve the desired catalyst loading. *t*-AmylOH was added into each reactor vial as necessary to maintain 2 mL total reaction volumes. Each reaction vial was made in duplicate. Hence, 4 conditions were tested per illumin8 run. Once all reactor vials were charged, the illumin8 vial carousel was assembled and sealed in the glovebox using J-Young taps and mixtures subsequently stirred for a further 5 minutes. The carousel was then removed from the glovebox and reaction mixtures frozen using a dry ice/acetone bath. The mixtures were subjected to three vacuum-CO (1 atm) cycles and subsequently charged with the appropriate CO pressure.

The illumin8 reactor was then assembled around the CO charged vials and the stirring and LEDs were switched on. After the reactions had been illuminated for the desired reaction time, the reactor was flushed with N_2 to displace CO, then opened to air to decompose the cobalt complex. The reaction mixtures were diluted with EtOAc and filtered through a PTFE plug directly into GC vials for analysis.

General Kinetic Substrate Order Determination Procedure

Concentration Modified *p*-Bromoanisole Stock: To a vial in a glovebox was added n-dodecane (2 mL), ArBr (relative) and topped up to 26 mL with *t*-amylOH. *(Example below for p-bromoanisole loadings)*

Concentration (M)	*p-Bromoanisole (mL)*
0.3	2.77
0.5	4.62
0.7	6.46
0.9	8.31

In a glovebox under N_2, $Co_2(CO)_8$ (0.117 mmol, 0.036g) was dissolved in t-amyl-OH (8 mL) and stirred for 15 minutes to create a catalyst stock solution. To a separate vial was added piperidine (1.16 mL, 11.7 mmol) and DBU (1.48 mL, 9.9 mmol) to create an amine stock solution. To each illumin8 reaction vial was added ***p*-Bromoanisole stock** (0.7 mL), amine stock solution (0.29 mL) and stirred. Subsequently, the catalyst stock solution (1 mL) was added to each vial and stirred again. This series of additions yields 0.3M – 0.9M substrate concentration reaction mixtures while maintaining constant reaction volumes. Once all reactor vials were charged, the illumin8 vial carousel was assembled and sealed in the glovebox using J-Young taps and reaction mixtures subsequently stirred for a further 5 minutes. The carousel was then removed from the glove box and reaction mixtures frozen using a dry ice/acetone bath. The mixtures were subjected to three vacuum-CO (1 atm) cycles and subsequently charged with the appropriate CO pressure. The illumin8 reactor was then assembled around the CO charged vials and the stirring and LEDs were switched on. After the reactions has been illuminated for the desired reaction time, the reactor was flushed with N_2 to displace CO, then opened to air to decompose the cobalt complex. The reaction mixtures were diluted with EtOAc and filtered through a PTFE plug directly into GC vials for analysis.

General DBU and Piperidine Order Determination Procedure

In a glovebox under N_2, $Co_2(CO)_8$ (0.117 mmol, 0.036g) was dissolved in *t*-amylOH (8 mL) and stirred for 15 minutes to create a catalyst stock solution. In a separate vial was added ArBr (9 mmol), n-dodecane (0.2 mL, 0.88 mmol), DBU (0.94 mL, 6.3 mmol, 0.7 eq), piperidine (0.712 mL, 7.2 mmol, 0.8 eq) and *t*-amylOH (5.89 mL) to create an organic stock solution. The organic stock solution was then dispensed into each reaction vial (1 mL). DBU (0.119 mL, 0.8 mmol, 0.8 eq.) and piperidine (0.099 mL, 1 mmol, 1 eq.) were then added into reaction vials as necessary for higher loading testing. The mixtures were stirred then catalyst stock solution added (1 mL). The illumin8 vial carousel was then assembled and sealed in the glovebox using J-Young taps and reaction mixtures subsequently stirred for a further 5 minutes. The carousel was then removed from the glove box and reaction mixtures frozen using a dry ice/acetone bath. The mixtures were subjected to three vacuum-CO (1 atm) cycles and subsequently charged with the appropriate CO pressure. The illumin8 reactor was then assembled around the CO charged vials and the stirring and LEDs were switched on. After the reactions has been illuminated for the desired reaction time, the reactor was flushed with N_2 to displace CO, then opened to air to decompose the cobalt complex. The reaction mixtures were diluted with EtOAc and filtered through a PTFE plug directly into GC vials for analysis.

General Hammett Kinetics Determination Procedure

Aryl Bromide Stock Solution Preparation: To a vial in a glovebox was added ArBr (60 mmol), n-dodecane (1 mL, 4.4 mmol), topped up to 30 mL total with *t*-amylOH. (2 M stock solution, diluted to 0.5 M through below procedure for reaction)

In a glovebox under N_2, $Co_2(CO)_8$ (0.117 mmol, 0.036g) was dissolved in t-amyl-OH (8 mL) and stirred for 15 minutes to create a catalyst stock solution. To a separate vial was measured DBU (1.478 mL, 9.9 mmol), piperidine (1.16 mL, 11.7 mmol) and t-amyl-OH (1.854 mL) to produce an amine stock solution. To each reaction vial was dispensed **Aryl Bromide Stock solution** (0.5 mL) followed by amine stock solution (0.5 mL). The reaction mixtures were subsequently stirred and then catalyst stock solution added (1 mL). Once all reactor vials were charged, the illumin8 vial carousel was assembled and sealed in the glovebox using J-Young taps and reaction mixtures stirred for a further 5 minutes. The carousel was then removed from the glove box and reaction mixtures frozen using a dry ice/acetone bath. The mixtures were subjected to three vacuum-CO (1 atm) cycles and subsequently charged with the appropriate CO pressure. The illumin8 reactor was then assembled around the CO charged vials and the stirring and LEDs were switched on. After the reactions has been illuminated for the desired reaction time, the reactor was flushed with N_2 to displace CO, then opened to air to decompose the cobalt complex. The reaction mixtures were diluted with EtOAc and filtered through a PTFE plug directly into GC vials for analysis.

General CO Order Determination Procedure

In alignment with the **General Hammett Kinetics Determination Procedure**, vials were prepared using stock solutions where necessary. Following vacuum-CO purging, the reactions were set to 1,2 and 3 bar CO respectively, depending on the desired kinetic experiment. Work up conditions and analysis were constant throughout data collection.

Raw Kinetic Data

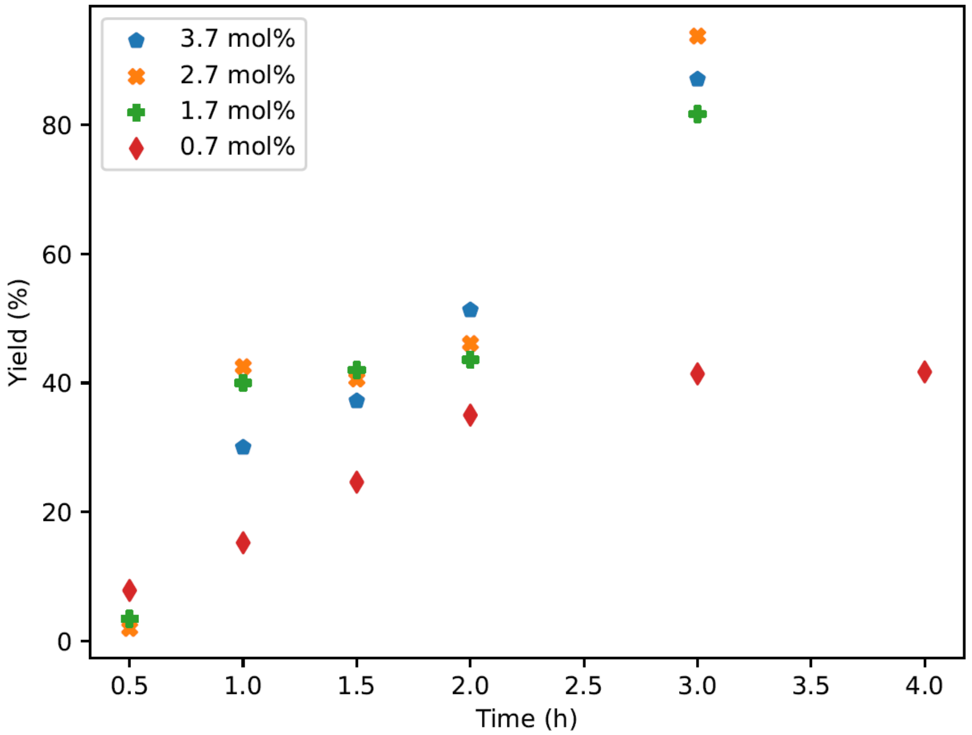

Figure S4: Kinetics for varied catalyst loading. R_{para} = OMe

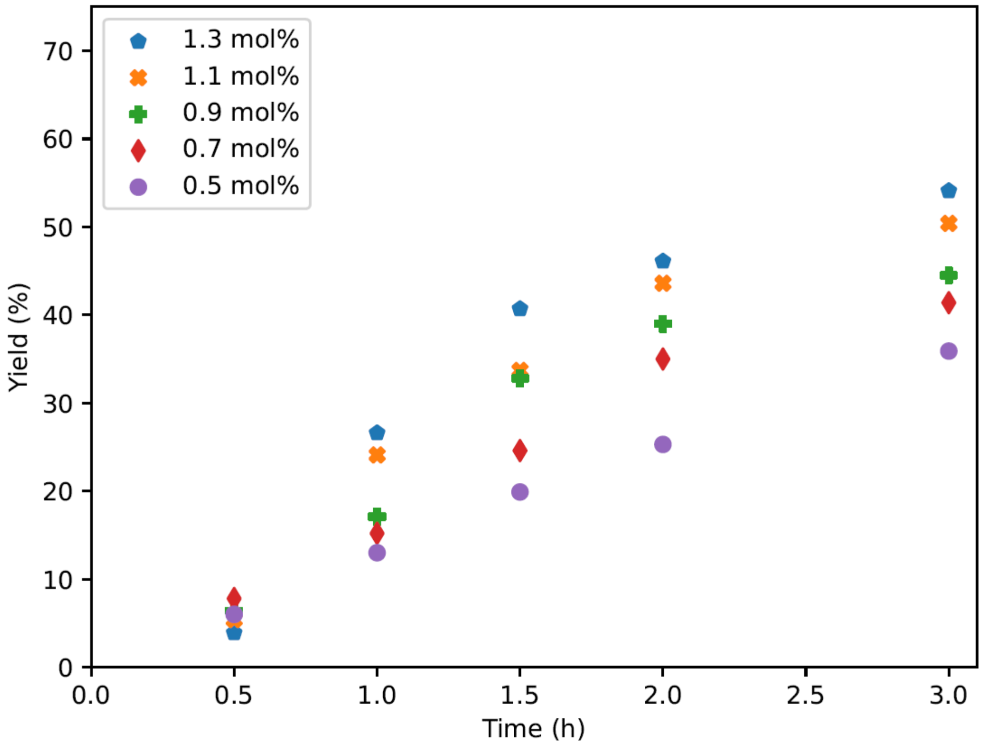

Figure S5: Kinetics for varied catalyst loading. R_{para} = OMe

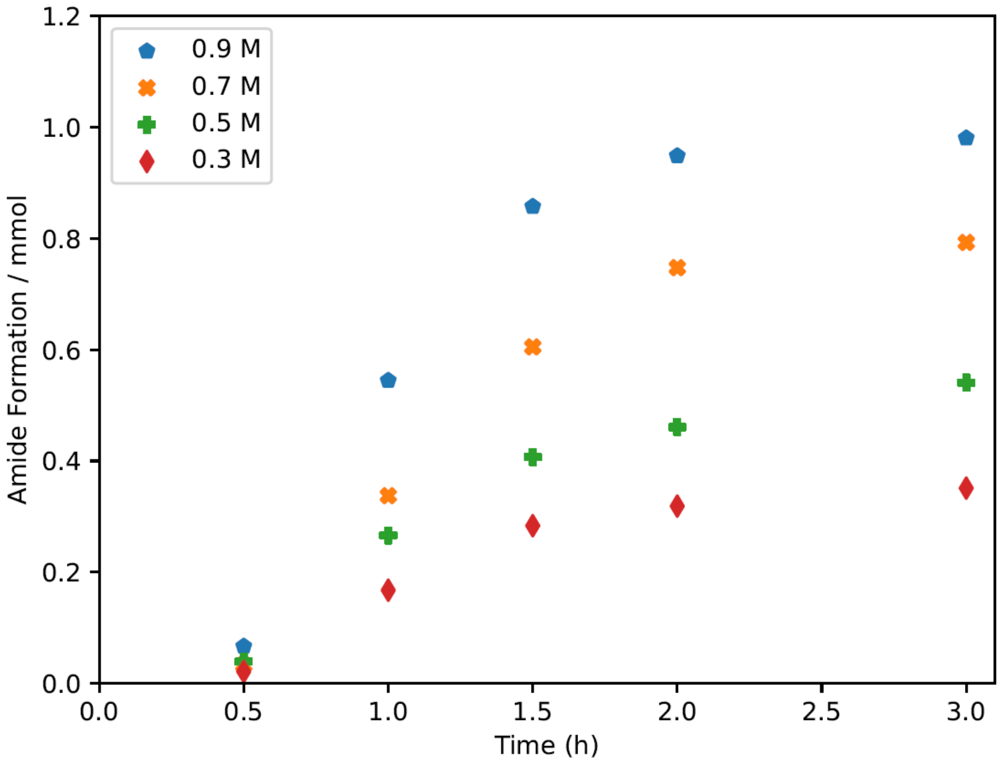

Figure S6: Kinetics for varied substrate loading. R_{para} = OMe

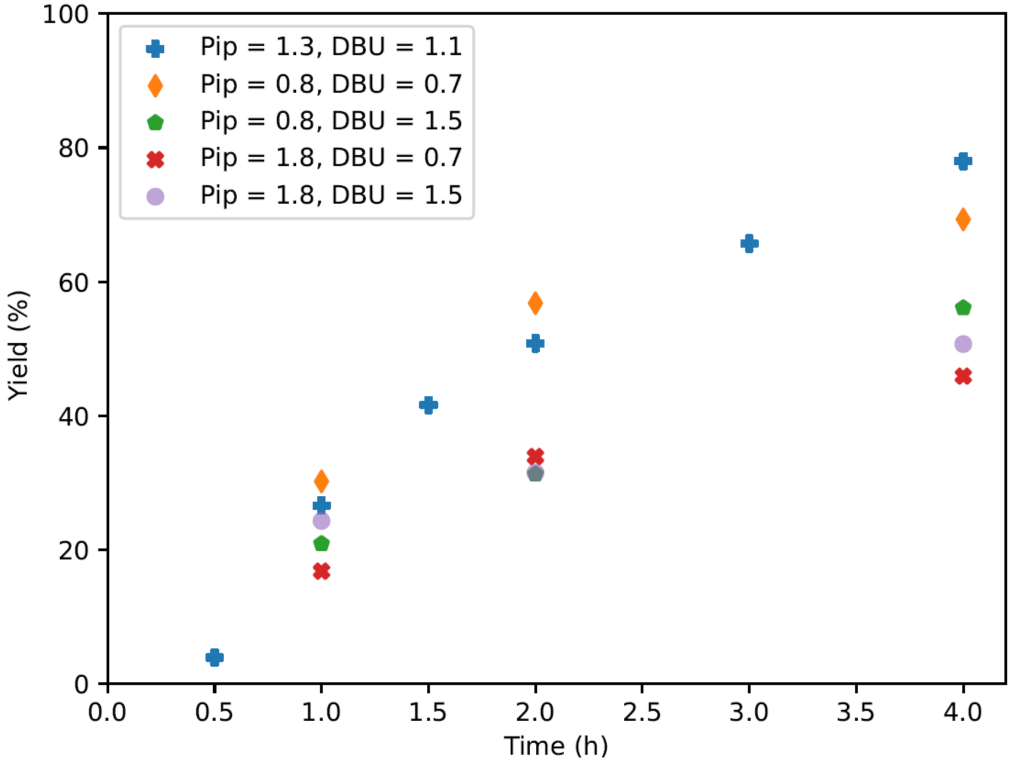

Figure S7: Kinetics for varied base and nucleophile loading. R_{para} = OMe

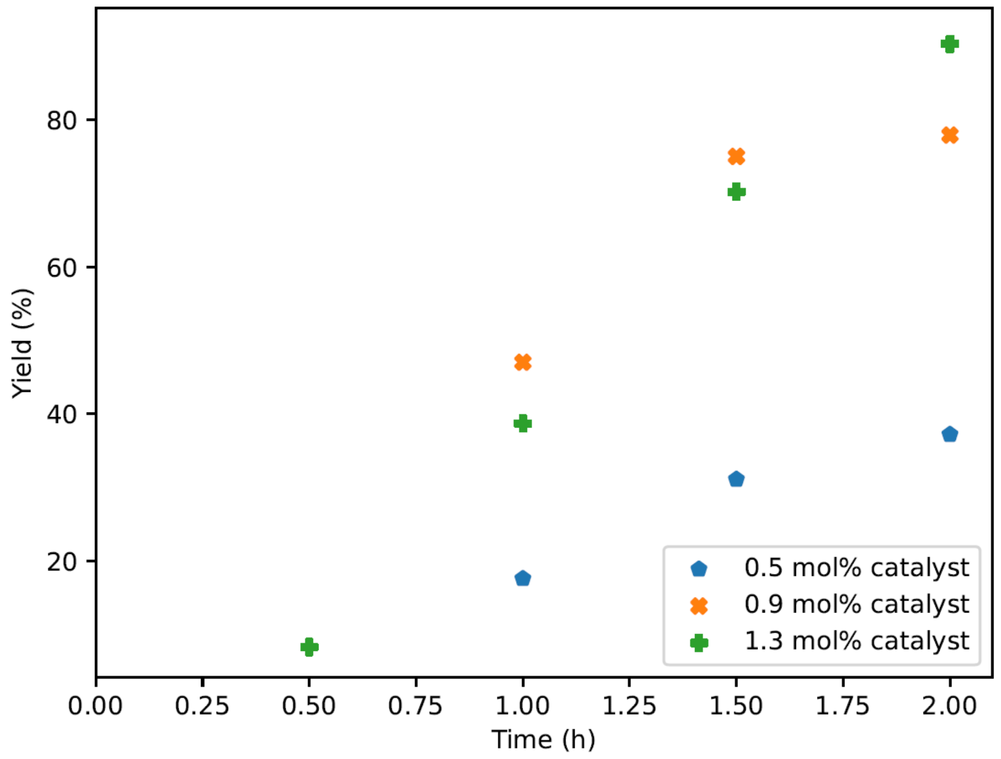

Figure S8: Kinetics for varied catalyst loading. $R_{para} = CF_3$

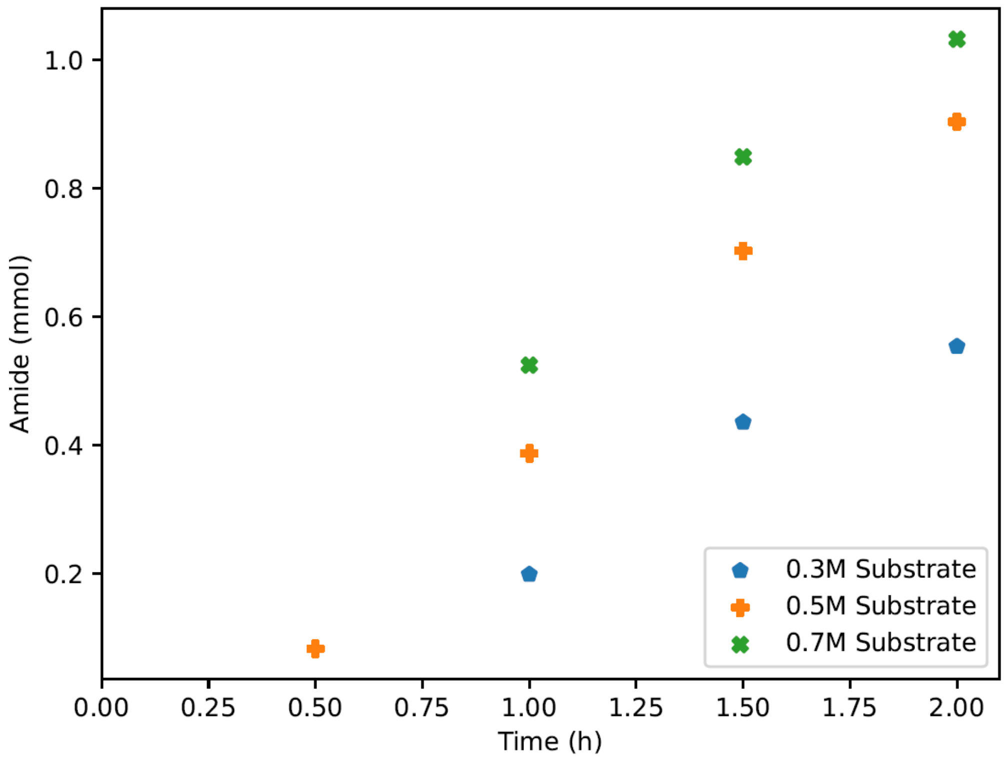

Figure S9: Kinetics for varied substrate loading. $R_{para} = CF_3$

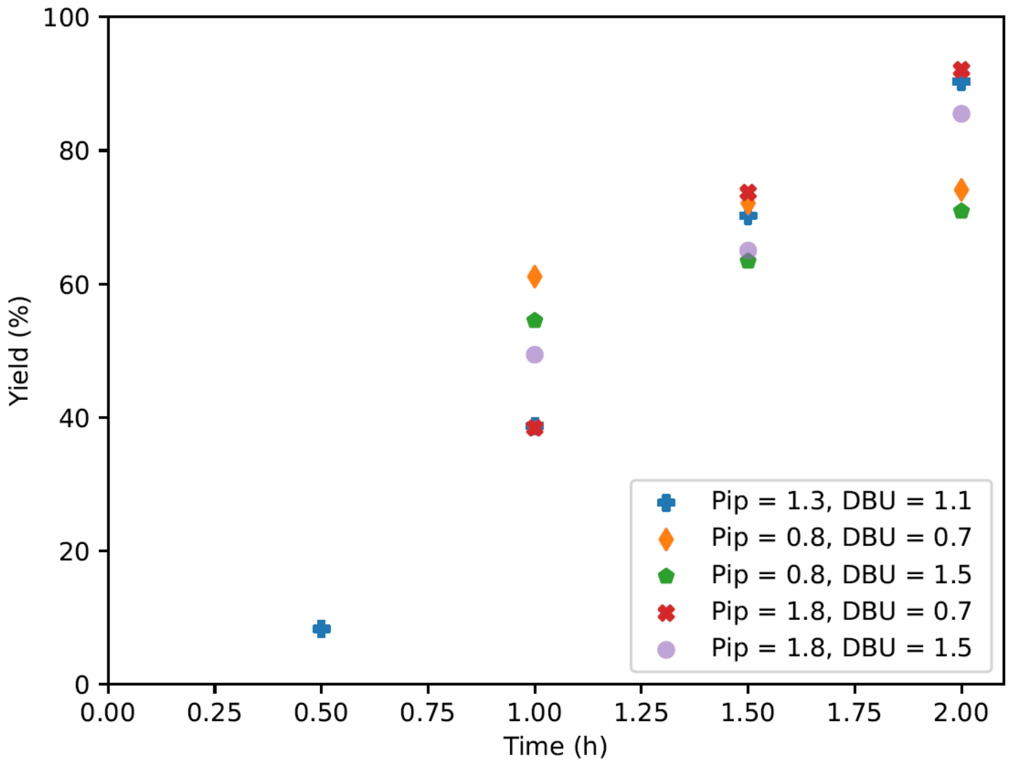

Figure S10: Kinetics for varied base and nucleophile loading. R_{para} = CF_3

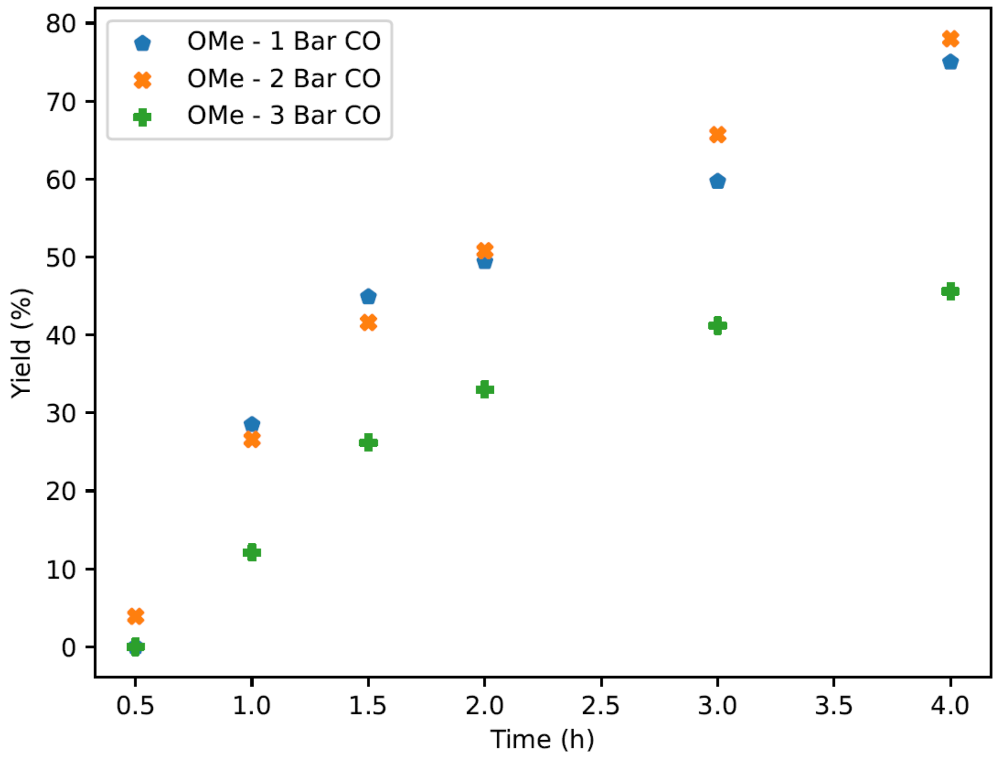

Figure S11: Kinetics for varied CO pressure. R_{para} = OMe

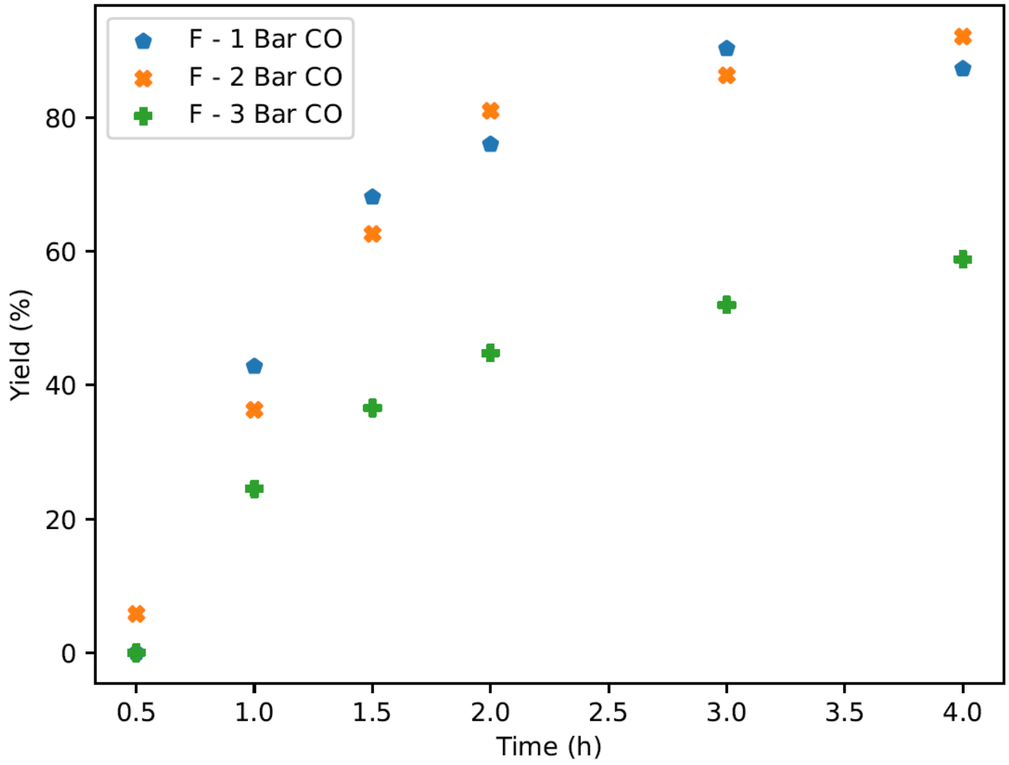

Figure S12: Kinetics for varied CO pressure. R_{para} = F

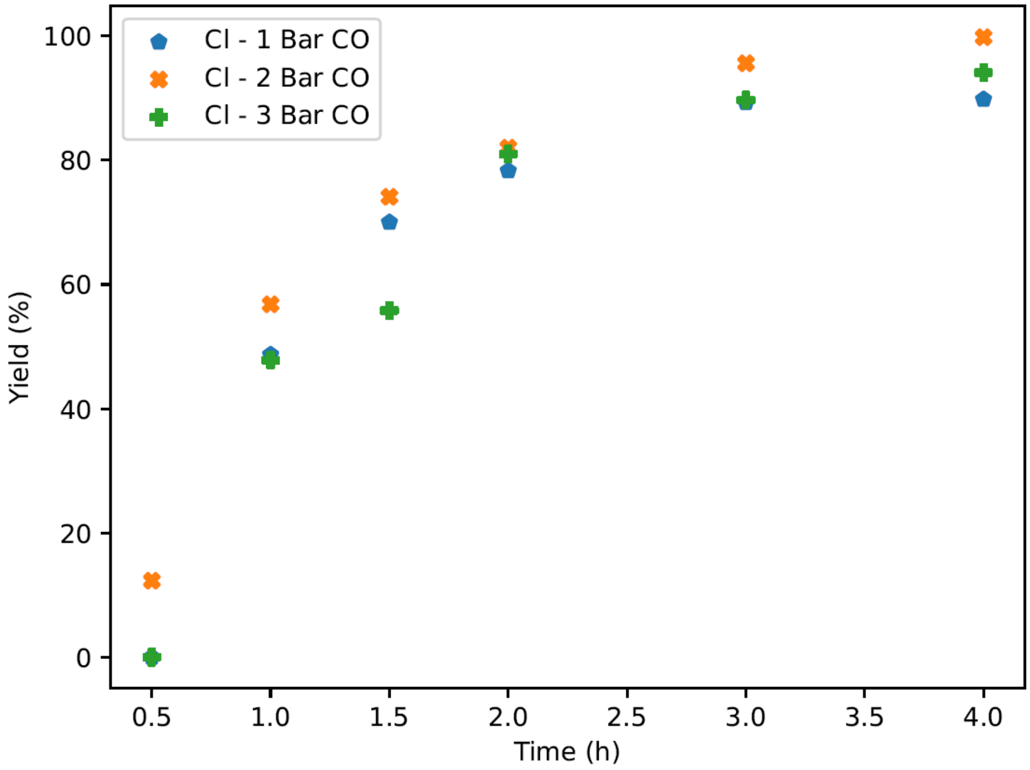

Figure S13: Kinetics for varied CO pressure. R_{para} = Cl

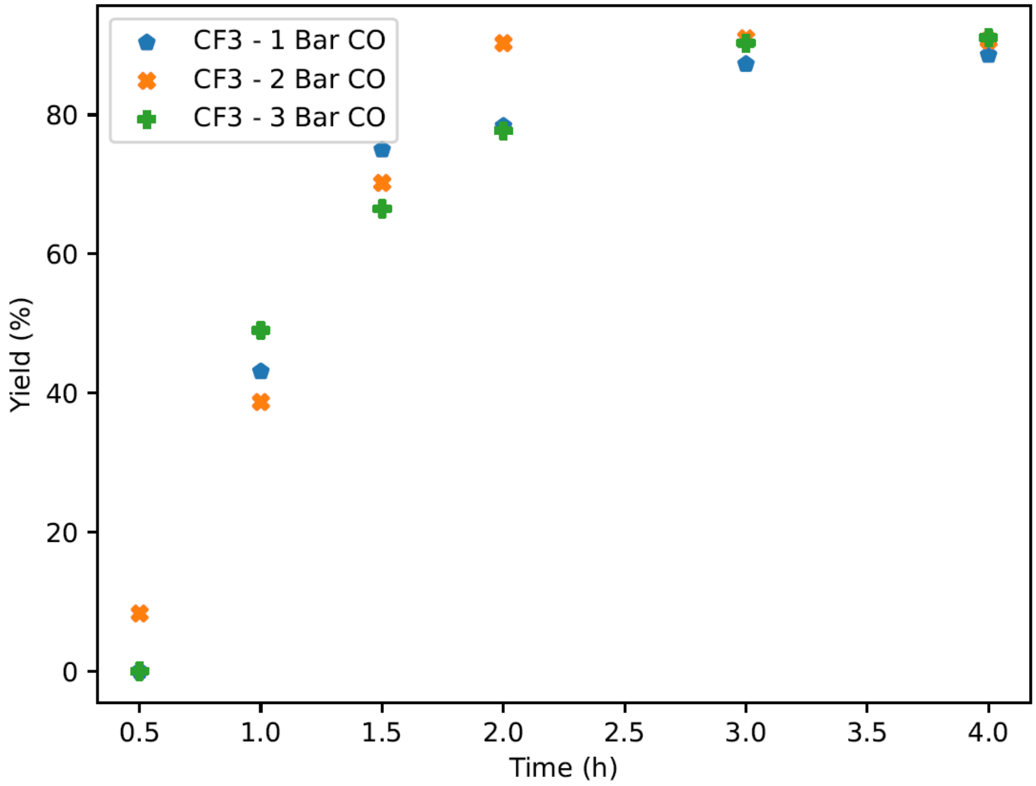

Figure S14: Kinetics for varied CO pressure. $R_{para} = CF_3$

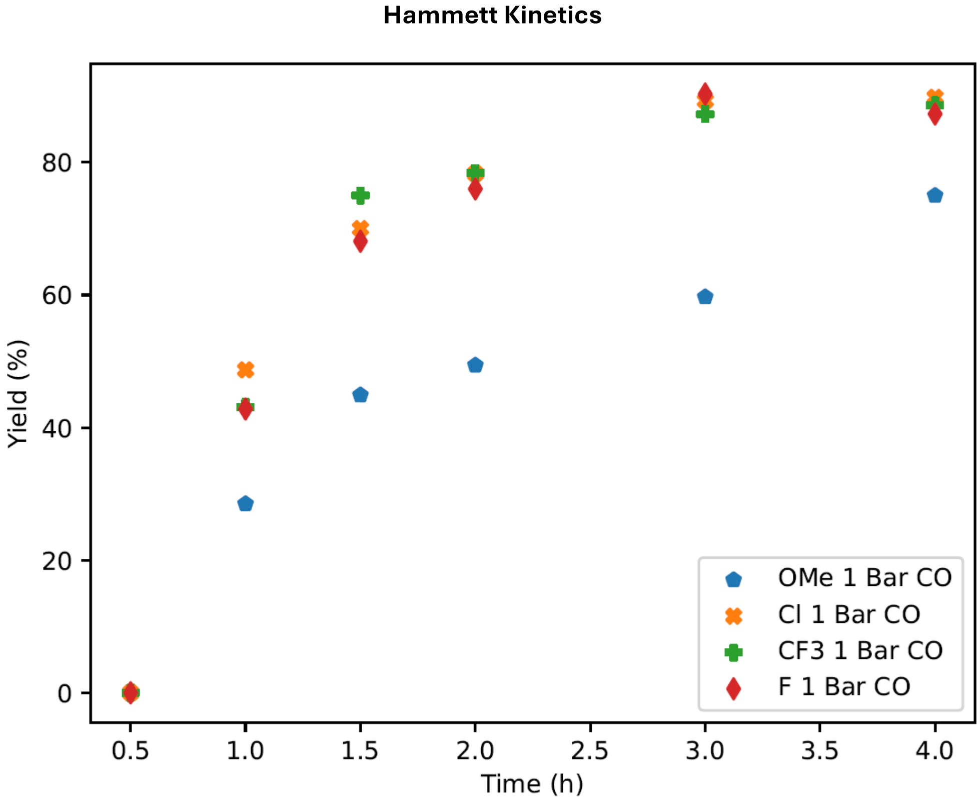

Figure S15: Hammett kinetics for varied para substitutions – 1 Bar CO Pressure

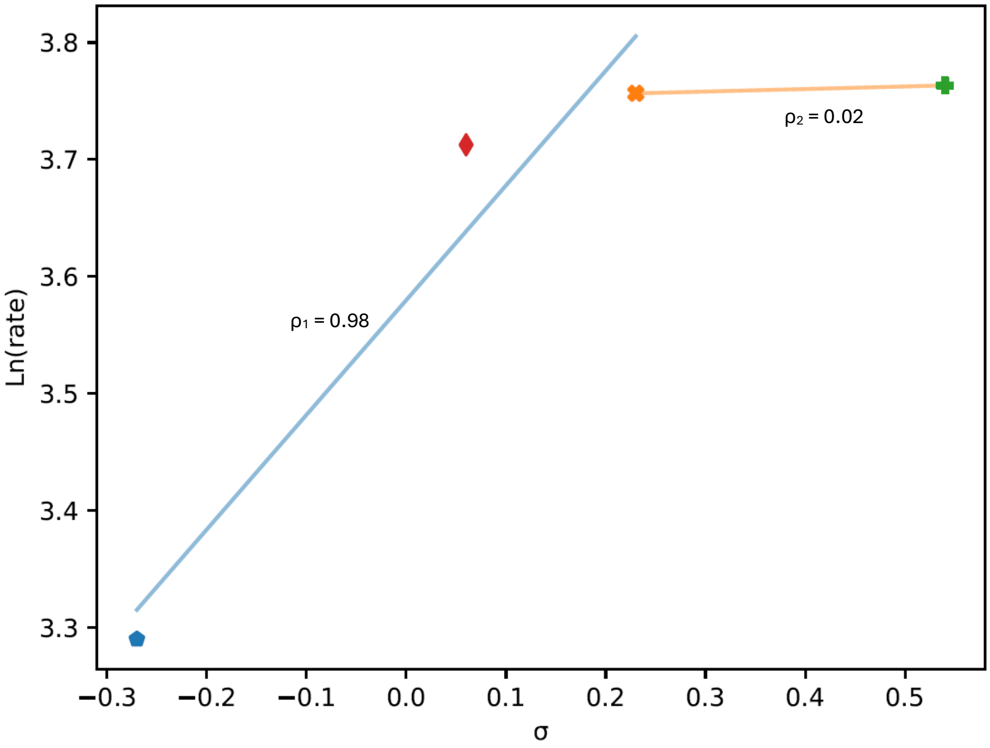

Figure S16: Hammett plot for varied para substitutions – 1 Bar CO Pressure

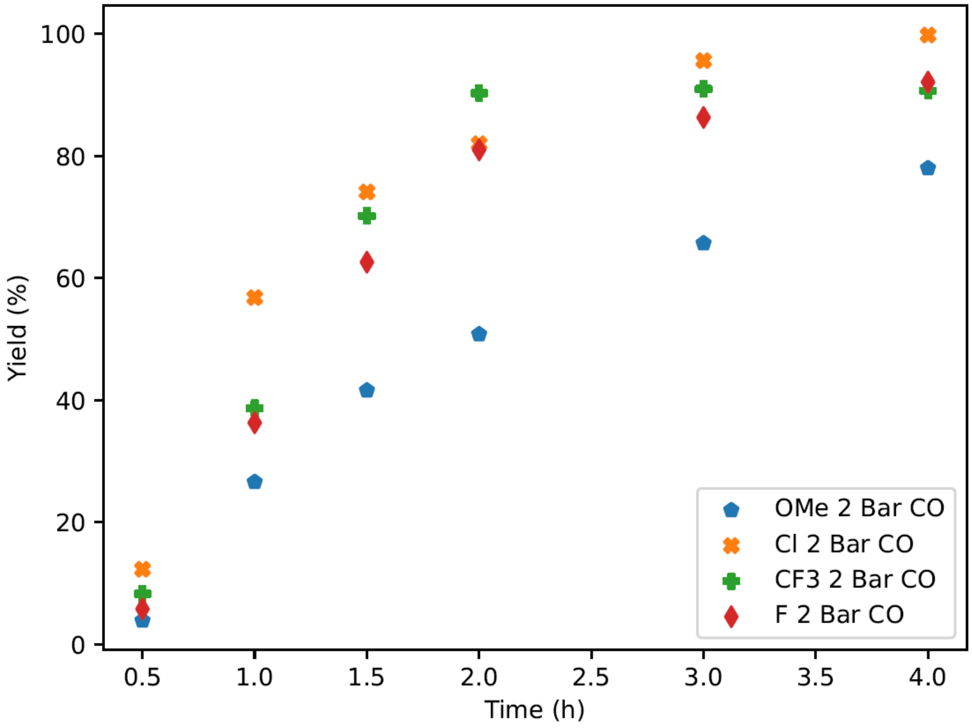

Figure S17: Hammett kinetics for varied para substitutions – 2 Bar CO Pressure

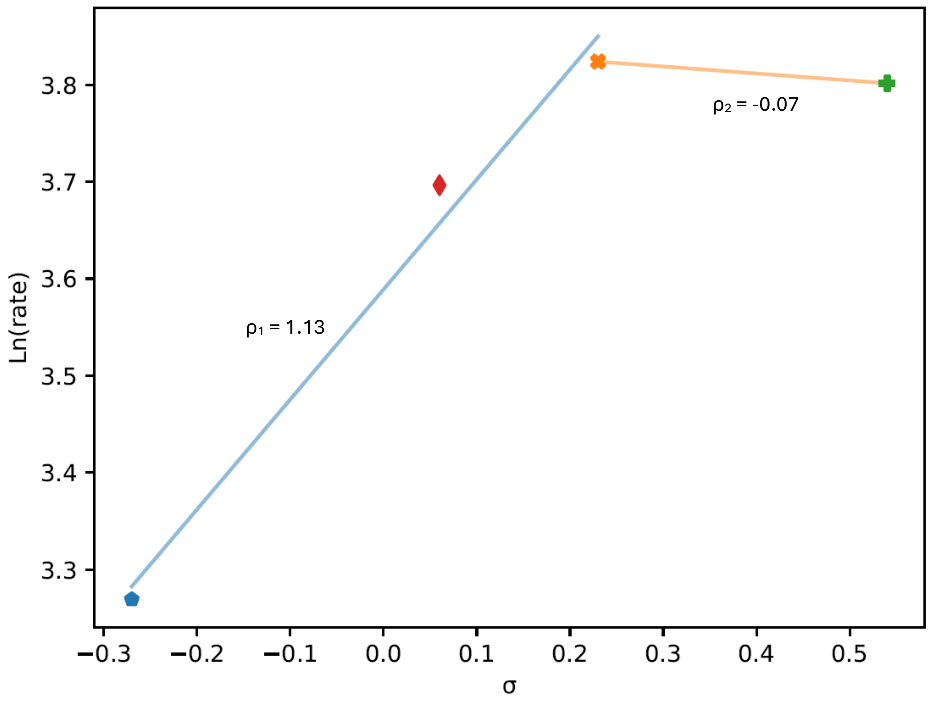

Figure S18: Hammett plot for varied para substitutions – 2 Bar CO Pressure

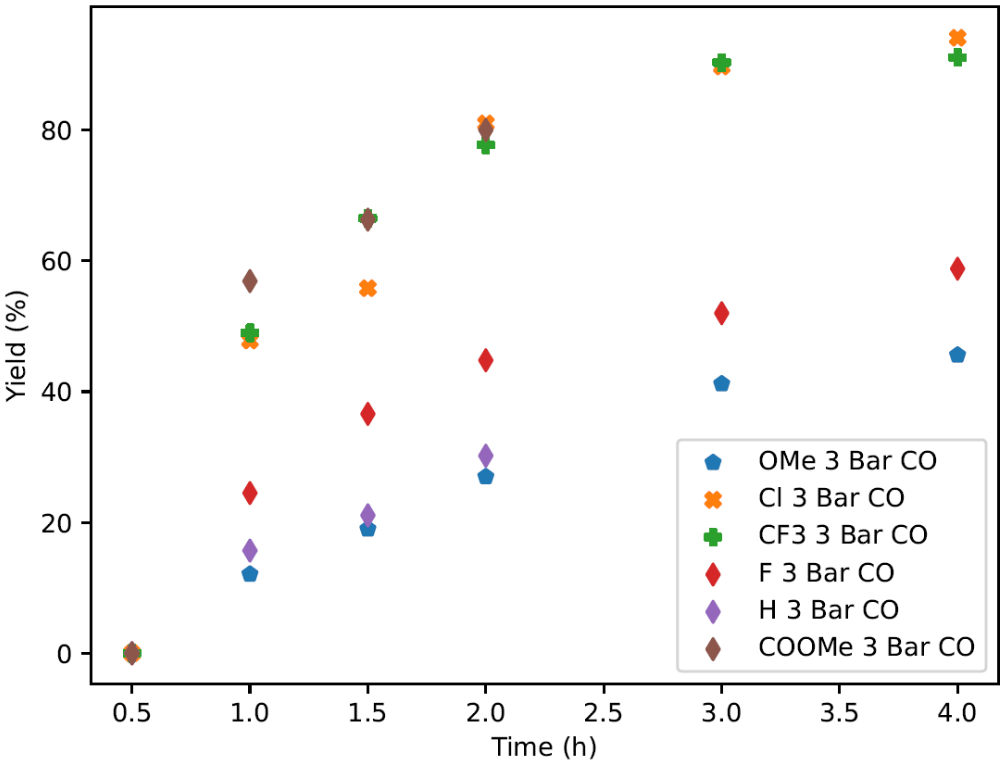

Figure S19: Hammett kinetics for varied para substitutions – 3 Bar CO Pressure

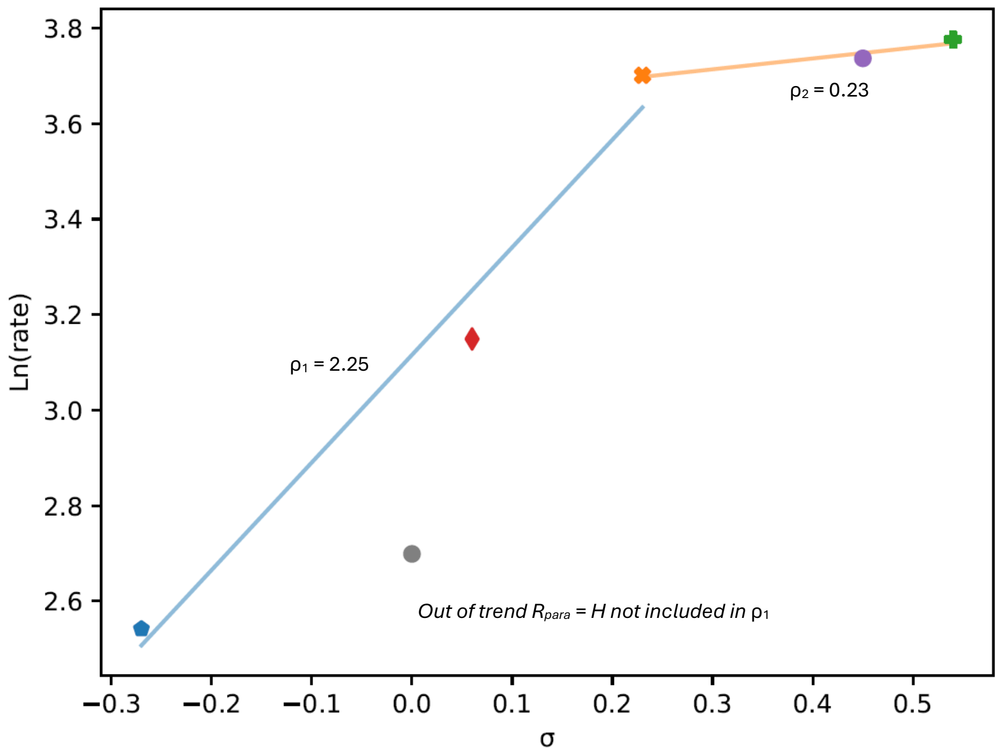

Figure S20: Hammett plot for varied para substitutions – 3 Bar CO Pressure

Miscellaneous Kinetics

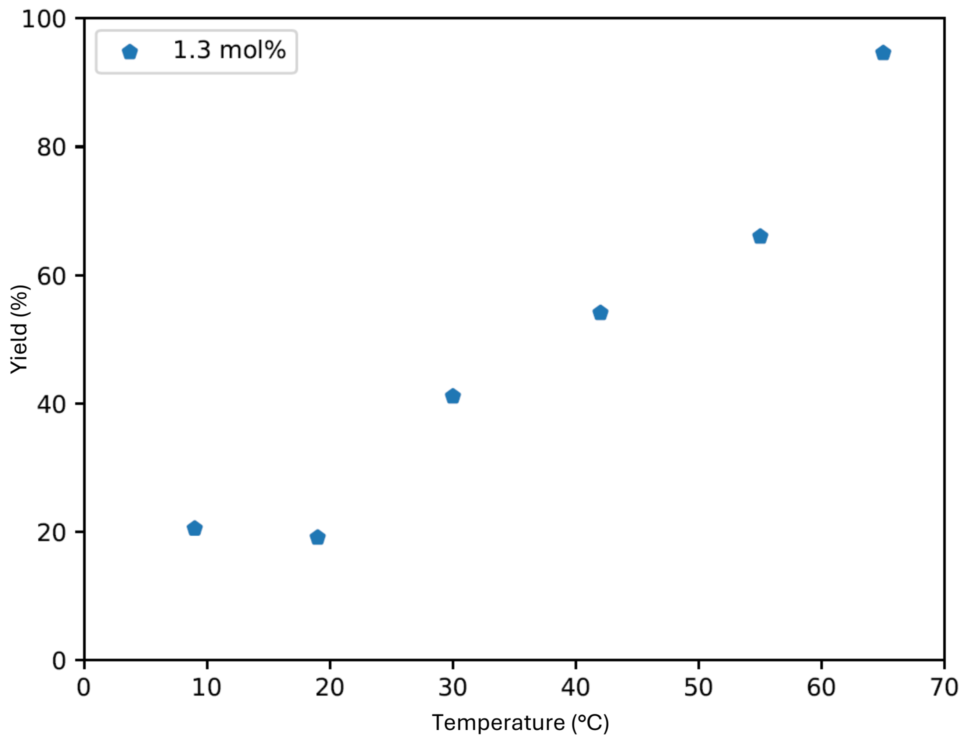

Figure S21: Temperature influence on rate of reaction (R_{para} = OMe, 3 h reaction, 2 Bar CO Pressure, 1.3 mol% [$Co_2(CO)_8$])

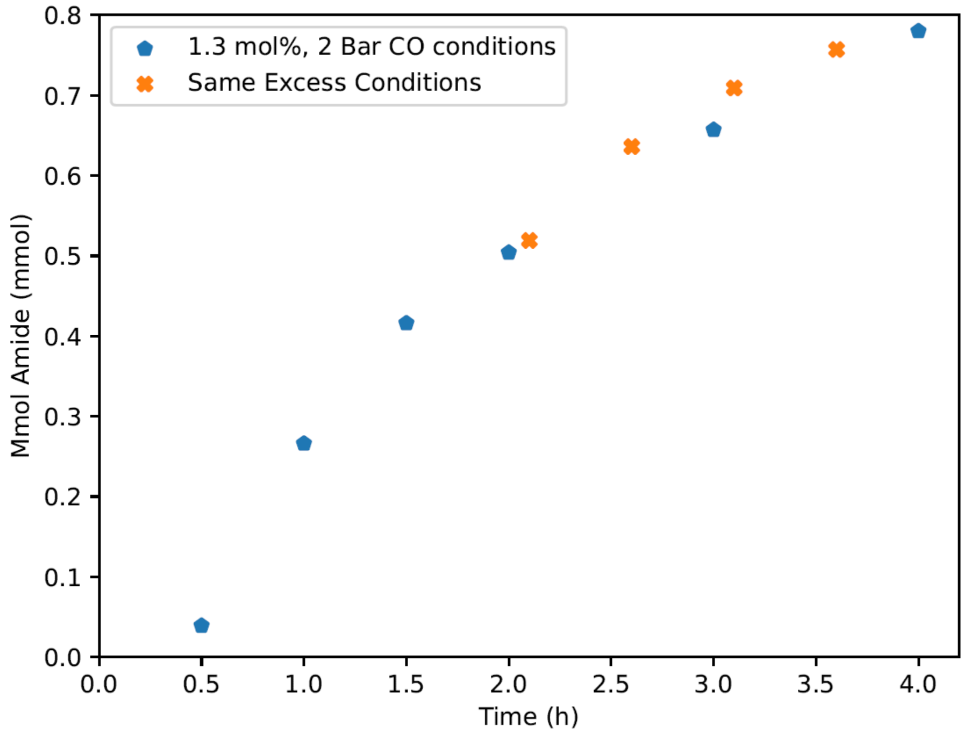

Figure S22: Same Excess Kinetics (R_{para} = OMe, 2 Bar CO Pressure, 1.3 mol% [$Co_2(CO)_8$])

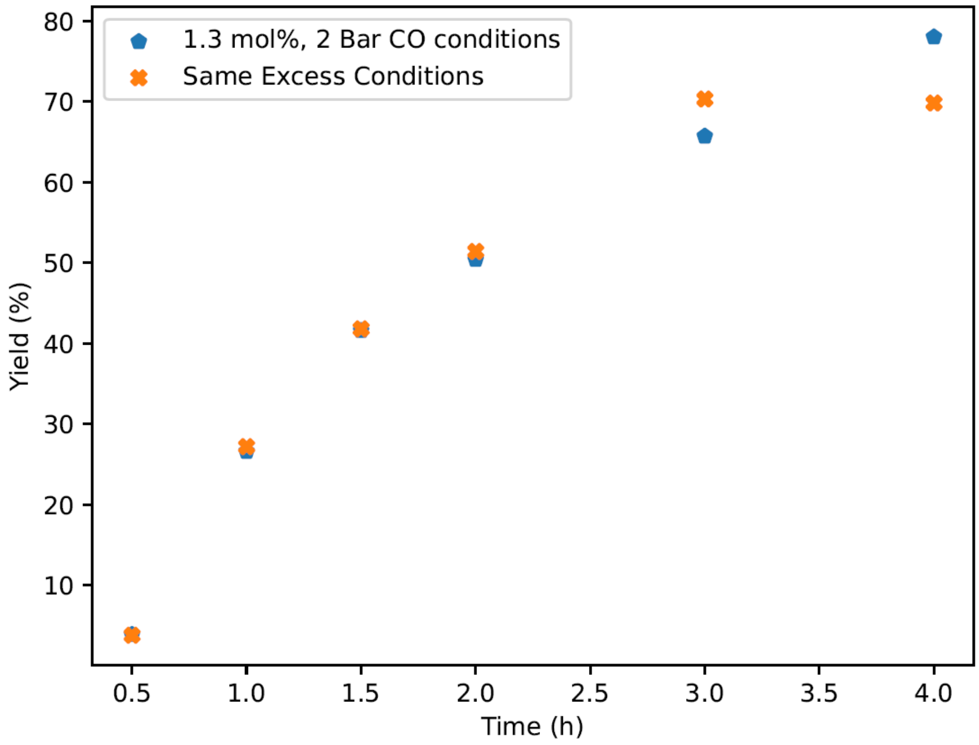

Figure S23: Same Excess Kinetics Yield Comparison (R_{para} = OMe, 2 Bar CO Pressure, 1.3 mol% [$Co_2(CO)_8$])

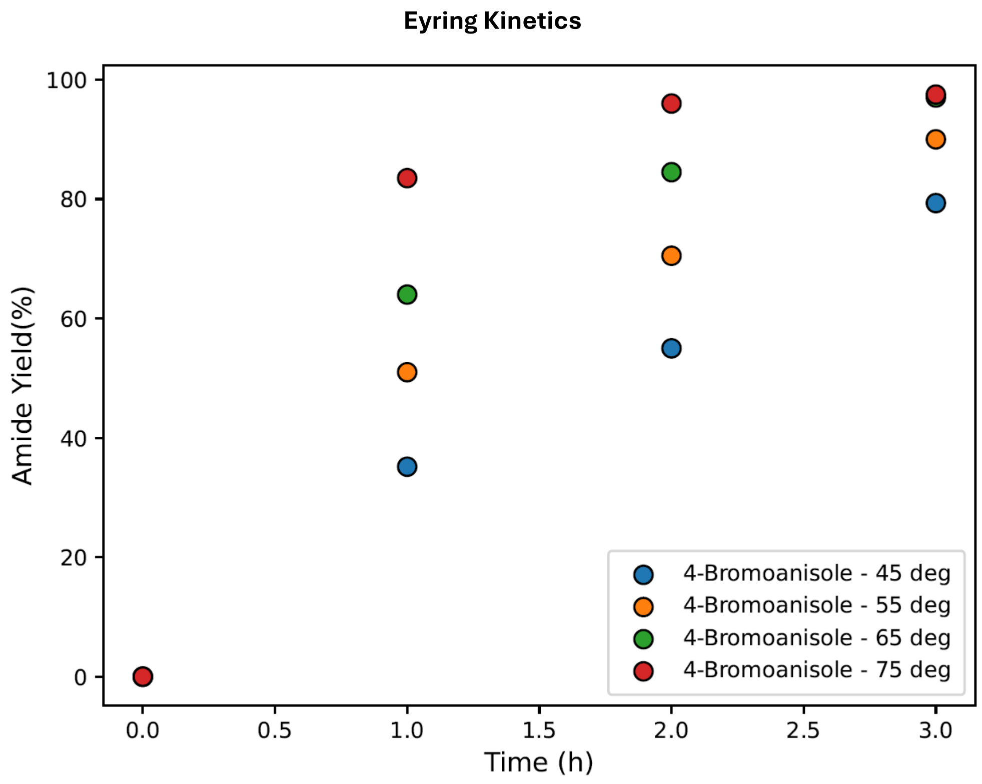

Figure S24: Raw Eyring Kinetics Data Plot (Halogen = Br, R_{para} = OMe, 2 Bar CO Pressure, 1.3 mol% [$Co_2(CO)_8$])

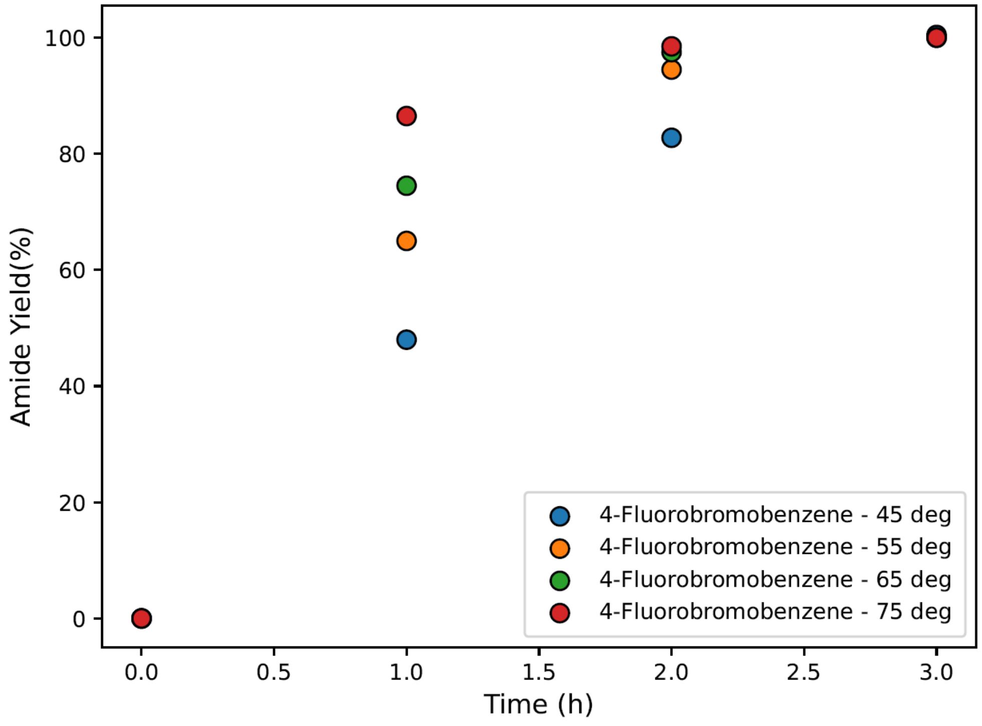

Figure S25: Raw Eyring Kinetics Data Plot (Halogen = Br, R_{para} = F, 2 Bar CO Pressure, 1.3 mol% [$Co_2(CO)_8$])

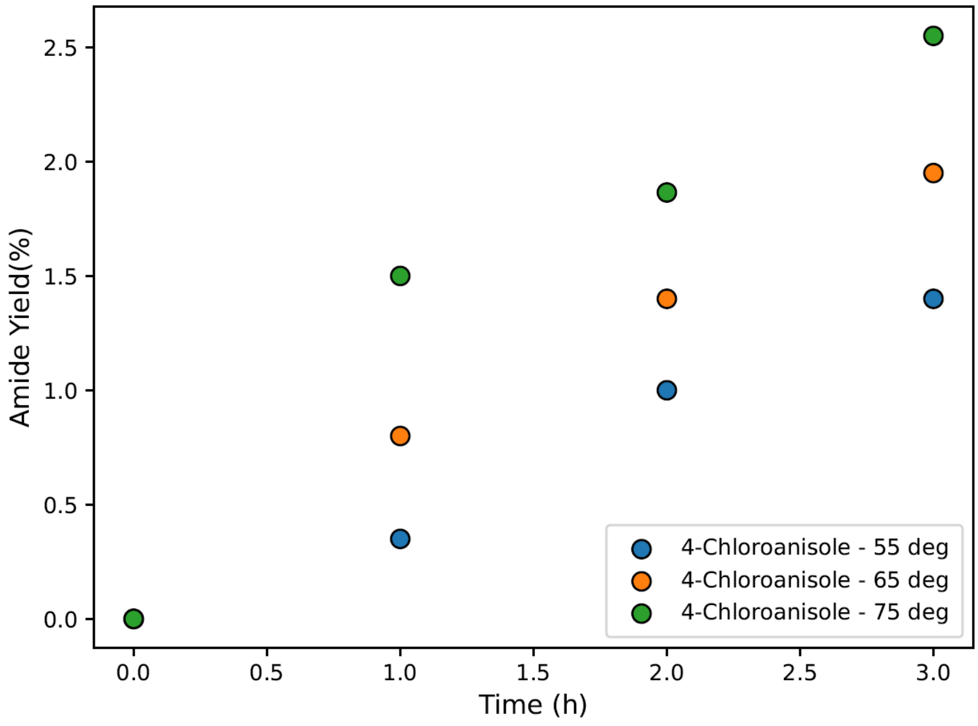

Figure S26: Raw Eyring Kinetics Data Plot (Halogen = Cl, R_{para} = OMe, 2 Bar CO Pressure, 1.3 mol% [$Co_2(CO)_8$])

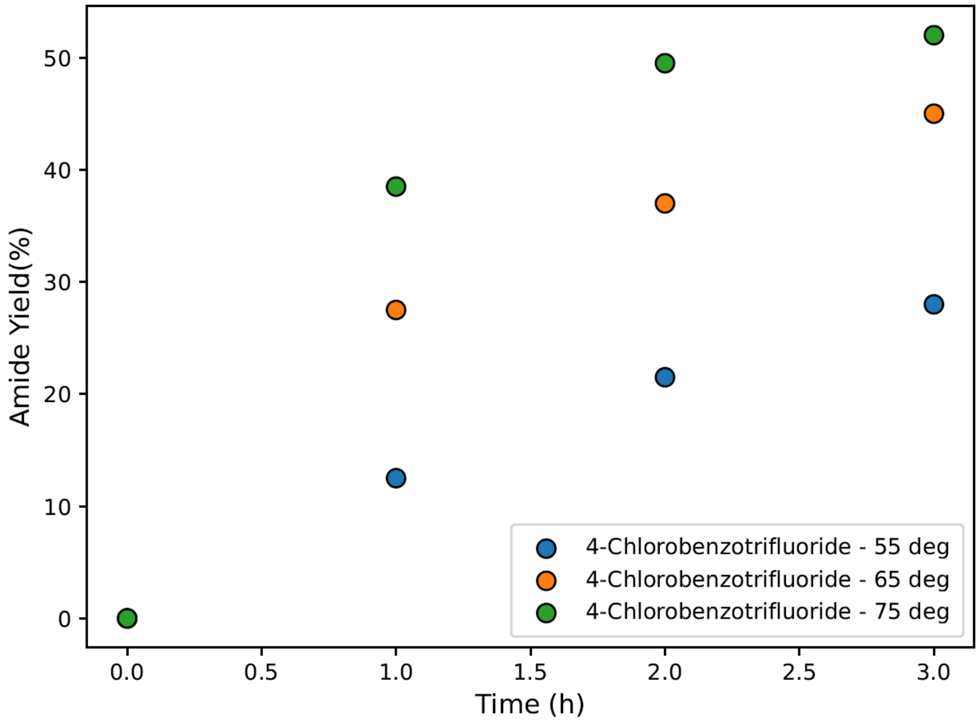

Figure S27: Raw Eyring Kinetics Data Plot (Halogen = Cl, R_{para} = CF_3, 2 Bar CO Pressure, 1.3 mol% [$Co_2(CO)_8$])

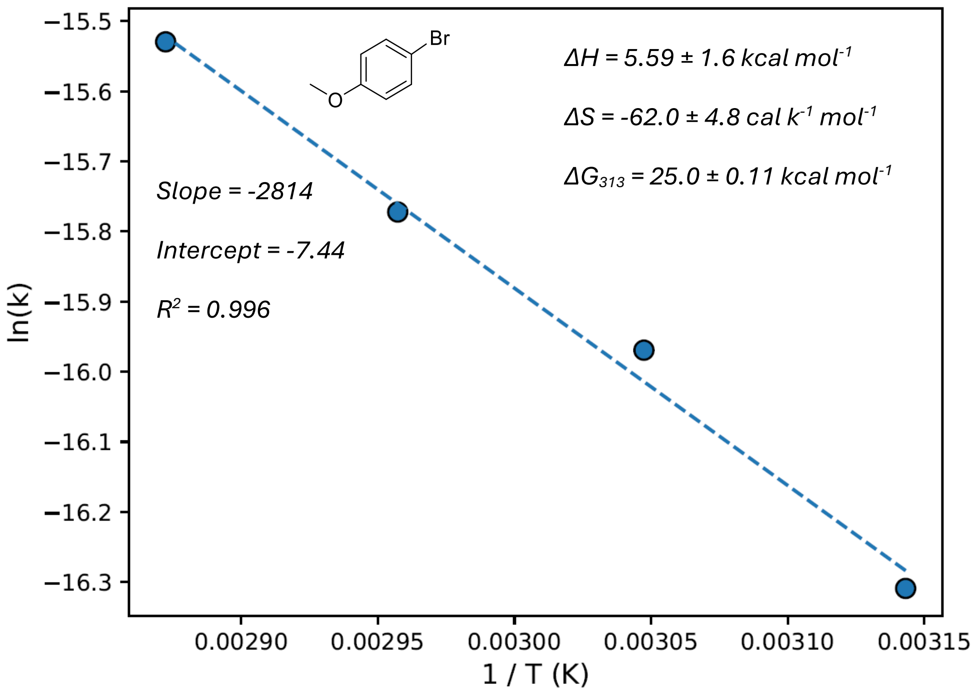

Figure S28: Eyring Kinetics Plot (Halogen = Br, R_{para} = OMe, 2 Bar CO Pressure, 1.3 mol% [$Co_2(CO)_8$])

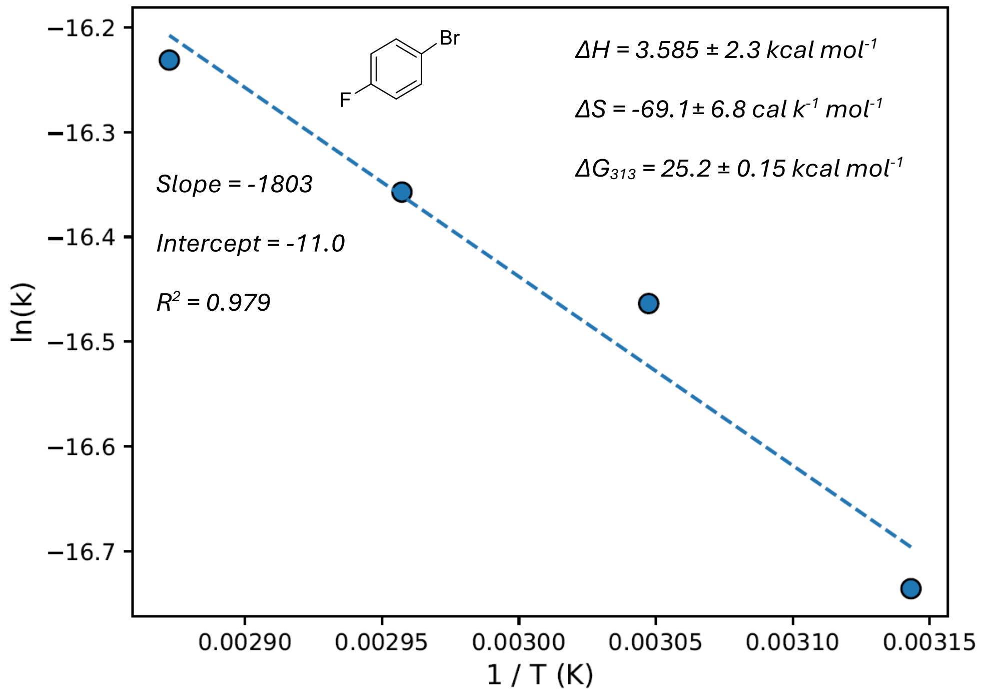

Figure S29: Eyring Kinetics Plot (Halogen = Br, R_{para} = F, 2 Bar CO Pressure, 1.3 mol% [$Co_2(CO)_8$])

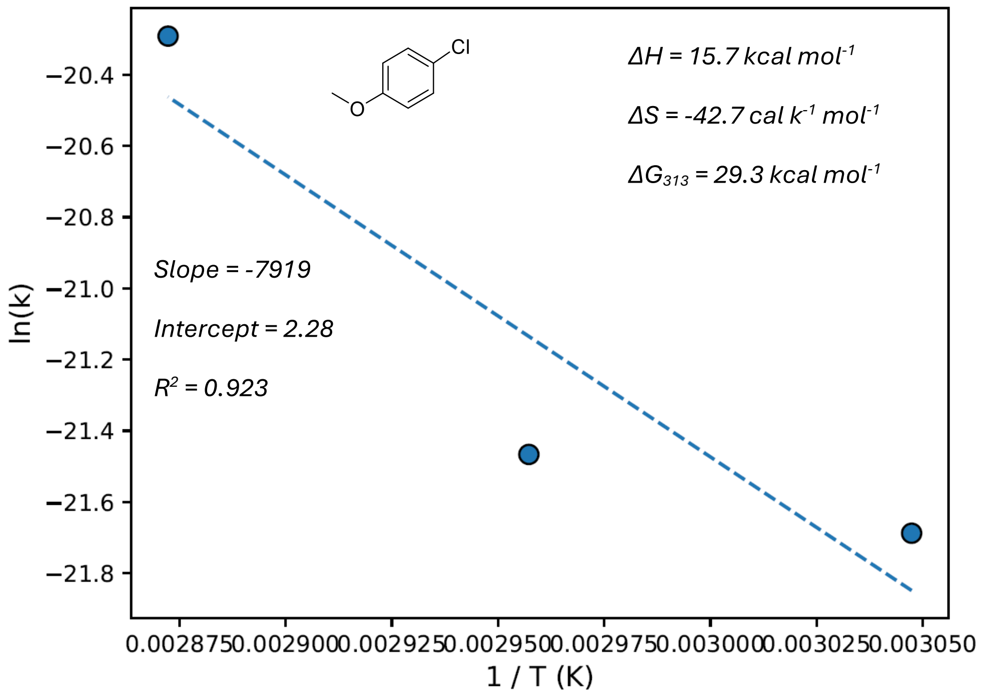

Figure S30: Eyring Kinetics Plot (Halogen = Cl, R_{para} = OMe, 2 Bar CO Pressure, 1.3 mol% [$Co_2(CO)_8$])

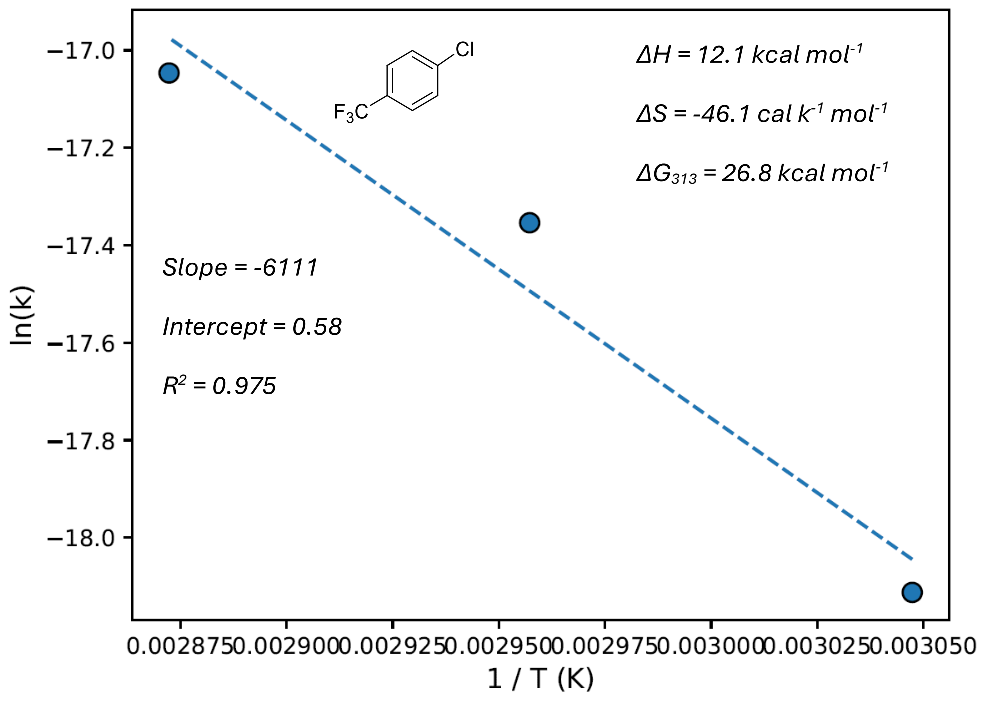

Figure S31: Eyring Kinetics Plot (Halogen = Cl, R_{para} = CF_3, 2 Bar CO Pressure, 1.3 mol% [$Co_2(CO)_8$])

Substrate	Temp (°C)	ΔH (kcal / mol)	ΔS (cal / k mol)	ΔG (kcal / mol)
4-Bromoanisole	45	5.59	-62.0	25.5
4-fluorobromobenzene	45	3.59	-69.1	25.5
4-Chloroanisole	45	15.74	-42.7	29.3

4-Chloroanisole	75	15.74	-42.7	30.6
4-Chlorobenzotrifluoride	45	12.14	-46.1	26.8
4-Chlorobenzotrifluoride	75	12.14	-46.1	28.1

Table S8: Comparison of chloride and bromide Eyring analysis. Chlorides ΔG calculated at 45 & 75 deg to compare operation conditions.

Kinetic Order Determination Tools

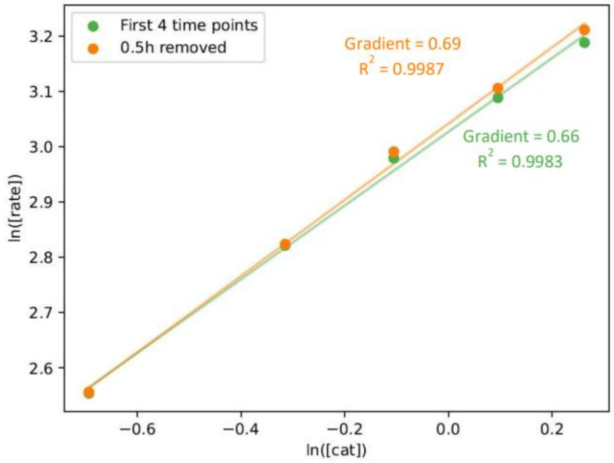

Figure S32: Log-log graphs used for initial catalyst order determination (R_{para} = OMe, 2 Bar CO Pressure)

<u>Auto-VTNA Order Determination: A guide to use.</u>

A formatted excel sheet is loaded into the tool via the 'Browse' Section. The GUI reads the document to determine the number of experiment and headings for quantities of chemicals measured.

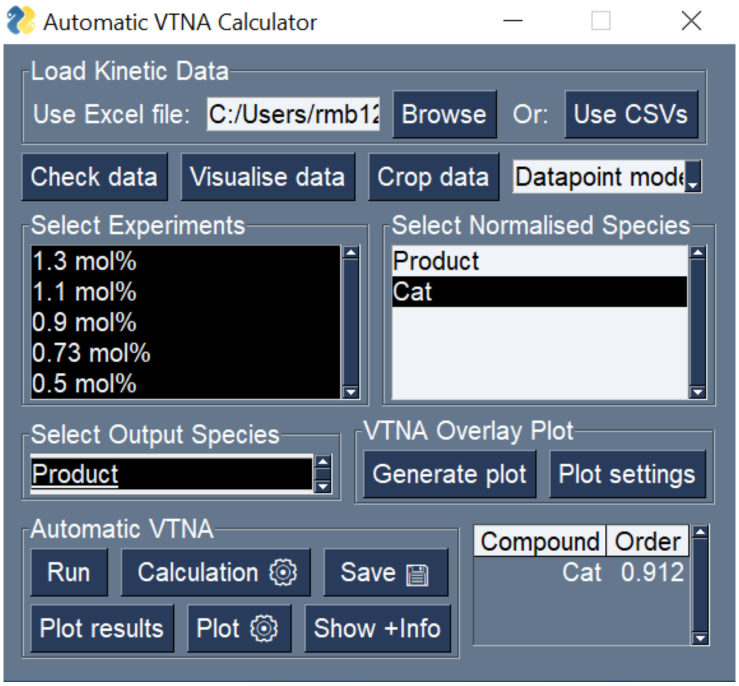

Figure S33: Home panel for Auto-VTNA GUI

From here, the specific datasets can be selected to be included in the calculation and the normalized species defined. The calculation settings can also be configured prior to running. In this case R^2 was selected over RMSE (default) for regression calculations. Subsequently the ‘Run’ button is clicked, giving a value for ‘calculated order in reagent’ in the bottom right. This can be visualized using the ‘plot results’ button.

Subsequently, using the ‘crop data’ button allows for specific removal of datapoints. The interface looks as follows:

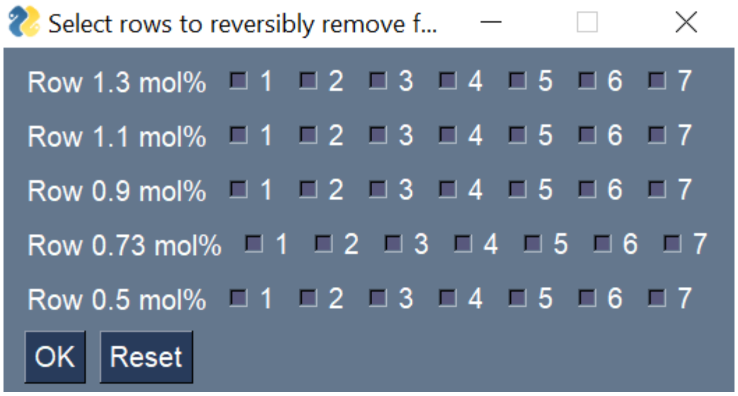

Figure S34: GUI for data cropping within Auto-VTNA tool

As discussed in the determination of catalyst order kinetics, this cropping gave rise to knowledge on the robustness of catalyst order dependent on data cropping. In our case, it was shown that removal of the induction period increased our order value for [catalyst] while removal of later kinetic data decreased it, in alignment with the findings of the log-log graphs, derived from initial rates. From this tool we were able to determine unique plots for each of the reagent contributions towards rate of reaction.

Auto-VTNA Order Determination Plots

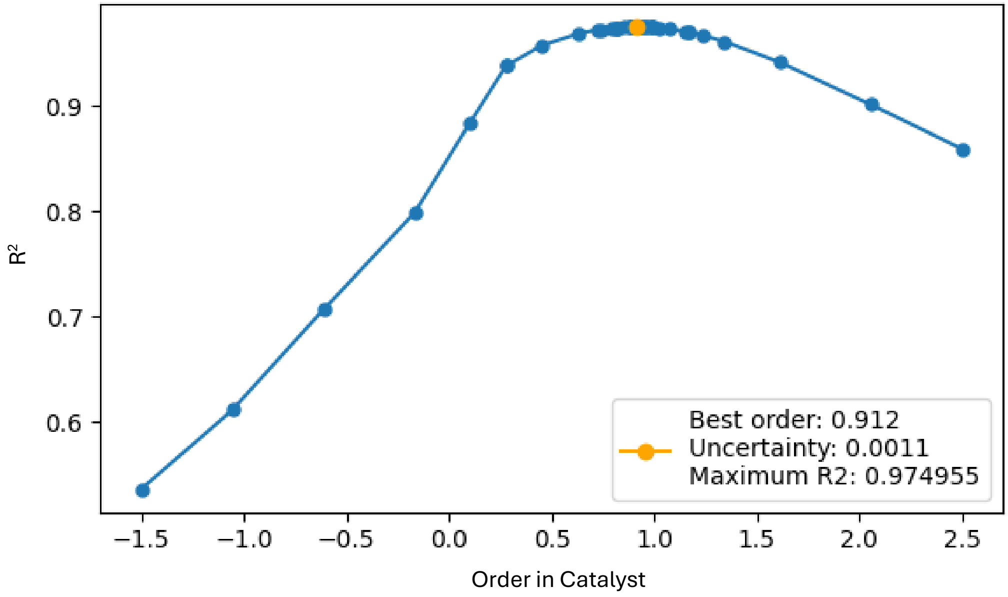

Figure S35: Auto-VTNA Catalyst order determination (R_{para} = OMe)

Kinetic Points Used	Order in Catalyst from Auto-VTNA
All Points	0.912
Deactivation Points Removed (4 points)	0.748
Induction Period Removed (5 points)	1.091
Deactivation and Induction Removed (9 points)	0.843

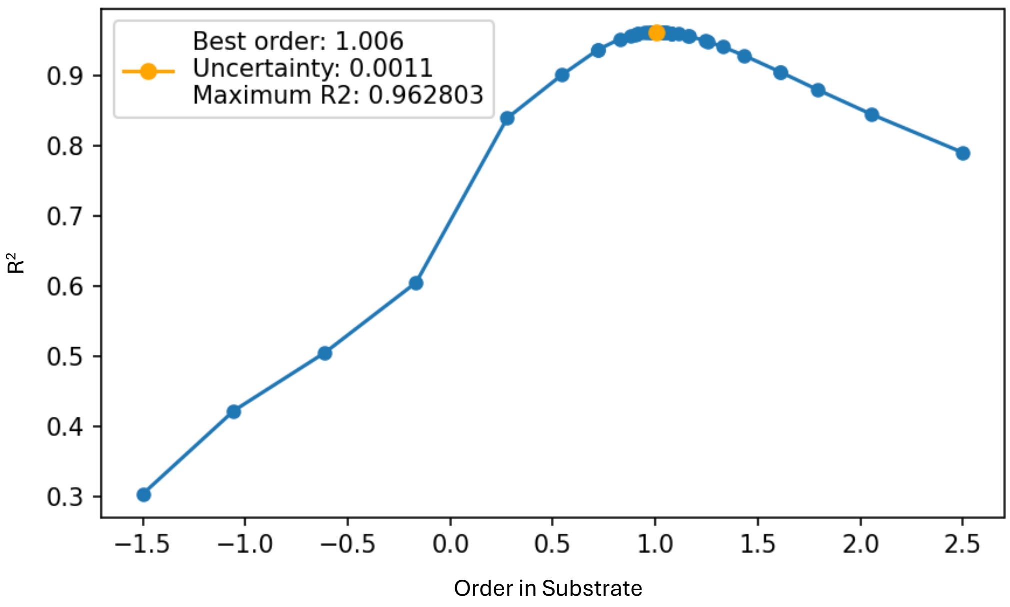

Figure S36: Auto-VTNA Substrate order determination (R_{para} = OMe)

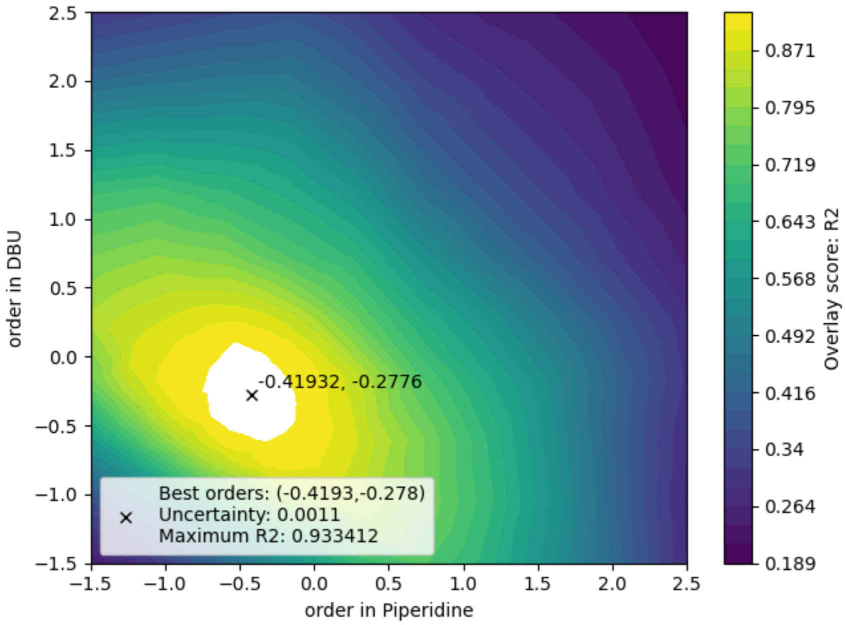

Figure S37: Auto-VTNA Base and nucleophile order determination (R_{para} = OMe)

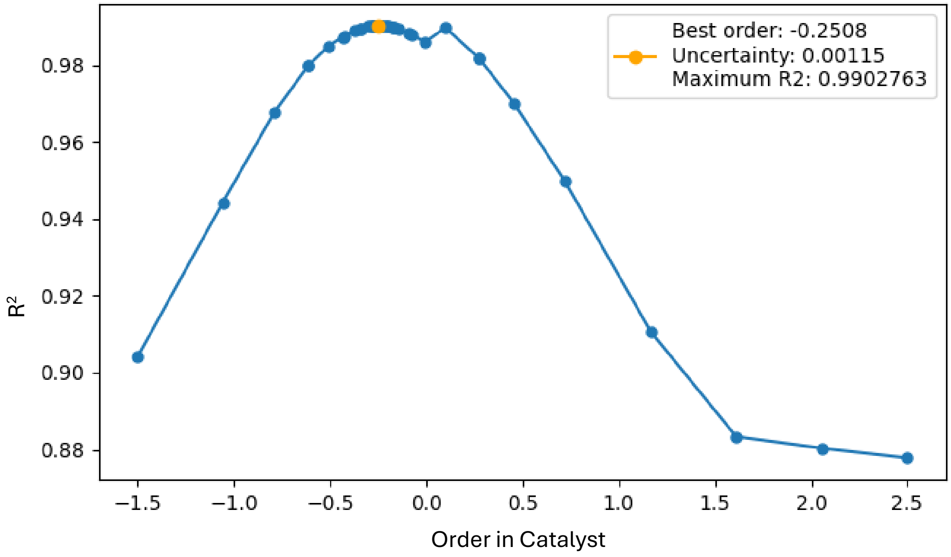

Figure S38: Auto-VTNA Catalyst order determination (R_{para} = CF_3)

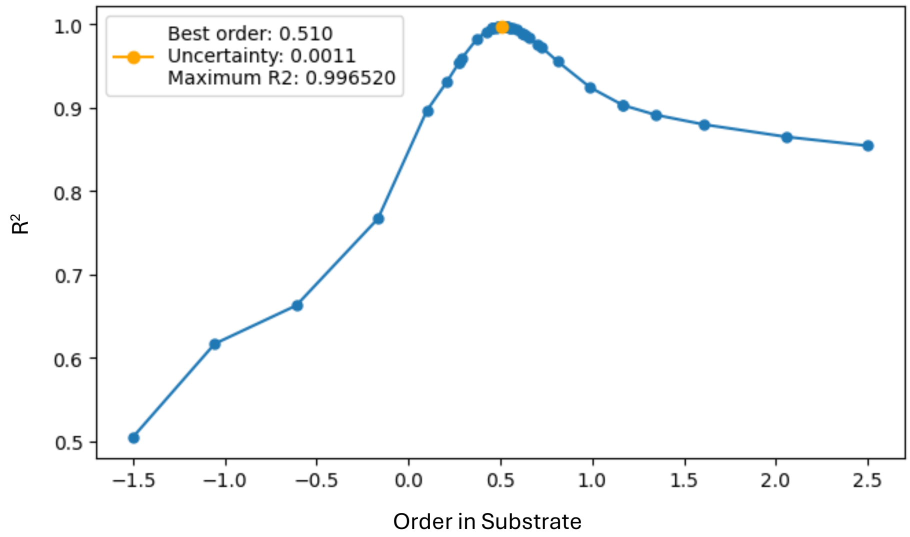

Figure S39: Auto-VTNA Substrate order determination (R_{para} = CF_3)

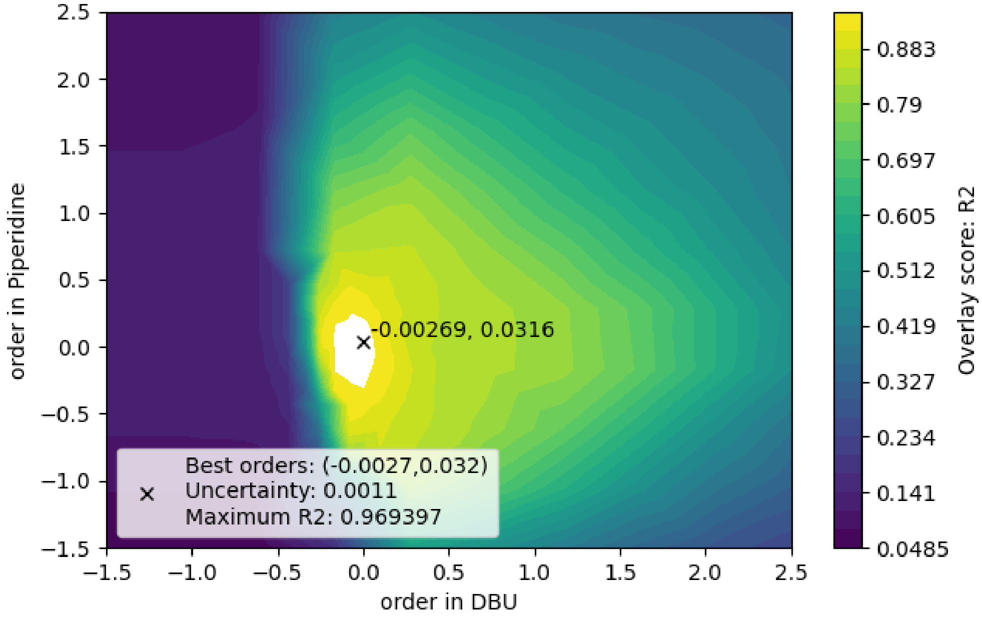

Figure S40: Auto-VTNA Base and nucleophile order determination (R_{para} = CF_3)

CO Order Kinetics (R_{para} = OMe, F, Cl, CF_3)

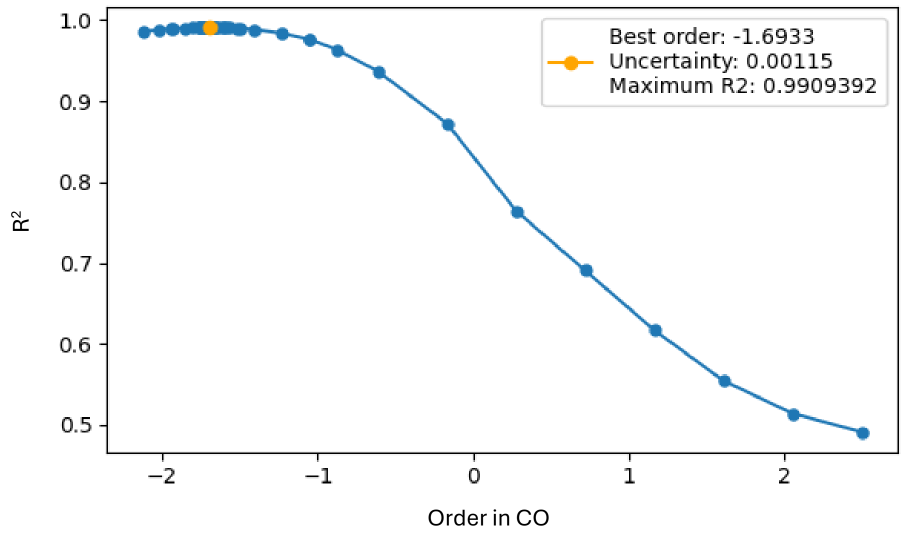

Figure S41: Auto-VTNA CO order determination (R_{para} = OMe)

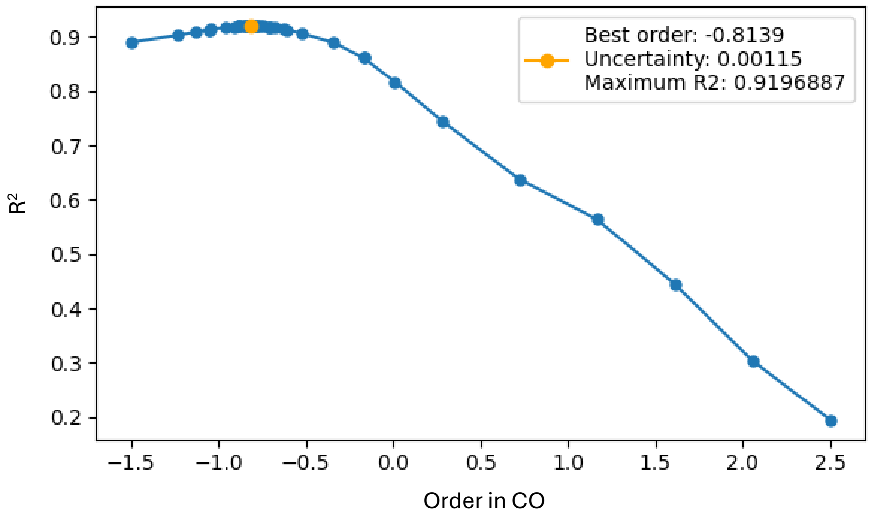

Figure S42: Auto-VTNA CO order determination (R_{para} = F)

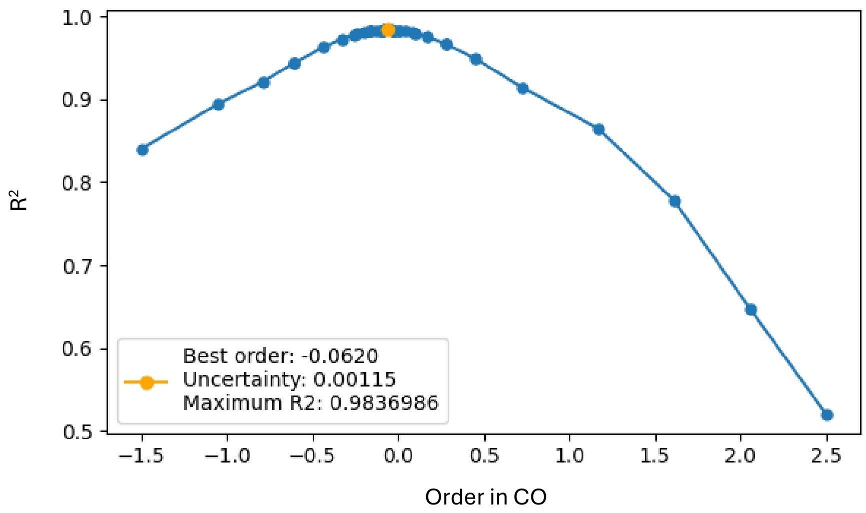

Figure S43: Auto-VTNA CO order determination (R_{para} = Cl)

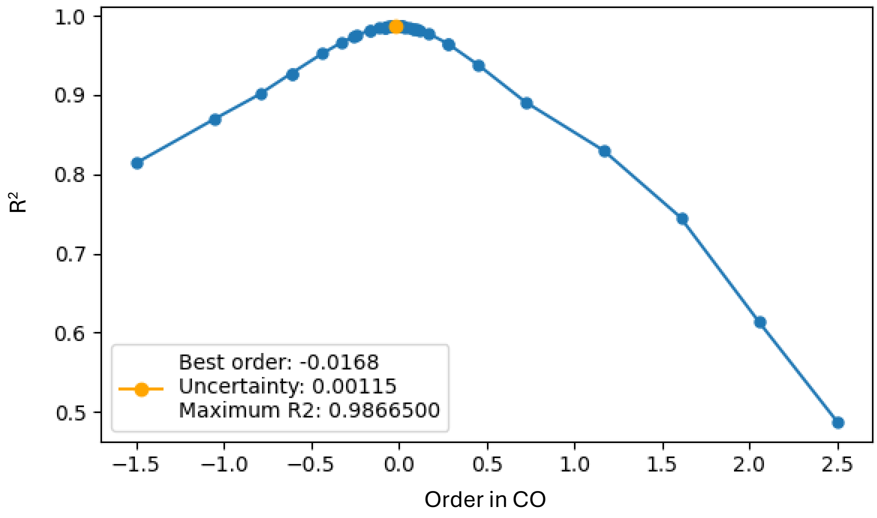

Figure S44: Auto-VTNA CO order determination (R_{para} = CF_3)

^{59}Co NMR Data

Synthesis of $K[Co(CO)_4]$

To an ampoule in an N_2 filled glovebox was added a stirrer bar, $Co_2(CO)_8$ (0.75 g, 2.2 mmol) and KOH (4.5 g, 80 mmol, 36 eq.). The ampoule was removed from the glovebox and dry THF (15 mL) was subsequently added via cannula transfer. Upon addition of solvent there was a significant degree of gas evolution. The reaction was left open to the nitrogen bubbler to limit pressure build up. After 16 h, stirring was halted and the reaction solution was transferred into a fresh ampoule via cannula filtration. Removal of solvent from the yellow THF solution in vacuo produced a sticky off-white solid. Azeotropic removal of remnant THF using n-pentane yielded the product as a dry free-flowing off-white powder (0.479g, 51% yield). IR (ATR) = 1848 cm^{-1}, ^{13}C NMR (126 MHz, THF): δ 223.79 – 206.49 (oct, J = 277.2 Hz), ^{59}Co NMR (118 MHz, THF): δ -3011.5 (s, J = 282.02 (^{13}C satellites)). Analytical data was in alignment with literature values.[3, 4]

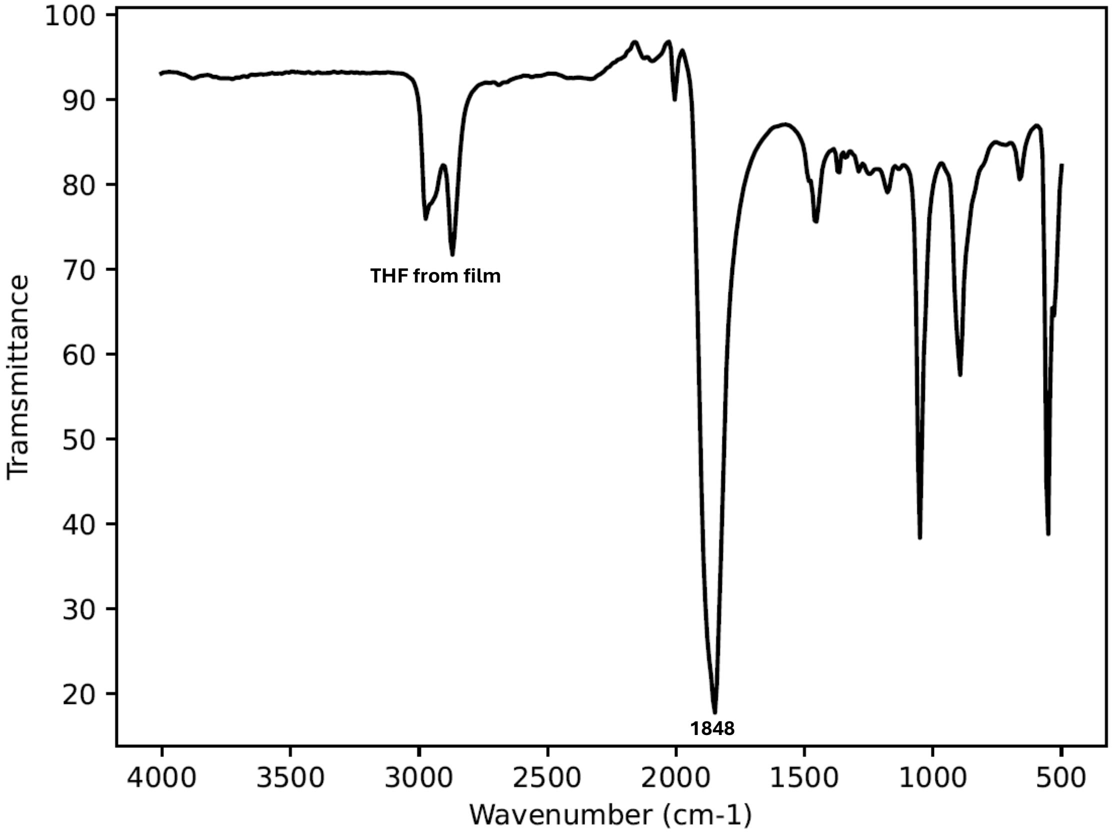

Figure S45: IR analysis of $K[Co(CO)_4]$ (THF film). Peaks were in agreement with literature.[1]

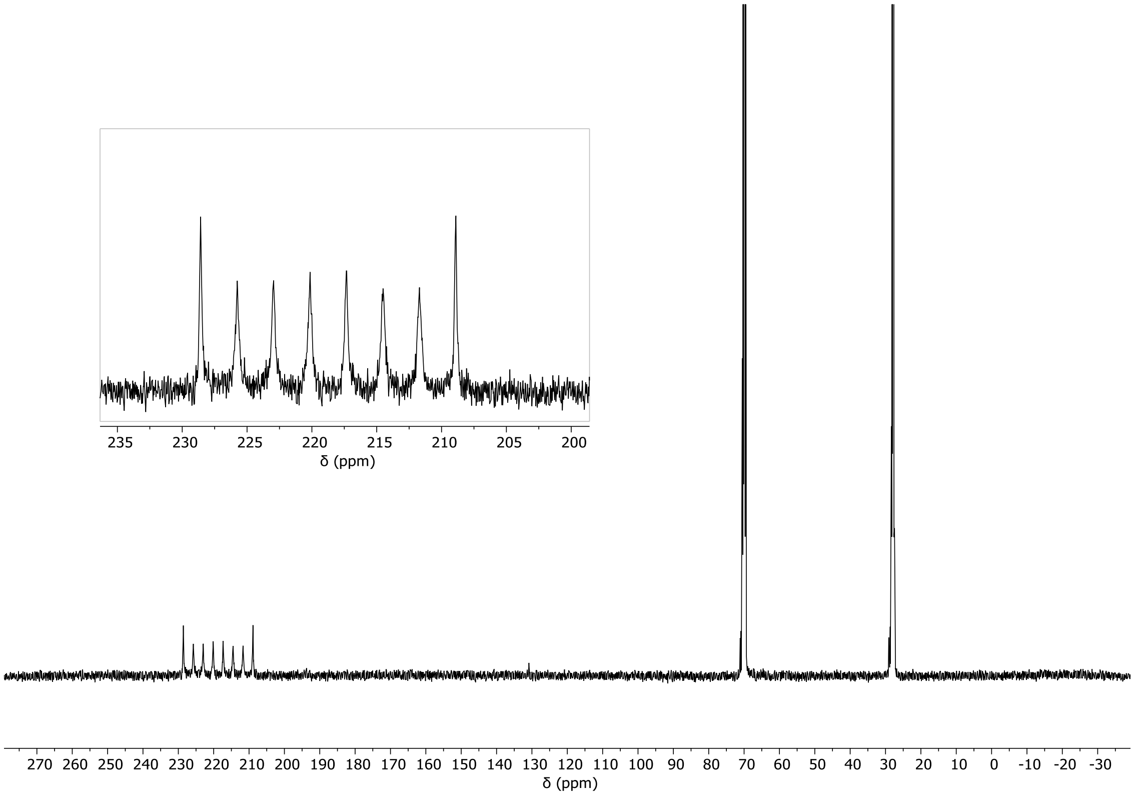

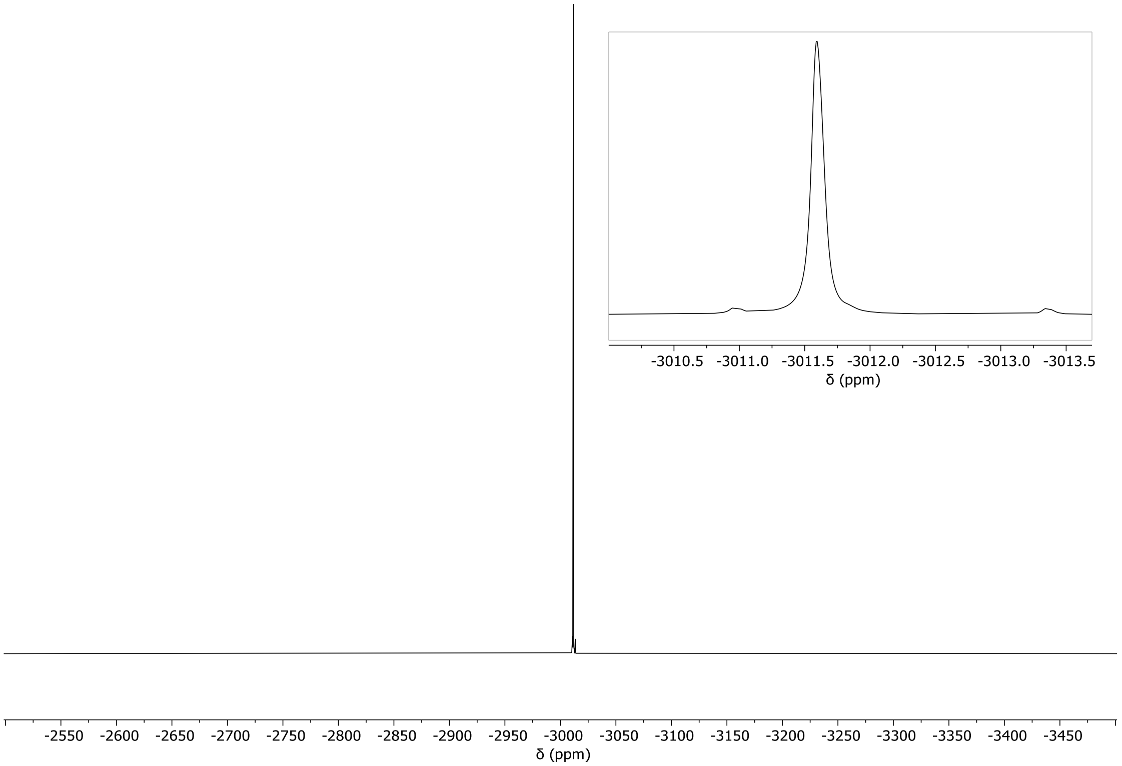

Figure S46: NMR Spectroscopy analysis of K[Co(CO)$_4$]. ^{13}C NMR (top), ^{59}Co NMR (bottom)

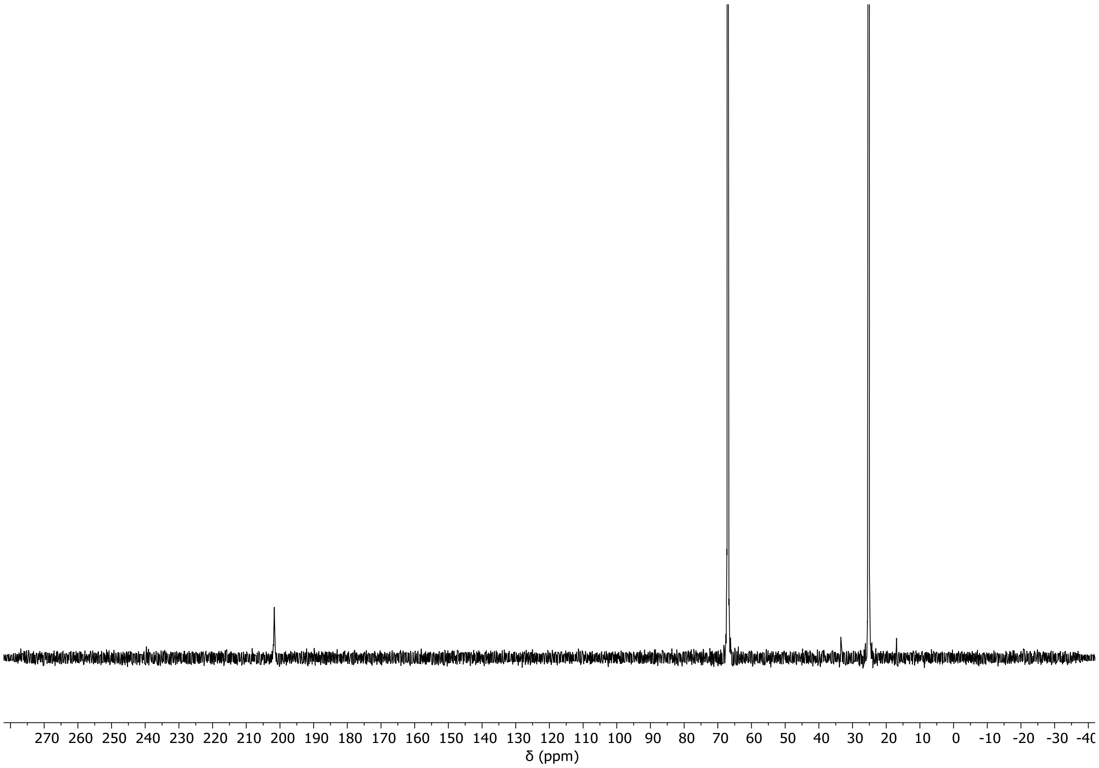

Figure S47: ^{13}C NMR Spectroscopy analysis of [$Co_2(CO)_8$].

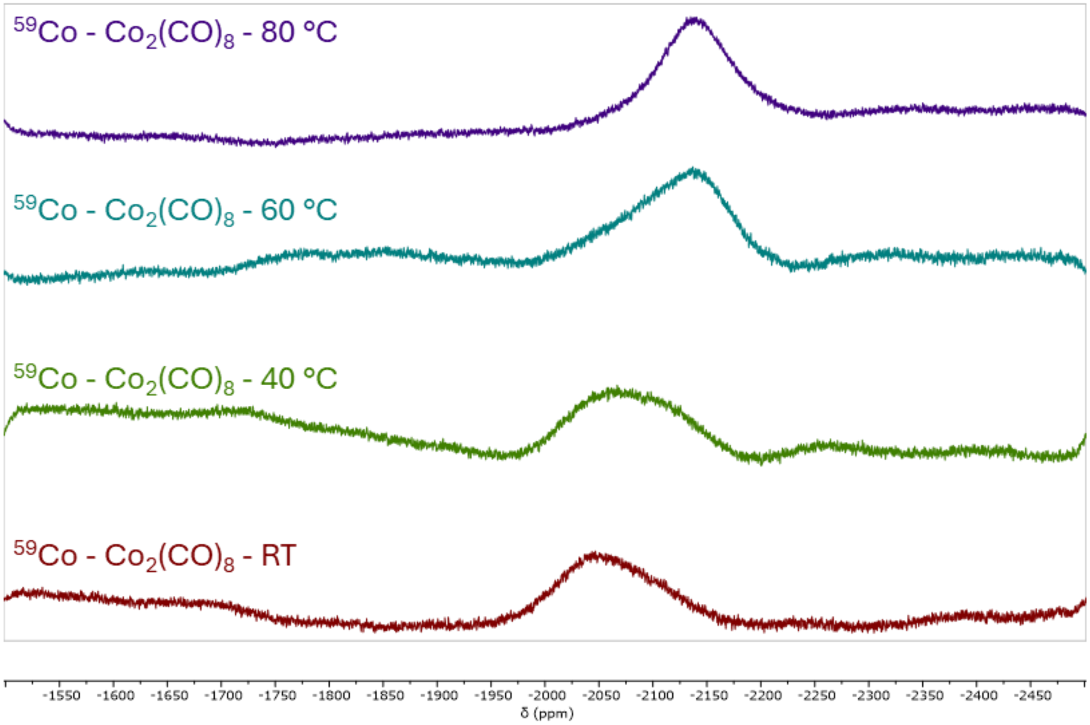

Figure S48: [59]Co NMR Spectroscopy analysis of [$Co_2(CO)_8$] at variable temperatures.

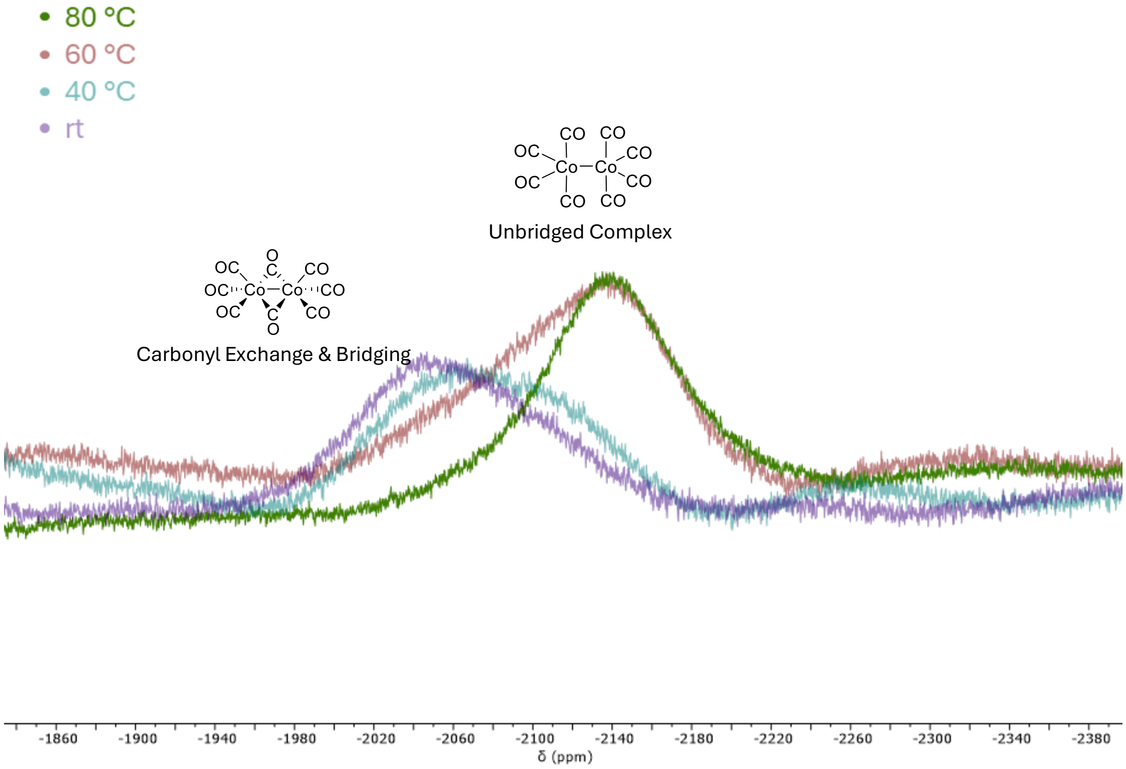

Figure S49: Overlayed [59]Co NMR Spectroscopy analysis of [$Co_2(CO)_8$] at variable temperatures.

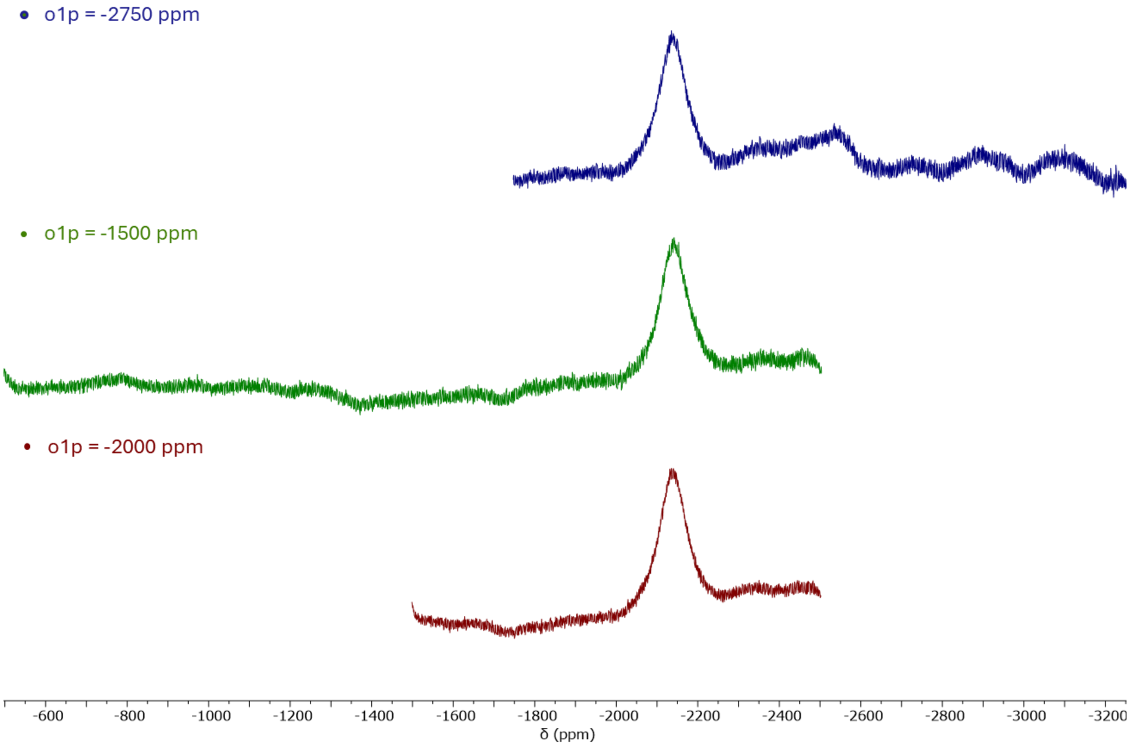

Figure S50: Offset testing of [59]Co NMR Spectroscopy analysis of [$Co_2(CO)_8$].

^{59}Co NMR Analysis Reaction Spectra – [$Co_2(CO)_8$]

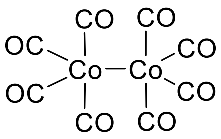

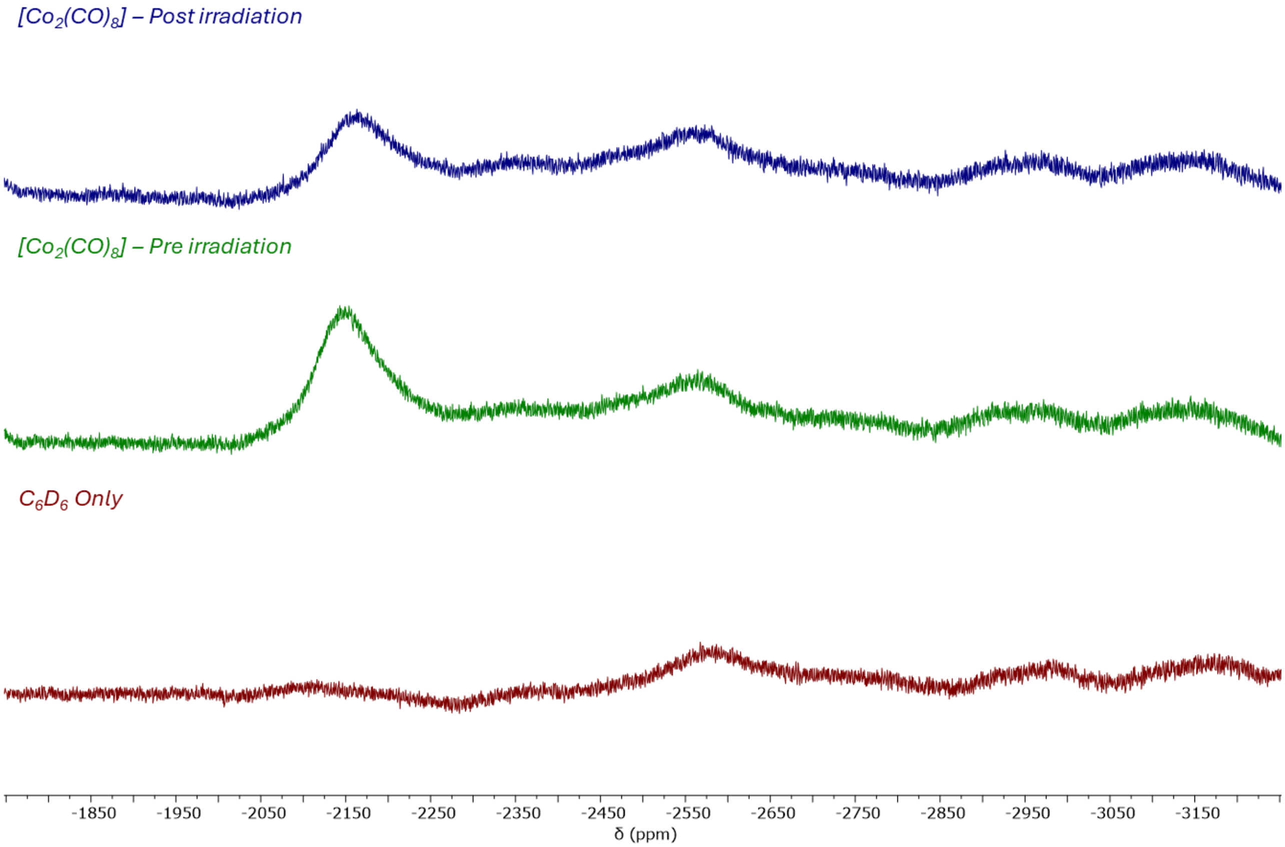

Figure S51: ^{59}Co NMR analysis of [$Co_2(CO)_8$] pre and post irradiation (365 nm LED, 10 W)

^{59}Co NMR Analysis Reaction Spectra – [$Co_2(CO)_8$] + Piperidine (1:1)

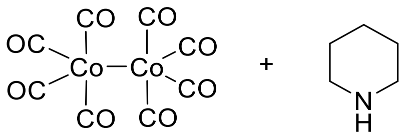

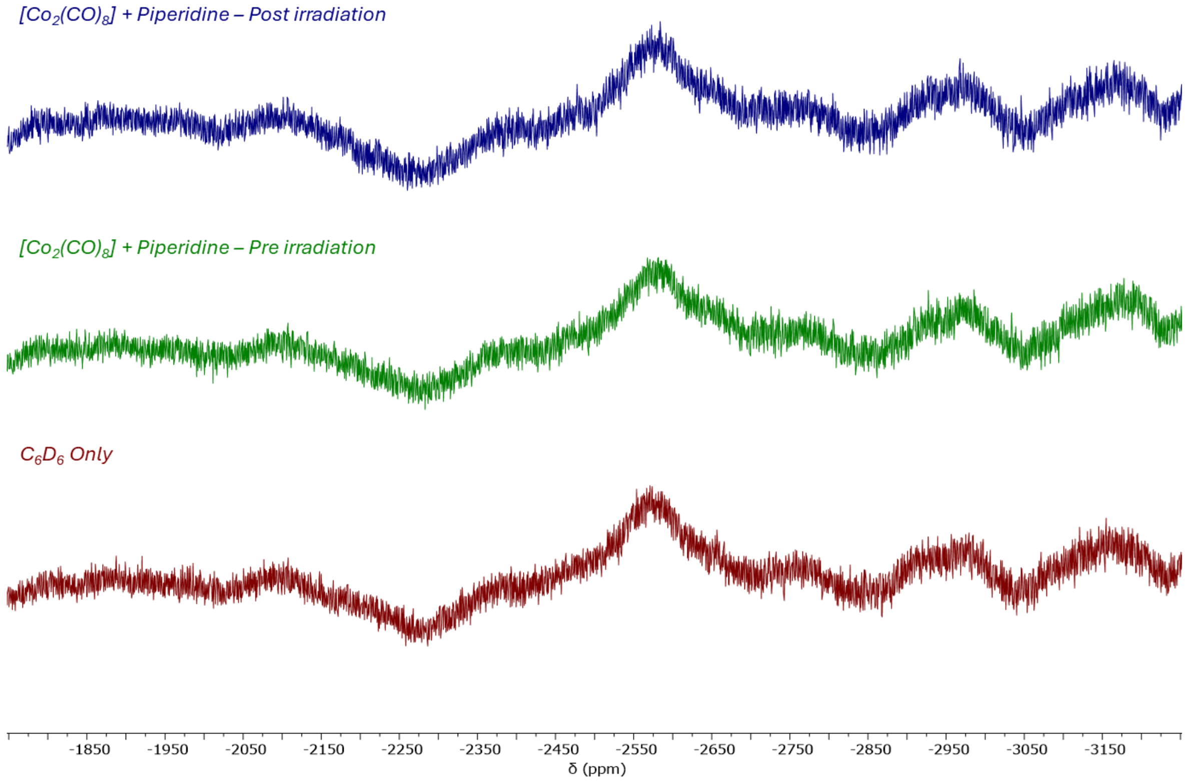

Figure S52: ^{59}Co NMR analysis of [$Co_2(CO)_8$] & piperidine, pre and post irradiation (365 nm LED, 10 W)

^{59}Co NMR Analysis Reaction Spectra – [$Co_2(CO)_8$] + Piperidine (1:20)

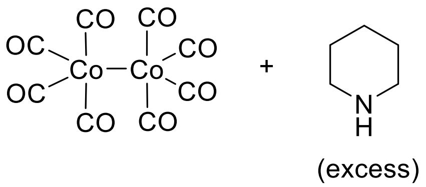

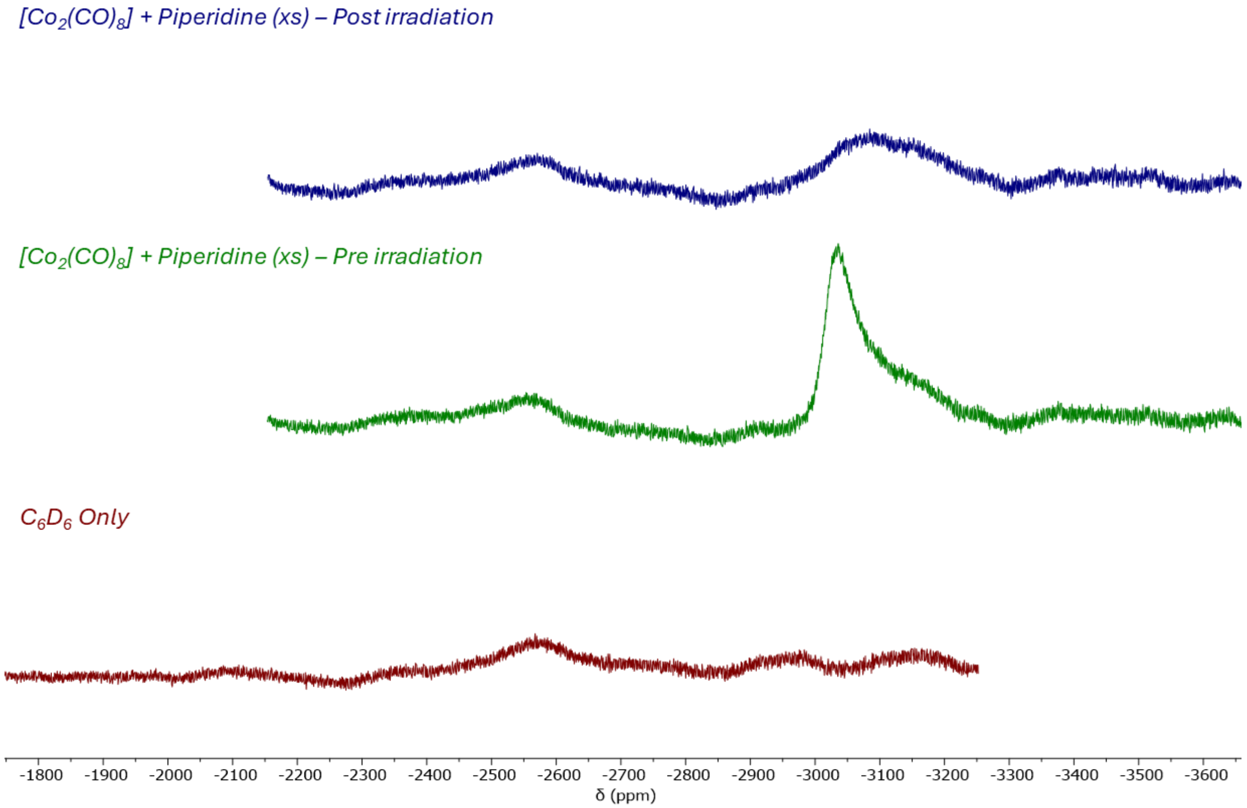

Figure S53: ^{59}Co NMR analysis of [$Co_2(CO)_8$] & piperidine (xs), pre and post irradiation (365 nm LED, 10 W)

^{59}Co NMR Analysis Reaction Spectra – [$Co_2(CO)_8$] + DBU (1:1)

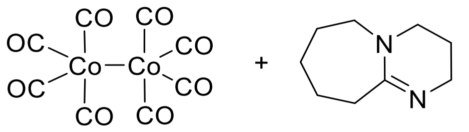

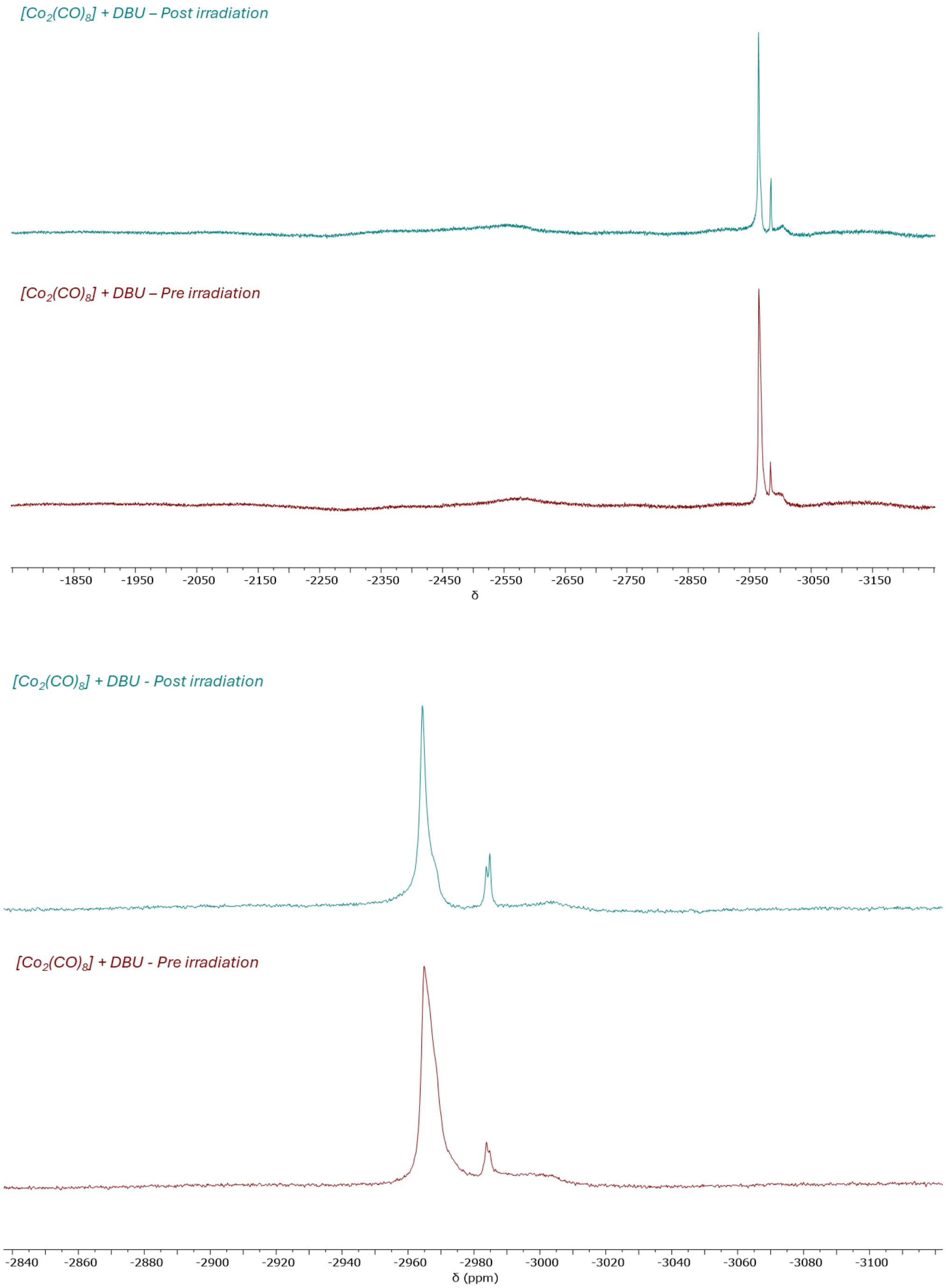

Figure S54: ^{59}Co NMR analysis of [$Co_2(CO)_8$] & DBU, pre and post irradiation (365 nm LED, 10 W). Bottom spectra zoomed for clarity

^{59}Co NMR Analysis Reaction Spectra – [$Co_2(CO)_8$] + DBU (1:20)

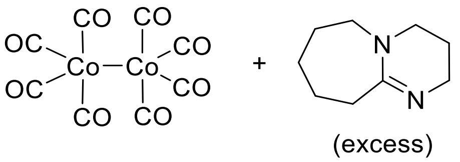

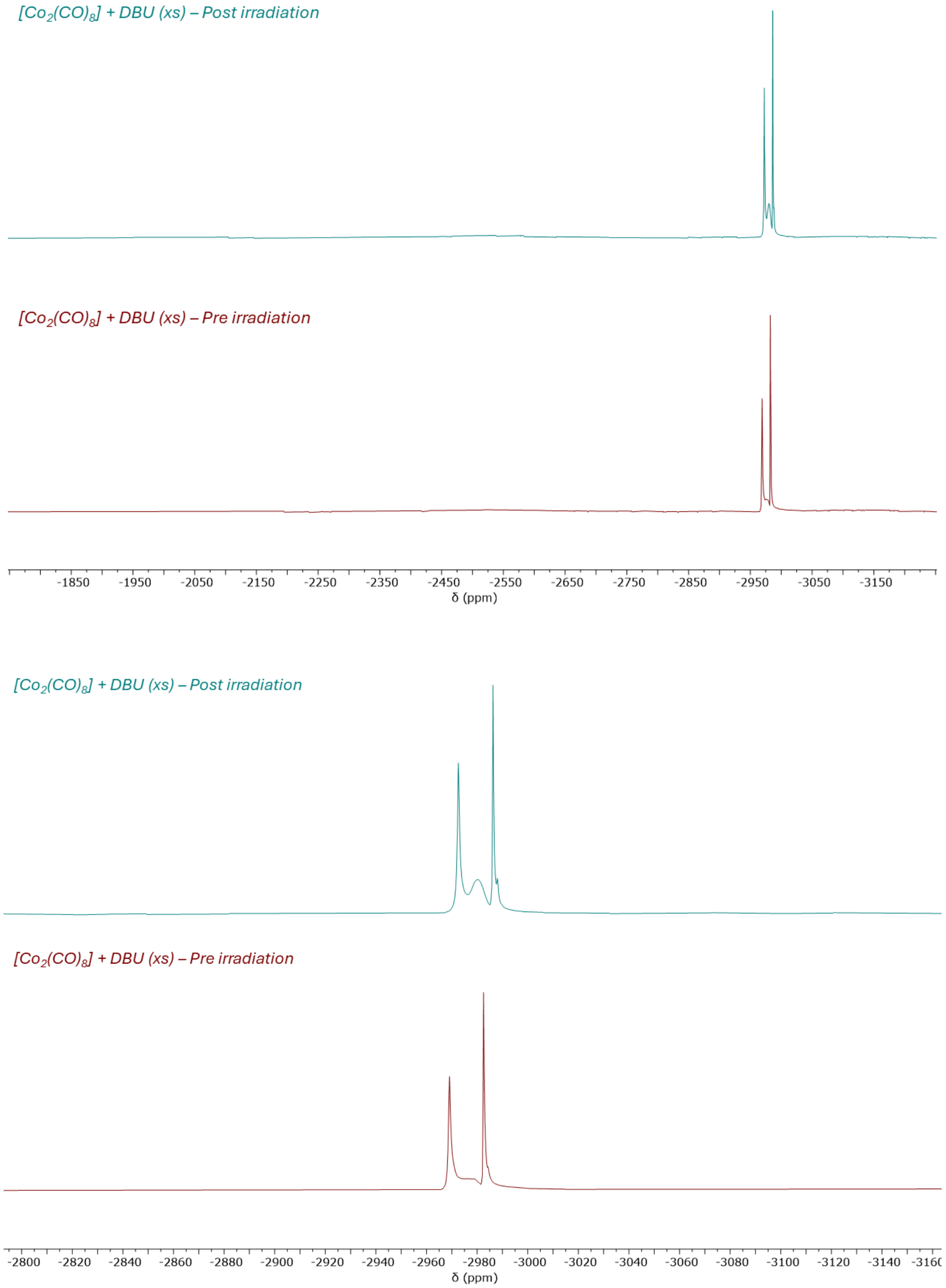

Figure S55: ^{59}Co NMR analysis of [$Co_2(CO)_8$] & DBU (xs), pre and post irradiation (365 nm LED, 10 W). Bottom spectra zoomed for clarity

^{59}Co NMR Analysis Reaction Spectra – $[Co_2(CO)_8]$ + ArBr (1:1)

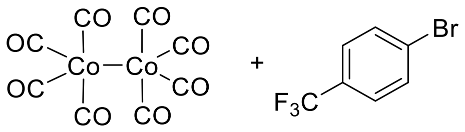

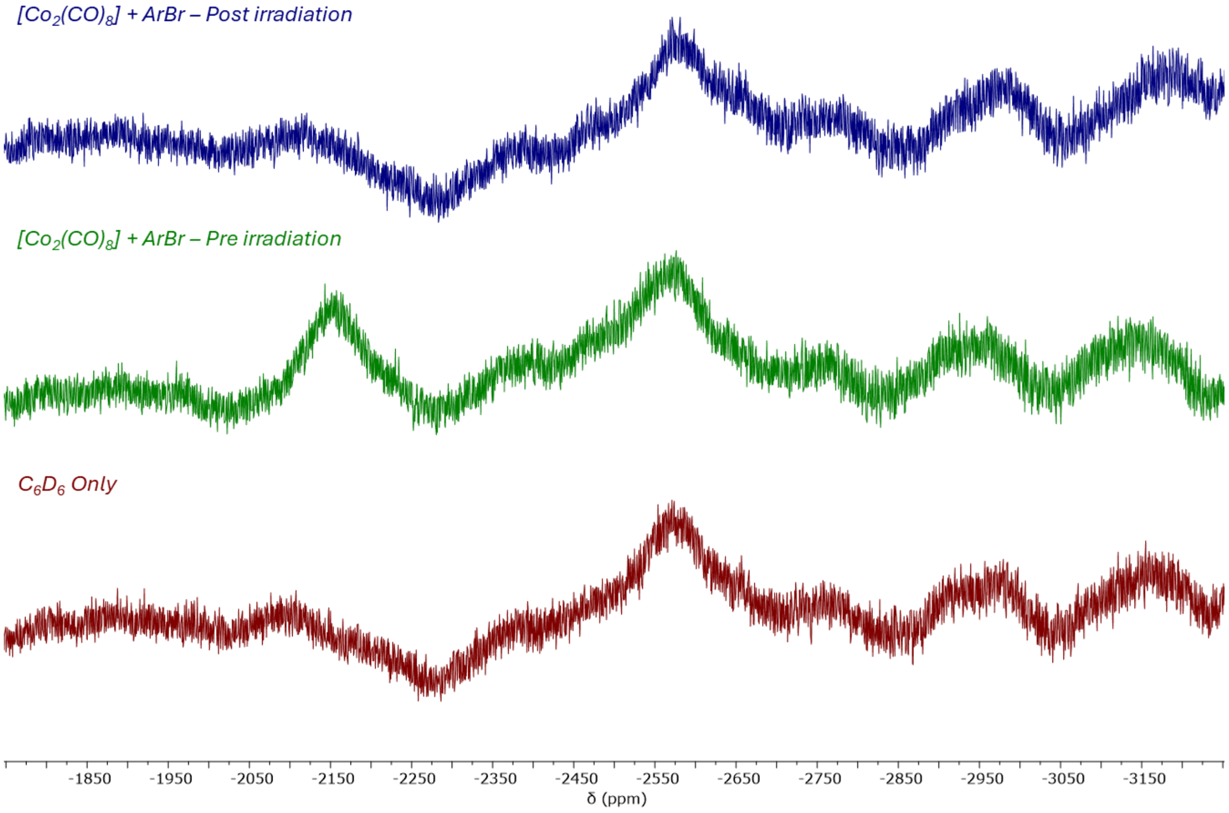

Figure S56: ^{59}Co NMR analysis of $[Co_2(CO)_8]$ & ArBr (CF_3), pre and post irradiation (365 nm LED, 10 W)

^{59}Co NMR Analysis Reaction Spectra – $[Co_2(CO)_8]$ + TMP (1:20)

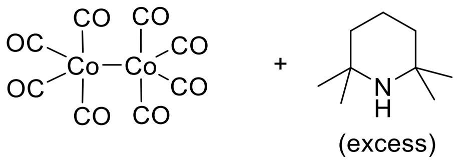

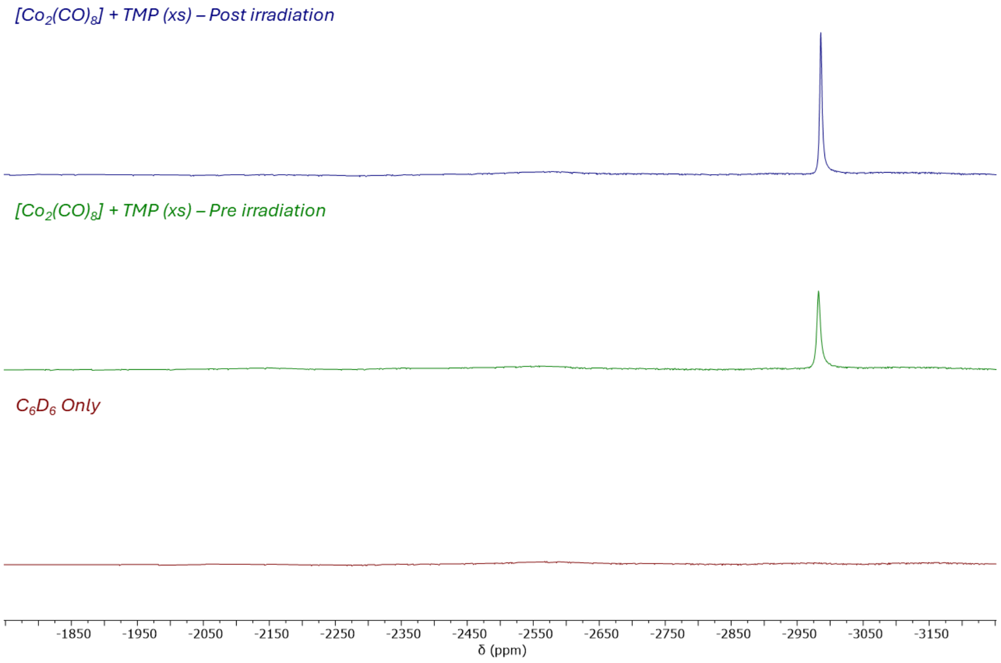

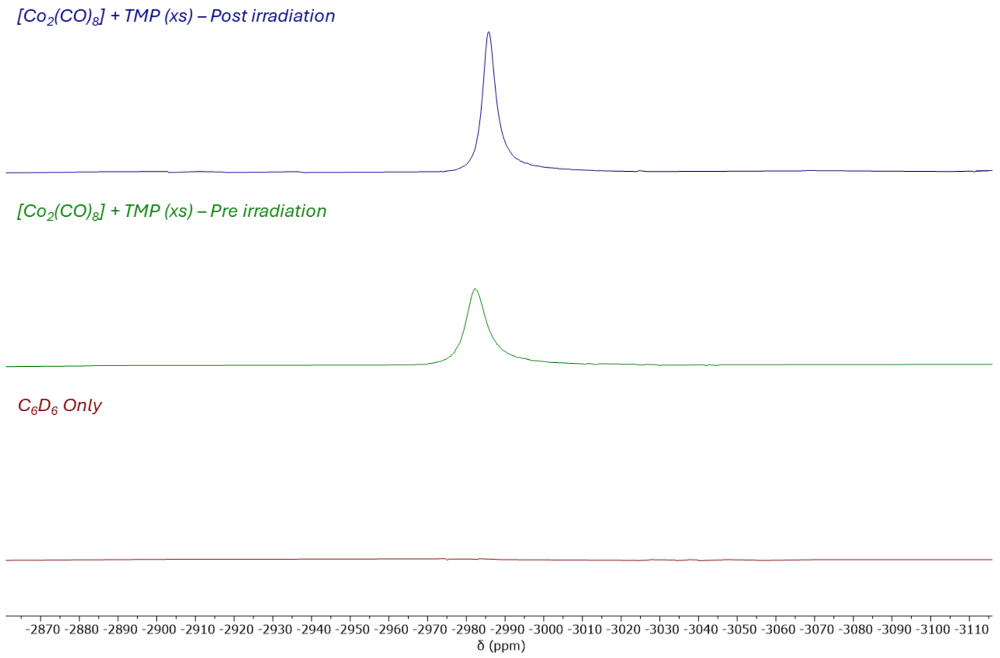

Figure S57: ^{59}Co NMR analysis of $[Co_2(CO)_8]$ & TMP, pre and post irradiation (365 nm LED, 10 W)

^{59}Co NMR Analysis Reaction Spectra – [$Co_2(CO)_8$] + ArBr + Piperidine (1:1:1)

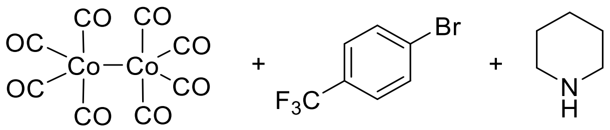

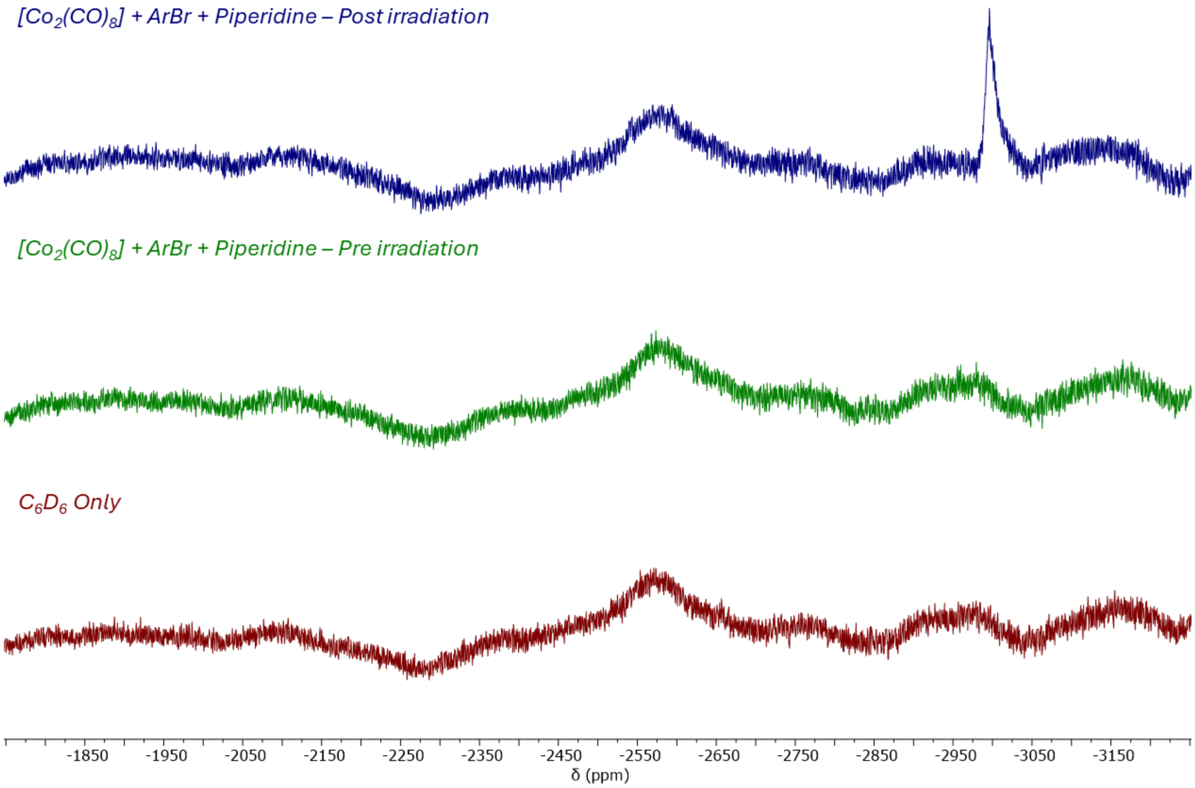

Figure S58: ^{59}Co NMR analysis of [$Co_2(CO)_8$] & ArBr (CF_3) & piperidine, pre and post irradiation (365 nm LED, 10 W)

^{59}Co NMR Analysis Reaction Spectra – [$Co_2(CO)_8$] + ArBr + Piperidine (1:20:20)

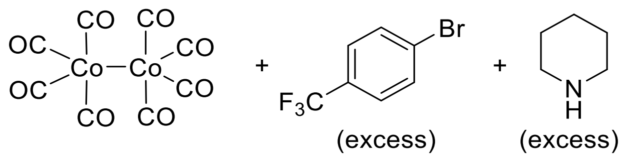

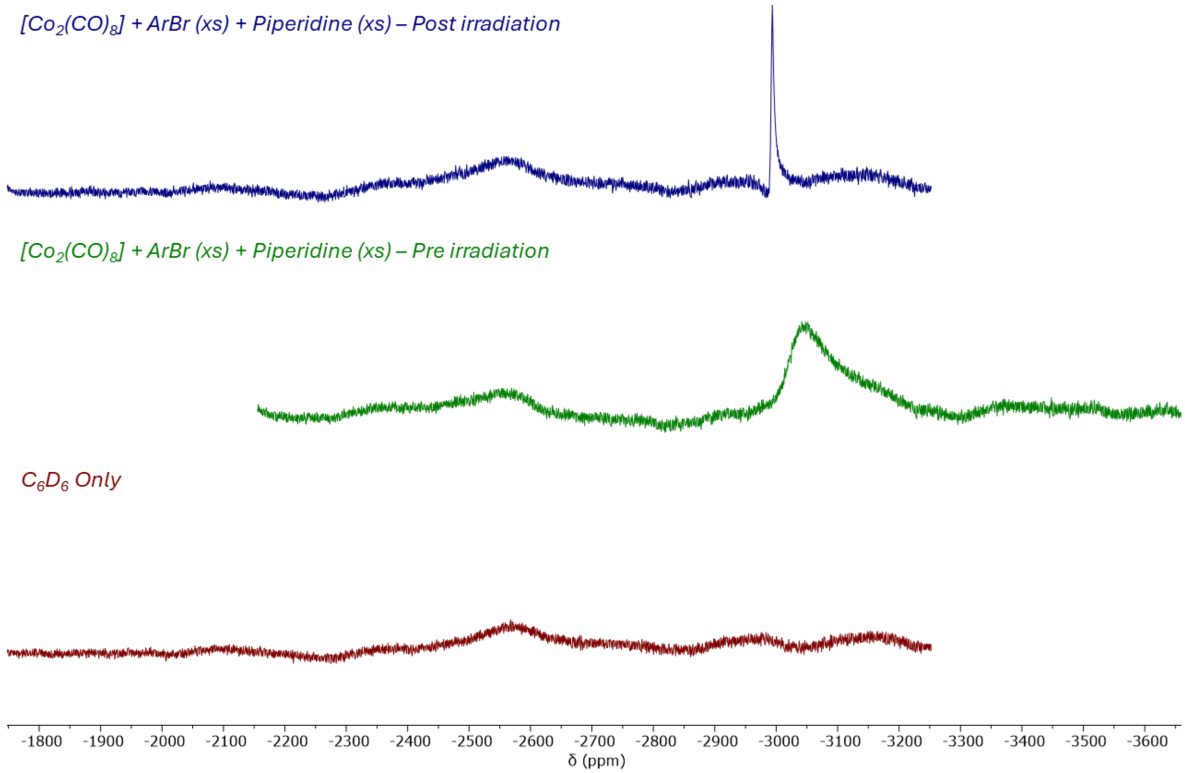

Figure S59: ^{59}Co NMR analysis of [$Co_2(CO)_8$] & ArBr (CF_3) (xs) & piperidine (xs), pre and post irradiation (365 nm LED, 10 W)

^{59}Co NMR Analysis Reaction Spectra – $[Co_2(CO)_8]$ + ArBr + DBU (1:1:1)

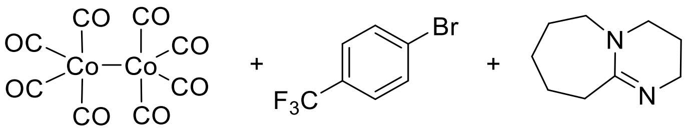

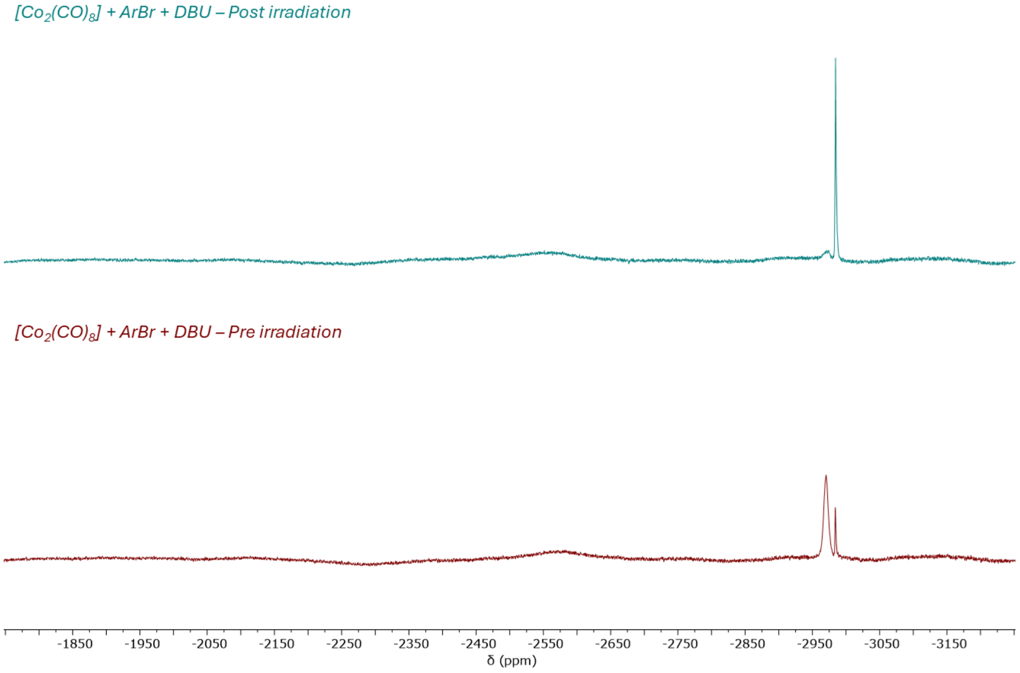

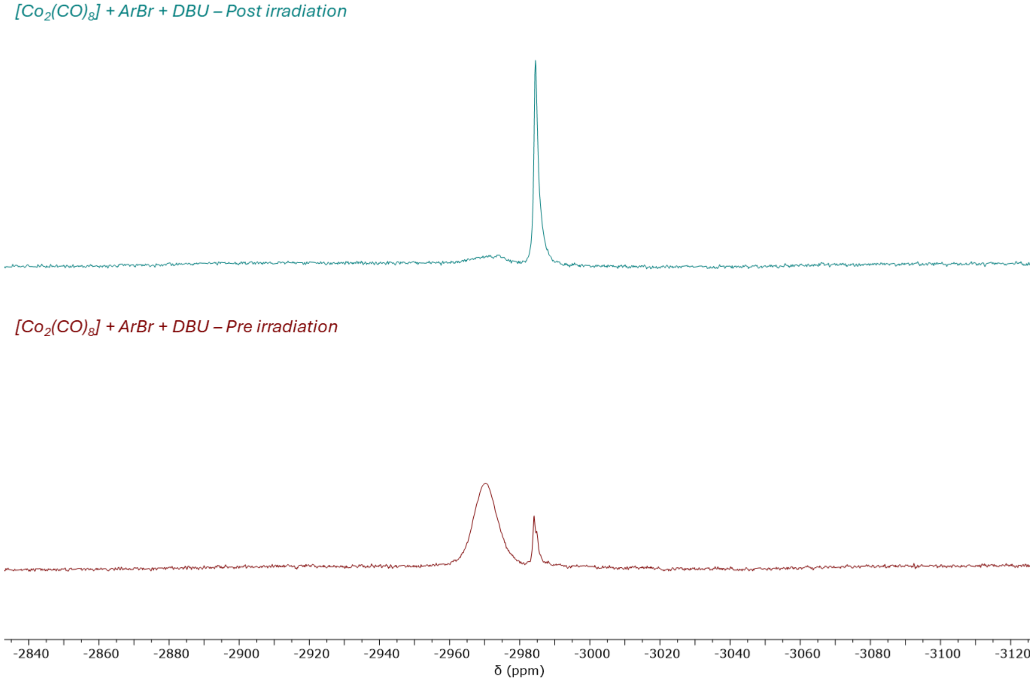

Figure S60: ^{59}Co NMR analysis of $[Co_2(CO)_8]$ & ArBr (CF_3) & DBU, pre and post irradiation (365 nm LED, 10 W)

^{59}Co NMR Analysis Reaction Spectra – [$Co_2(CO)_8$] + Piperidine + DBU (1:1:1)

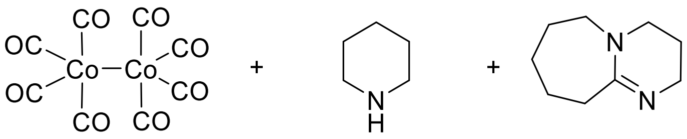

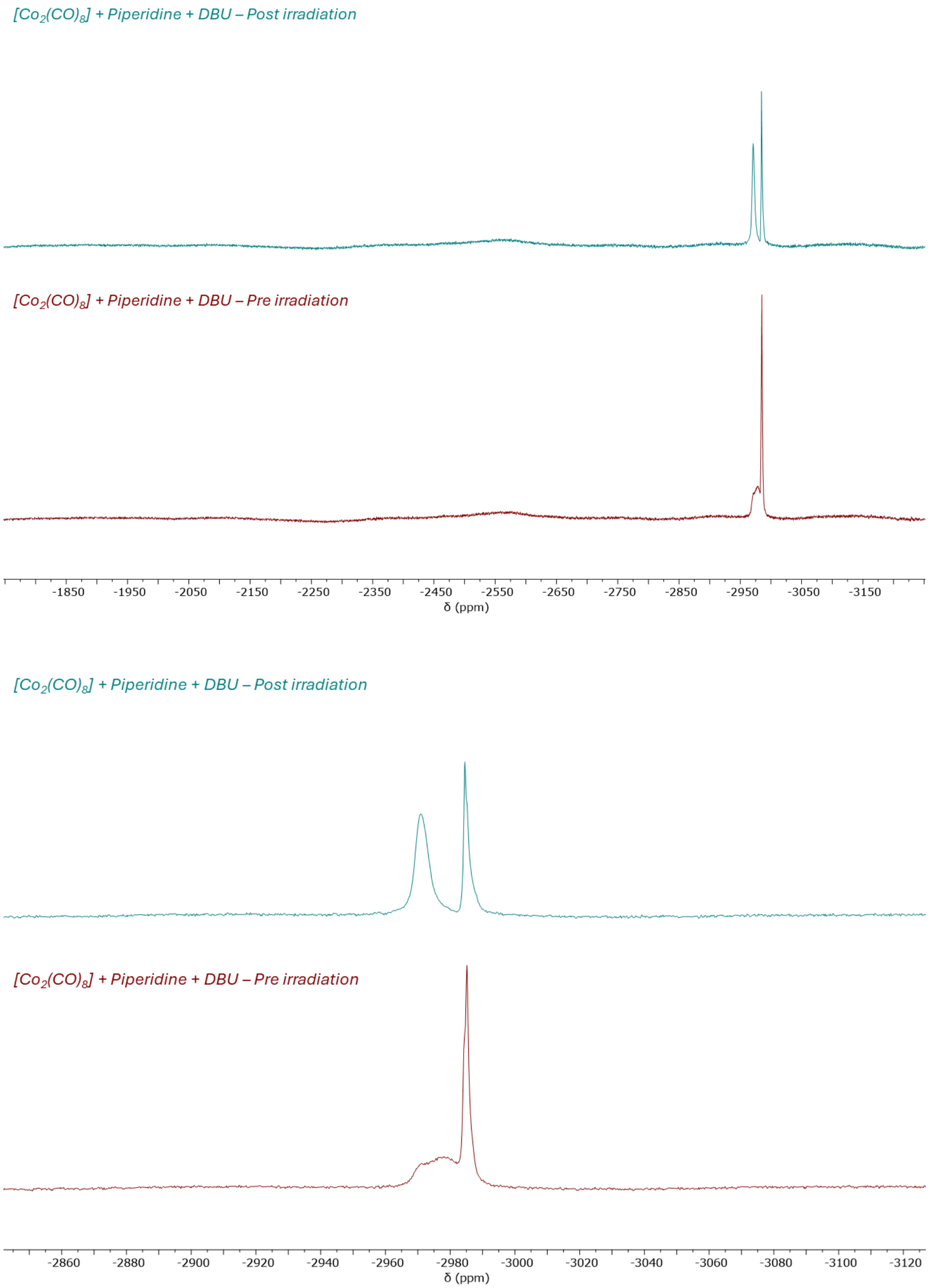

Figure S61: ^{59}Co NMR analysis of [$Co_2(CO)_8$] & piperidine & DBU, pre and post irradiation (365 nm LED, 10 W)

^{59}Co NMR Analysis Reaction Spectra – $[Co_2(CO)_8]$ + ArBr + Piperidine + DBU (1:1:1:1)

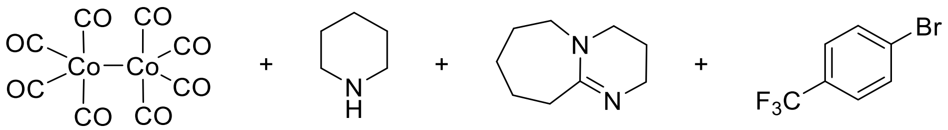

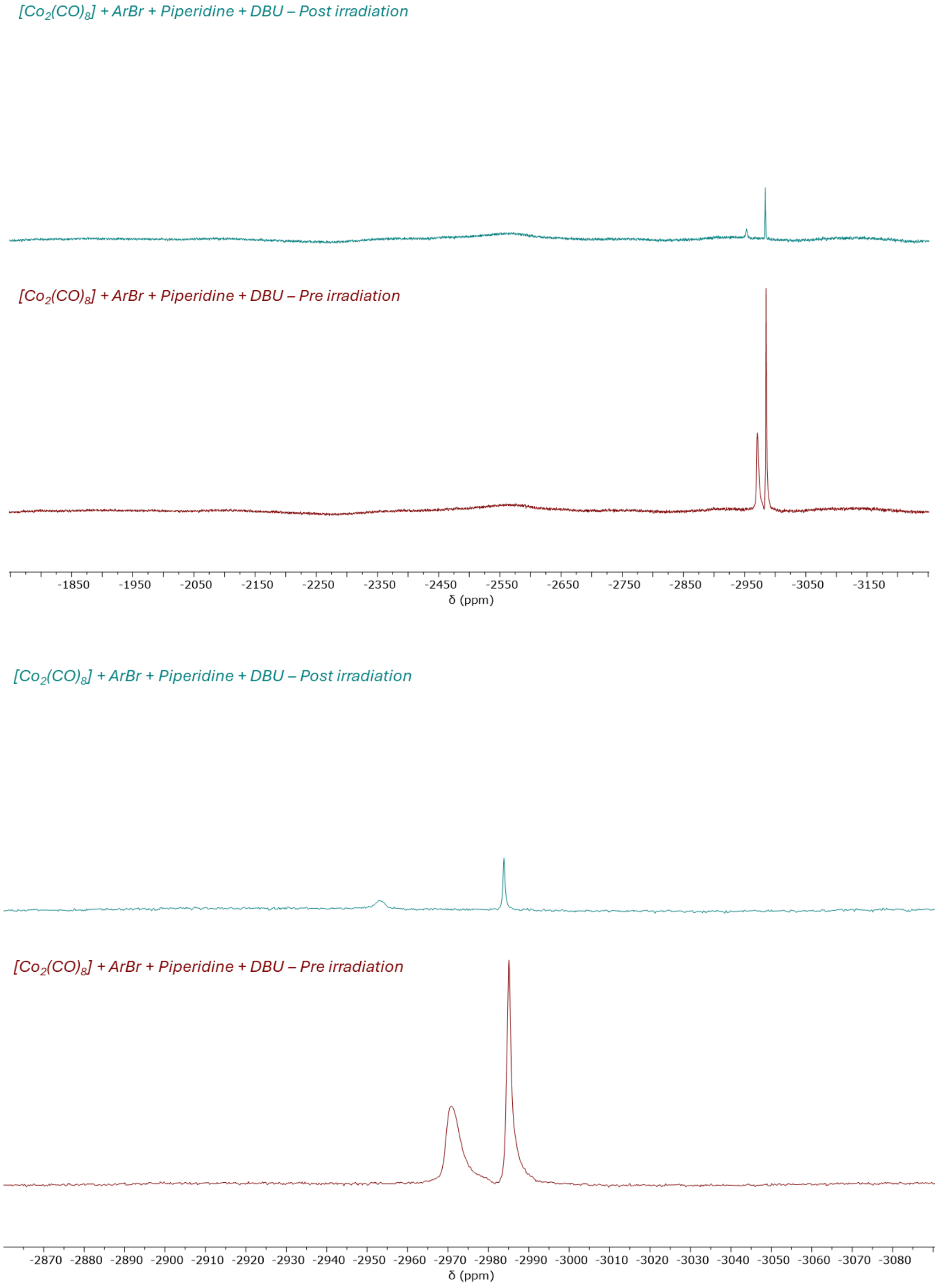

Figure S62: ^{59}Co NMR analysis of $[Co_2(CO)_8]$ & ArBr (CF_3) & piperidine & DBU, pre and post irradiation (365 nm LED, 10 W)

^{59}Co NMR Analysis Reaction Spectra – K[Co(CO)$_4$] + Piperidine (xs)

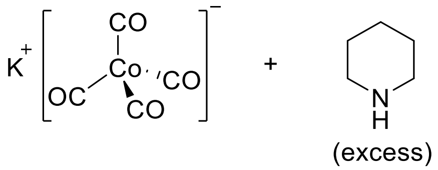

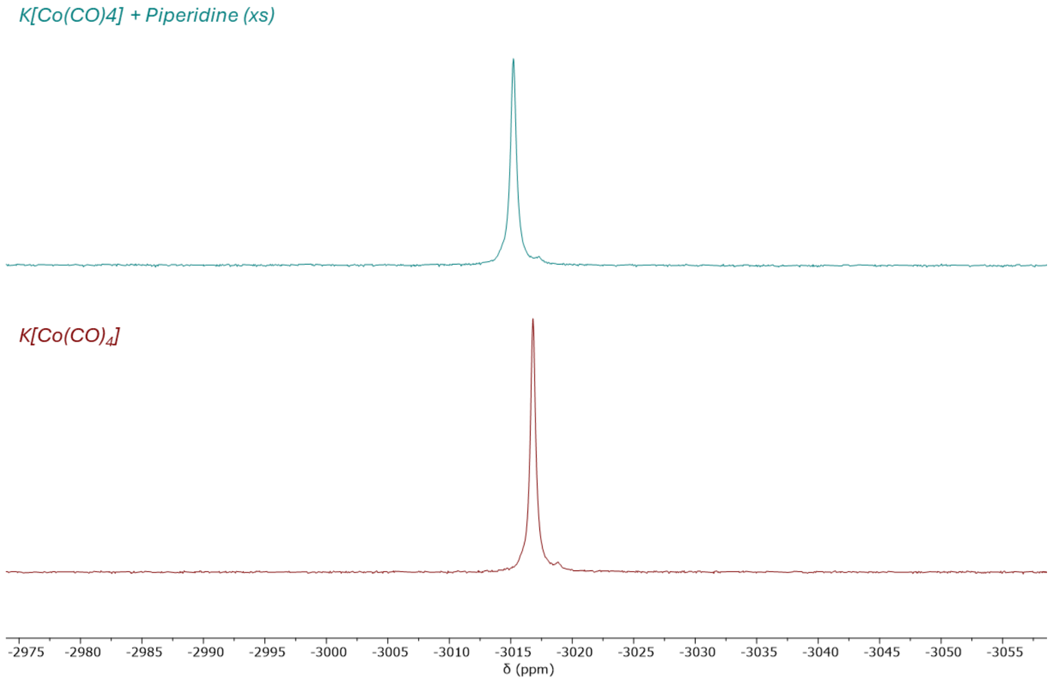

Figure S63: ^{59}Co NMR analysis of K[Co(CO)$_4$] & piperidine. Overnight irradiation (365 nm LED, 10 W) of samples caused no observable change to spectra

^{59}Co NMR Analysis Reaction Spectra – K[Co(CO)$_4$] + Piperidine (xs)

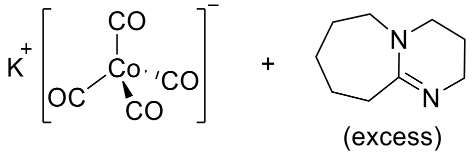

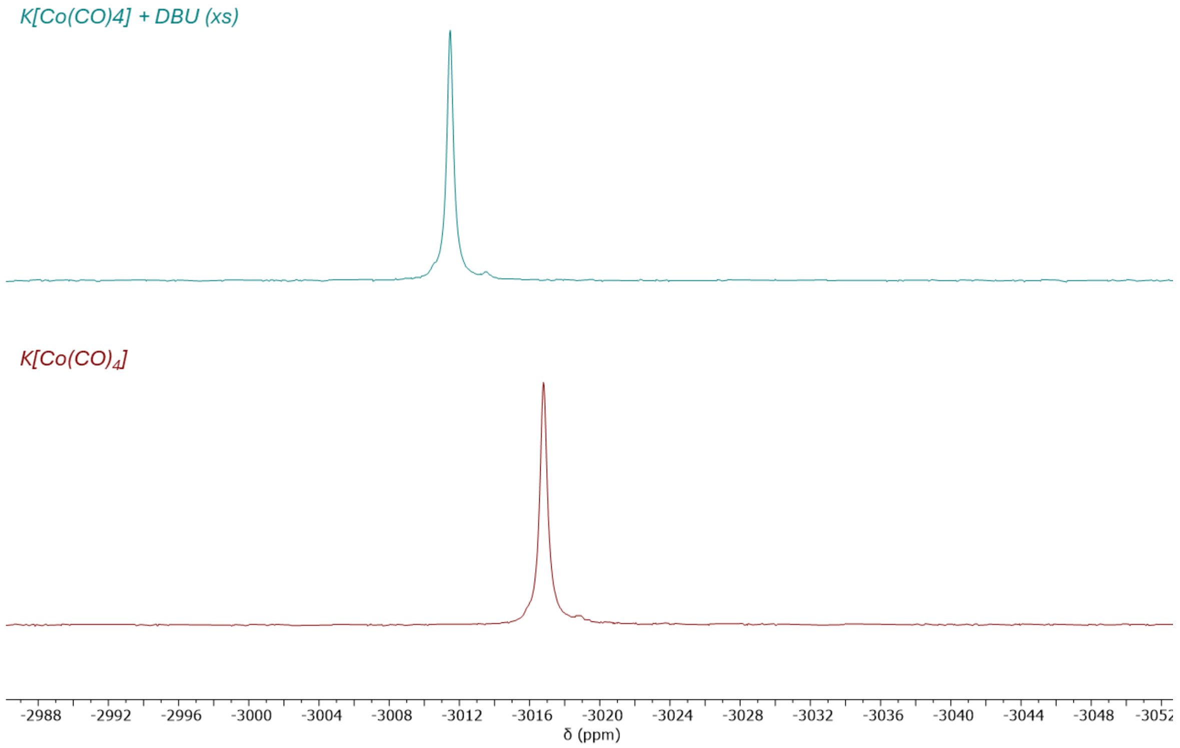

Figure S64: ^{59}Co NMR analysis of K[Co(CO)$_4$] & DBU. Overnight irradiation (365 nm LED, 10 W) of samples caused no observable change to spectra

^{59}Co NMR Analysis Reaction Spectra – K[Co(CO)$_4$] + ArBr (xs)

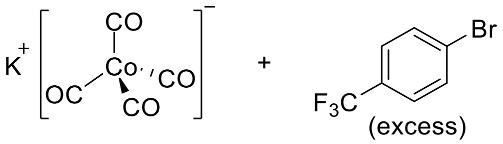

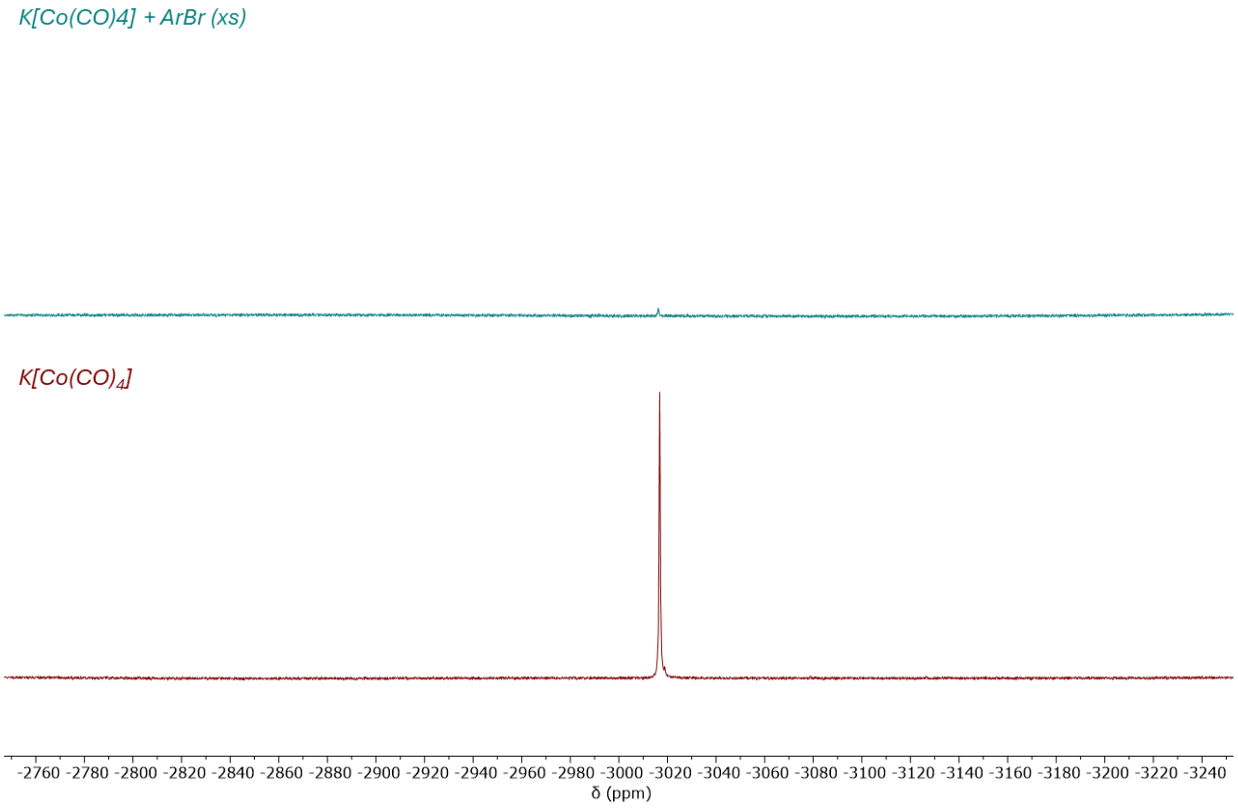

Figure S65: ^{59}Co NMR analysis of K[Co(CO)$_4$] & ArBr (CF$_3$)

IR Data

IR spectra of **$Co_2(CO)_8$**

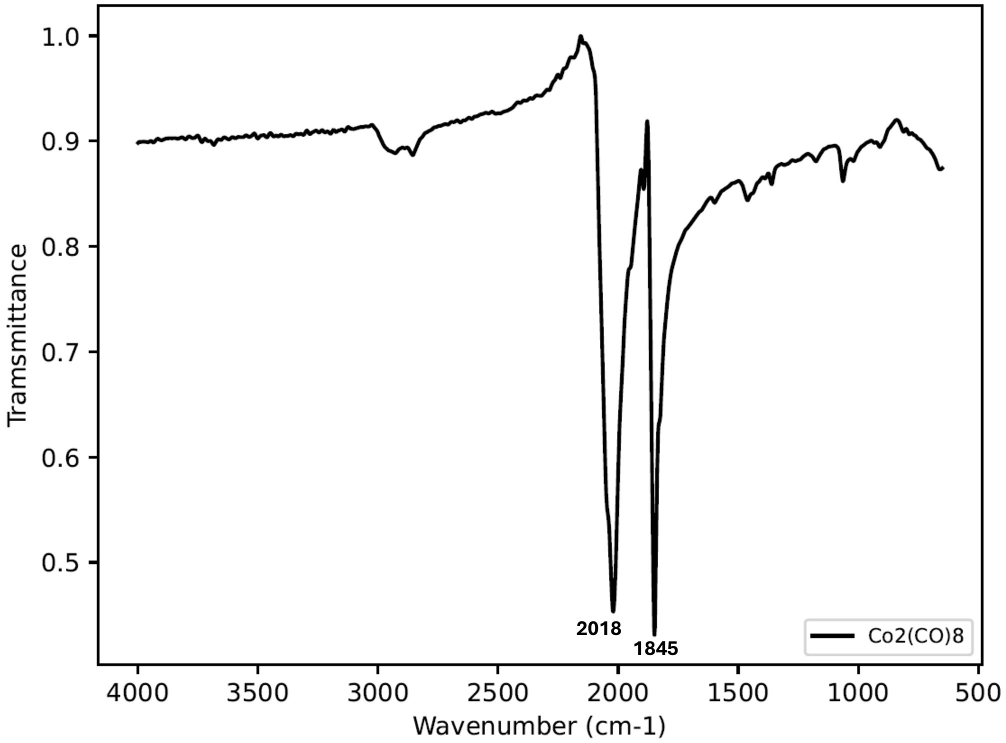

IR spectra of **4-(trifluoromethyl)-bromobenzene**

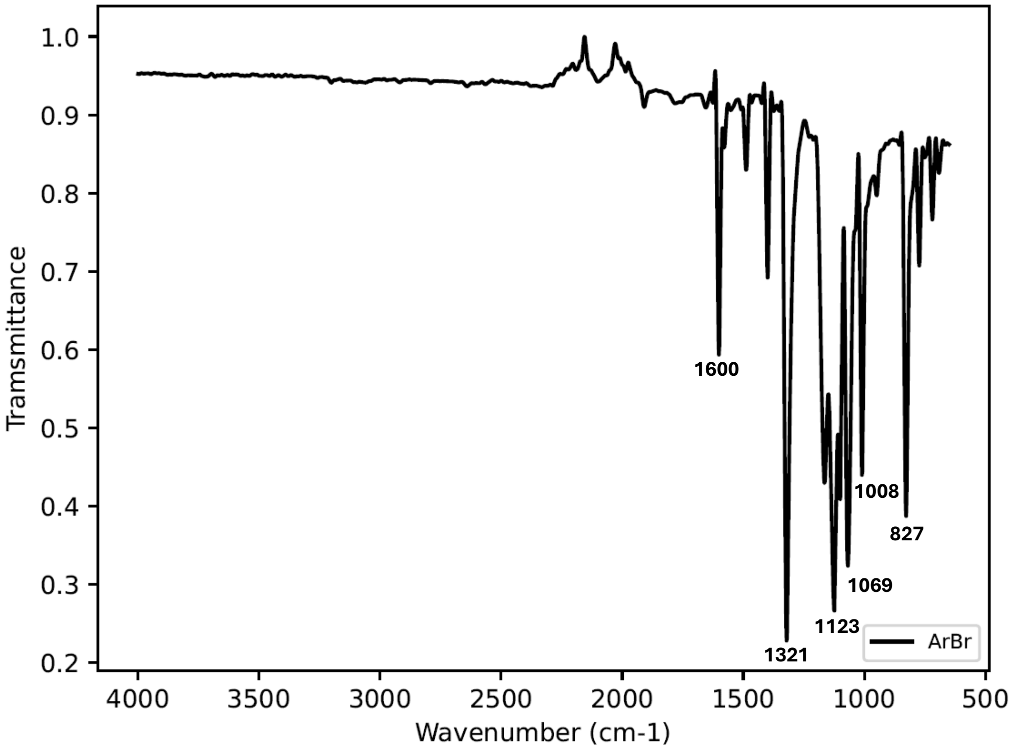

IR spectra of **Piperidine**

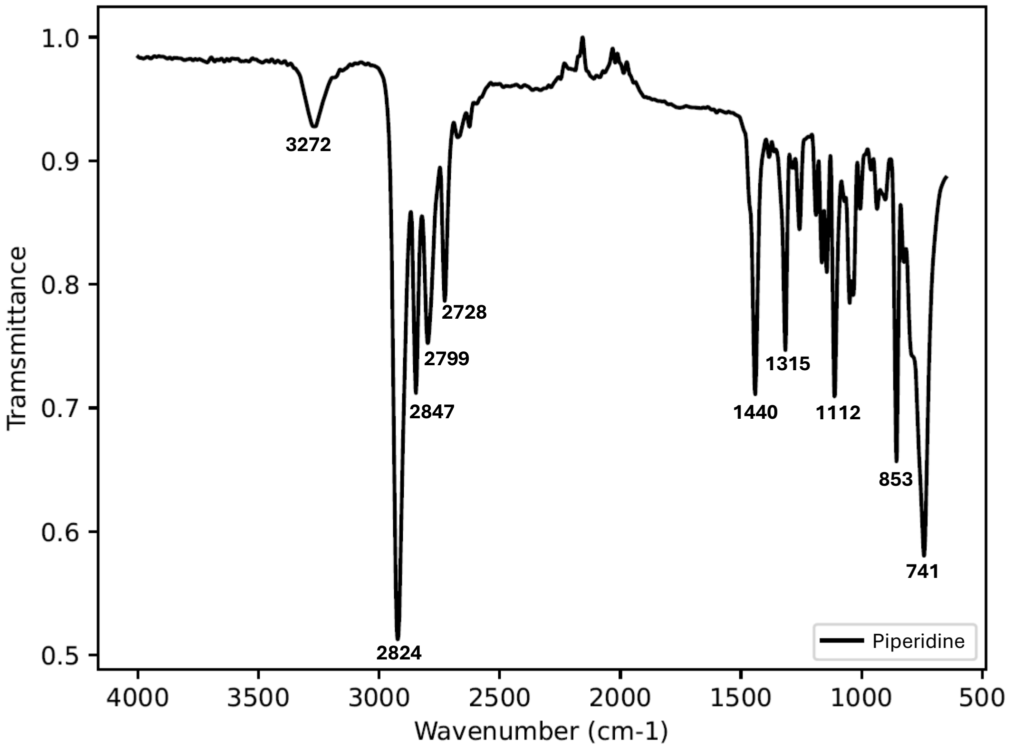

IR spectra of **DBU**

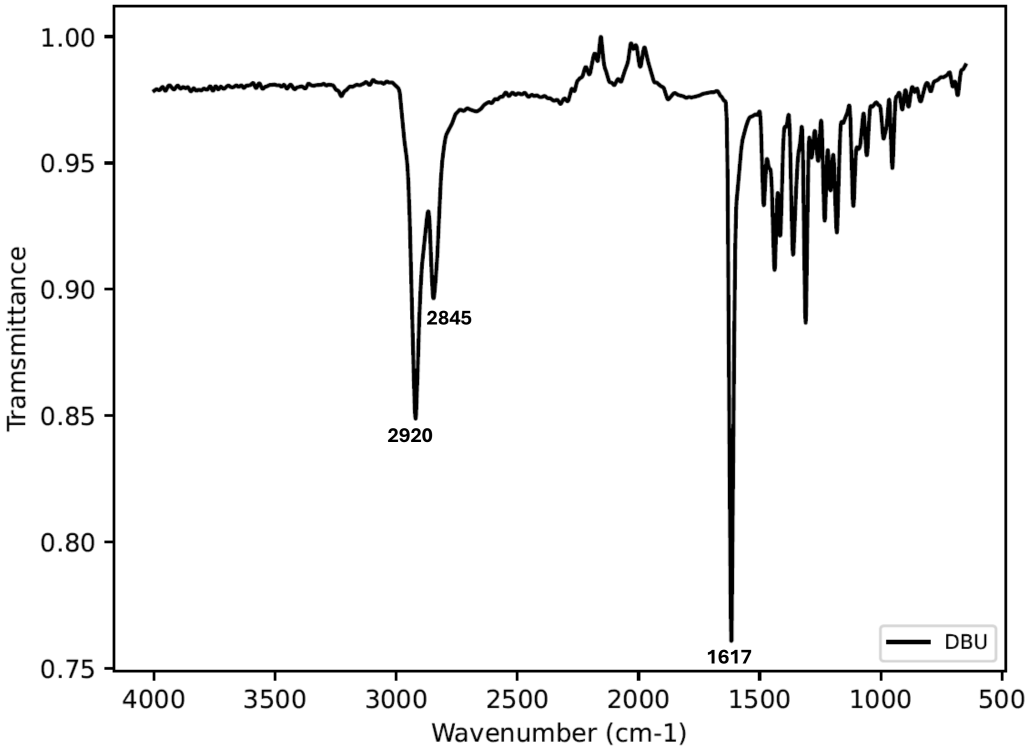

IR comparison spectra of **$Co_2(CO)_8$** & **ArBr**

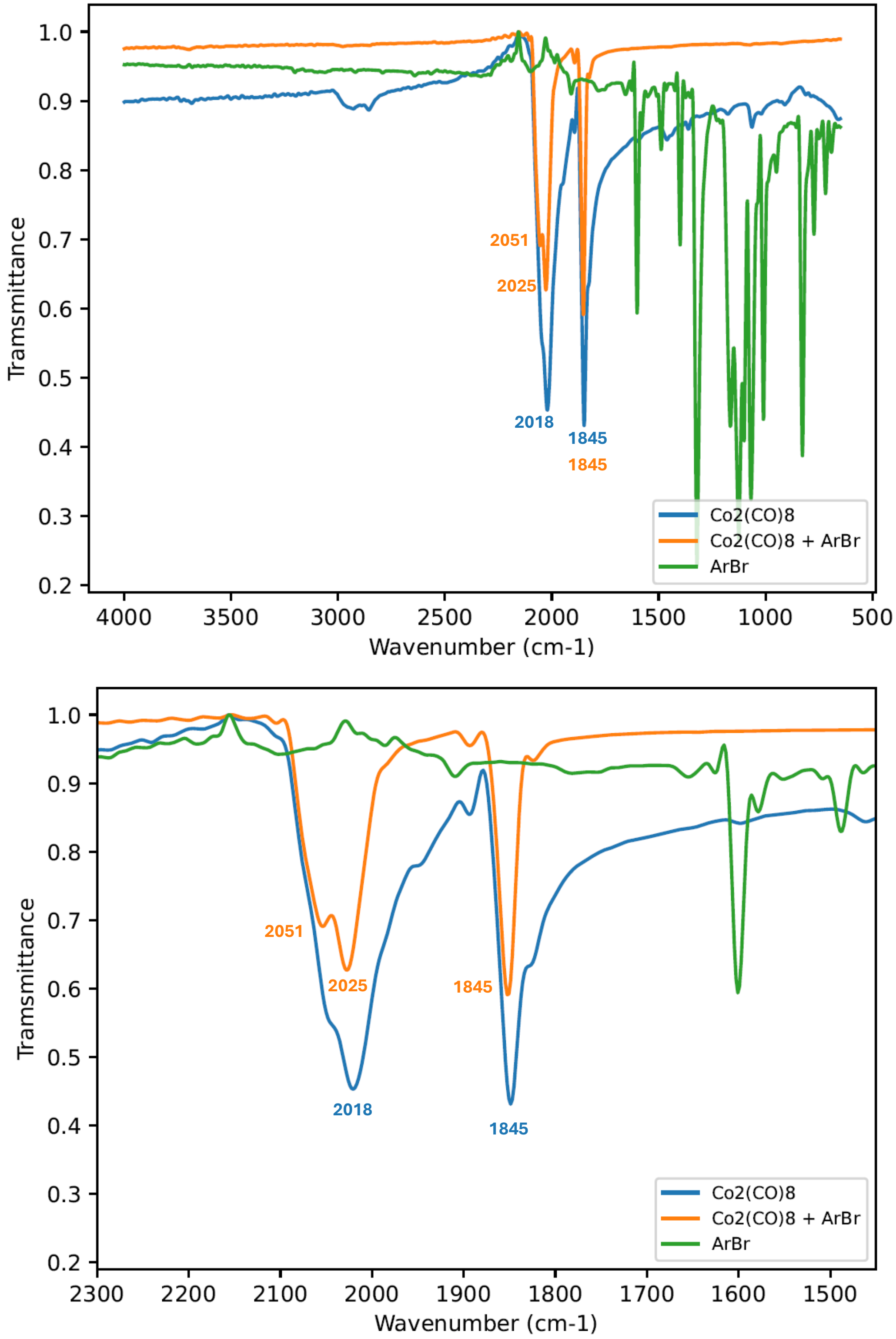

Figure S66: IR analysis of [$Co_2(CO)_8$] & ArBr (CF_3). Zoomed spectra is shown (bottom)

IR comparison spectra of **$Co_2(CO)_8$** & **Piperidine**

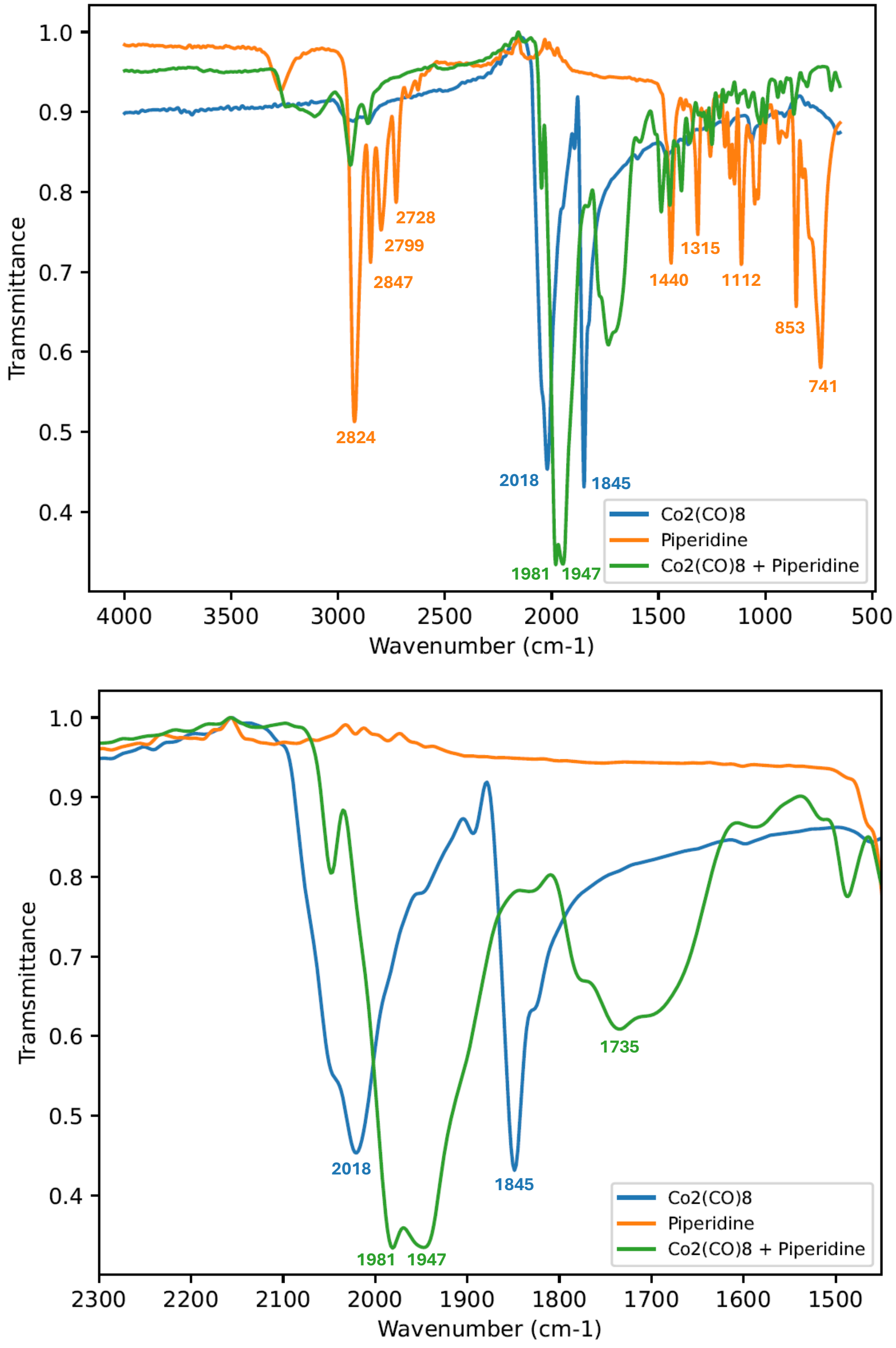

Figure S67: IR analysis of [$Co_2(CO)_8$] & Piperidine. Zoomed spectra is shown (bottom)

IR comparison spectra of **$Co_2(CO)_8$** & **DBU**

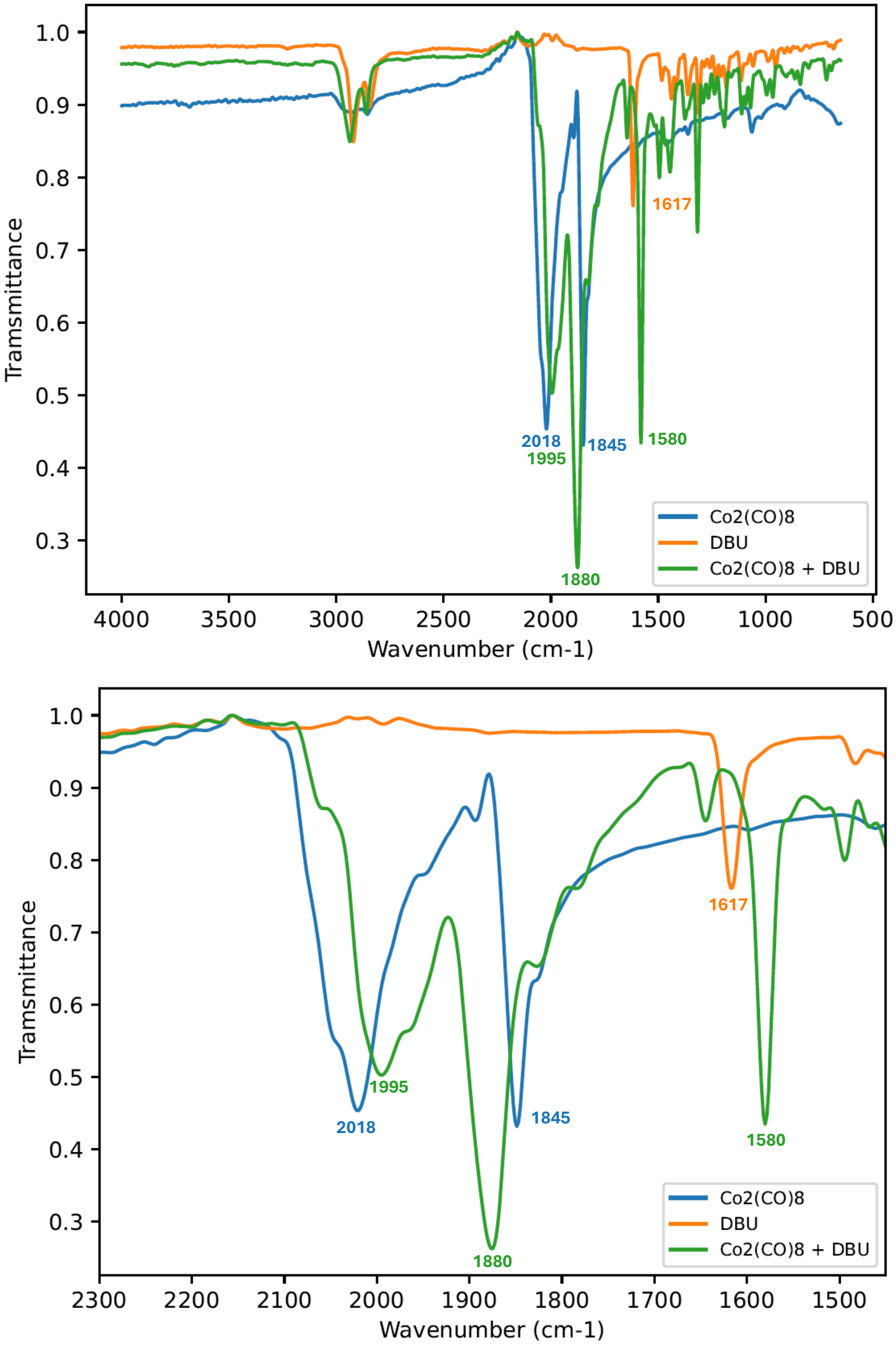

Figure S68: IR analysis of [$Co_2(CO)_8$] & DBU. Zoomed spectra is shown (bottom)

IR comparison spectra of **$Co_2(CO)_8$** & **ArBr** & **DBU**

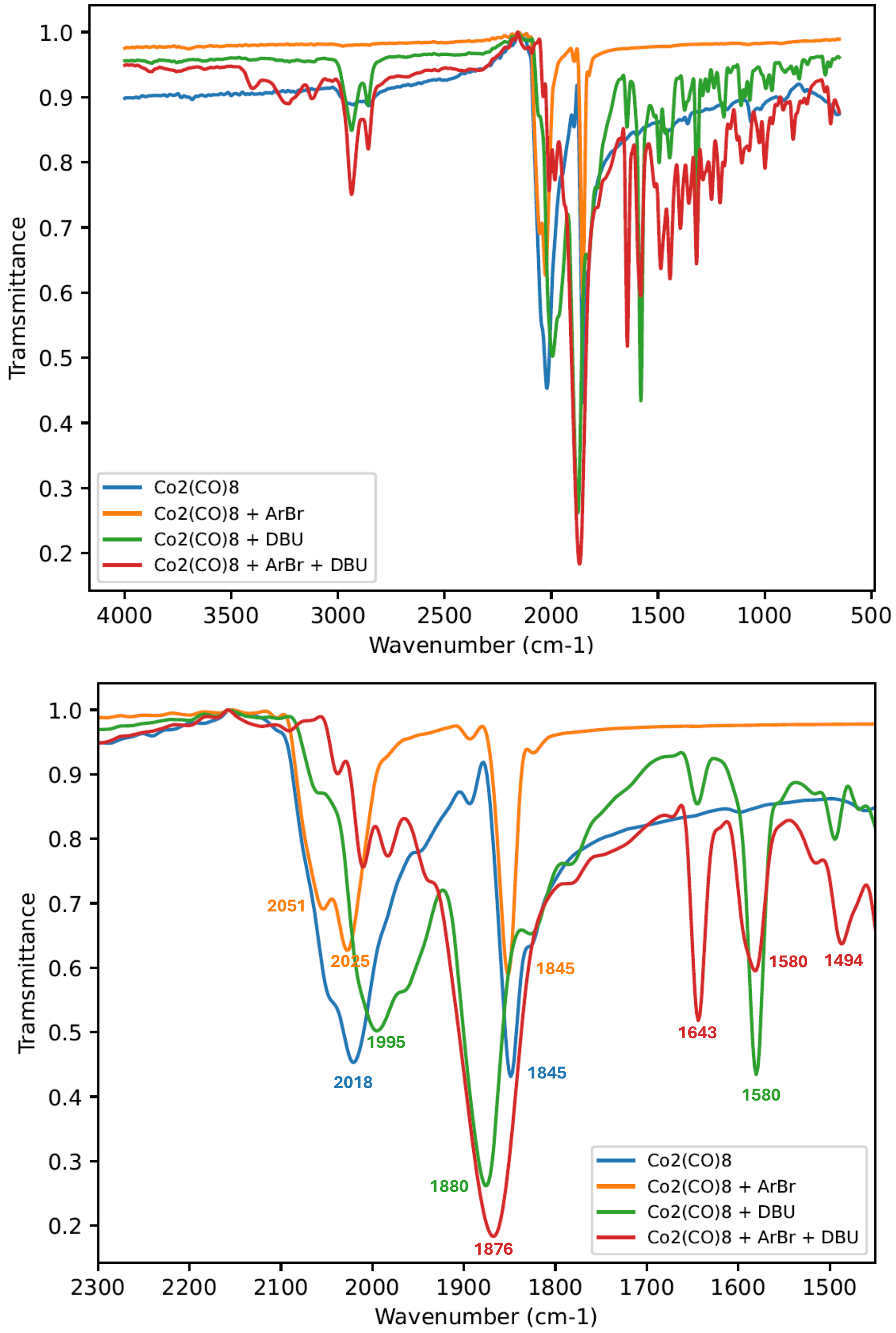

Figure S69: IR analysis of [$Co_2(CO)_8$] & ArBr (CF_3) & DBU. Zoomed spectra is shown (bottom)

IR comparison spectra of **$Co_2(CO)_8$** & **Piperidine** & **DBU**

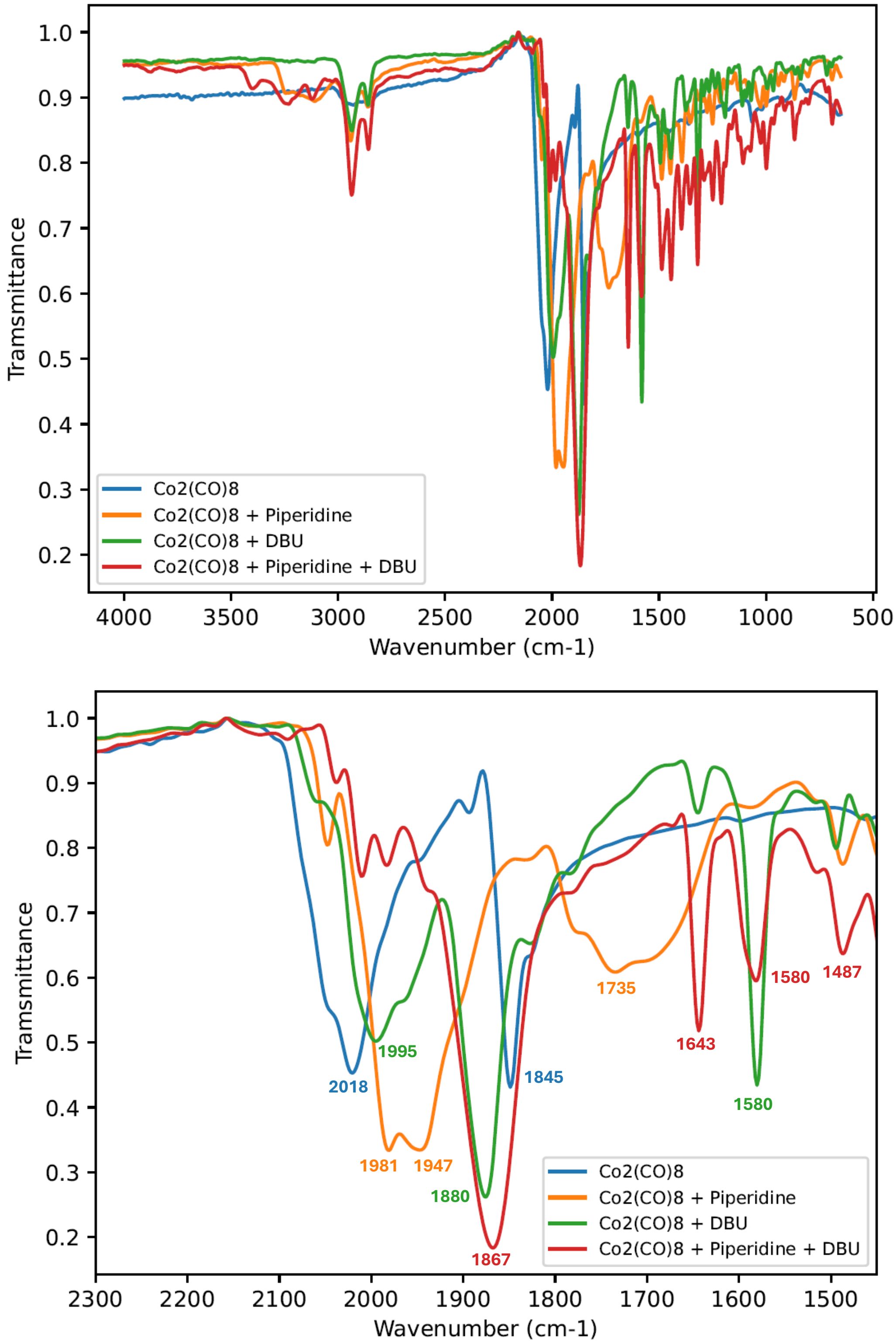

Figure S70: IR analysis of [$Co_2(CO)_8$] & Piperidine & DBU. Zoomed spectra is shown (bottom)

IR comparison spectra of **$Co_2(CO)_8$** & **ArBr** & **Piperidine**

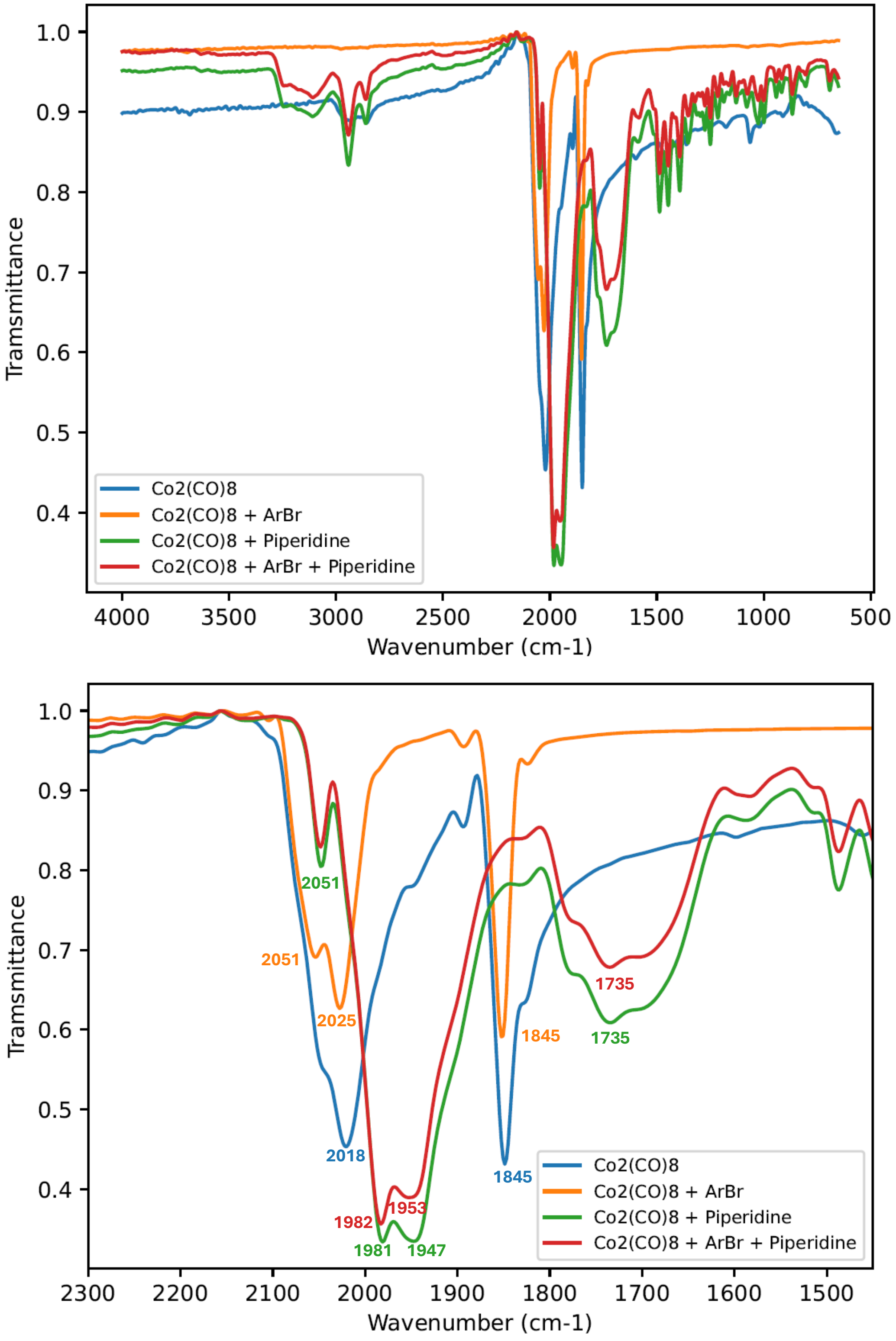

Figure S71: IR analysis of [$Co_2(CO)_8$] & ArBr (CF_3) & Piperidine. Zoomed spectra is shown (bottom)

IR comparison spectra of **Mixed System Components**

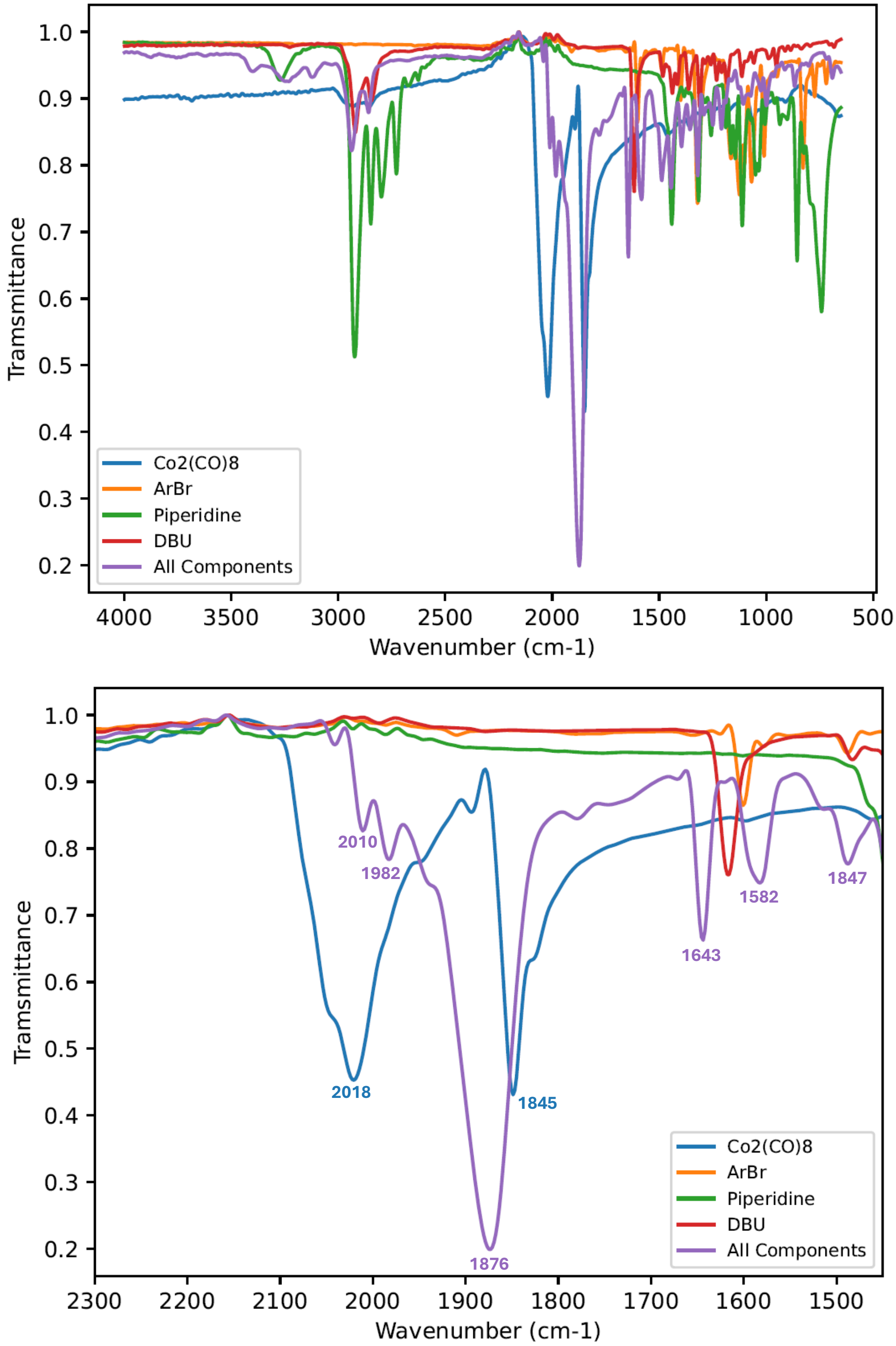

Figure S72: IR analysis of fully mixed system versus unique component IR spectra. Zoomed spectra is shown (bottom)

IR comparison spectra of **Relevant System Combinations**

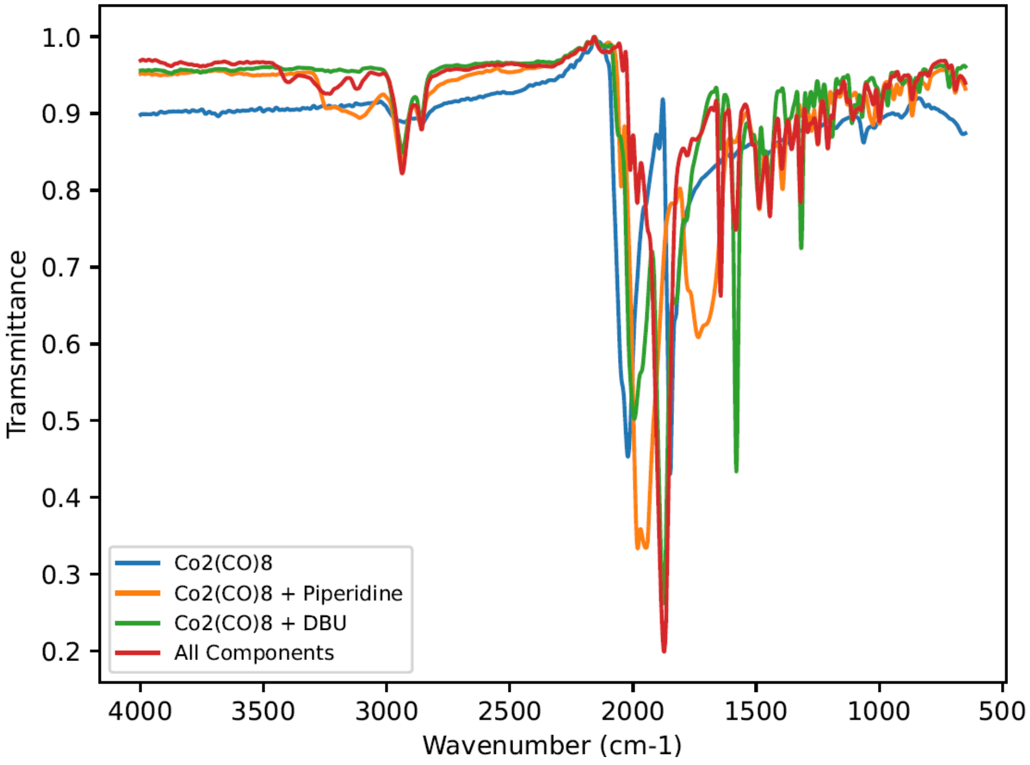

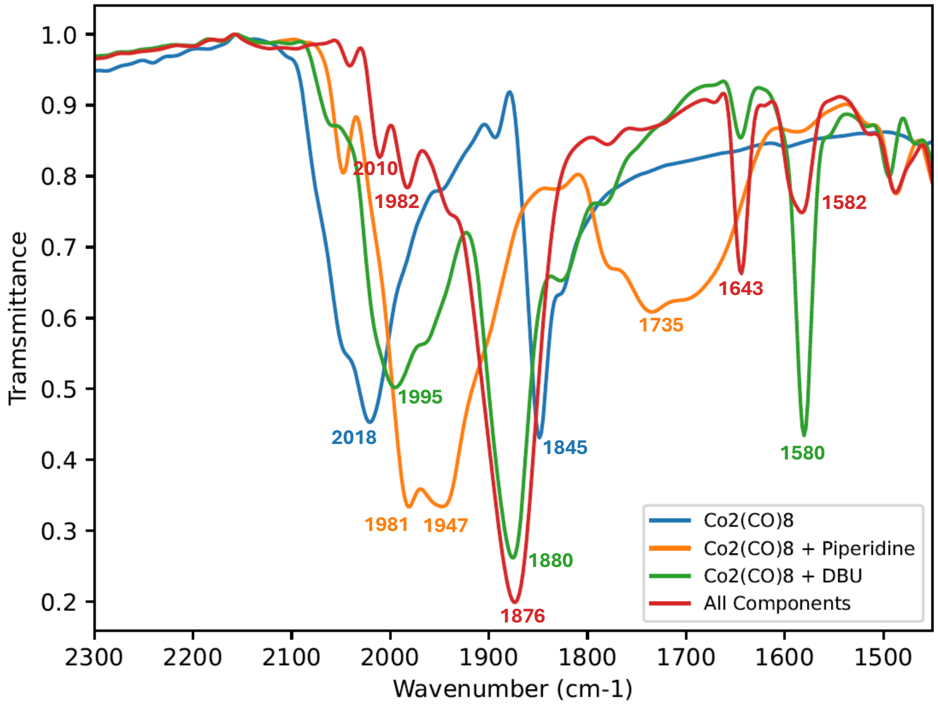

Figure S73: IR analysis of most relevant mixtures versus fully mixed system. Zoomed spectra is shown (bottom)

IR comparison spectra of **$Co_2(CO)_8$** & **K[$Co(CO)_4$]** & **All Components**

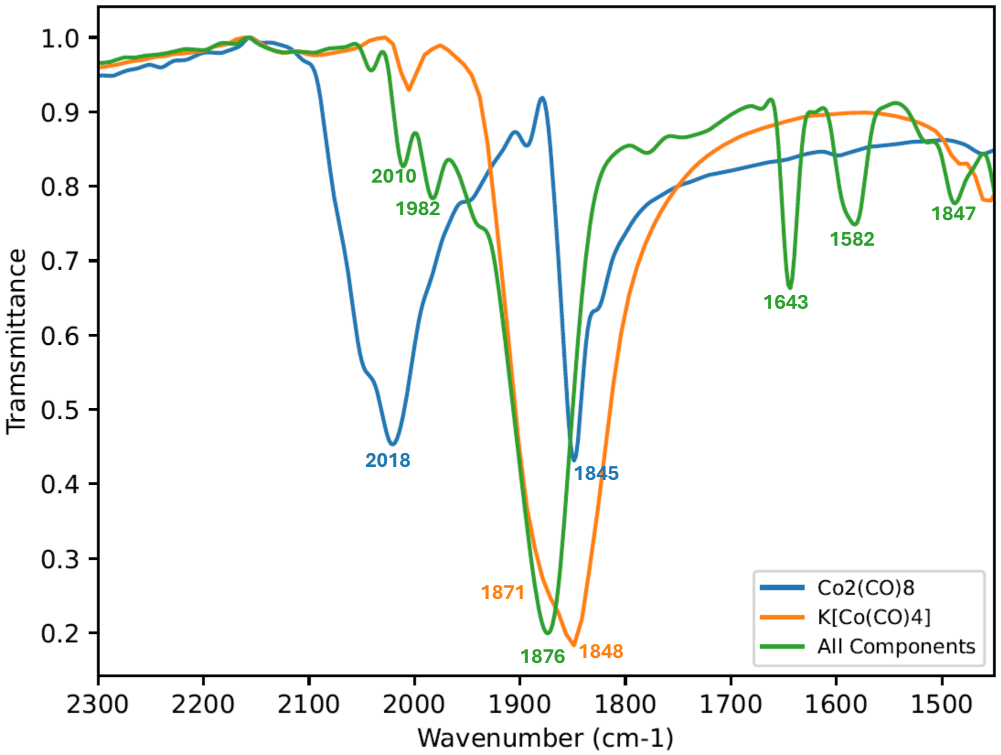

Figure S74: IR analysis of [$Co_2(CO)_8$], K[$Co(CO)_4$] & All components mixture. Zoomed spectra is shown (bottom)

Catalyst Cycle Probing Experiments

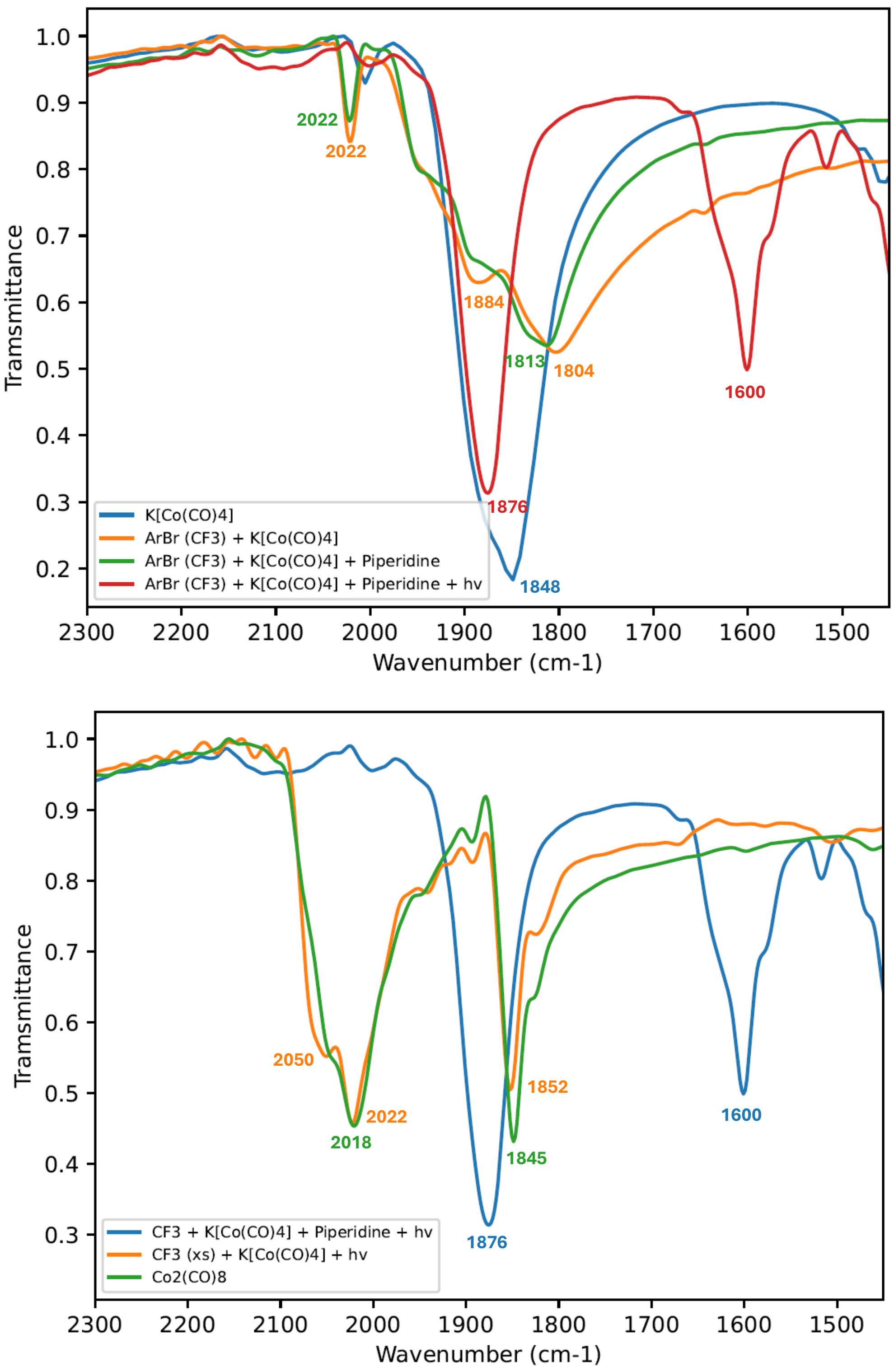

Figure S75: IR analysis of K[Co(CO)$_4$] addition mixtures. The data highlighted that light was necessary for interaction of piperidine, even where the [Co(CO)$_4$] had been activated with ArBr. Irradiation of just K[Co(CO)$_4$] and ArBr resulted in [Co$_2$(CO)$_8$] formation.

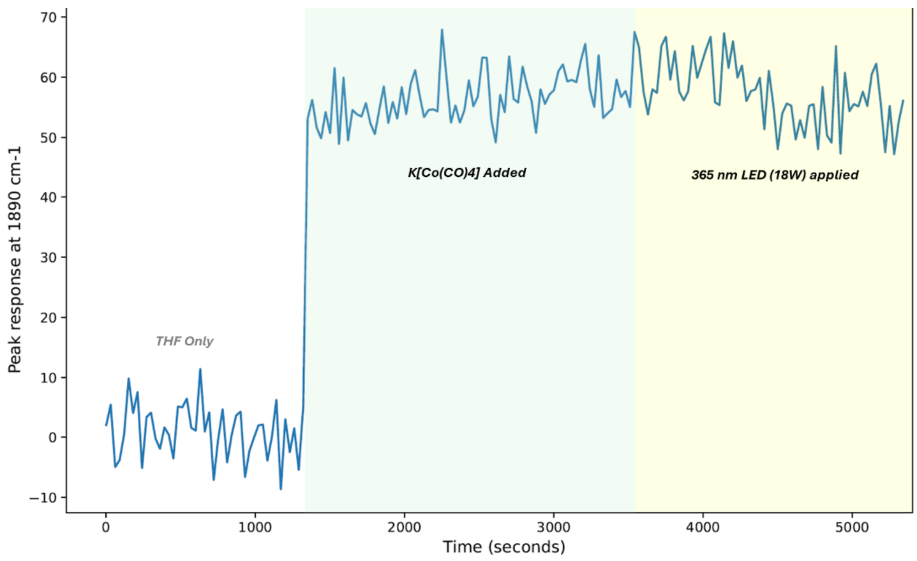

Figure S76: Online IR analysis of K[Co(CO)$_4$] in THF under irradiative conditions. Reaction was continuously flushed with N_2 to irreversibly remove CO from the system if produced. A lack of K[Co(CO)$_4$] consumption suggested that irradiative CO loss was not occurring

Radical Nature Testing

β-bromostyrene was used as a substrate for carbonylation. The unpurified reagent was found to contain a mixture of isomers in a 92:8 ration (E:Z). The reagent was purified according to literature procedure to yield the pure (E) isomer, allowing comparison between mixed and pure substrate mixtures in the forward carbonylation reaction.[5] β-bromostyrene (2.56 mL, 20 mmol) and NaOH (0.68 g, 17 mmol, 0.85 equiv.) were added to a round-bottom flask and dissolved in ^{i}PrOH (20 mL). The reaction mixture was then stirred and heated to reflux and left for 2h. The reaction was left to cool and diluted with H_2O (10 mL) and pentane (10 mL). The organic phase was extracted with water (2 x 10 mL) and subsequently dried with $NaSO_4$. The remaining solvent was removed under reduced pressure. Subsequent distillation (70 °C, 5 mbar) yielded the product as a pale yellow oil (2.37 g ,65%).

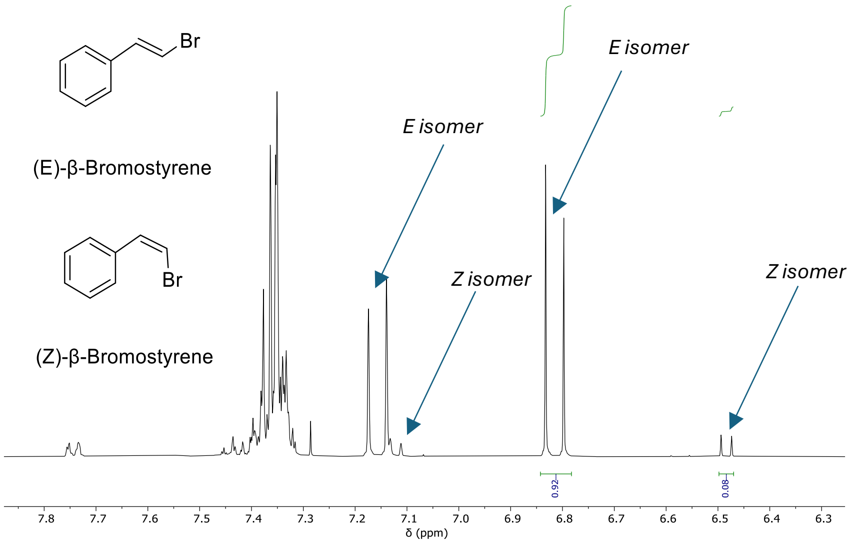

Figure S77: Mixed isomer sample of β-Bromostyrene starting substrate

Once isolated, mixed isomer and pure (E) β-bromostyrene samples were used as substrates for photocatalysed aminocarbonylation reactions. In a glovebox under N_2, $Co_2(CO)_8$ (0.2 mmol, 0.068g) was weighed into an ampoule, sealed under N_2 and removed from the glovebox. *t*-AmylOH (4 mL) was added to the ampoule using standard Schlenk line techniques to create a catalyst stock solution. In a separate, nitrogen flushed flask was added DBU (0.657 ml, 4.4 mmol), piperidine (0.515 ml, 5.2 mmol, 1.3 equiv.) and t-amylOH (2.83 ml) to afford a 4 mL amine stock solution. To each illumin8 vial was added aryl vinyl bromides (1 mmol) under a flow of N_2. Subsequently, the amine stock solution (1 mL) was added into each reactor vial, stirred and then followed by addition of the catalyst stock solution (1 mL). Reaction mixtures were then frozen using a dry ice/acetone bath. The mixtures were then subjected to three vacuum-CO (1 bar) cycles and subsequently charged with the appropriate CO pressure. The illumin8 reactor was then assembled around the CO charged vials, then stirring and LEDs were switched on. After 16 hours of illumination the headspace was opened to air and reaction mixtures were diluted with EtOAc (25 mL) and HCl (1M, 10 mL). The extracted organic layer was dried and analysed crude to assess product distribution.

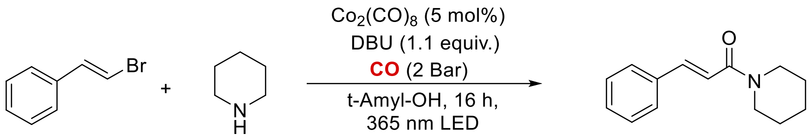

From mixed isomers

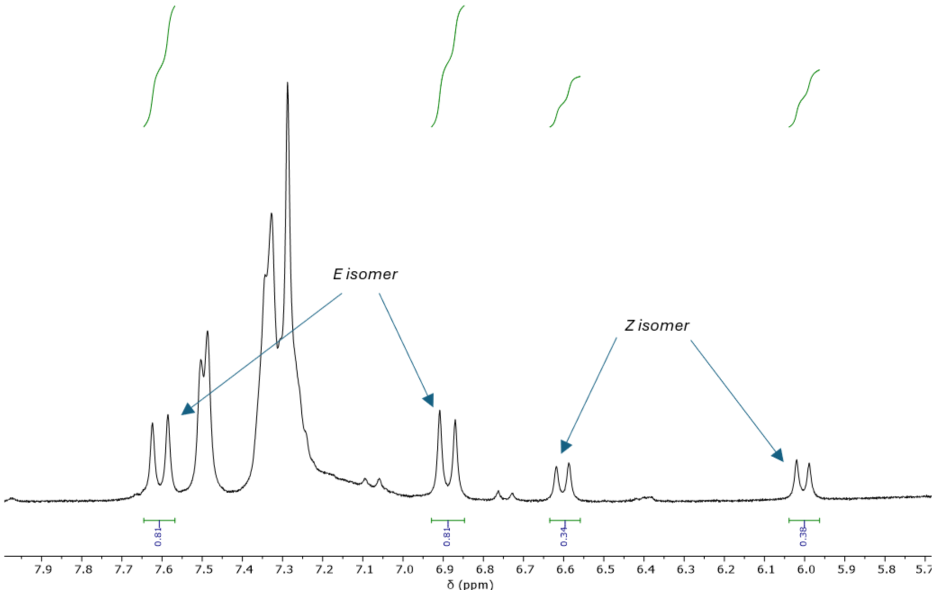

Figure S78: Carbonylation product mixture from mixed isomer sample of β-Bromostyrene. Significant scrambling of the stereochemistry is observed with product E:Z ratio of 68:32 amide (Conversion of substrate = 93%).[6, 7]

From pure isomer (E)

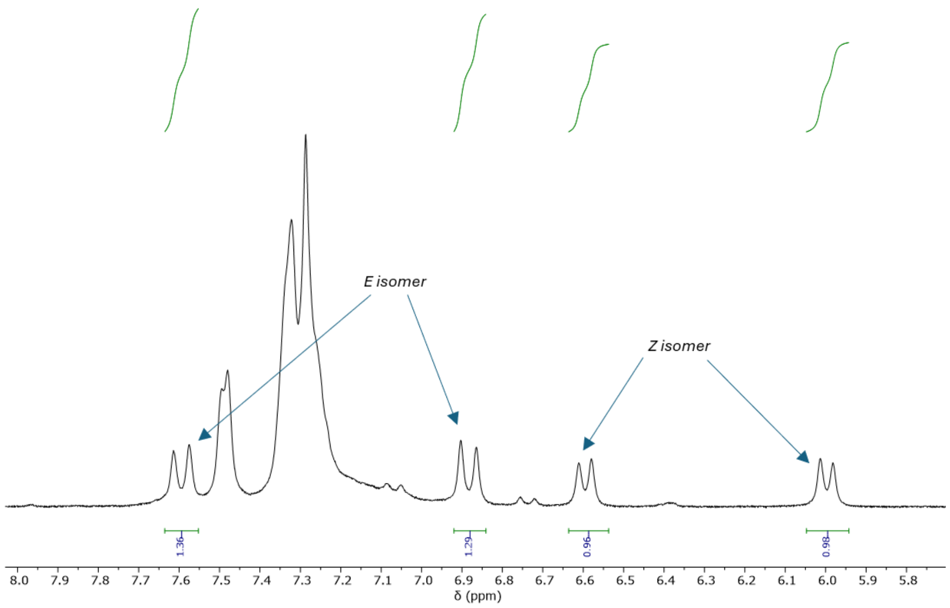

Figure S79: Carbonylation product mixture from pure isomer sample of β-Bromostyrene (E). Significant scrambling of the stereochemistry is observed with product E:Z ratio of 57:43 amide (Conversion of substrate = 92%). [6, 7]

Investigation of Acyl Cobalt Complex Synthesis

From previous literature precedent, in absence of phosphine ligands, cobalt acyl complexes spontaneously decarbonylate to form aryl complexes in absence of a CO headspace (see below).

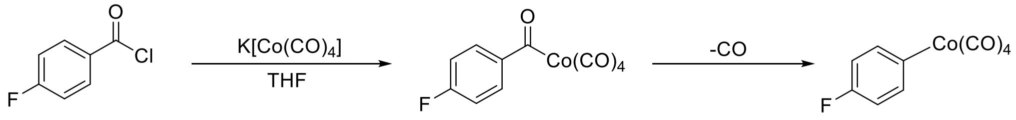

To investigate this, 4-fluorobenzoylchloride was synthesized according to **General Stoichiometric Amide Synthesis Procedure**. In a glovebox under an N_2 environment, acyl chloride (15.8 - 103 mg, 0.1 – 0.65 mmol) was added to a solution of K[Co(CO)$_4$] (21 mg, 0.1 mmol) in THF (1 mL) at variable loadings. Reaction mixtures were then transferred into a J-Young NMR tube and analysed by ^{1}H, ^{19}F and ^{59}Co NMR spectroscopy. Once analysed, tubes were subject to freeze-pump-thaw cycles then filled with CO (1 bar). The tubes were then analysed by the same methods.

^{19}F NMR Spectra Comparison

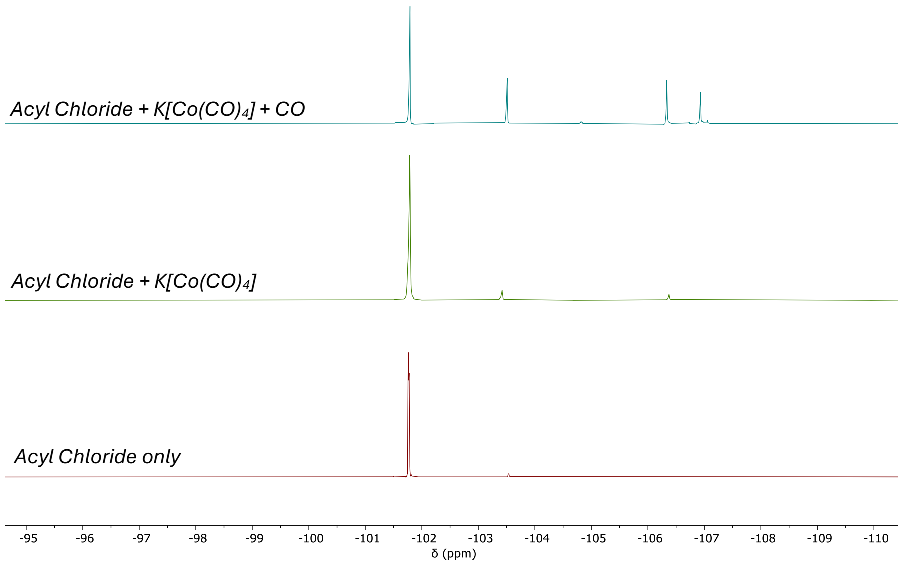

^{59}Co NMR Spectra Comparison

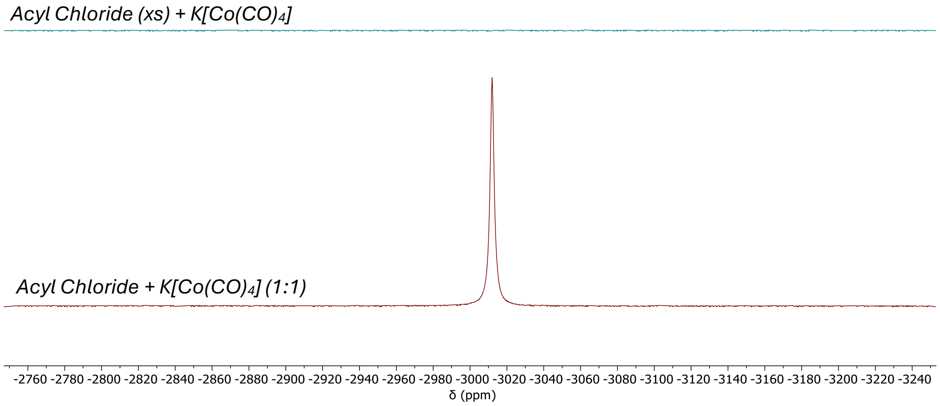

Figure S80: Sequential addition of 4-fluorobenzoyl chloride to K[Co(CO)$_4$]. ^{19}F NMR analysis (top) shows formation of new fluorine containing products upon addition. Implementation of a CO headspace in NMR tube (1 bar) affords a new peak assigned to acyl cobalt complex. Excess titration of acyl chloride leads to full consumption of K[Co(CO)$_4$] by ^{59}Co NMR analysis (bottom). This finding closely aligns with prior hypothesis of equilibria aryl/acyl complexation.

Radical Trap Synthesis and Carbonylation Testing

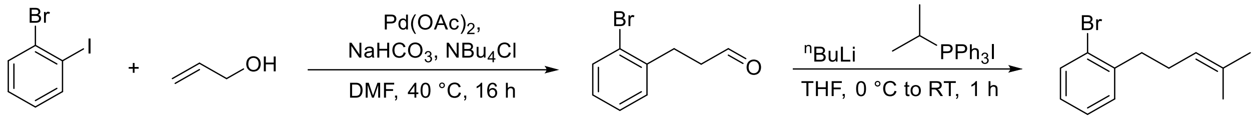

Synthesis of 1-bromo-2-(4-methylpent-3-en-1-yl)benzene was conducted from a literature procedure according to the above scheme.[8] Product alkene was isolated in 60% yield (430 mg isolated from 3 mmol 2-bromoiodobenzene). ^{1}H NMR (400 MHz, $CDCl_3$) δ 7.52 (d, 1H), 7.25 – 7.17 (m, 2H), 7.05 (ddd, J = 7.9, 6.2, 2.8 Hz, 1H), 5.20 (ddq, J = 8.7, 5.7, 1.4 Hz, 1H), 2.75 (dd, 2H), 2.35 – 2.24 (q, 2H), 1.70 (s, 3H), 1.57 (s, 3H).^{1}H NMR spectroscopy was in agreement with previous literature. [8]

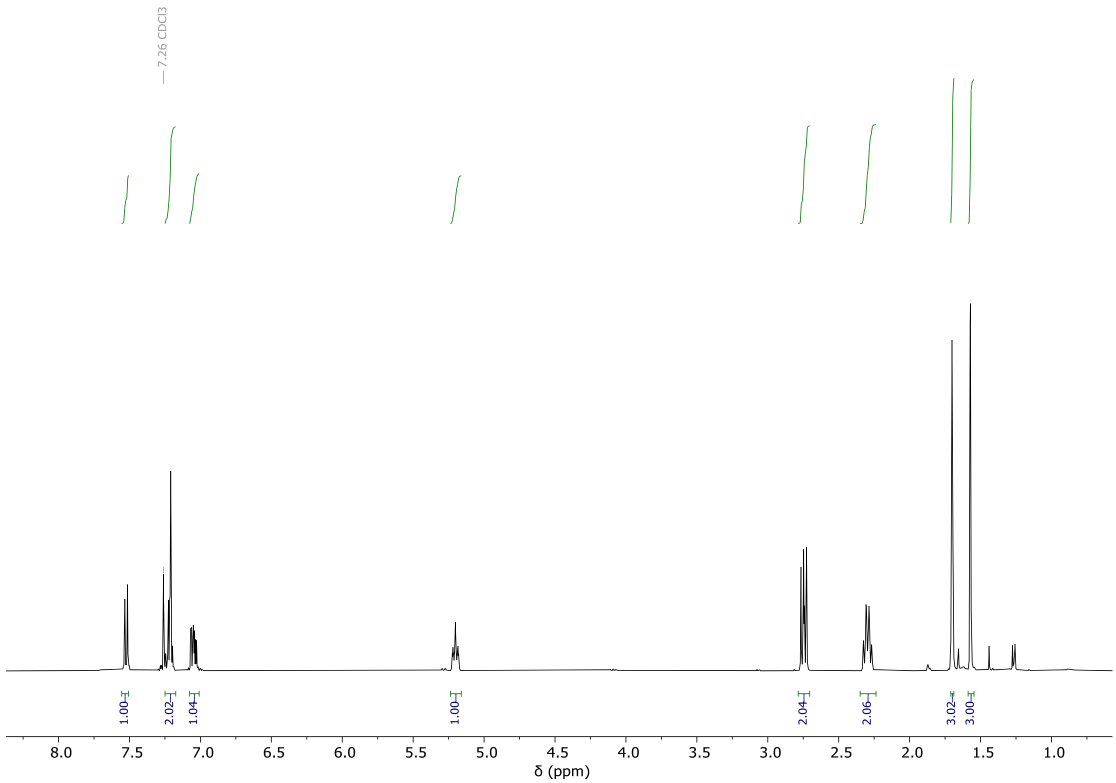

Figure S81: Acquired spectra of pure 1-bromo-2-(4-methylpent-3-en-1-yl)benzene following column chromatographic isolation.

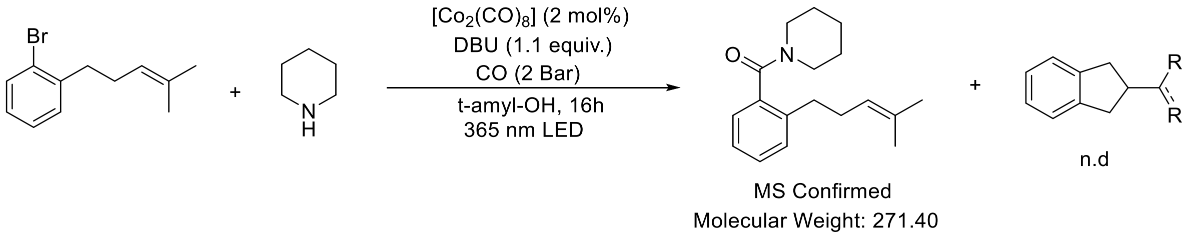

1-bromo-2-(4-methylpent-3-en-1-yl)benzene was subjected to standard conditions as described in **General Aminocarbonylation Procedure B**: In a glovebox under N_2, $Co_2(CO)_8$ (0.04 mmol, 0.0136g) was weighed into an ampoule, sealed under N_2 and removed from the glovebox. *t*-AmylOH (2 mL) was added to the ampoule using standard Schlenk line techniques to create a catalyst stock solution. In a separate, nitrogen flushed flask was added DBU (0.329 ml, 2.2 mmol), piperidine (0.258 ml, 2.6 mmol, 1.3 equiv.) and t-amylOH (1.413 ml) to afford a 2 mL amine stock solution. To an illumin8 vial was added 1-bromo-2-(4-methylpent-3-en-1-yl)benzene (0.239 g, 1 mmol) under a flow of N_2. Subsequently, the amine stock solution (1 mL) was added into each reactor vial, stirred and then followed by addition of the catalyst stock solution (1 mL). Reaction mixtures were then frozen using a dry ice/acetone bath. The mixtures were then subjected to three vacuum-CO (1 bar) cycles and subsequently charged to 2 Bar CO pressure. The illumin8 reactor was then assembled around the CO charged vials, then stirring and LEDs were switched on. After 16 hours of illumination the headspace was opened to air and reaction mixtures were diluted with EtOAc (15 mL) and HCl (1M, 10 mL). The organic products were extracted using EtOAc (3 x 15 mL), dried and analysed by ^1H NMR spectroscopy to assess product distribution.

A modest starting material conversion of 10% was recorded. No evidence of cyclized product was noted based on the precedent set using AIBN in the prior literature.[8] TLC-MS analysis confirmed the molecular ion peak describing the expected piperidine inserted carbonylation product.

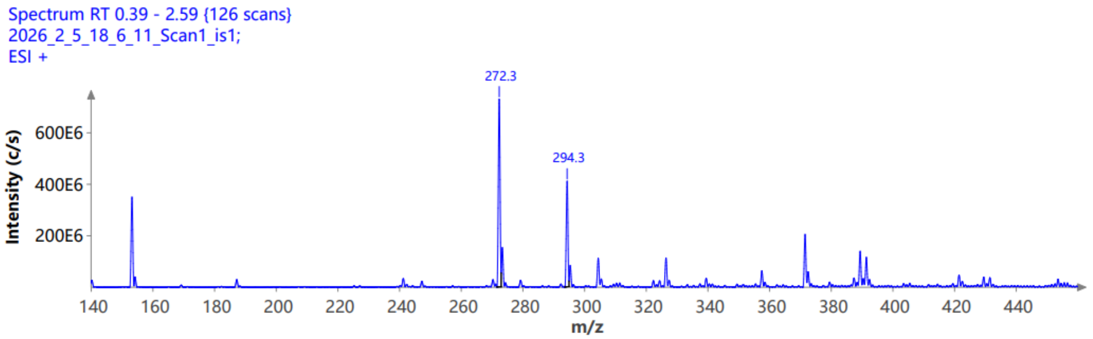

Figure S82: TLC-MS analysis of reaction crude mixture.

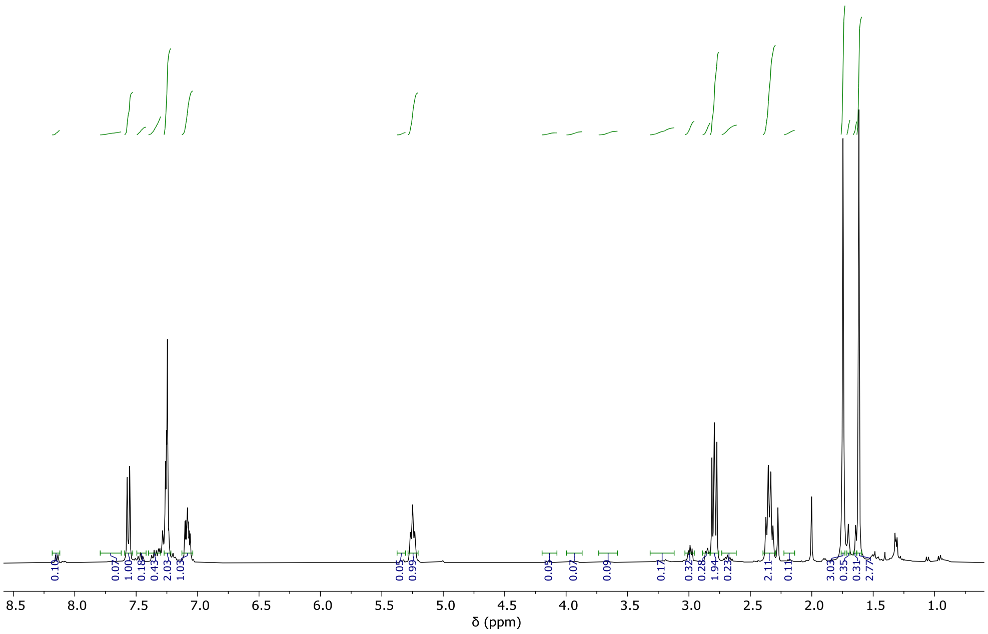

Figure S83: ^{1}H NMR spectroscopic analysis of worked up and dried reaction mixture

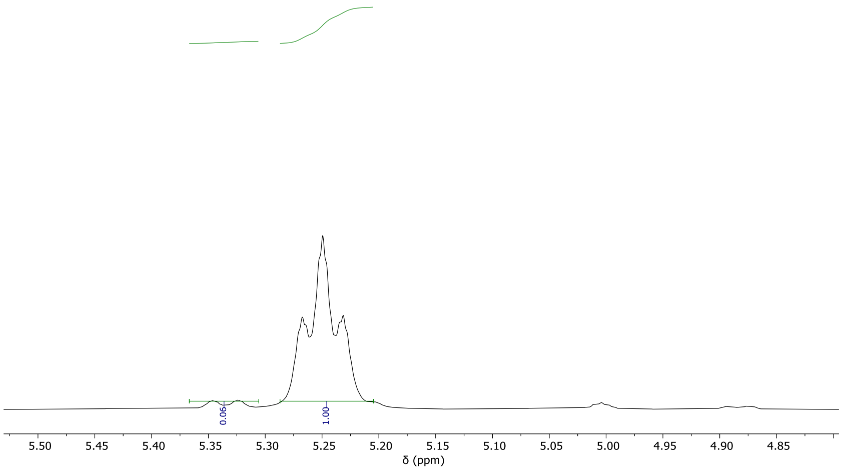

Zoomed in ^{1}H alkene region.

Figure S84: Zoomed alkene region of ^{1}H NMR spectra in S82.

SET vs Bond Breaking Competition Experiment

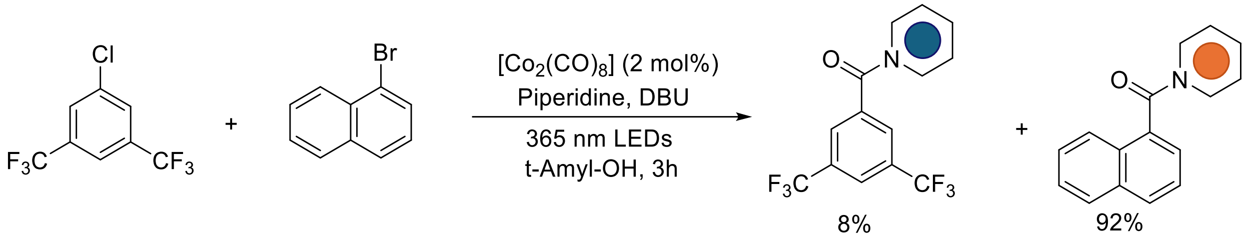

A competition experiment was hosted between two electrophile substrates. The electronically activated chloride has a lower reduction potential (-2.07 V) versus the unactivated naphthalene bromide (-2.23 V). An SET process is therefore expected to favour the insertion into the chloride while bond breaking focused reactivity will favour reaction of the naphthalene bromide. Reactions were hosted via modified **General Aminocarbonylation Procedure B**. In a glovebox under N_2, $Co_2(CO)_8$ (0.04 mmol, 0.0136g) was weighed into an ampoule, sealed under N_2 and removed from the glovebox. *t*-AmylOH (2 mL) was added to the ampoule using standard Schlenk line techniques to create a catalyst stock solution. In a separate, nitrogen flushed flask was added DBU (0.329 ml, 2.2 mmol), piperidine (0.258 ml, 2.6 mmol, 1.3 equiv.) and t-amylOH (1.413 ml) to afford a 2 mL amine stock solution. To an illumin8 vial was added 3,5-di(trifluoromethyl)bromobenzene (0.293 g, 1 mmol) and 1-bromonaphthalene (0.207 g, 1 mmol) under a flow of N_2. Subsequently, the amine stock solution (1 mL) was added into each reactor vial, stirred and then followed by addition of the catalyst stock solution (1 mL). Reaction mixtures were then frozen using a dry ice/acetone bath. The mixtures were then subjected to three vacuum-CO (1 bar) cycles and subsequently charged to 2 Bar CO pressure. The illumin8 reactor was then assembled around the CO charged vials, then stirring and LEDs were switched on. After 16 hours of illumination the headspace was opened to air and sampled for crude 1H NMR analysis. Following this, reaction mixtures were diluted with EtOAc (15 mL) and HCl (1M, 10 mL). The organic products were extracted using EtOAc (3 x 15 mL), dried and further analysed by ^1H NMR spectroscopy to assess product distribution.

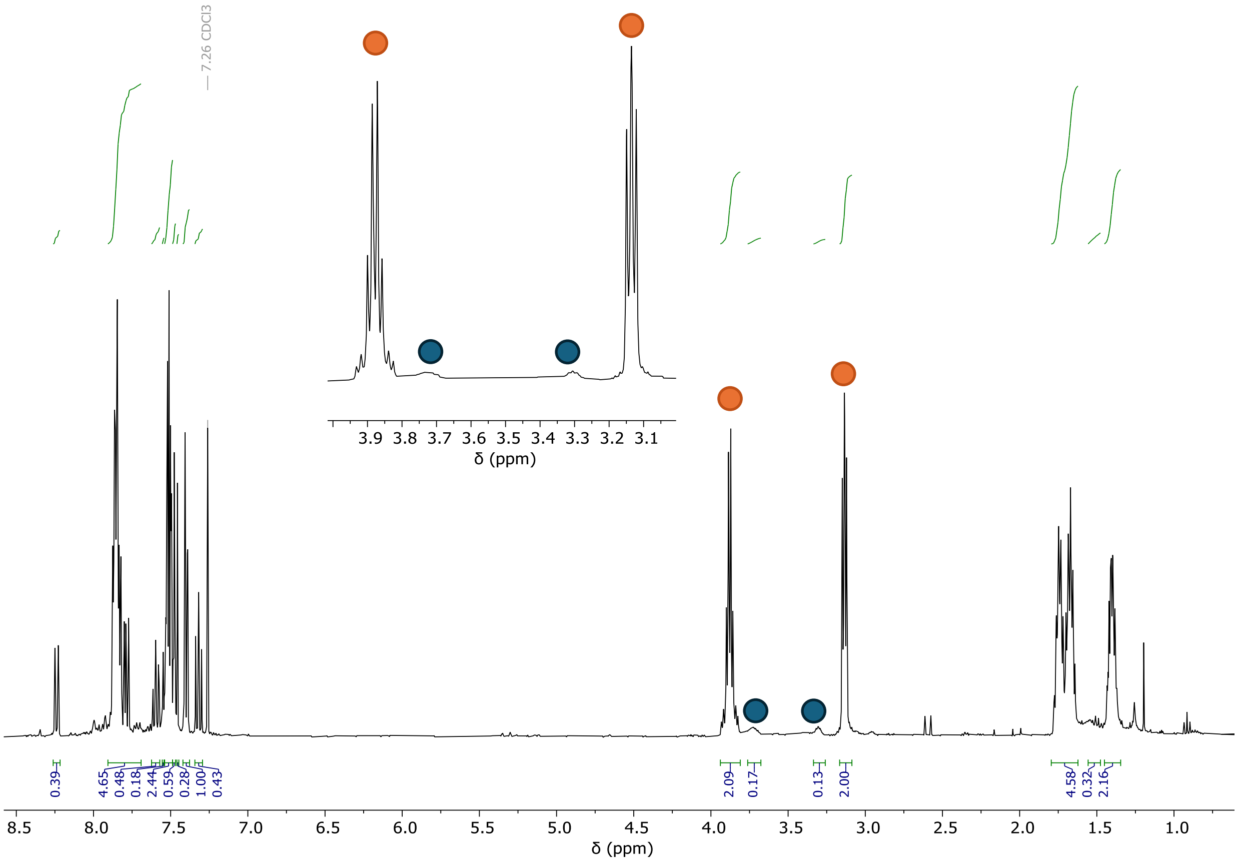

Figure S85: Crude product distribution of competition carbonylation experiment.

The naphthalene substrate reacted preferably under carbonylation conditions (92:8), (Br:Cl), suggesting that cleavage of carbon-halogen bonds dictates reactivity rather than SET. Given the excess concentration of piperidine, and evidence of full conversion of 1-bromonaphthalene starting material, it is feasible that the chloride reagent conversion occurs subsequently to bromide consumption.

Quantum Yield Calculation:

Experimentally determined rate of reaction (Ar-CF_3): 0.54 mmol/h ==> 1.50 x10^{-7} mol/s (SI)

Experimentally determined rate of reaction (Ar-OMe): 0.27 mmol/h ==> 7.50 x10^{-8} mol/s (SI)

Rate of Photon Delivery (1 x 10W, 365 nm LED): 7.00 mmol/h ==> 1.94 x10^{-6} mol/s (SI)

QY (Φ) = Rate of Reaction* / Rate of Photon Delivery

QY(CF_3) = 0.077 = 7.7%

QY (OMe) = 0.0387 = 3.9%

**Assuming 1 photon = 1 reaction*

GC Calibration Curves

4-(methoxy)benzoylpiperidine – Synthesis & Calibration

For calibration, 4-(methoxy)benzoylpiperidine was synthesized via **General Stoichiometric Amide Synthesis Procedure** (3.55 g ,88%). The product was isolated as a yellow oil with no further purification necessary. ^{1}H NMR (400 MHz, $CDCl_3$) δ 7.37 (d, J = 8.2 Hz, 2H), 6.90 (d, J = 8.2 Hz 2H), 3.83 (s, 3H), 3.60 (s, 4H), 1.69 (m, 2H), 1.60 (s, 4H). Physical and spectral data agreed with literature data.[9]

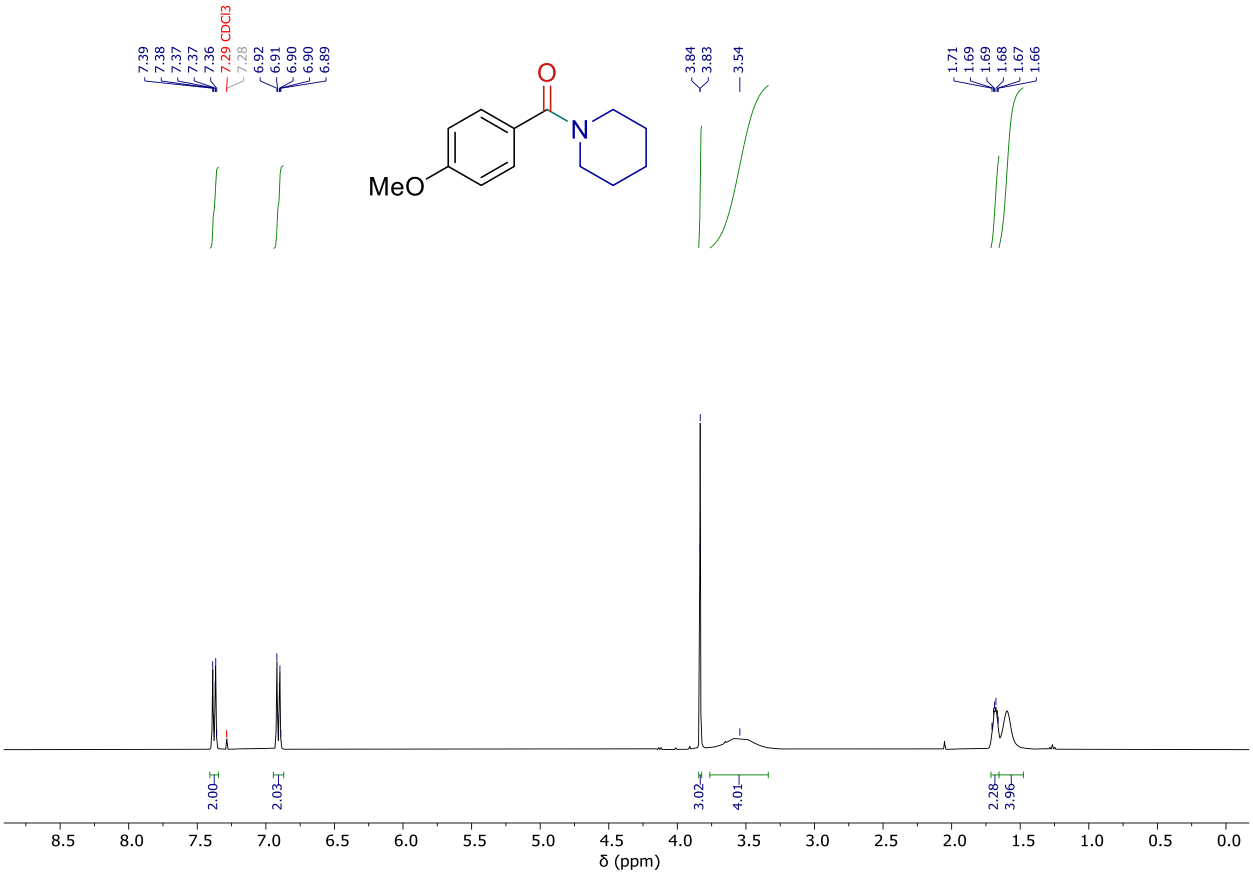

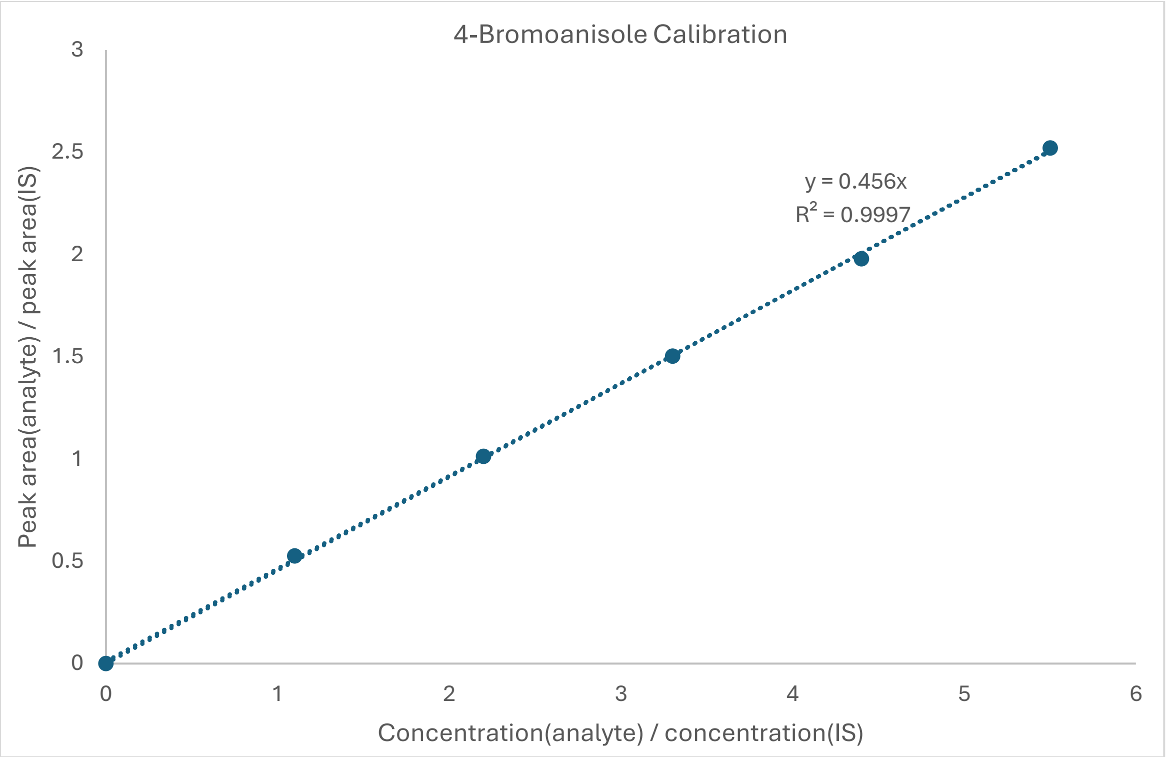

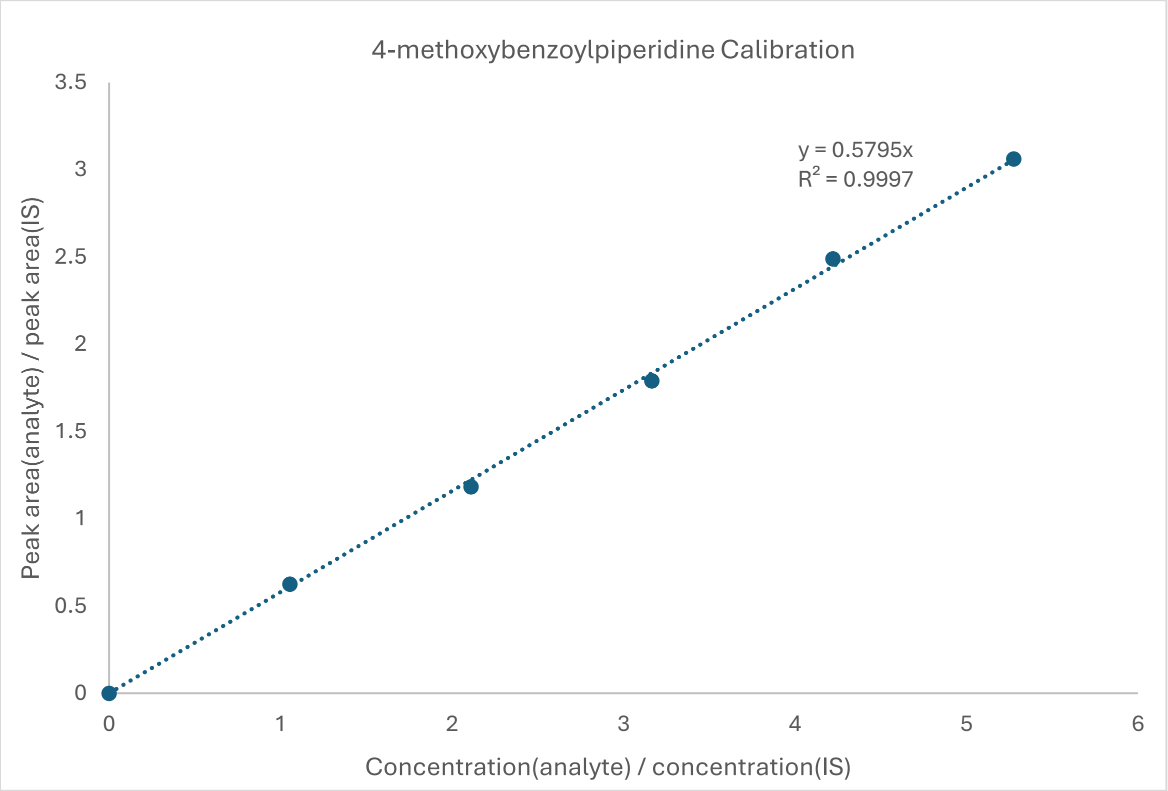

Figure S81: GC Calibration curve for 4-bromoanisole (top) and 4-methoxybenzoylpiperidine (bottom) versus n-dodecane

4-(fluoro)benzoylpiperidine – Synthesis & Calibration

For calibration, 4-fluorobenzoylpiperidine was synthesized via **General Stoichiometric Amide Synthesis Procedure** (0.37 g, 46%). The product was isolated as a white solid with no further purification necessary. ^{1}H NMR (400 MHz, $CDCl_3$) δ 7.44 – 7.31 (m, 2H), 7.05 (t, J = 8.6 Hz, 2H), 3.91 – 3.12 (m, 4H), 1.74 – 1.40 (m, 6H). ^{19}F NMR (376 MHz, $CDCl_3$) δ -110.95. Physical and spectral data agreed with literature data.[9]

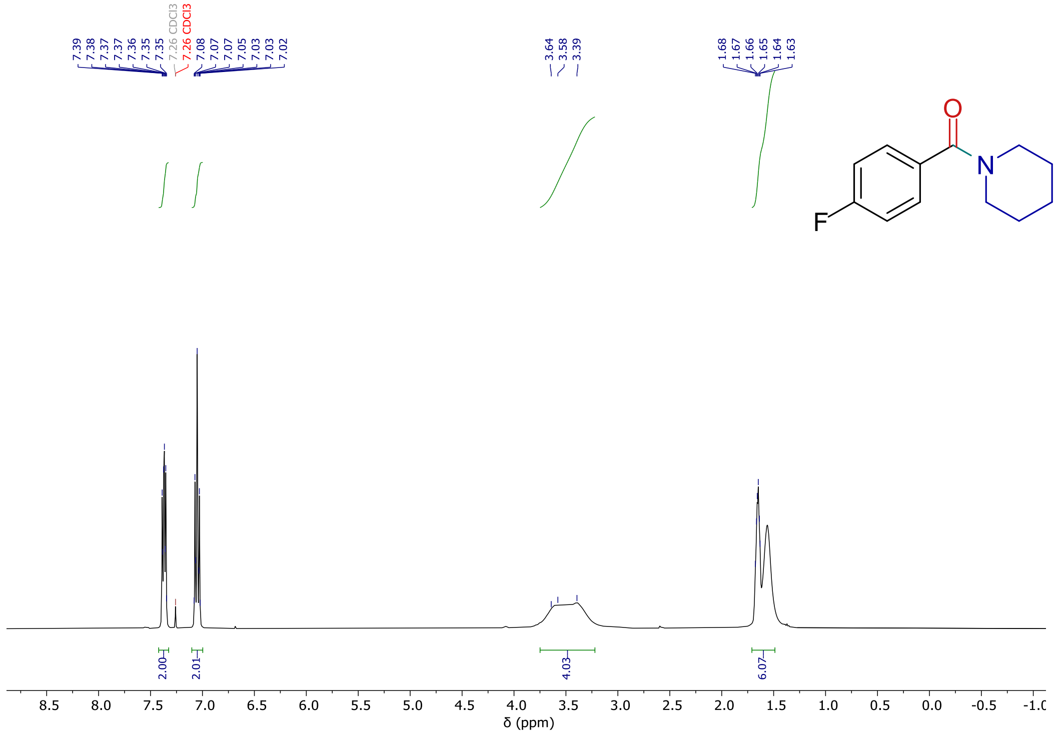

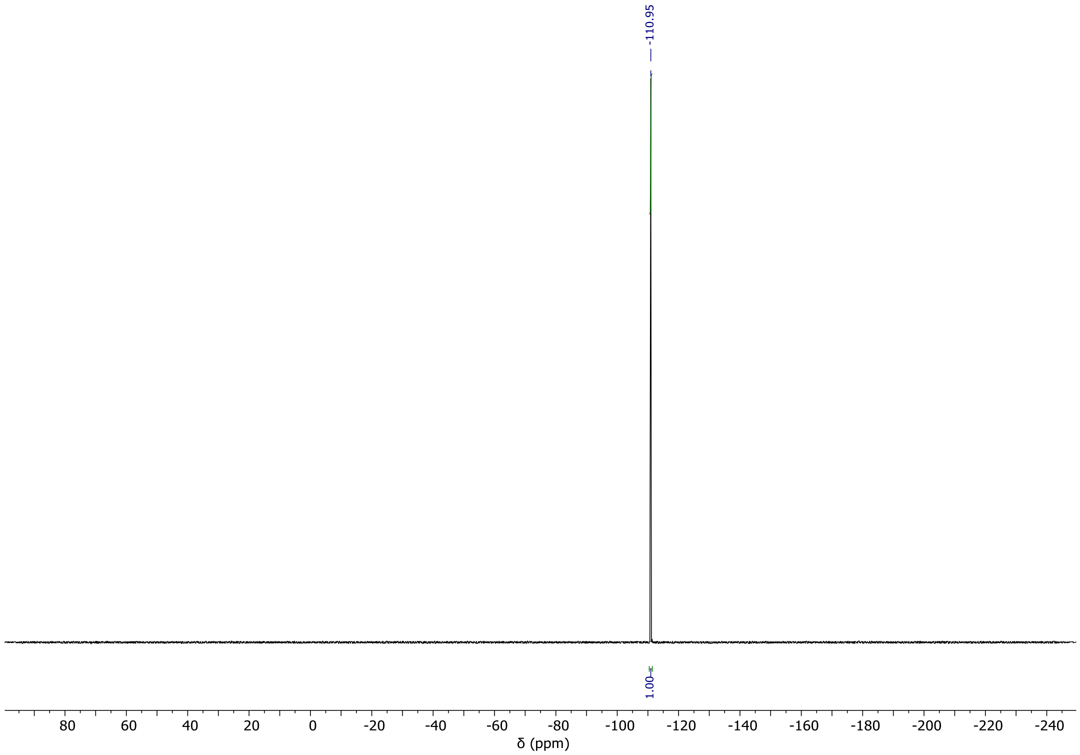

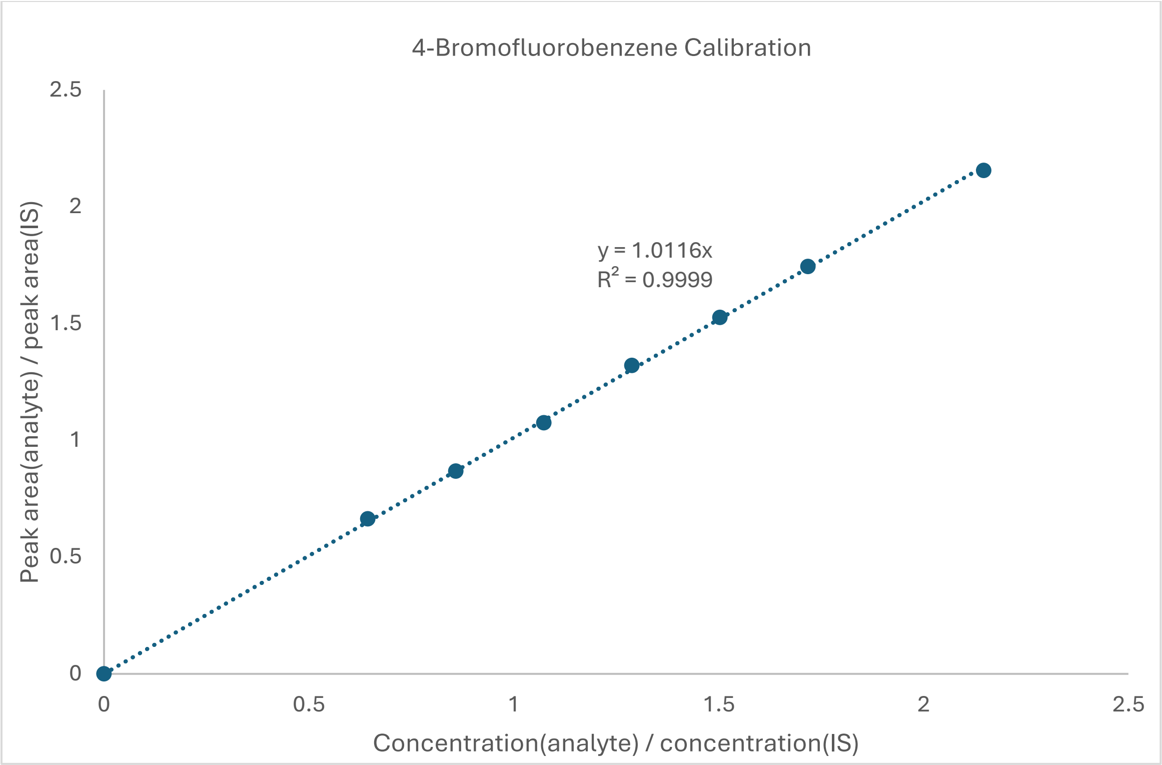

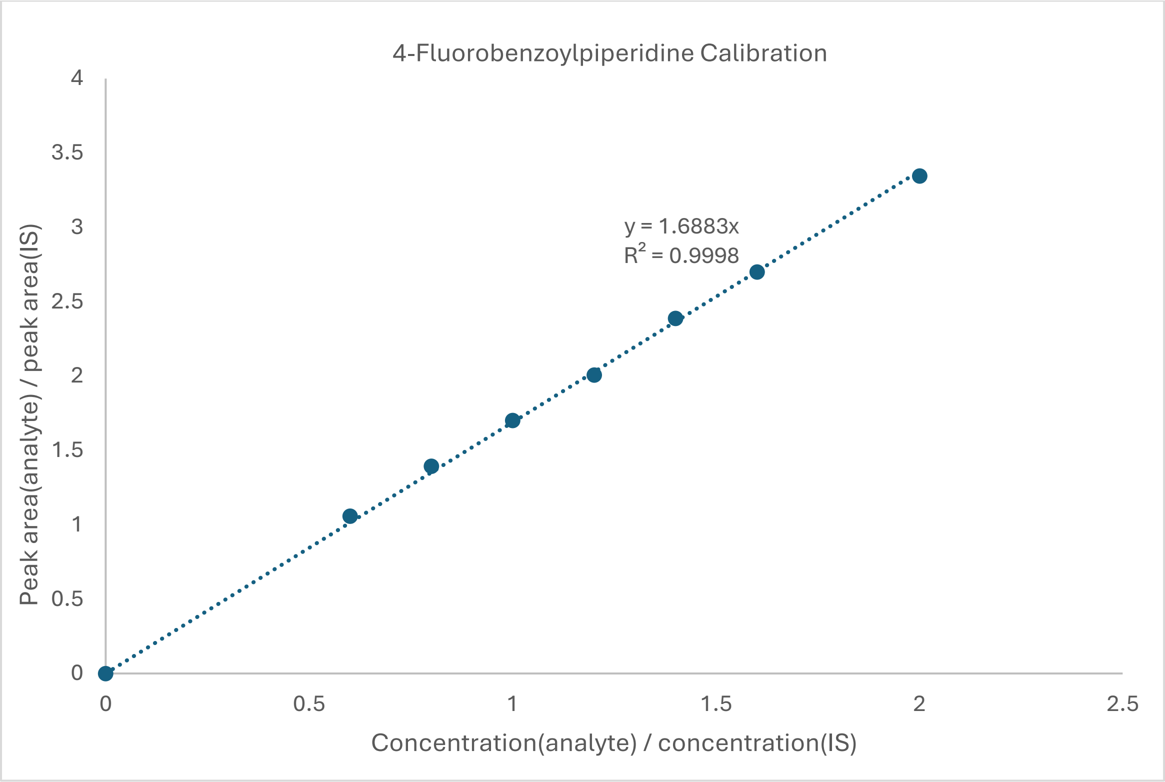

Figure S82: GC Calibration curve for 4-bromofluorobenzene (top) and 4-fluorobenzoylpiperidine (bottom) versus n-dodecane

Benzoylpiperidine – Synthesis & Calibration

For calibration, benzoylpiperidine was synthesized via **General Stoichiometric Amide Synthesis Procedure** (2.24 g, 68%). The product was isolated as a pale orange oil with no further purification necessary. ^{1}H NMR (400 MHz, $CDCl_3$) δ 7.35 (s, 5H), 3.67 (s, 2H), 3.30 (s, 2H), 1.63 (s, 4H), 1.48 (s, 2H). Physical and spectral data agreed with literature data.[9]

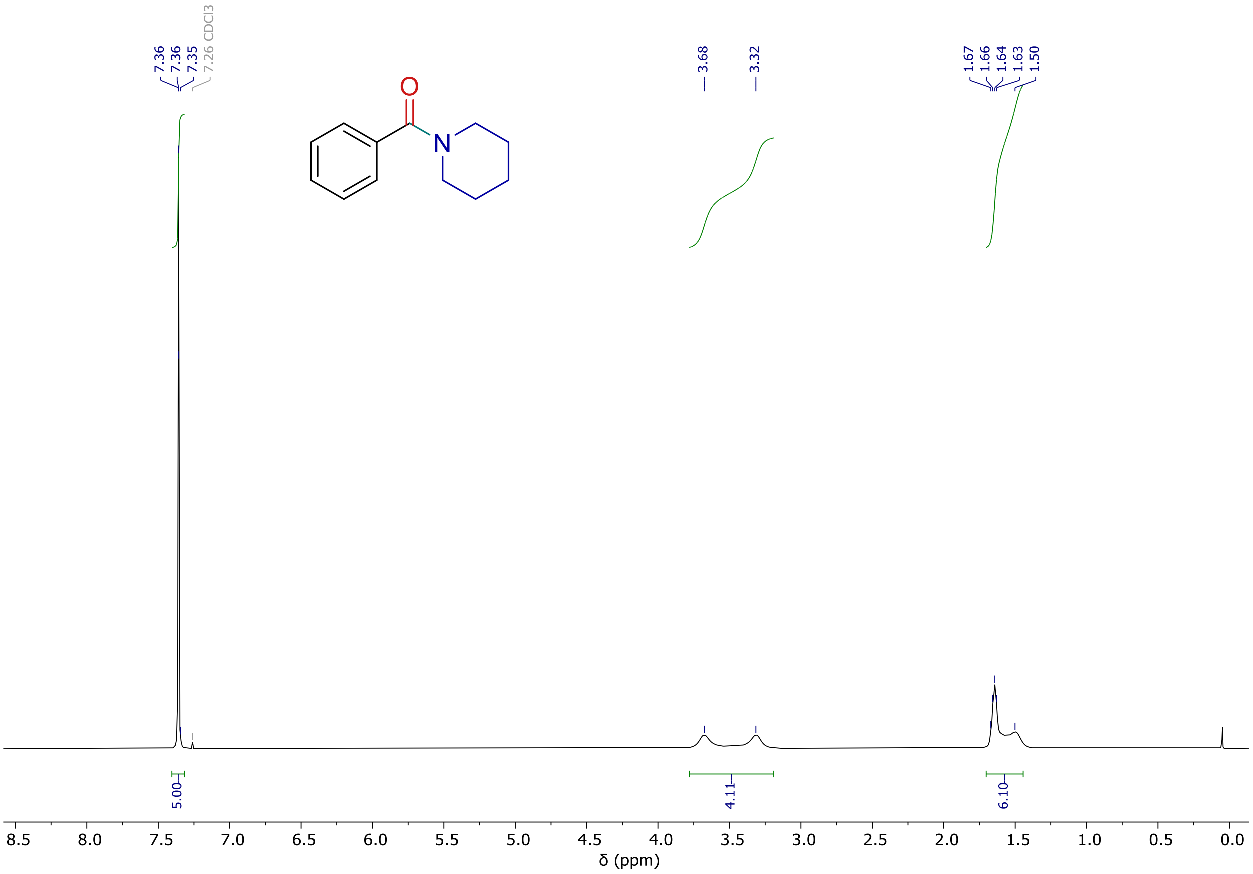

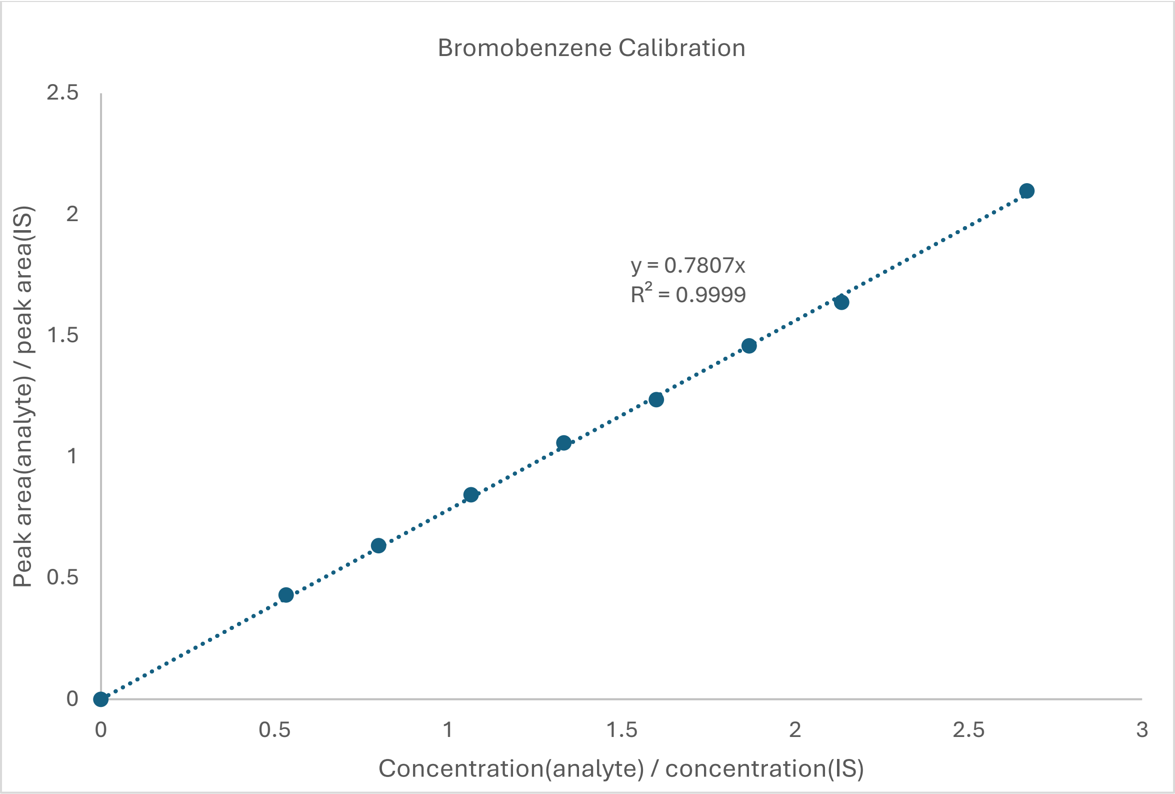

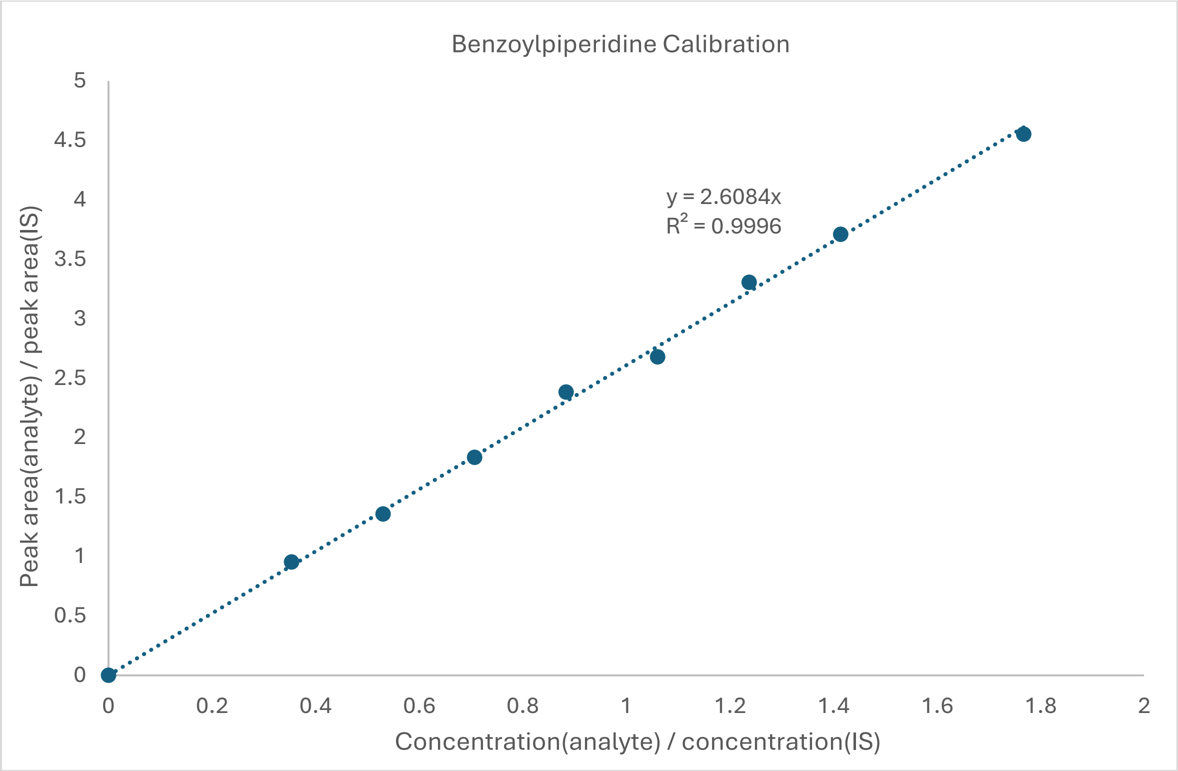

Figure S83: GC Calibration curve for 4-bromobenzene (top) and benzoylpiperidine (bottom) versus n-dodecane

4-(chloro)benzoylpiperidine – Synthesis & Calibration

For calibration, 4-chlorobenzoylpiperidine was synthesized via **General Stoichiometric Amide Synthesis Procedure** (4.03 g, 90%). The product was isolated as a white solid with no further purification necessary. ^{1}H NMR (400 MHz, $CDCl_3$) δ 7.43 – 7.30 (m, 4H), 3.67 (br s, 2H), 3.35 (br s, 2H), 1.79 – 1.64 (m, 3H). Physical and spectral data agreed with literature data.[10]

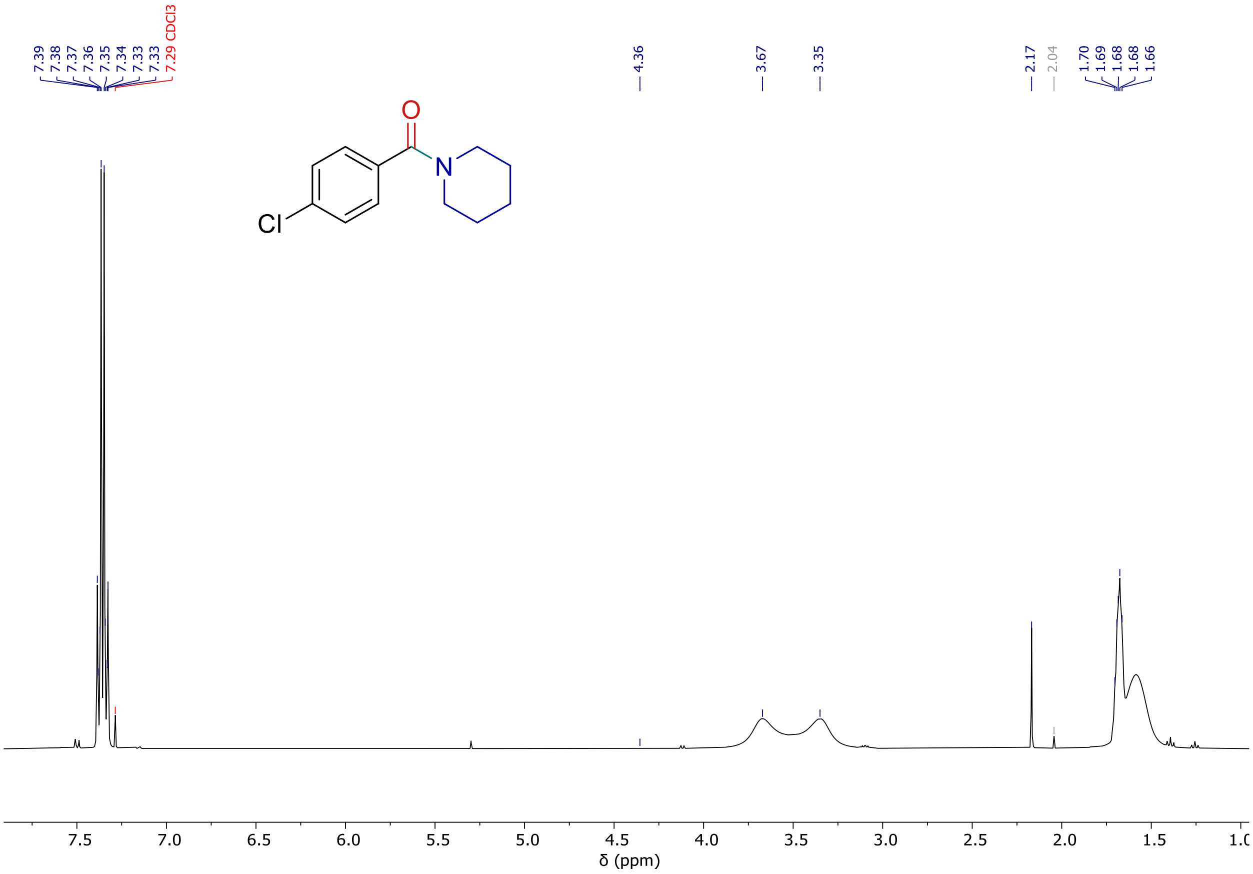

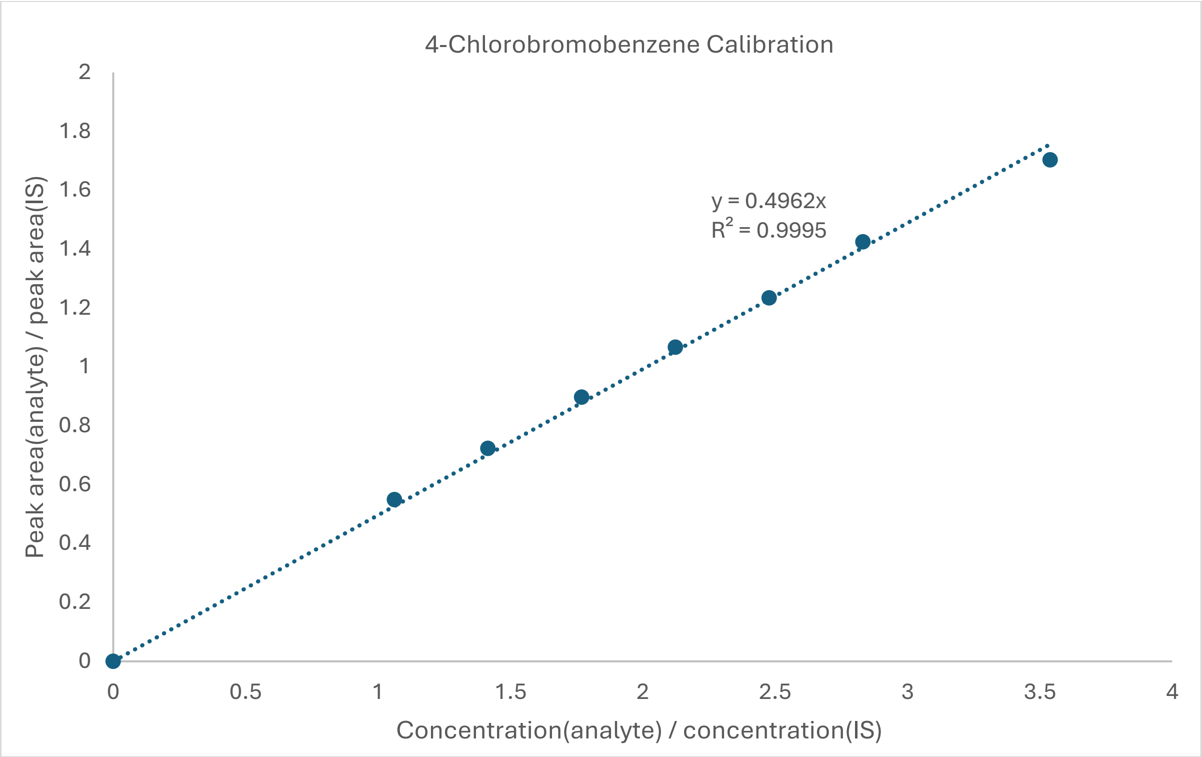

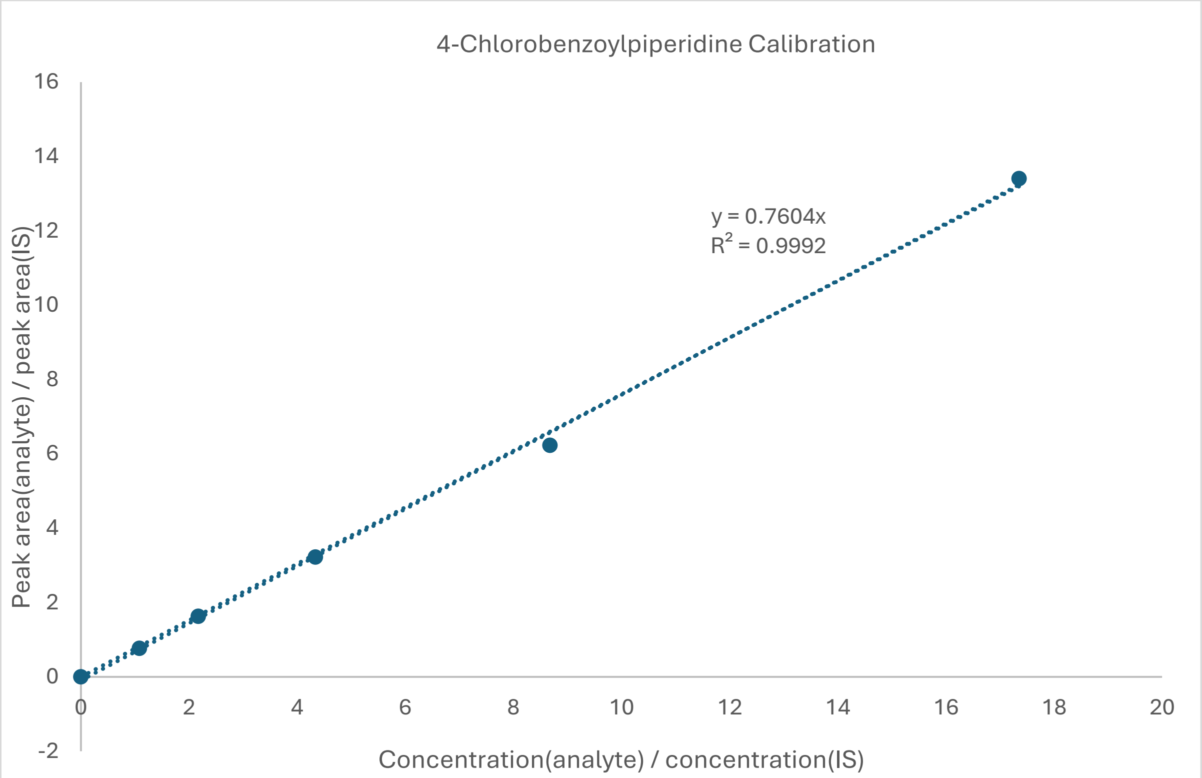

Figure S84: GC Calibration curve for 4-chlorobromobenzene (top) and 4-chlorobenzoylpiperidine (bottom) versus n-dodecane

Methyl 4-(1-piperidinylcarbonyl)benzoate – Synthesis & Calibration

For calibration, methyl 4-(1-piperidinylcarbonyl)benzoate was synthesized via **General Stoichiometric Amide Synthesis Procedure** (0.98 g, 80 %). The product was isolated as a white solid with no further purification necessary (0.98 g, 80 %). ^{1}H NMR (400 MHz, $CDCl_3$) δ 8.07 (d, J = 8.0 Hz, 2H), 7.44 (d, J = 8.0 Hz, 2H), 3.93 (s, 3H), 3.71 (s, 2H), 3.28 (s, 2H), 1.68 (s, 4H), 1.51 (s, 2H). Physical and spectral data agreed with literature data.[9]

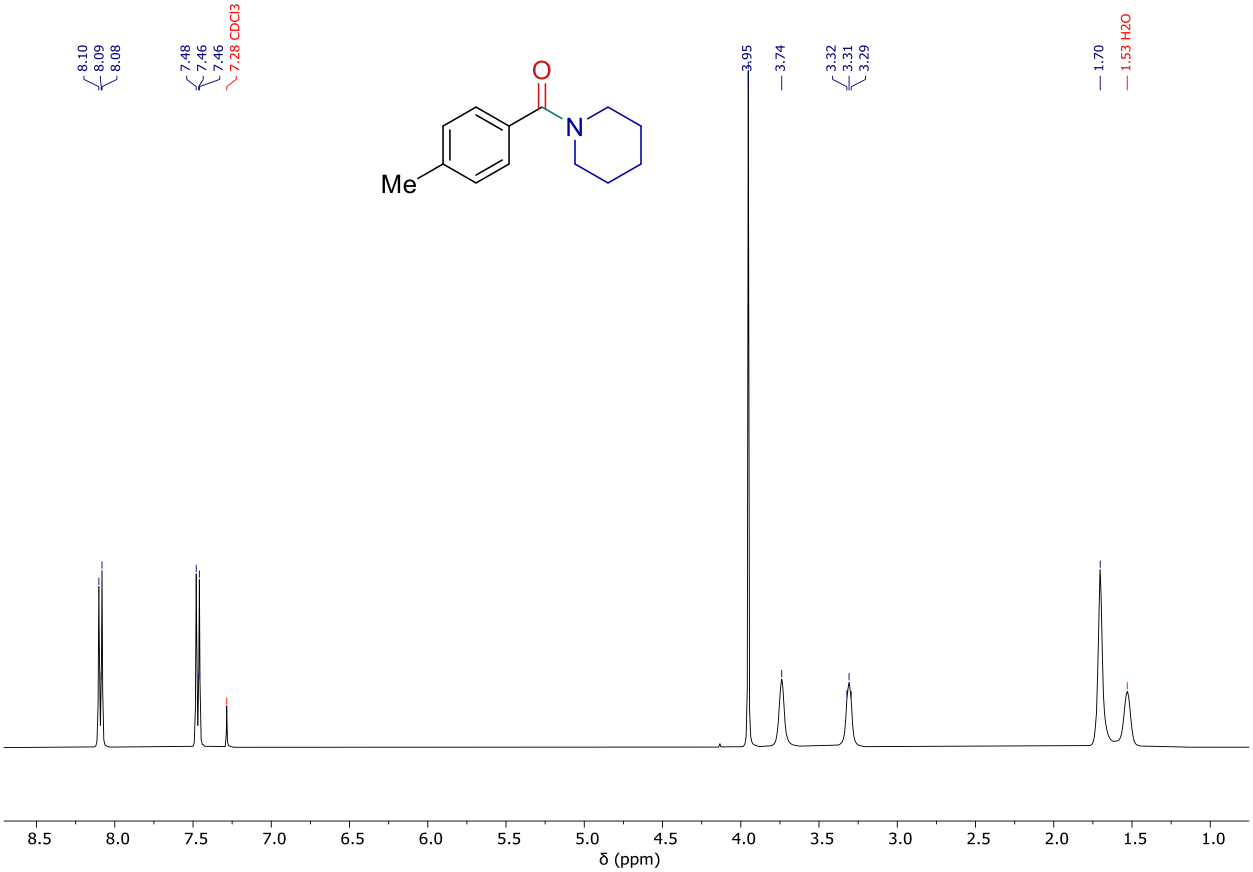

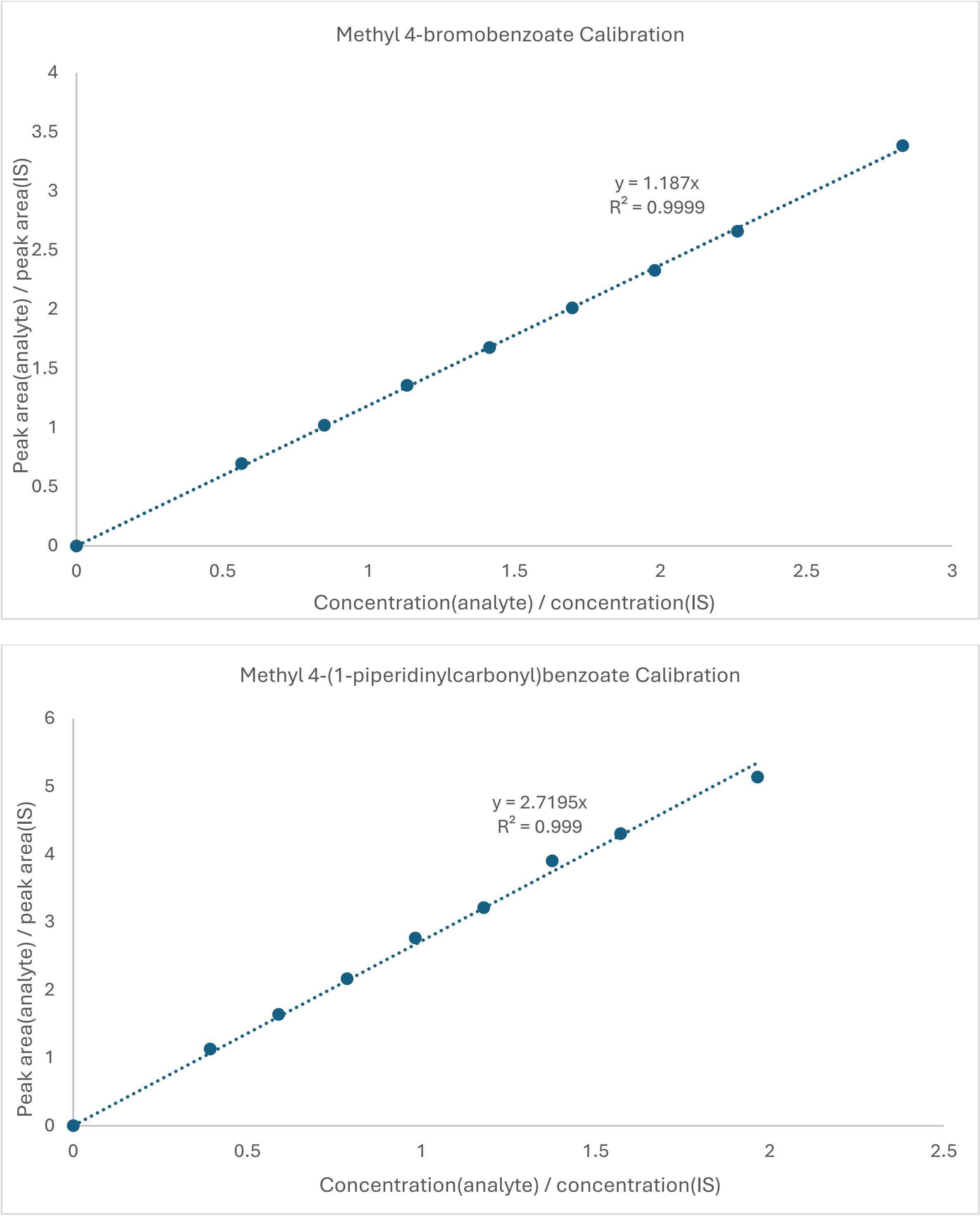

Figure S85: GC Calibration curve for methyl-4-bromobenzoate (top) and methyl 4-(1piperidinylcarbonyl)benzoate (bottom) versus n-dodecane

4-(trifluoromethyl)benzoylpiperidine – Synthesis & Calibration

For calibration, 4-(trifluoromethyl)benzoylpiperidine was synthesized via **General Stoichiometric Amide Synthesis Procedure** (3.99 g, 58%). The product was isolated as a white solid with no further purification necessary. ^{1}H NMR (400 MHz, $CDCl_3$) δ 7.68 (d, *J* = 8.0 Hz, 2H), 7.52 (d, *J* = 8.0 Hz, 2H), 3.74 (m, 2H), 3.31 (m, 2H), 1.70 (m, 4H), 1.58 – 1.47 (m, 2H). ^{19}F NMR (376 MHz, $CDCl_3$) δ (-110.9). Physical and spectral data agreed with literature data.[9]

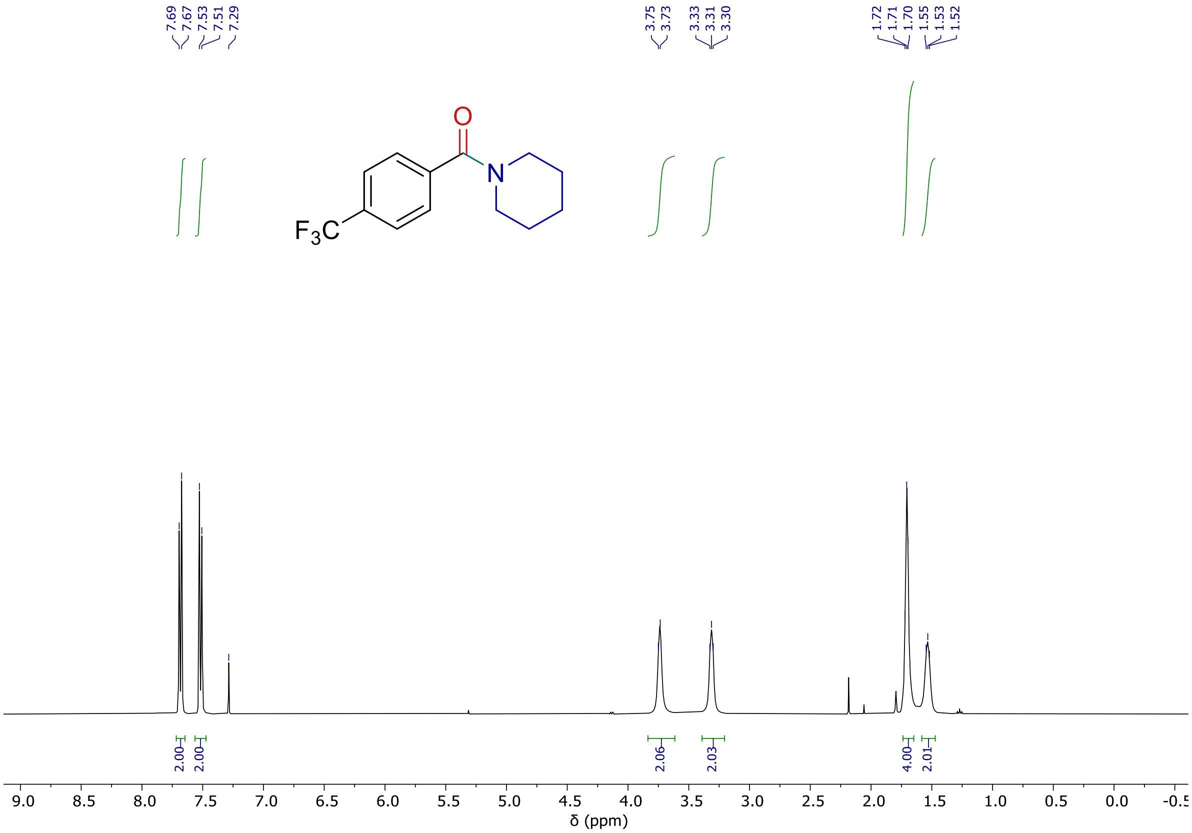

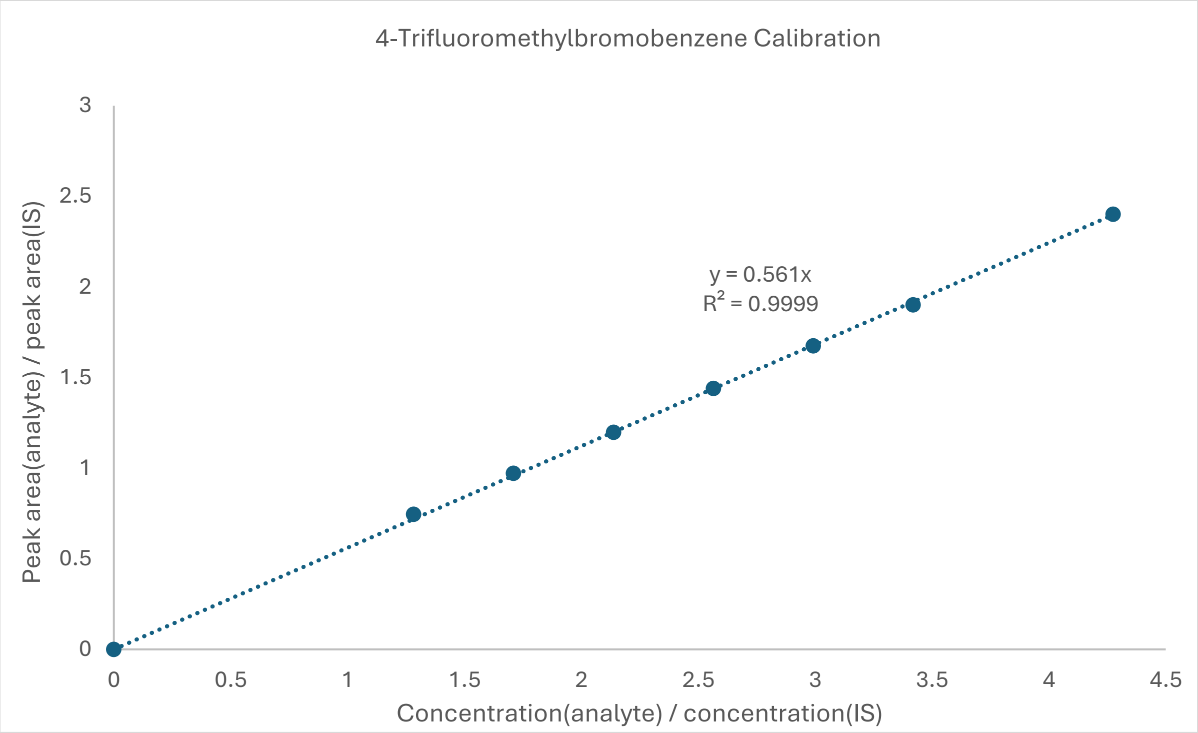

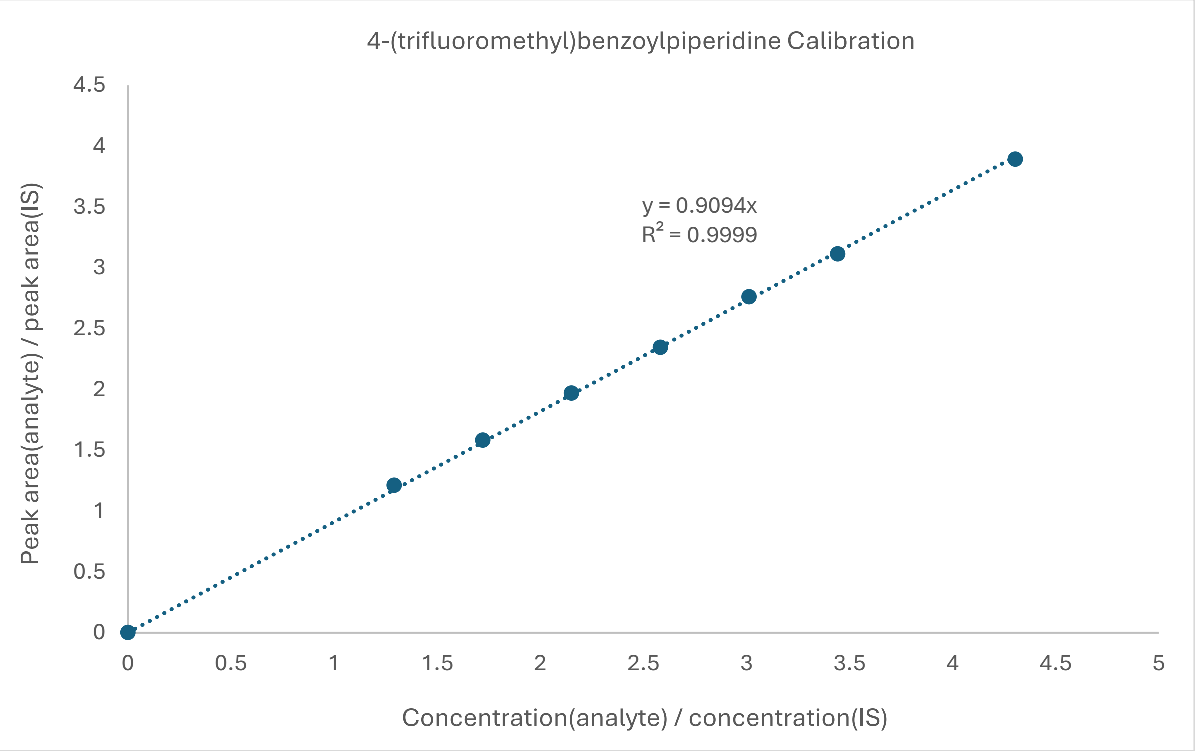

Figure S88: GC Calibration curve for 4-trifluoromethylbromobenzene (top) and 4-(trifluoromethyl)benzoylpiperidine (bottom) versus n-dodecane

^{1}H, ^{19}F NMR Spectra - Scope

(SI-1) - N-(p-methoxybenzoyl)piperidine – The desired product was obtained from **4-Bromoanisole** *via* **General Aminocarbonylation Procedure B**. The product was collected as a yellow oil (228 mg, 99 %). ^{1}H NMR (400 MHz, $CDCl_3$) δ 7.38 (d, J = 8.3 Hz, 2H), 6.91 (d, J = 8.3 Hz, 2H), 3.83 (s, 3H), 3.54 (br s, 4H), 1.74 – 1.64 (m, 2H), 1.60 (br s, 2H). The desired product was also synthesized from **4-Chloroanisole** via **General Aminocarbonylation Procedure C** (134 mg, 61%). Physical and spectra data agreed with literature values.[9]

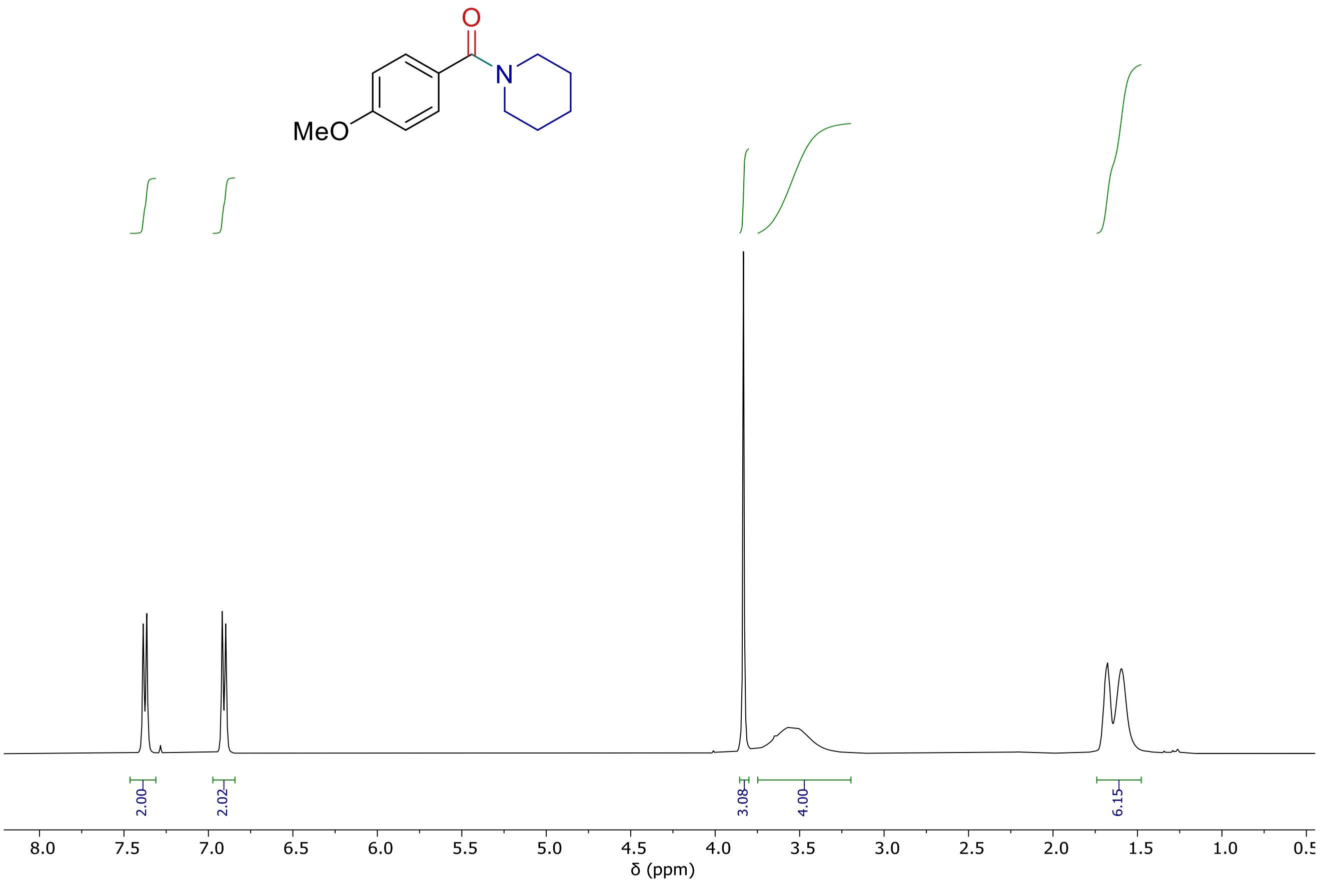

(SI-2) - N-benzoylpiperidine - The desired product was obtained from **4-Bromobenzene** *via* **General Aminocarbonylation Procedure B**. The product was collected as a clear oil (194 mg, >99%). ^{1}H NMR (400 MHz, $CDCl_3$) δ 7.36 – 7.31 (m, 5H), 3.66 (s, 2H), 3.28 (s, 2H), 1.61 (s, 4H), 1.46 (s, 2H). The product was collected as a yellow oil (228 mg, 99 %). ^{1}H NMR. The desired product was also synthesized from **4-Chlorobenzene** via **General Aminocarbonylation Procedure C** (170 mg, 90%). Physical and spectra data agreed with literature values.[9]

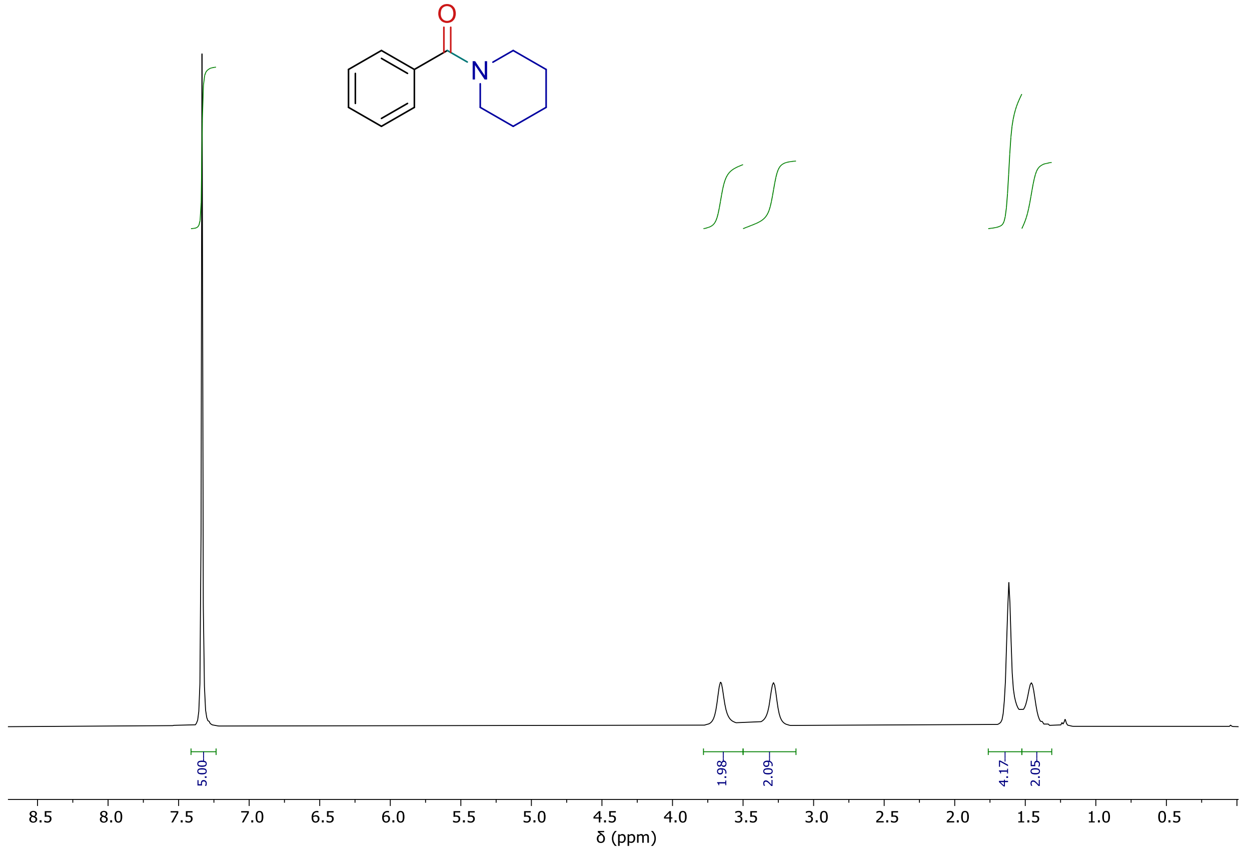

(SI-3) - **N-(p-fluorobenzoyl)piperidine -** The desired product was obtained from **4-Fluorobromobenzene** *via* **General Aminocarbonylation Procedure B**. The product was collected as a clear oil (205 mg, 99 %). ^{1}H NMR (400 MHz, $CDCl_3$) δ 7.41 – 7.33 (m, 2H), 7.04 (t, J = 8.7 Hz, 2H), 3.65 (s, 2H), 3.32 (s, 2H), 1.73 – 1.42 (m, 6H). ^{19}F NMR (377 MHz, $CDCl_3$) δ -110.85 (s). The desired product was also synthesized from **4-Fluorochlorobenzene** via **General Aminocarbonylation Procedure C** (134 mg, 65%). Physical and spectra data agreed with literature values.[9]

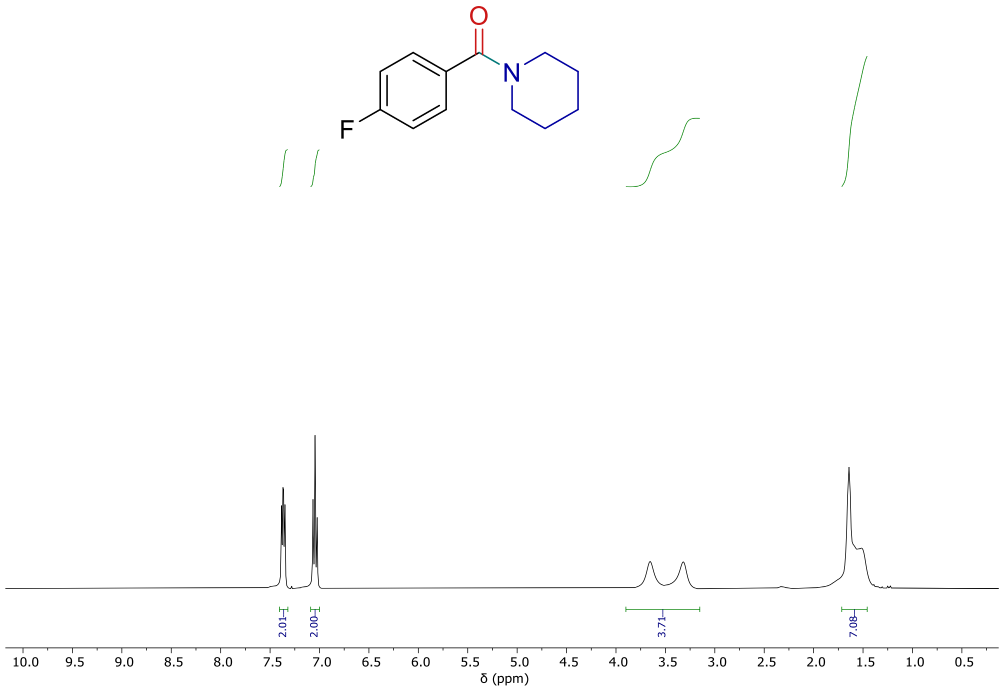

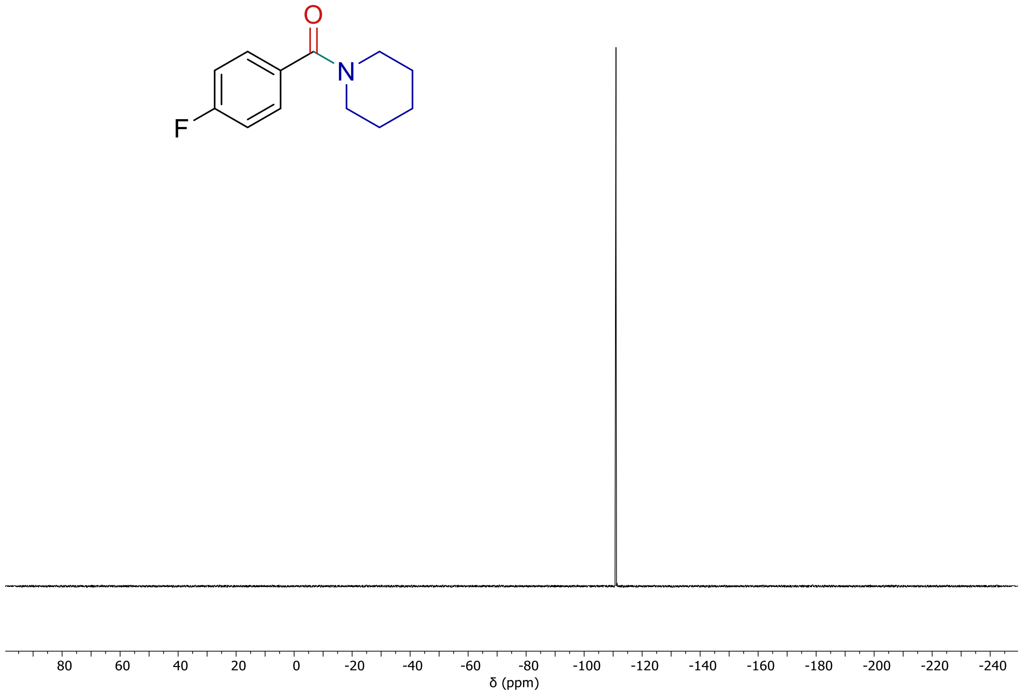

(SI-4) - Methyl 4-(piperidine-1-carbonyl)benzoate - The desired product was obtained from **Methyl 4-bromobenzoate** *via* **General Aminocarbonylation Procedure B**. The product was collected as a white solid (250 mg, >99 %). ^{1}H NMR (400 MHz, $CDCl_3$) δ 8.06 – 7.97 (m, 2H), 7.39 (d, J = 7.9 Hz, 2H), 3.86 (s, 3H), 3.65 (s, 2H), 3.22 (s, 2H), 1.72 – 1.36 (m, 6H). The desired product was also synthesized from **Methyl 4-chlorobenzoate** via **General Aminocarbonylation Procedure C** (199 mg, 80%). Physical and spectra data agreed with literature values.[9]

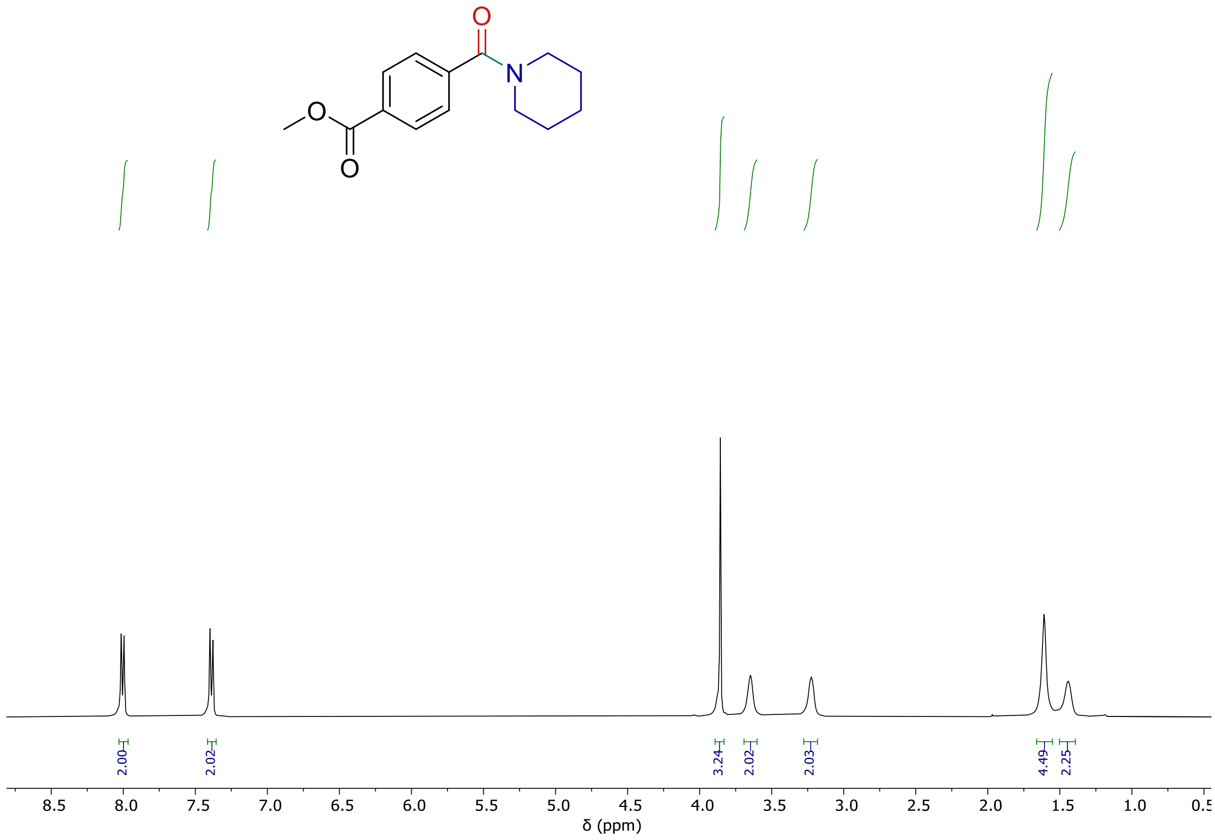

(SI-5) - Piperidin-1-yl(4-(trifluoromethyl)phenyl)methanone - The desired product was obtained from **4-bromobenzotrifluoride** *via* **General Aminocarbonylation Procedure B**. The product was collected as a white solid (246 mg, 96 %). ^1H NMR (400 MHz, $CDCl_3$) δ 7.67 (d, J = 7.8 Hz, 2H), 7.51 (d, J = 7.9 Hz, 2H), 3.73 (s, 2H), 3.30 (s, 2H), 1.69 (s, 4H), 1.52 (s, 2H). ^{19}F NMR (377 MHz, $CDCl_3$) δ -62.86. The desired product was also synthesized from **4-chlorobenzotrifluoride** via **General Aminocarbonylation Procedure C** (230 mg, 89%). Physical and spectra data agreed with literature values.[9]

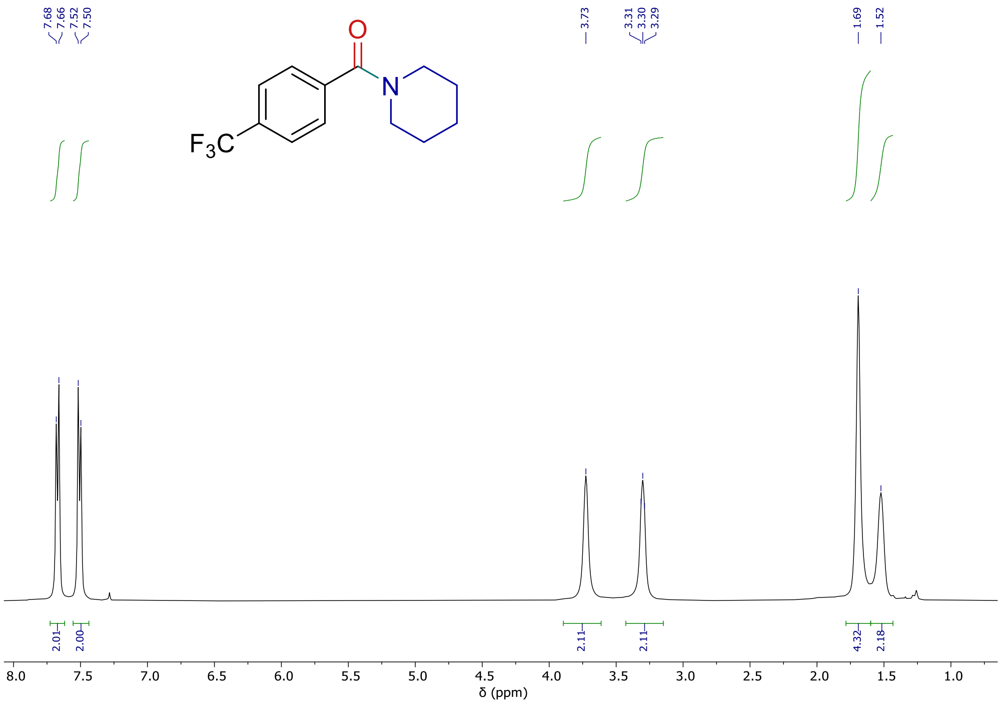

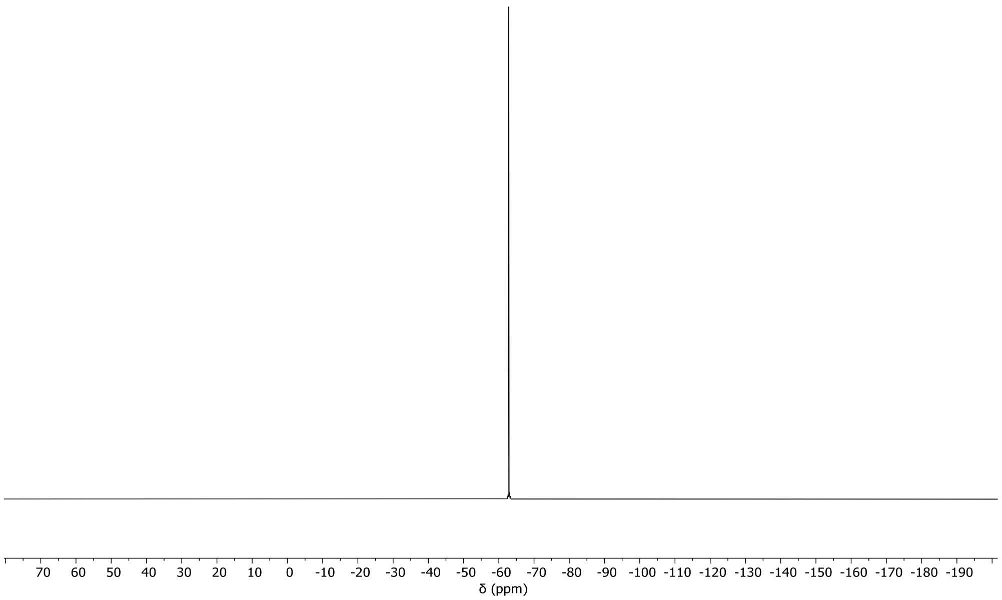

(SI-6) - 4-(piperidine-1-carbonyl)benzonitrile - The desired product was obtained from **4-bromobenzonitrile** *via* **General Aminocarbonylation Procedure B**. The product was collected as a white powder (117 mg, 55 %). ^{1}H NMR (400 MHz, $CDCl_3$) δ 7.70 (d, J = 7.9 Hz, 2H), 7.49 (d, J = 7.9 Hz, 2H), 3.71 (s, 2H), 3.28 (s, 2H), 1.69 (s, 4H), 1.52 (s, 2H). Physical and spectra data agreed with literature values.[9]

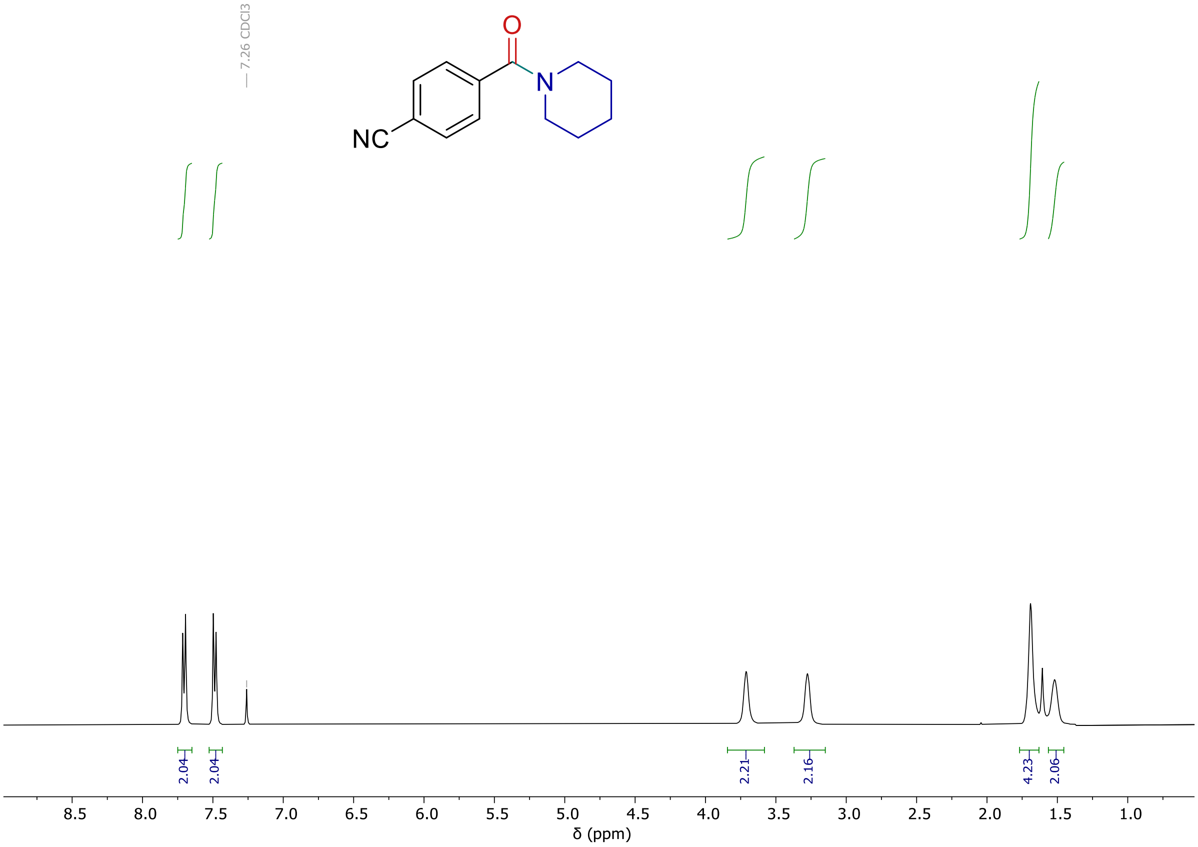

(SI-7) - Naphthalen-2-yl(piperidin-1-yl)methanone - The desired product was obtained from **2-Bromonaphthalene** *via* **General Aminocarbonylation Procedure B**. The product was collected as a white solid (198 mg, 83 %). ^1H NMR (400 MHz, $CDCl_3$) δ 7.91 – 7.82 (m, 3H), 7.56 – 7.45 (m, 3H), 7.39 (s, 1H), 3.75 (s, 2H), 3.39 (s, 2H), 1.73 – 1.63 (m, 4H), 1.54 (m, 2H). Physical and spectra data agreed with literature values.[9]

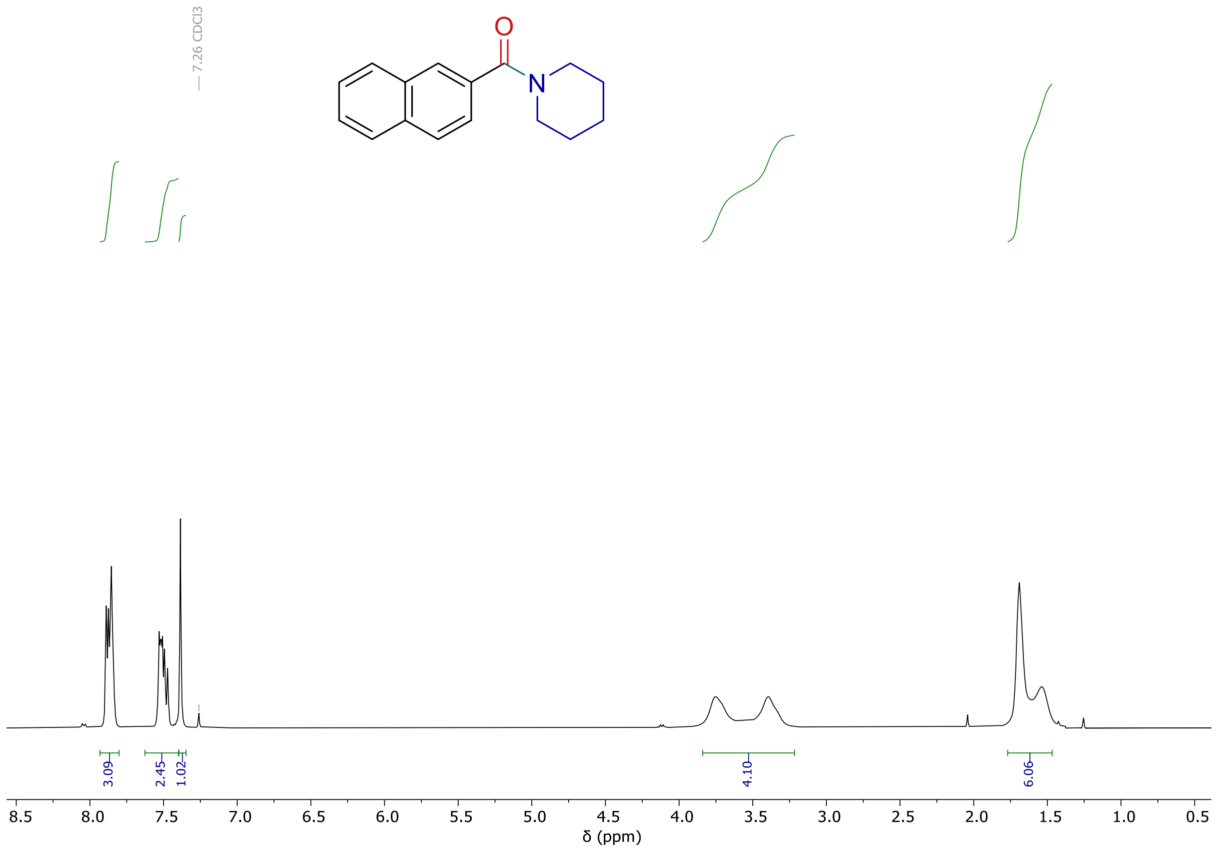

(SI-8) - Piperidin-1-yl(quinolin-6-yl)methanone - The desired product was obtained from **6-Bromoquinoline** *via* **General Aminocarbonylation Procedure B**. The product was collected as a yellow oil (244 mg, >99 %). ^{1}H NMR (400 MHz, $CDCl_3$) δ 8.94 (s, 1H), 8.17 (d, J = 8.3 Hz, 1H), 8.12 (d, J = 8.7 Hz, 1H), 7.88 (s, 1H), 7.70 (dd, J = 8.7, 1.9 Hz, 1H), 7.47 – 7.40 (m, 1H), 3.74 (br s, 2H), 3.36 (br s, 2H), 1.81 – 1.40 (m, 6H). Physical and spectra data agreed with literature values.[9]

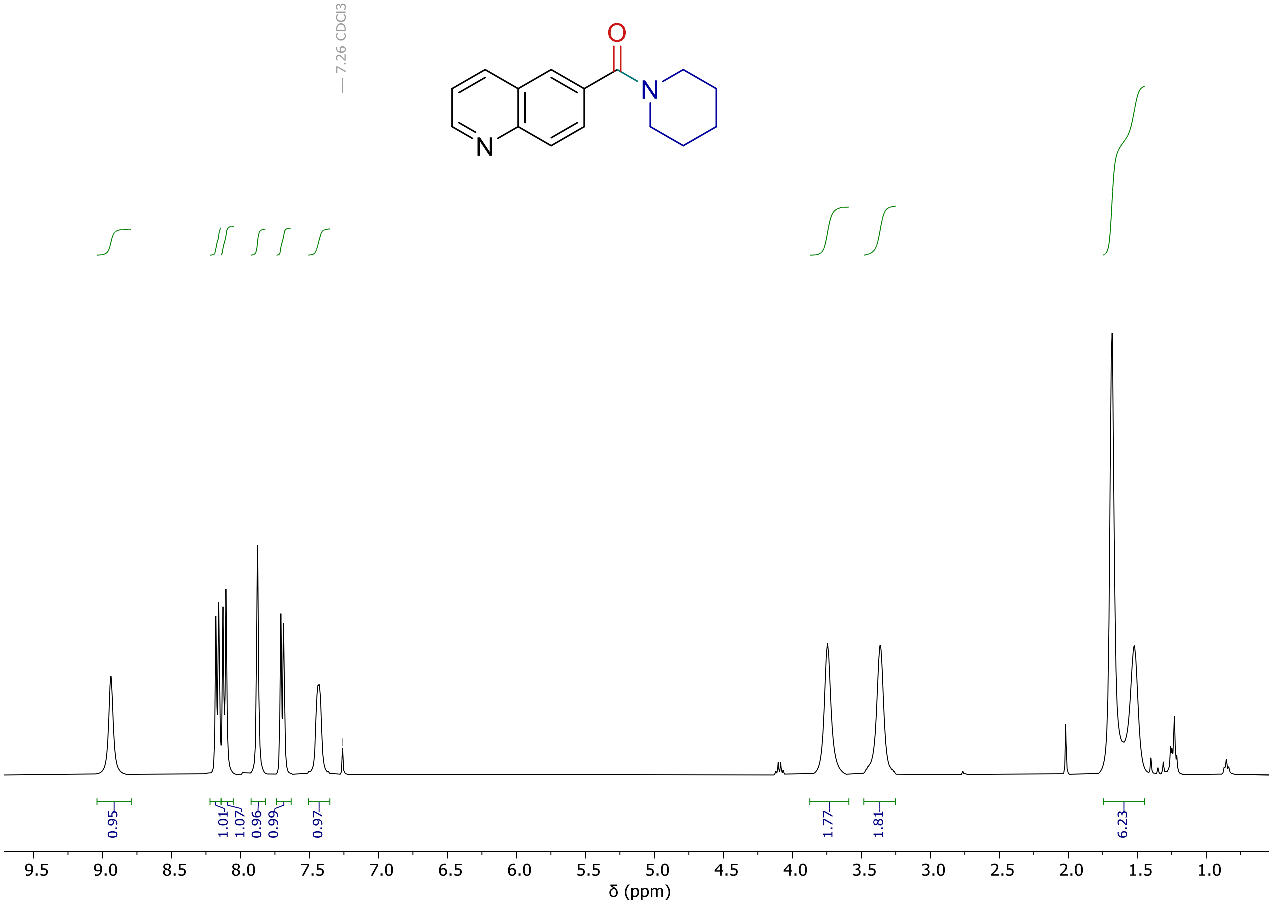

(SI-9) - Piperidin-1-yl(pyridin-3-yl)methanone - The desired product was obtained from **3-bromopyridine** *via* **General Aminocarbonylation Procedure B** with 1M NaOH in place of HCl. The product was collected as a yellow oil (189 mg, 99 %). ^{1}H NMR (400 MHz, $CDCl_3$) δ 8.62 (s, 2H), 7.71 (d, 1H), 7.33 (t, J = 4.9 Hz, 1H), 3.69 (s, 2H), 3.33 (s, 2H), 1.66 (s, 4H), 1.57 – 1.43 (m, 2H). Physical and spectra data agreed with literature values.[9]

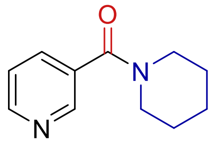

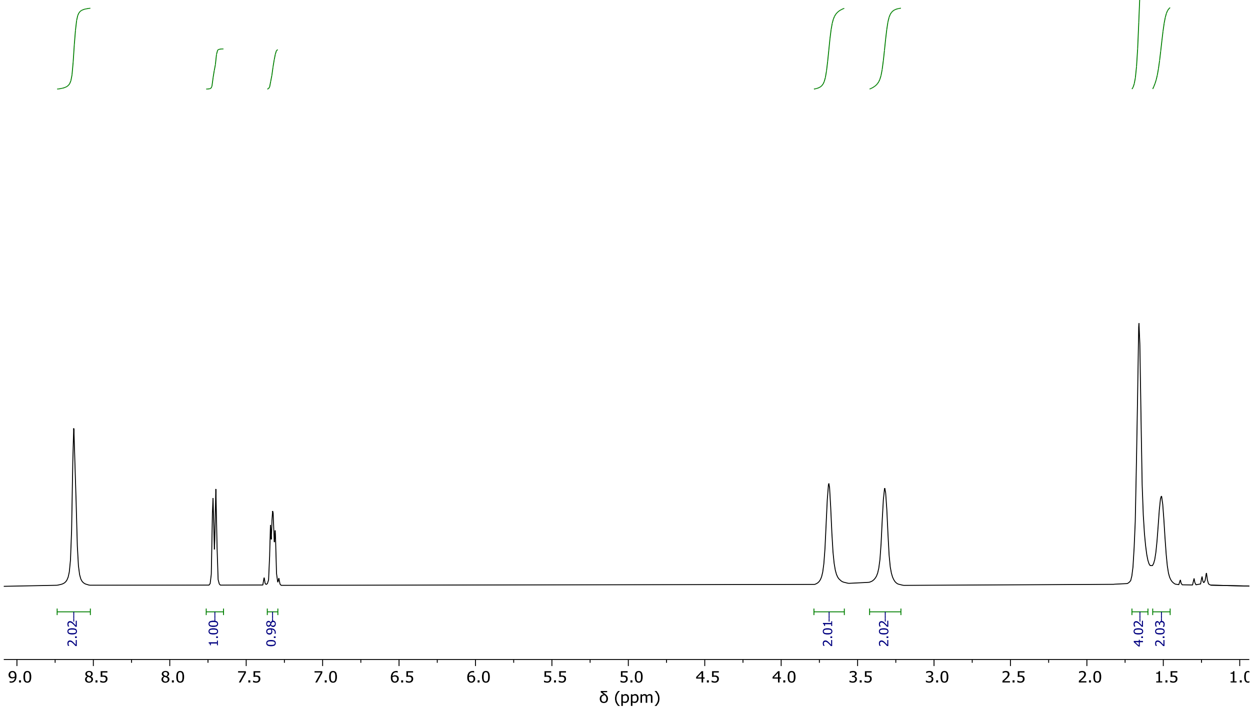

(SI-10) - N-(4-chlorobenzoyl)piperidine - The desired product was obtained from **4-Chlorobromobenzene** *via* **General Aminocarbonylation Procedure B**. The product was collected as a white solid (207 mg, 93 %). ^{1}H NMR. The product was collected as a clear oil (156 mg, 75 %). ^{1}H NMR (400 MHz, $CDCl_3$) δ 7.35 (q, J = 8.5 Hz, 4H), 3.69 (s, 2H), 3.33 (s, 2H), 1.89 – 1.34 (m, 6H). The desired product was also synthesized from **1,4-Dichlorobenzene** via **General Aminocarbonylation Procedure C** (190 mg, 85%). Physical and spectra data agreed with literature values.[10]

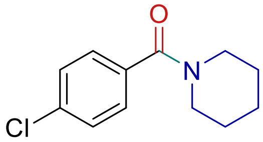

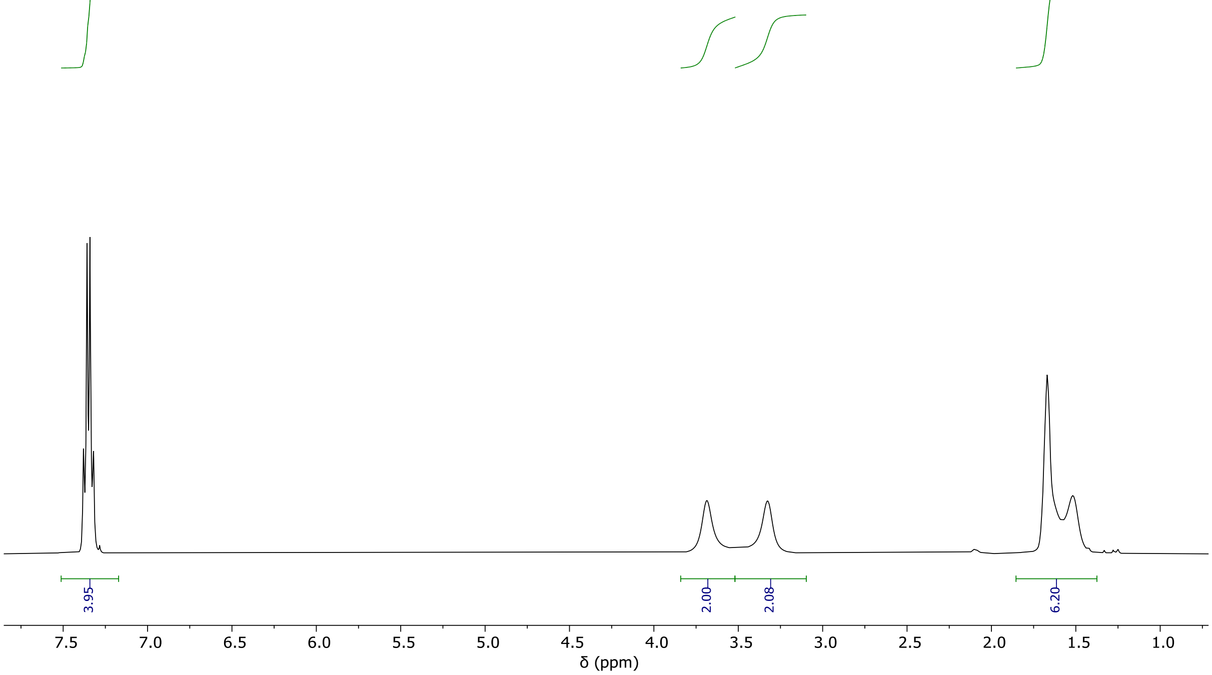

(SI-11) - Piperidin-1-yl(4-(trifluoromethoxy)phenyl)methanone- The desired product was obtained from **1-Bromo-4-(trifluoromethoxy)benzene** *via* **General Aminocarbonylation Procedure B**. The product was collected as a colourless oil (280 mg, >99%). ^{1}H NMR (400 MHz, $CDCl_3$) δ 7.46 – 7.38 (m, 2H), 7.22 (d, J = 8.2 Hz, 2H), 3.68 (s, 2H), 3.31 (s, 2H), 1.66 (q, J = 5.0 Hz, 4H), 1.51 (s, 2H). ^{19}F NMR (377 MHz, $CDCl_3$) δ -57.88. The desired product was also synthesized from **1-Chloro-4-(trifluoromethoxy)benzene** via **General Aminocarbonylation Procedure C** (244 mg, 89%). Physical and spectra data agreed with literature values.[11]

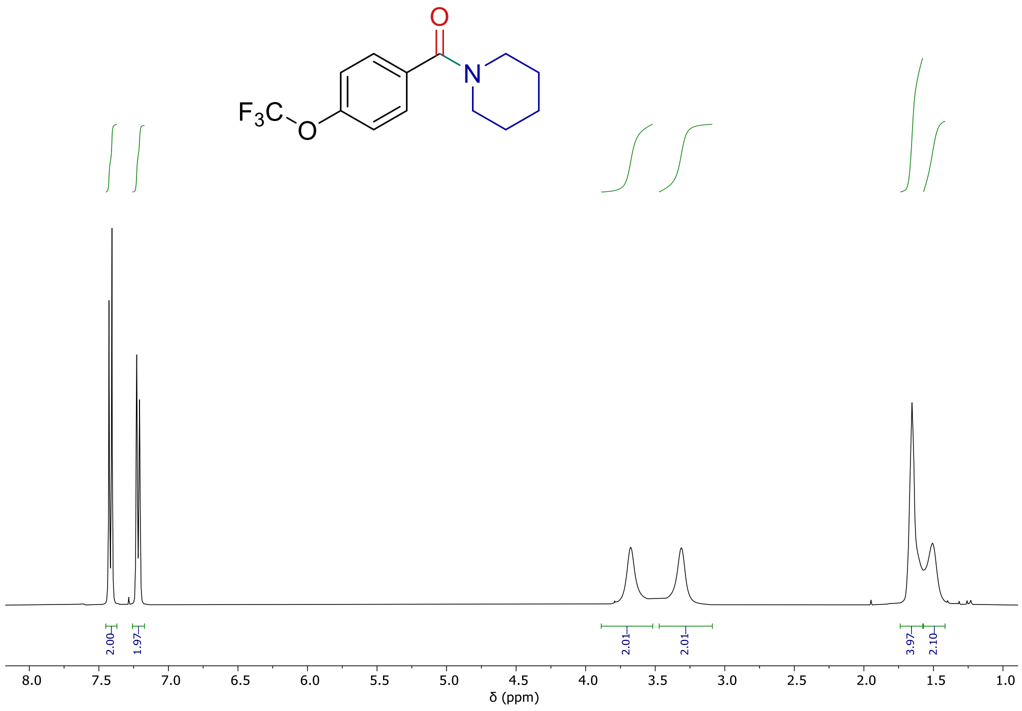

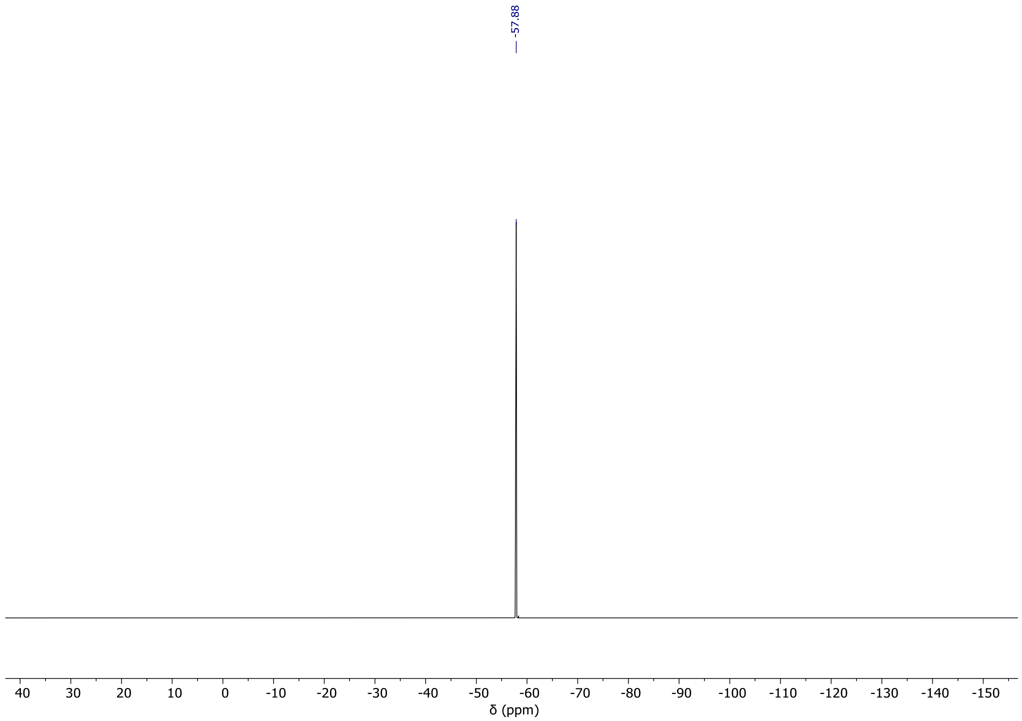

(SI-12) - 1,4-Phenylenebis(1-piperidinylmethanone) - The desired product was obtained from **1,4-Dichlorobenzene** *via* **General Aminocarbonylation Procedure C** using 0.5 eq. of ArCl. The product was collected as a white solid (193 mg, 86 %). ^{1}H NMR (400 MHz, $CDCl_3$) δ 7.41 (s, 2H), 3.71 (br s, 2H), 3.32 (br s, 2H), 1.75 – 1.42 (m, 6H). Physical and spectra data agreed with literature values.[10]

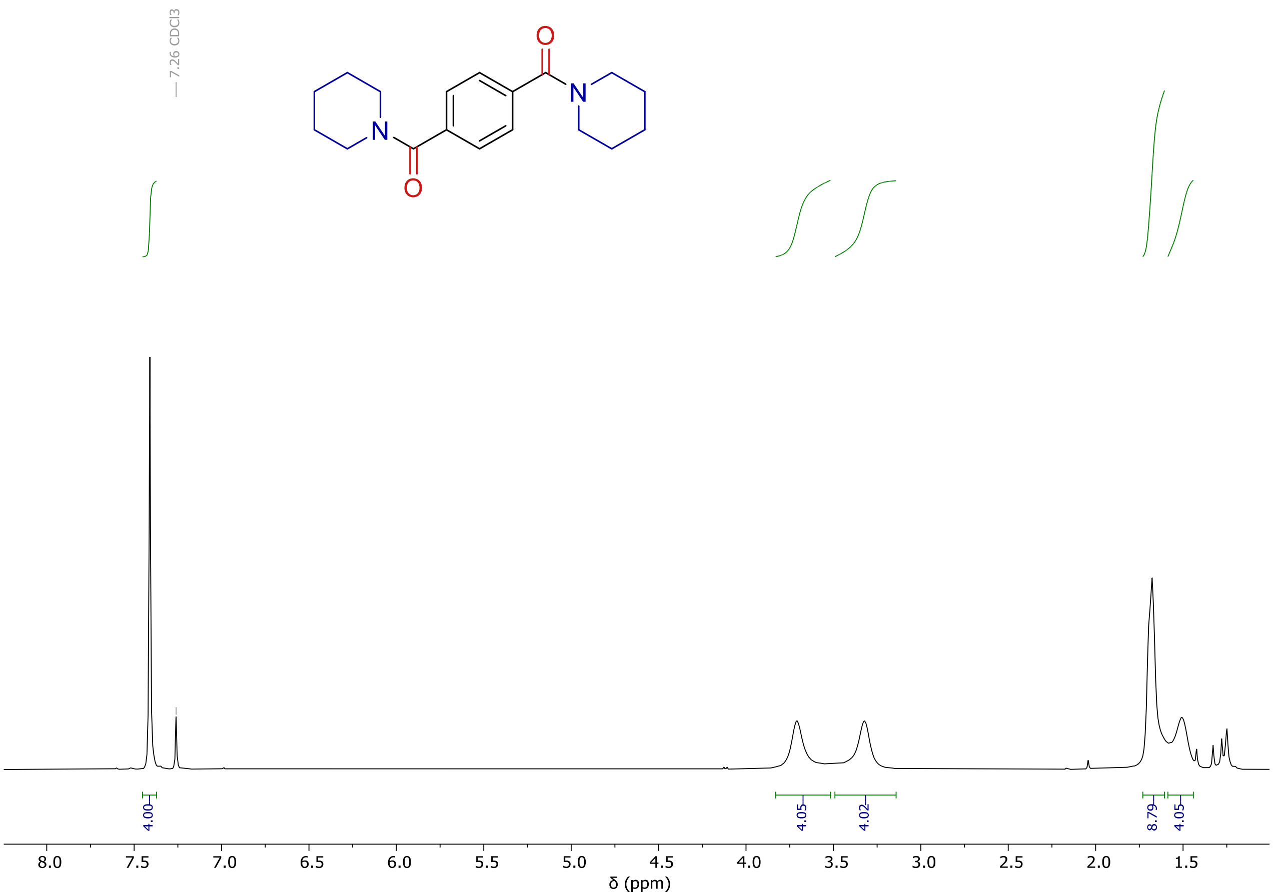

(SI-13) - N-(3,3-dimethylacryloyl)piperidine - The desired product was obtained from **Isocrotyl bromide** *via* **General Aminocarbonylation Procedure B**. The product was quantified using in-situ ^{1}H NMR relative to a trimethoxybenzene (TMB) internal standard (76%). ^{1}H NMR (400 MHz, $CDCl_3$) δ 5.59 (s, 1H), 3.34 (br s, 4H), 1.64 (s, 6H), 1.52 – 1.29 (m, 6H). Spectra data agreed with literature values.[12]

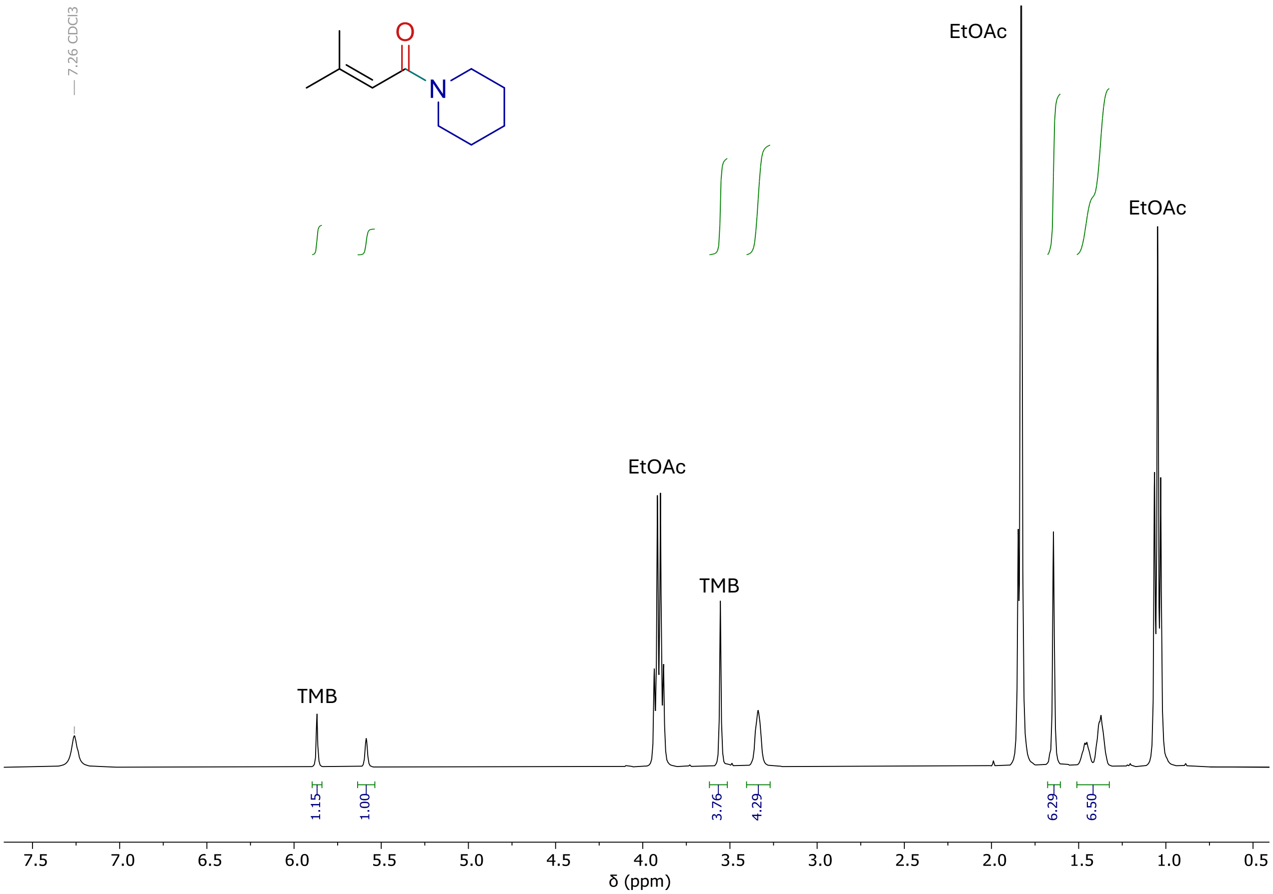

(SI-14) - 4-Fluoro-N-phenylbenzamide - The desired product was obtained from **4-fluorobromobenzene** *via* **General Aminocarbonylation Procedure B** coupled with aniline as a nucleophile. The product was quantified using in-situ ^{19}F NMR relative to a trifluorotoluene internal standard (90% yield). ^{19}F NMR (377 MHz, $CDCl_3$) δ -104.80. Spectra data agreed with literature values.[13] Crude ^{1}H NMR analysis corroborated product formation.[14]

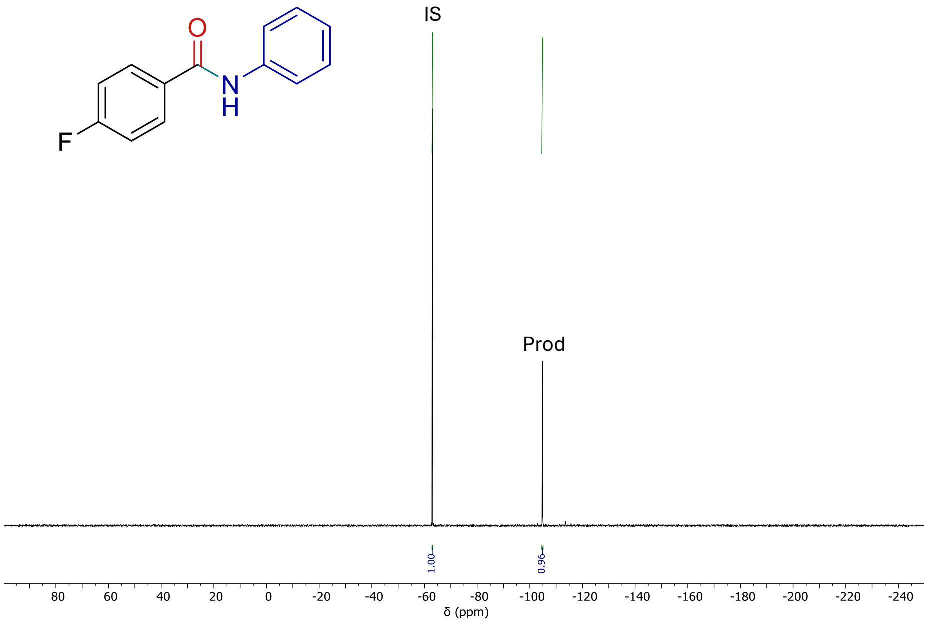

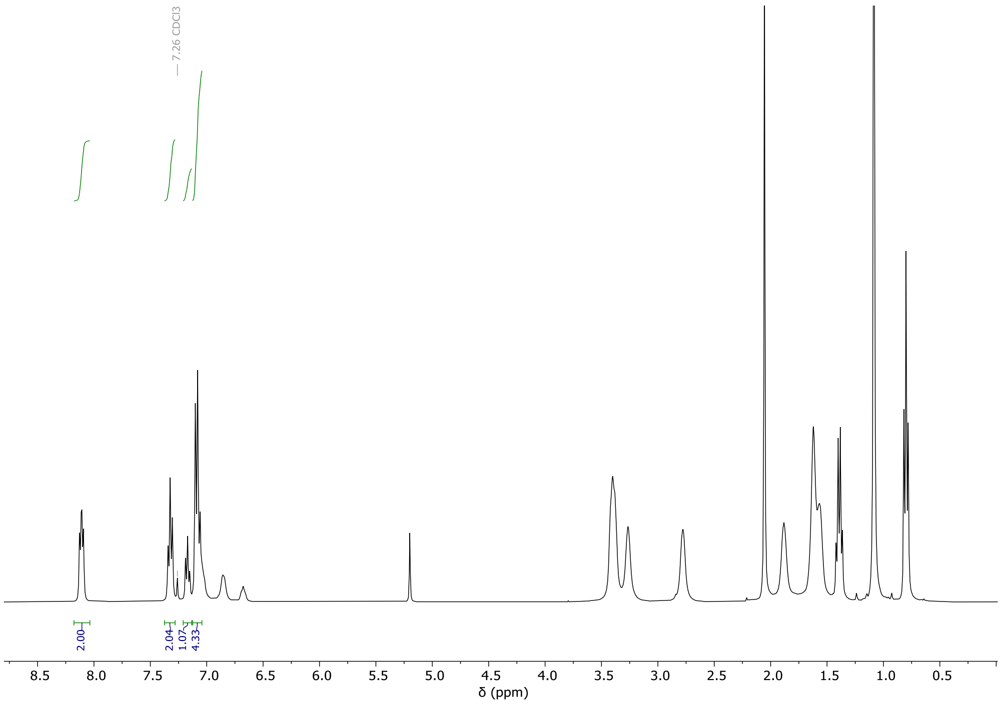

(SI-15) - Phenyl 4-fluorobenzoate - The desired product was obtained from **4-fluorobromobenzene** *via* **General Aminocarbonylation Procedure B** coupled with phenol as a nucleophile. The product quantified using in-situ ^{19}F NMR relative to a trifluorotoluene internal standard (87% yield). ^{19}F NMR (377 MHz, $CDCl_3$) δ -104.75. Spectra data agreed with literature values.[15] Crude ^1H NMR analysis corroborated product formation.[16]

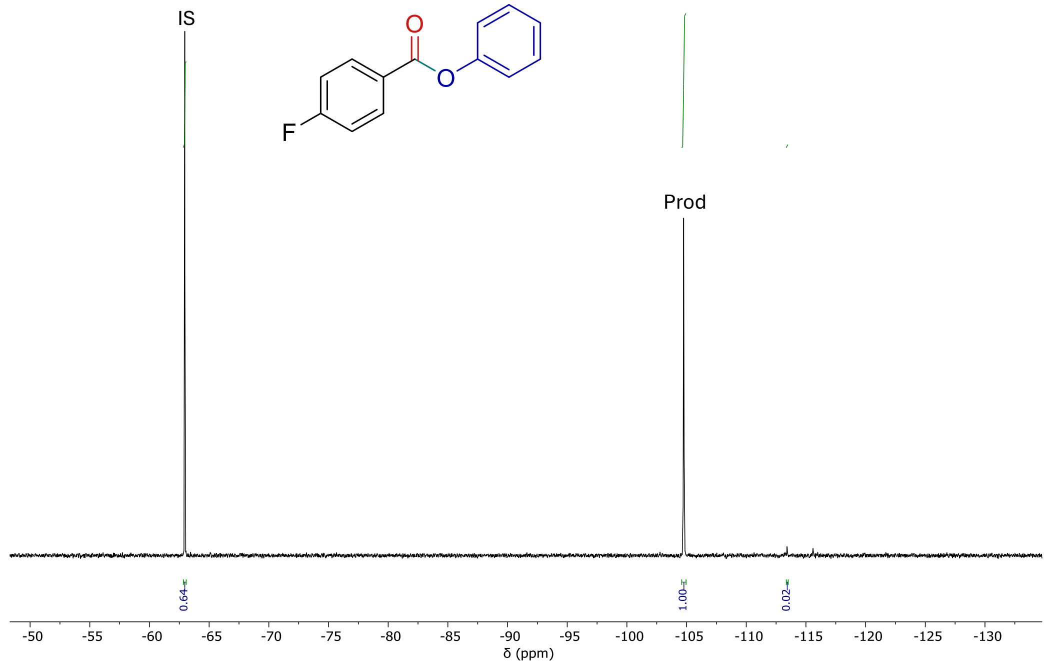

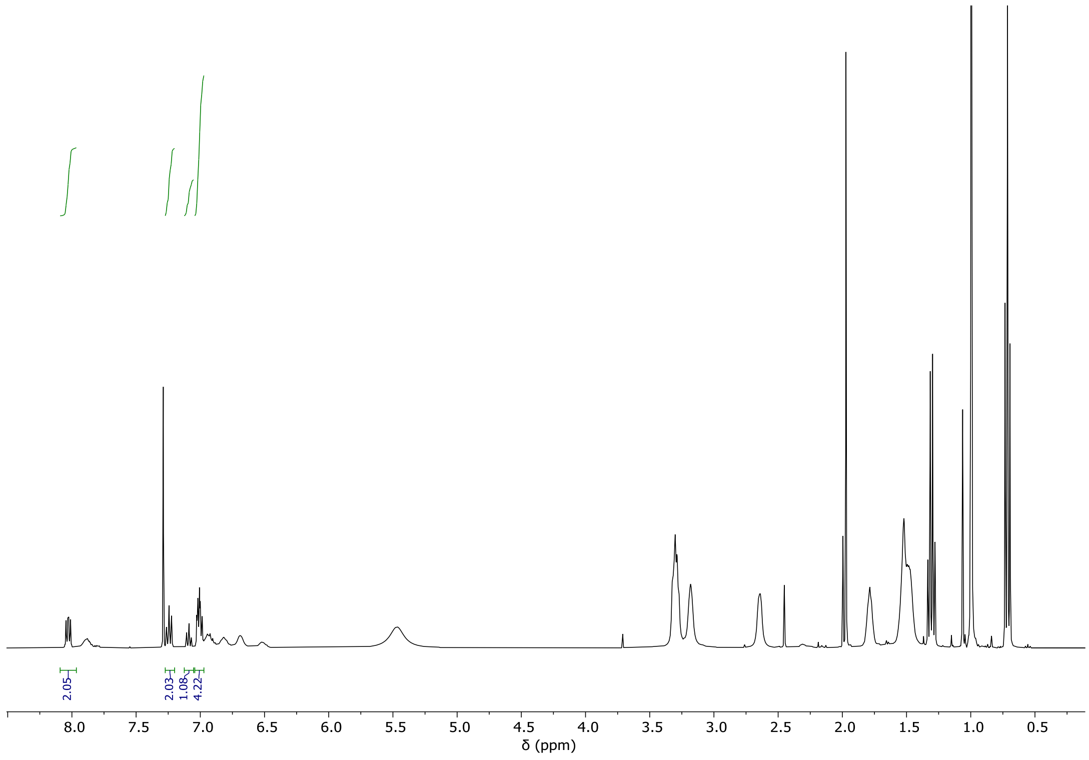

(SI-16) - Isoindolin-1-one - The desired product was obtained from **2-Bromobenzylamine** *via* **General Aminocarbonylation Procedure B** in absence of piperidine. The product was collected as a white solid (57 mg, 85 %). ^{1}H NMR (400 MHz, $CDCl_3$) δ 8.39 (s, 1H), 7.89 – 7.83 (m, 1H), 7.60 – 7.50 (m, 1H), 7.49 – 7.37 (m, 2H), 4.47 (s, 2H). Physical and spectra data agreed with literature values.[17]

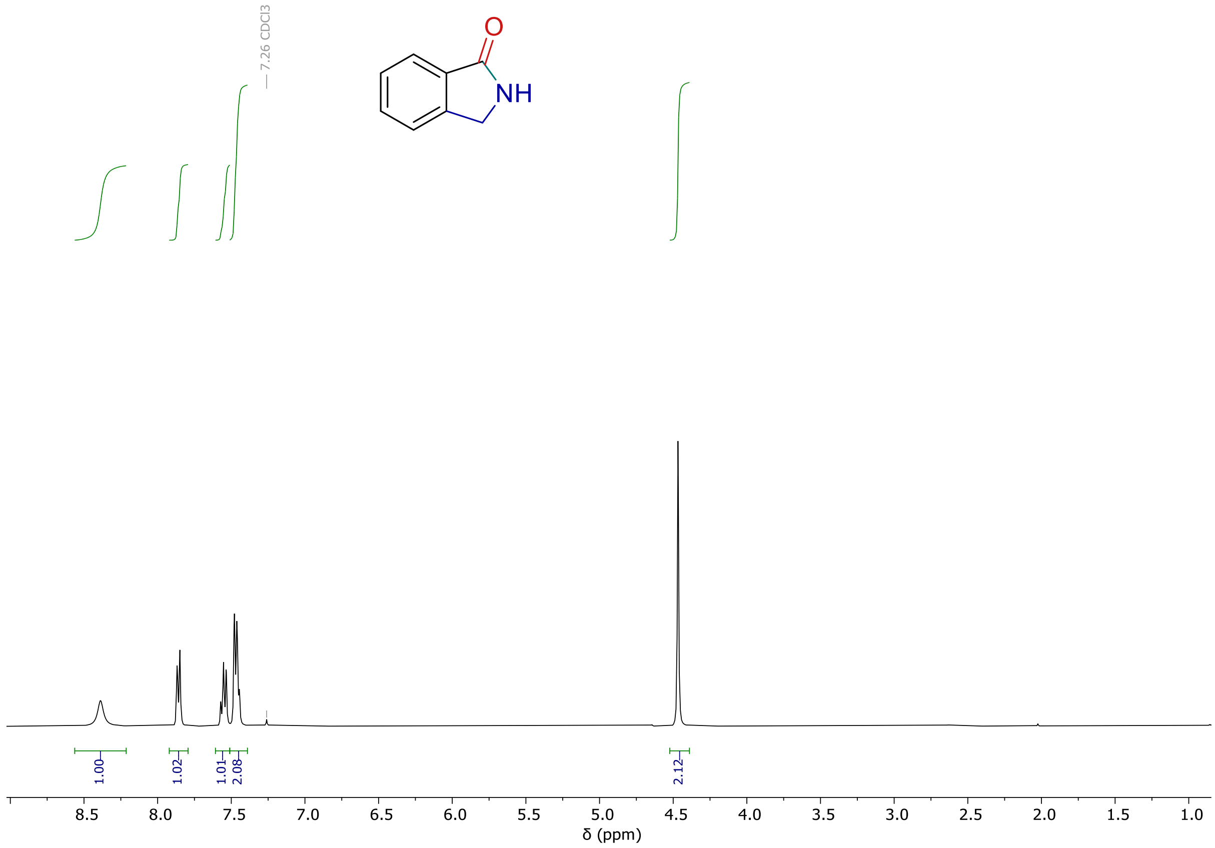

(SI-17) – 1-isobenzofuranone - The desired product was obtained from **2-Bromobenzyl alcohol** *via* **General Aminocarbonylation Procedure B** in absence of piperidine. The product was collected as a white solid (141 mg, >99 %). ^{1}H NMR (400 MHz, $CDCl_3$) δ 7.93 (d, J = 7.5 Hz, 1H), 7.70 (t, J = 7.5 Hz, 1H), 7.59 – 7.48 (m, 2H), 5.34 (s, 2H). Physical and spectra data agreed with literature values.[18]

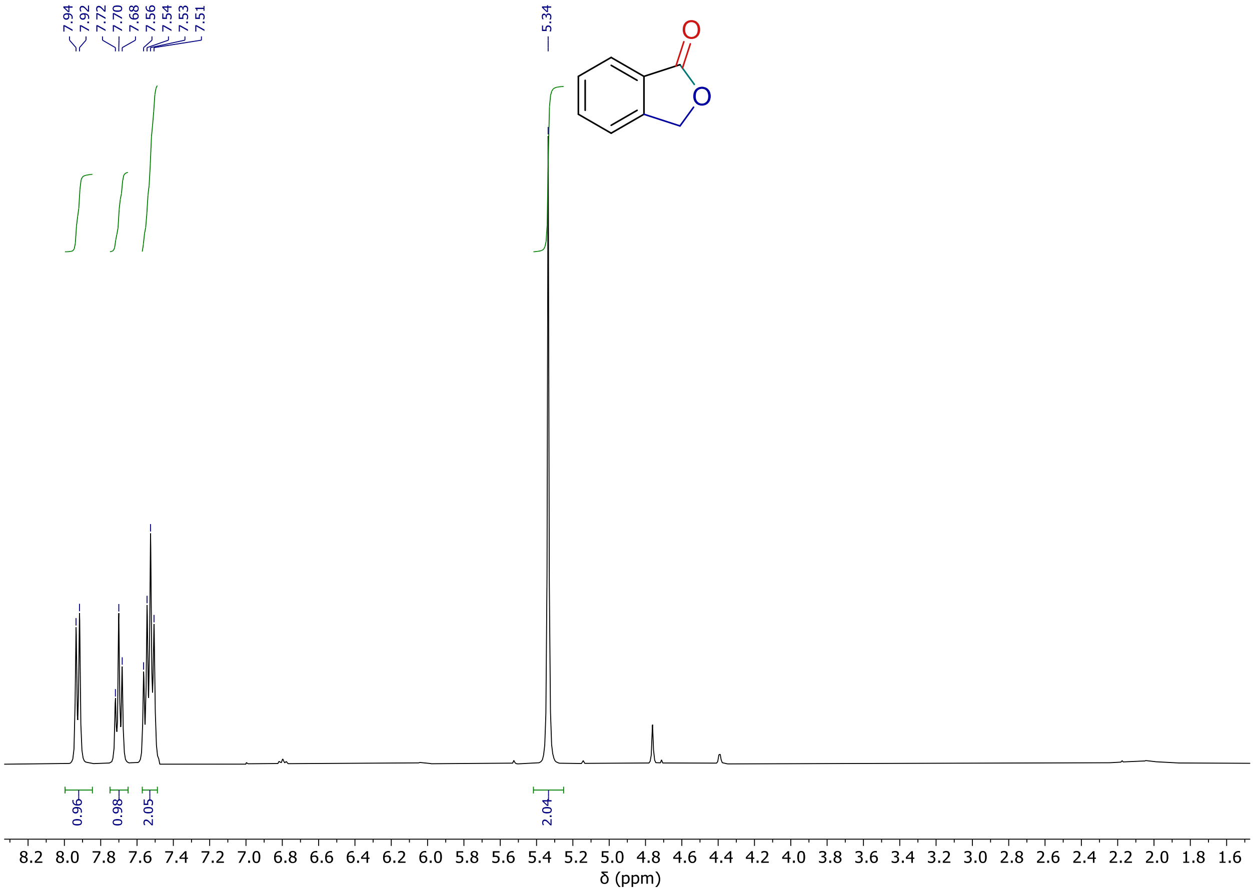

X-Ray Data

Crystal **[TMPH][Co(CO)$_4$]** was grown directly from a reaction solution of $Co_2(CO)_8$ (6.8 mg) and 2,2,6,6-tetramethylpiperidine (68 uL) in C_6D_6 (0.4 mL). The tube was subject to ^{59}Co NMR analysis, during which time it was heated to 80 °C for approximately 5 minutes. The tube was subsequently irradiated (365 nm LEDs (1 x 10W)) for 1 h and measured by NMR spectroscopy once more (80 °C for 5 minutes). Upon standing for 2 weeks, colourless XRD quality crystals were grown from the solution. The crystals were isolated in a nitrogen filled glovebox and coated with X-Ray oil for handling outside of an inert environment. ^{59}Co NMR (118 MHz, C_6D_6) δ -2985 (bs).

We believe that synthesis occurs *via* deprotonation of an additional equivalent of TMP, for which there was an excess available in solution. The proposed route is based on prior literature.

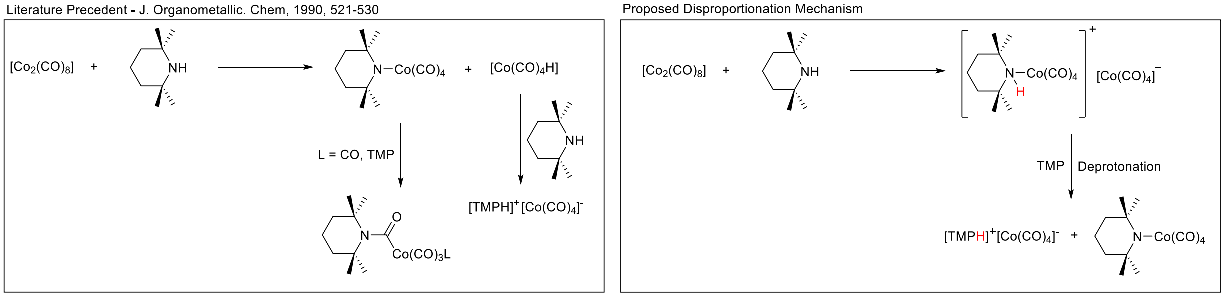

Figure S86: Proposed synthetic pathways to furnish synthesis of [TMPH][Co(CO)$_4$] complex

Data were collected using an Agilent Xcalibur 3 E diffractometer, and the structures were solved and refined using the OLEX2[19], SHELXTL and SHELX-2013[20, 21], program systems. $R_1 = \Sigma||F_o| - |F_c||/\Sigma|F_o|$; $wR_2 = [\Sigma[w(F_o^2 - F_c^2)^2] / \Sigma[w(F_o^2)^2]]^{1/2}$; $w^{-1} = \sigma_2(F_o^2) + (aP)^2 + bP$.

[TMPH][Co(CO)$_4$]: CCDC number: 2465848, formula: $[C_9H_{20}N][C_4CoO_4]$, formula weight: 313.23, colour, habit: colourless column, crystal size 0.334 x 0.299 x 0.196 mm, crystal system: monoclinic, space group: $P2_1/c$ (no. 14), a = 9.6454(2) Å, b = 11.0423(2) Å, c = 14.3289(3) Å, α = 90 °, β = 91.725(2) °, γ = 90 °, V = 1525.44(5) Å^3, Z = 4, T = 173.05(10) K, D_c = 1.364 g·cm^{-3}, radiation used: Mo Kα, μ = 1.133 mm^{-1}, F(000) = 656, absorption correction: analytical, min-max transmission: 0.774 - 0.855, Θ range for data collection: 2.805 - 28.338 °, no. of unique reflns measured: 3457, obs [F > 4σ(F)]: 2934, R_{int} = 0.0342, R_{sigma} = 0.0247, completeness to Θ(full): 0.999 to 25.242 °, no. of parameters: 184, restraints: 0, R_1 [F > 4σ(F)] = 0.0311, wR_2 [F > 4σ(F)] = 0.0771, R_1 [all data] = 0.0414, wR_2 [all data] = 0.0824, GooF = 1.083, largest diff. Fourier peak: 0.600 eÅ^{-3}, hole: -0.278 eÅ^{-3}.

The two N–H hydrogens H1 and H2 were found in the Δ*F* maps and freely refined.

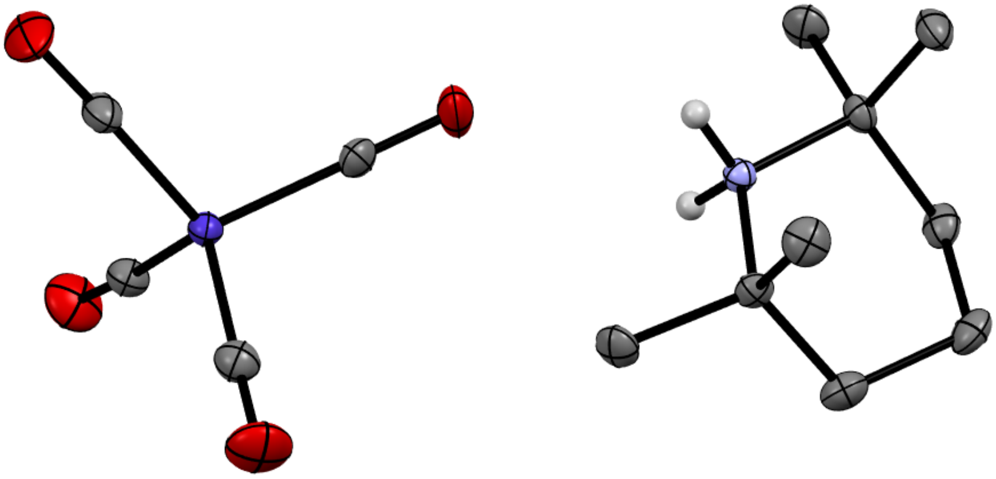

*Figure S87: X-ray structure of **[TMPH][Co(CO)$_4$]**. Most hydrogen atoms are omitted for clarity.*

References

(1) Keenan, M.; Abbott, M. J.; Alexander, P. W.; Armstrong, T.; Best, W. M.; Berven, B.; Botero, A.; Chaplin, J. H.; Charman, S. A.; Chatelain, E.; et al. Analogues of Fenarimol Are Potent Inhibitors of Trypanosoma cruzi and Are Efficacious in a Murine Model of Chagas Disease. *J. Med. Chem.* **2012**, *55* (9), 4189-4204. DOI: 10.1021/jm2015809.

(2) Xie, W.; Zhao, M.; Cui, C. Cesium Carbonate-Catalyzed Reduction of Amides with Hydrosilanes. *Organometallics* **2013**, *32* (24), 7440-7444. DOI: 10.1021/om400951n.

(3) Ellis, J. E.; Barger, P. T.; Winzenburg, M. L.; Warnock, G. F. Highly reduced organometallics XXVII. Synthesis, isolation and characterization of trisodium tricarbonylcobaltate(3 -), and initial studies on its derivative chemistry. *J. Organomet. Chem.* **1990**, *383* (1), 521-530. DOI: https://doi.org/10.1016/0022-328X(90)85151-N.

(4) Carpenter, A. E.; Chan, C.; Rheingold, A. L.; Figueroa, J. S. A Well-Defined Isocyano Analogue of HCo(CO)4. 2: Relative Brønsted Acidity as a Function of Isocyanide Ligation. *Organometallics* **2016**, *35* (14), 2319-2326. DOI: 10.1021/acs.organomet.6b00375.

(5) Müller, D.; Alexakis, A. Copper-Catalyzed Asymmetric 1,4-Addition of Alkenyl Alanes to N-Substituted-2-3-dehydro-4-piperidones. *Organic Letters* **2012**, *14* (7), 1842-1845. DOI: 10.1021/ol3004436.

(6) Tzaras, D.-I.; Gorai, M.; Jacquemin, T.; Arndt, T.; Zimmermann, B. M.; Breugst, M.; Teichert, J. F. Site-Selective Copper(I)-Catalyzed Hydrogenation of Amides. *Journal of the American Chemical Society* **2025**, *147* (2), 1867-1874. DOI: 10.1021/jacs.4c14174.

(7) Liu, J.; Zhao, T.; Li, J.-R.; Zhang, H.-W.; Zhang, T.; Zou, Z.-M. Eight new amide alkaloids from Piper Longum and their anti-inflammatory activities. *Fitoterapia* **2025**, *186*, 106804. DOI: https://doi.org/10.1016/j.fitote.2025.106804.

(8) Pierson, C. N.; Hartwig, J. F. Mapping the mechanisms of oxidative addition in cross-coupling reactions catalysed by phosphine-ligated Ni(0). *Nature Chemistry* **2024**, *16* (6), 930-937. DOI: 10.1038/s41557-024-01451-x.

(9) Veatch, A. M.; Alexanian, E. J. Cobalt-catalyzed aminocarbonylation of (hetero)aryl halides promoted by visible light. *Chem. Sci.* **2020**, *11* (27), 7210-7213, 10.1039/D0SC02178D. DOI: 10.1039/D0SC02178D.

(10) Annereau, M.; Salmain, M.; Corcé, V. Photoinduced CO-releasing molecule (photoCORM) as an in situ CO surrogate for palladium-catalysed aminocarbonylation. *Chem. Commun.* **2024**, *60* (29), 3934-3937, 10.1039/D4CC00524D. DOI: 10.1039/D4CC00524D.

(11) Castral, T. C.; Matos, A. P.; Monteiro, J. L.; Araujo, F. M.; Bondancia, T. M.; Batista-Pereira, L. G.; Fernandes, J. B.; Vieira, P. C.; da Silva, M. F. G. F.; Corrêa, A. G. Synthesis of a Combinatorial Library of Amides and Its Evaluation against the Fall Armyworm, Spodoptera frugiperda. *J. Agric. Food Chem* **2011**, *59* (9), 4822-4827. DOI: 10.1021/jf104903t.

(12) Takamatsu, M.; Sekiya, M. Reactions of 1-Trichloromethyl-substituted Amines with Potassium tert-Butoxide. *Chem. Pharm. Bull.* **1980**, *28* (10), 3098-3105. DOI: 10.1248/cpb.28.3098.

(13) Yang, Y.; Yu, X.; He, N.; Huang, X.; Song, X.; Chen, J.; Lin, J.; Jin, Y. FeCl3-catalyzed oxidative amidation of benzylic C–H bonds enabled by a photogenerated chlorine-radical. *Chem. Commun.* **2023**, *59* (68), 10299-10302, 10.1039/D3CC03186A. DOI: 10.1039/D3CC03186A.
(14) Wang, Q.-D.; Liu, X.; Zheng, Y.-W.; Wu, Y.-S.; Zhou, X.; Yang, J.-M.; Shen, Z.-L. Iron-Mediated Reductive Amidation of Triazine Esters with Nitroarenes. *Org. Lett.* **2024**, *26* (1), 416-420. DOI: 10.1021/acs.orglett.3c04180.
(15) Liu, Y.; Shirai, Y.; Okada, I.; Ohmura, R.; Liang, F.; Tsuda, A. Flow Photo-on-Demand Synthesis of Vilsmeier Reagent and Acyl Chlorides from Chloroform and Its Applications to Continuous Flow Synthesis of Carbonyl Compounds. *Org. Process Res. Dev.* **2024**, *28* (5), 1632-1639. DOI: 10.1021/acs.oprd.3c00267.
(16) Guha, S.; Crochet, A.; Sukumar, T.; Ravva, M. K.; Sen, S.; Gremaud, L. Unlocking Indazole Synthesis from α-Diazo-β-Ketoesters via Aryne Trapping: A Streamlined Approach. *Eur. J. Org. Chem.* *n/a* (n/a), 2500296. DOI: https://doi.org/10.1002/ejoc.202500296.
(17) Yang, D.; Xu, H.; Huang, D.; Zhao, H. Rhodium(III)-Catalyzed Intramolecular Benzylic C(sp3)–H Amidation for the Synthesis of Isoindolinones. *Synthesis* **2022**, *55* (03), 481-488. DOI: 10.1055/a-1954-4920.
(18) Zhai, H.; Wei, Z.; Jing, X.; Duan, C. A Porphyrin-Faced Zn8L6 Cage for Selective Oxidation of C(sp3)–H Bonds and Sulfides. *Inorg. Chem.* **2024**, *63* (31), 14375-14382. DOI: 10.1021/acs.inorgchem.4c01009.
(19) Dolomanov, O. V.; Bourhis, L. J.; Gildea, R. J.; Howard, J. A. K.; Puschmann, H. OLEX2: a complete structure solution, refinement and analysis program. *J. Appl. Crystallogr.* **2009**, *42* (2), 339-341. DOI: doi:10.1107/S0021889808042726.
(20) Sheldrick, G. M. SHELXT - Integrated space-group and crystal-structure determination. *Acta Crystallogr. Sect. A Found. Adv.* **2015**, *71* (1), 3-8. DOI: doi:10.1107/S2053273314026370.
(21) Sheldrick, G. M. Crystal structure refinement with SHELXL. *Acta Crystallogr. Sect. C Struct. Chem.* **2015**, *71* (Pt 1), 3-8. DOI: 10.1107/s2053229614024218 From NLM.
